# 21st Congress of the European Burns Association (EBA)

**DOI:** 10.3390/ebj6030049

**Published:** 2025-09-03

**Authors:** Nadia Depetris, Alette E. E. de Jong, Jill Meirte, Thomas Leclerc, Jose Ramon Martinez Mendez, Clemens Schiestl, Frank Siemers, Andy Williams, Paul P. M. van Zuijlen, Jyrki Vuola, Stian Almeland, Luís Cabral, Bernd Hartmann

**Affiliations:** 1 Burn Center, CTO Hospital, Città Della Salute e Della Scienza, 10126 Turin, Italy; 2 Burn Center, Rode Kruis Ziekenhuis, 1942 LE Beverwijk, The Netherlands; 3 Department of Rehabilitation Sciences and Physiotherapy, REVAKI-MOVANT, Faculty of Medicine and Health Sciences, University of Antwerp, 2610 Wilrijk, Belgium; 4 Oscare, Organisation for Burns, Scar After-Care and Research, 2170 Antwerp, Belgium; 5 Percy Military Teaching Hospital, 92140 Clamart, France; 6 Val-de-Grâce Military Medical Academy, 75005 Paris, France; 7 Burn Unit, Hospital Universitario La Paz, 28046 Madrid, Spain; 8 Department of Surgery, University Children’s Hospital, 8032 Zürich, Switzerland; 9 Department of Plastic, Hand Surgery and Burn Care, BG Klinikum Bergmannstrost, 06112 Halle, Germany; 10 Burns Unit, Chelsea and Westminster Hospital NHS Foundation Trust, London SW10 9NH, UK; 11 Department of Plastic Reconstructive and Hand Surgery, Movement Sciences (AMS) Institute, Amsterdam UMC, Location VUmc, 1081 HZ Amsterdam, The Netherlands; 12 Burn Center and Department of Plastic and Reconstructive Surgery, Red Cross Hospital, 1942 LE Beverwijk, The Netherlands; 13 Association of Dutch Burn Centers (ADBC), 1941 AJ Beverwijk, The Netherlands; 14 Paediatric Surgical Center, Emma Children’s Hospital, Amsterdam UMC, 1105 AZ Amsterdam, The Netherlands; 15 Helsinki Burn Centre, Department of Plastic Surgery, Jorvi Hospital, Helsinki University Hospital, University of Helsinki, Karvasmäentie 8, 02740 Espoo, Finland; 16 Department of Plastic, Hand and Reconstructive Surgery, Norwegian National Burn Center, Haukeland University Hospital, NO-5021 Bergen, Norway; 17 Department of Clinical Medicine, University of Bergen, NO-5021 Bergen, Norway; 18 Department of Plastic Surgery and Burns Unit, Coimbra University Hospital Centre (CHUC), 3000-075 Coimbra, Portugal; 19 Burn Center, Department of Plastic Surgery, BG Klinikum Unfallkrankenhaus Berlin, 12683 Berlin, Germany

**Keywords:** burns, burn care, burn center

## Abstract

Abstracts of the plenary and special interest sessions, workshops, and oral and poster presentations of the 21st EBA Congress in Berlin, Germany, from 3 to 6 September 2025.

## 1. Introduction

We are proud to present the Conference Report of the 21st European Burns Association (EBA) Congress (3–6 September 2025, Berlin, Germany). We have designed a program featuring diverse sessions, interactive workshops, and unique networking events, all dedicated to fostering collaboration and advancing the standards of burn care together.

More than just a conference, the EBA Congress serves as a vital multidisciplinary forum. It is a place where burn care experts and researchers from across Europe and beyond gather to exchange crucial insights, cultivate innovative ideas, and forge powerful collaborations that drive progress in burn treatment and recovery. We firmly believe that our collective action and mutual support are essential for enhancing patient care. This unified approach empowers burn care professionals to champion policy changes at national and international levels, secure increased funding, and amplify public awareness of burn injuries. Such advocacy is vital for ensuring access to excellent resources, state-of-the-art facilities, and robust support systems for burn patients and their families, enabling prompt and effective responses during large-scale emergencies.

At the heart of the entire EBA Congress program is our guiding theme, “A Vision for the future.” This principle embraces a multifaceted approach to burn care, inviting us to look beyond immediate survival and collectively embrace a holistic, progressive outlook in both the European and global contexts. This shared vision involves not only continuous advancements in medical and surgical techniques but also a profound commitment to the emotional, psychological, and social well-being of burn survivors. It includes pioneering new prevention strategies to reduce the incidence of injuries, fostering equitable access to high-quality burn care across diverse regions, and strengthening international collaborations for knowledge exchange and disaster preparedness. Our shared vision of the future also addresses the evolving challenges of global health, such as the impact of climate change on injury patterns and the critical need for resilient healthcare systems capable of responding to mass casualty events. By collectively striving for these comprehensive goals, we as healthcare professionals and support networks play a pivotal role in facilitating the complete journey to recovery for burn survivors, ensuring they can rediscover their purpose and fully reintegrate into society.

Attendees can anticipate a dynamic preliminary program featuring an exciting array of sessions, including

Plenary Sessions: Our plenary sessions will delve into critical subjects such as the “Prevention of burn injuries”, the nuanced aspects of “Intimacy, sexuality and body image after burn injuries”, a crucial discussion on “Are we going backwards? The future of treatment in children with severe burns”, and the vital topic of “Sustainability in burn care”, addressing environmental considerations and long-term resource management in the field. Additionally, we will explore innovations in “Skin Engineering” and examine “How we see the future” through the lenses of international experts and emerging young professionals.

Workshops: Participate in engaging workshops, including interactive sessions on “Beyond the Manuscript: Open Insights into Academic Publishing”, practical insights into “Camouflage and Intimacy/sexuality”, the collaborative “Rehabilitation Relay: Building the Strongest Burn Recovery Plans Together”, and essential discussions on “Ethics in burn care” and “Burn care in austere conditions.”

Special Events: We warmly encourage attendance at our special events, such as the “Welcome to EBA: Breakfast” on Thursday, September 4th. This is a unique opportunity for first-time attendees and new members to connect directly with EBA ExCo members and various committees, fostering a strong sense of community from day one.

We are also deeply honored to announce the distinguished speaker for this year’s prestigious Rudy Hermans Lecture, Yvonne Wilson. A preeminent specialist in pediatric burn care, her profound commitment to addressing severe burns in children has left an indelible mark on the field. Her invaluable insights are certain to inspire all of us dedicated to advancing burn care.

## 2. Acknowledgments

Thanks are due to all the EBA Committees cooperating to put together this meeting. The assistance of all the staff members of Congress Care and the Editorial Office of the *European Burns Journal* in preparing this congress is gratefully recognized. All the industries and companies which supported this congress are also appreciatively acknowledged. Moreover, deep gratitude must be shown to all the professionals, researchers, burn survivors’ associations, and family members who actively and incessantly work to improve burn care.

## 3. Plenary and Special Interest Sessions

***Wednesday, 3 September, 09:00–10:30***

**Plenary session 1 Interactive Educational Course: Clinical Challenges in Burns and Infection**

**Moderators Dominique Potokar (France), Thomas Leclerc (France)**

Speaker: Denys Surkov

The primary objective of this session is to address the critical issues surrounding infectious complications in patients with burn injuries, particularly those arising from nosocomial infections caused by multidrug-resistant (MDR) microbial strains. These pathogens represent one of the most pressing challenges in contemporary burn care, contributing significantly to increased morbidity and mortality within this vulnerable patient cohort. Key topics to be explored include infection control strategies in burn units, antimicrobial stewardship and optimization of antibiotic therapy, preventive approaches to mitigate the onset and progression of sepsis in burn patients.

A multidisciplinary panel of experts will lead the discussion, with active participation encouraged from all relevant healthcare professionals and early-career physicians with an interest in infectious disease management and critical care. The session will critically examine questions such as: Are we equipped to combat MDR microbial isolates effectively? What are the predominant sources and transmission pathways of infection in burn centers? How can multidisciplinary teamwork dramatically improve infection control? Should we prioritize strict patient isolation and advanced environmental control technologies, or adopt a more open model of intensive care that includes family involvement in patient care—even within ICU settings? How to adjust our strategies to local situations? These complex and multifaceted challenges will be analyzed during the panel discussion, with the aim of identifying the most efficacious strategies for infection prevention and control, and establishing key priorities to enhance the quality of care for burn patients.

***Wednesday, 3 September 12:00–13:00***

**Plenary session 2: Prevention of burn injuries**

This plenary session on Prevention of Burns aims to highlight the magnitude of the problem, why we need Prevention of Burns as a priority NOW and the way forward. The mother of a child and a young person will share their challenges and resilience of lived experience of burns. The short presentations from the panel members will discuss why it is not all gloom and doom! We have a very interesting panel from WHO, public health, NGOs and Burn care professionals, all discussing collective roles in prevention of burns. We look forward to your interactions at this session and building the next steps. Together we can make a difference.

***Wednesday, 3 September 14:00–15:00***

**Plenary session 3: Intimacy, sexuality and body image after burn injuries**

**Moderators Stefania Simone, Jill Meirte**

Speakers: Jonathan Bayuo, Sabrina Belemkasser, Ina Heggland and Silje Martina Hammersmark-Kvernsveen

This plenary session will address the critical and often overlooked aspects of intimacy, sexuality, and body image in burn survivors. Featuring insights from leading experts in burn care, psychology, and personal stories from burn survivors, the session aims to provide a comprehensive understanding of the challenges and strategies for supporting patients in these areas. Interactive elements, including live Q&A and interactive discussions, will ensure an engaging and participatory experience for all attendees.

The aim of this session is to enhance the knowledge and skills of healthcare professionals in addressing the intimate and psychological needs of burn survivors. By fostering an open dialogue and sharing practical tools and resources, we hope to improve patient care and support the holistic recovery of burn survivors.

We look forward to your participation in this important and impactful session.

***Wednesday, 3 September, 17:15–18:45***

**Plenary session 4 How we see the future (international perspectives)\Moderators: Stian Almeland, Clemens Schiestl**

Speakers: Tom Potokar, Benjamin Wabwire, José Ramón Martínez Mendez, David Harrington, Thomas Leclerc, Francois Stapelberg, Elena Conde Montero and Bruno Balmelli

Representatives from leading international burn societies will convene to discuss the future of burn care, emphasizing key objectives and challenges. The discussion will encompass technological advancements, the disproportionate burden in low- and middle-income countries, and a paradigm shift from survival to long-term patient reintegration and quality of life. This session aims to promote collaboration and formulate unified strategies to advance burn care, research, and prevention on a global scale.

***Thursday, 4 September 10:30–12:00***

**Plenary session 5: Are we going backwards? The future of treatment in children with severe burns (over 50% TBSA) in Europe**

Moderators: Clemens Schiestl, Mamta Shah

Panelists: Ingo Königs, Djordje Kravlijanac, Rok Kralj, Sophie Böttcher, Sabri Demir, Marcello Zambarelli and Lesia Strilka

The treatment of severe burns in children with more than 50% TBSA has become a problem in many European countries due to the decreasing number of cases. As we all know, education and economic prosperity are the most important factors in reducing these serious accidents. We are very satisfied with this development. On the other hand, the question arises as to the impact of the now low number of cases of children with a burnt TBSA above 50% on the quality of this children. This is the question we want to explore in this session. Starting with the question of whether this assumption is valid for the whole of Europe, examples will be given of how this development can be countered.

***Thursday, 4 September 15:45–17:15***

**Plenary session 6: Continued education of Burn Care Professionals**

Moderators: Luis Cabral, Jill Meirte

Speakers: Mark Fisher, Dominique Potokar, Sigrid Brokke, Naiem Moiemen, Andy Williams and Alette de Jong

This session aims to explore innovative approaches and best practices in the continued education of burn care professionals. Join us for an inspiring session highlighting global efforts in burn care education. Topics include the EBA Staff Exchange Program, the power of social media in professional learning, building a new burn unit in Cairo, global training initiatives by Interburns, the value of the EMSB course, and a nurse-specific program from The Netherlands. With leading international speakers and interactive Q&A, this session showcases innovation, collaboration, and the future of burn education. By sharing insights from various experts, the session seeks to enhance the knowledge, skills, and collaboration among burn care providers globally, ultimately improving patient outcomes.

***Friday, 5 September, 07:30–08:30***

**Coffee with the Experts: Debridement why, when and how**

**Stian K. Almeland and Tom Potokar**

In this session, two experts will explore the vital role of debridement in managing severe burn injuries across settings. They will examine various techniques, including surgical and enzymatic methods, discussing their advantages and disadvantages. The discussion focuses on how, when, and why to debride, emphasizing its role in preventing infection, promoting healing, and wound preparation. The session aims to provide attendees with a thorough understanding of debridement, offering practical insights and encouraging dialogue on burn care best practices.

***Friday, 5 September, 10:30–12:00***

**Plenary Session 7: North Macedonia Burn Mass Casualty: Lessons Learned and Future Preparedness**

**Moderators: Stian K. Almeland and Thomas Leclerc**

Speakers: Igor Peev and Representatives from DG ECHO

EBA Experts will convene with local medical professionals who responded to the initial crisis, along with representatives from European institutions that facilitated the swift activation of the EU Civil Protection Mechanism, to discuss the tragic Kočani nightclub fire in North Macedonia earlier this year. The session will examine lessons learned, ranging from prevention and disaster preparedness to the significance of coordinated international response.

***Friday, 5 September 16:45–18:15***

**Plenary session 8: Skin Engineering**

Moderators: Clemens Schiestl and Jyrki Vuola

Speakers: Clemens Schiestl, Thomas Leclerc, Stian K. Almeland, Rocio G.C. Valencia, Céline Auxenfans and Sophie Böttcher

In the future, autologous skin substitutes could play an important role in further developing modern treatments for patients with severe or massive burn injuries. The European Burn Association’s stated goal is to ensure that patients with severe burns receive modern treatment throughout Europe. To this purpose, the European Skin Engineering Network (ESEN) was established three years ago under the EBA’s umbrella. The first step, supported by the European Union’s COST Action program, is to achieve four important interim goals:

1. Making the regulatory requirements for production laboratories affiliated with a European burn center less complicated.
2. Encouraging collaboration between individual laboratories to exploit existing synergies and promote cooperation.
3. A well-founded, practical training module for interprofessional teams who wish to use or already use autologous skin substitutes produced in a lab.
4. A database of all patients treated with this innovative method.

During the penalty session, we will first address the fundamental question of the extent to which autologous skin substitutes produced in a laboratory could form part of a contemporary treatment strategy, from the perspectives intensive care medicine and burn surgery.

This will be followed by a brief overview of the goals of the program and the European COST Action project and ESEN.

We will round off the topic with a presentation that attempts to describe future developments in this field.

**Saturday, 6 September 07:30–08:00**

**Coffee with the Experts: Hot Seat Challenge: Multidisciplinary Decision-Making in Face and Neck Burn Care**

Mark Fisher and Peter Moortgat

This interactive session places participants at the centre of a high-stakes, multidisciplinary “Hot Seat” challenge. Starting from a virtual case of a patient with severe neck and face burns, the audience will follow the treatment journey across four key specialties: surgery, wound care nursing, physiotherapy/occupational therapy, and psychology. For each discipline, a realistic complication will be introduced. The expert and attendees will propose solutions via live interaction, followed by expert commentary. The audience will then vote for the most effective solution. An AI engine will simulate the projected outcome of each decision using published clinical data, providing immediate feedback on functional, aesthetic, and psychosocial consequences. The process will be repeated for all four disciplines. This session will offer a unique, evidence-informed perspective on complex burn care.

**Saturday, 6 September 08:30–10:00**

***Plenary session 9: Sustainability in burn care***

Moderators: Alette de Jong, Nicole Lee, Tim Verhaak

Speakers: Tim Verhaak, Heather Baid, Nicole Lee, Halldor Hardarson, Shobha Chamania, Helma Hofland, Willemke Stilma and Patrick Deckers

Burn care stands at the crossroads of medical innovation and environmental responsibility. This plenary session dives into the theme of sustainability in burn care, highlighting how the field can evolve to meet the needs of patients today without compromising the health and well-being of future generations. Our expert speakers will share efforts and practical strategies that can inspire burn care professionals across the globe. From green team interventions that spark vital behavioural change in ICUs and burn centres, to airway management practices redesigned for sustainability, the use of fish skin, banana leaves, and papaya, and sustainable food systems, these initiatives show how even small shifts can drive impact.

**Saturday, 6 September 10:30–12:00**

***Plenary Session 10: How we see the future (the youngests perspectives)***

Moderators: Clemens Schiestl, Nadia Depetris

Speakers David Schieffelers, Joana Costa, Ahmed Mokhallalati, Lesia Strilka, Sophia Paraskevopoulou, Sofia Khon and Romana Merza

The Future of Burn Care is Here. Will You Be Part of It? Join us for “The Youngest Perspectives,” a unique plenary session that offers an exclusive look into the future of burn care. We’ll hear directly from a new generation of experts across different specialties from all over the world their motivations, their personal journeys into this challenging field, and the single most impactful change they believe is needed to transform patient care. You’ll discover visionary ideas—from groundbreaking techniques to new collaborative models—that could redefine our work. The session will conclude with a lively, interactive panel discussion. You won’t just be listening; you’ll have the opportunity to engage with these rising stars and contribute to the conversation. Come and be a part of this vital dialogue. Your voice, combined with these fresh perspectives, will help shape the future of burn care.

## 4. Meetings

**M.001 Thursday, 4 September 12:30–13:30**

**Research Committee Meeting: Presentation and Discussion of Research Committee activities**

Paul van Zuijlen (BE), Stian Almeland (NO), Luis Cabral (PT), Nadia Depetris (IT), Alette de Jong (NL), Athina Lavrentieva (GR), Bretislav Lipovy (CZ), Clemens Schiestl (CH), Mamta Shah (UK).

The primary objective of the Research Committee is to design, plan and support multidisciplinary research activities contributing to Burn Care advancements. All the professionals interested in supporting and conducting research in burn care are invited to the meeting on Thursday 4 September, to discover and discuss the present and future activities of the Research Committee, and to join it. If you are interested in joining the Research Committee and contributing to its activities, get in touch with Clemens Schiestl. Write an email introducing yourself and expressing your interest directly to clemens.schiestl@kispi.uzh.ch.

**M.002 Thursday, 4 September 12:30–13:30**

**PAM Committee Meeting: Presentation and Discussion of PAM Committee activities**

Jill Meirte (BE), Lottie Armitage (UK), Gregoire Bondu (FR), Sigrid Brokke (NO), Klaudia Kokkola (FI), Dominique Potokar (FR), Stefania Simone (CH))

The PAM (Professionals Allied to Medicine) Committee covers a wide professional group of non-physicians associated with burn care (Nurses, Physiotherapists, Occupational Therapists, Social Workers, Psychologists, Dieticians, and other professionals). All PAM professionals are invited to the meeting on Thursday 4 September to discover and discuss the past and future activities of the PAM Committee, and to join it, to cooperate in better and more comprehensive European Burn Care.

Moreover, during the PAM Committee meeting, new members will be elected. If you are interested in joining the PAM Committee and contributing to its activities, get in touch with Jill Meirte. Write an email introducing yourself and expressing your interest directly to jill.meirte@uantwerpen.be.

**M.005 Friday, 5 September 12:30 pm–13:30 pm**

**Prevention Committee meeting: Presentation and Discussion of Prevention Committee activities**

Mamta Shah (UK), Karl Bodenschatz (DE), Laetitia Goffinet (FR), Heidi Gottwald (DE), Koen Maertens (BE)

The Prevention Committee aims to prevent burn injuries in Europe and beyond.

All burn care professionals, educationalists, and community workers wishing to promote burns prevention are invited to the meeting on Friday 5 September.

Come to the meeting to discover the present and future activities of the EBA Prevention Committee, and start your cooperation. If you are interested in joining the Prevention Committee and contributing to its activities, get in touch with Mamta Shah. Write an email introducing yourself and expressing your interest directly to mamta.shah@manchester.ac.uk.

## 5. Workshops

***Wednesday, 3 September 15:45–17:15***

**Workshop Beyond the Manuscript: Open Insights into Academic Publishing**

***Isabel Nelson (UK), Anya Osborn (UK), Naiem Moiemen (UK), Peter Vogt (Germany)***

This workshop, led by MDPI in collaboration with the European Burn Journal (EBJ), will cover a range of topics from Open Access Publishing, Editorial Process and Peer Review, offering an open and transparent insight into the academic publishing world. The session will also introduce the EBA’s own journal, its Editors-in-Chief, and highlight its achievements so far, providing valuable insights for early-career and established researchers alike.

***Thursday, September 4th 08:30–10:00***

**Workshop Camouflage & Body Image Intimacy/Sexuality**

This workshop will address the critical and often overlooked aspects of body image and intimacy in the rehabilitation of burn survivors. The aim is to enhance the knowledge and practical skills of healthcare professionals in supporting the psychological and intimate needs of burn survivors. Through open dialogue and the introduction of supportive tools, resources and camouflage technique, we seek to foster more confident and holistic care. We look forward to your participation in this important and impactful session

We will have 2 stations in this workshop.

Station 1: Camouflage station by Kryolan Professional Make Up.

This session helps professionals understand camouflage as one valuable technique that can assist burn survivors in managing changes to their body image.

Station 2: Sexuality & Intimacy Station by Professionals Allied to Medicine (PAM) committee members, 2 experts in the field, and burn survivors.

By encouraging open dialogue, we aim to normalize sexuality and intimacy as essential components of holistic burn care, gather further input to build on the initial work; practical recommendations and supportive tools in this important area, and refine and optimize the two supportive tools for both healthcare professionals and patients.

***Thursday, 4 September 13:45–15:15***

**Workshop Rehabilitation Relay: Building the Strongest Burn Recovery Plans Together**

Join us for an engaging, team-based challenge where participants race to create optimal rehabilitation strategies for real-life burn cases. This interactive session emphasizes holistic, personalized care through collaboration across disciplines. Work together to address complex physical, psychological, and social needs—because the strongest recovery plans are built as a team. Ready, set, rehabilitate! Created by the Professions Allied to Medicine (PAM) Committee team Lottie Armitage, Stefania Simone, Gregoire Bondu, Sigrid Brokke, Klaudia Kokkola, Dominique Potokar, Jill Meirte

***Friday, 5 September* 8:30–10:00**

**Workshop 4: Epidemiological data collection in burn care**

Kenn Dunn, Ludwig Bransky, Lindsay Damkat-Thomas, Oliver Thamm, Emily Bebbington

This workshop focuses on the crucial role of prospective data collection in burn care. Despite significant advancements in burn treatment, a lack of standardized data collection hinders a comprehensive understanding of burn injury trends, outcomes, and resource allocation. Experts will present burn registies from different developed countries and discuss current challenges and propose solutions for harmonizing data collection methods. Key topics include the importance of a minimum data set, the integration of electronic health records, and the ethical considerations of data sharing. A roadmap for a collaborative, worldwide burn registry would enable more effective prevention strategies, optimize treatment protocols, and ultimately improve patient outcomes. Participants are invited to actively join the discussion.

***Friday, 5 September 13:45–15:15***

**Ethical Challenges in the Digital Age- A Workshop for Burn Care Professionals**

Session faculty: Laura Pompermaier (chair), Sigrid Brokke, Shah Mamta, Romana Merz, Mark Fisher, David Schieffelers, Paul Van Zuijlen

This interactive workshop addresses the ethical complexities of digital sharing in burn care. Through live polling, case-based group discussions, and real-world examples, participants explore the Four Principles of Biomedical Ethics—Autonomy, Beneficence, Non-maleficence, and Justice. Activities include peer reflection, ethical analysis of clinical images, and practical tools like a checklist for responsible sharing. Designed for clinicians, the session offers insights and strategies to ethically navigate online case sharing in clinical and academic settings.

## 6. Oral Presentations

*O01.1 Investigating the impact of pediatric burn injury on the brain using MRI*

**Allahham A** ^1,2^, Fear M ^1,2^, Martin L ^1,2,3^, Vos S ^2,4^, Shoesmith V ^1,3^, Wood F ^1,2,3,5^

^1^ Fiona Wood Foundation, Perth, Australia, ^2^ University of Western Australia, Perth, Australia, ^3^ Perth Children’s Hospital, Perth, Australia, ^4^ National Imaging Facility, Perth, Australia, ^5^ Burn Service of Western Australia, Perth, Australia

Oral presentations 01—Basic research 1, 3 September 2025, 15:45–17:15

Background:

Children with burn injuries have a higher risk of being admitted to hospital for mental health conditions. Previous animal model studies have shown significant transcriptomic and metabolomic changes in the central nervous system (CNS) 3 months after a non-severe burn, suggesting a physiological impact of burn trauma on the brain that persists after recovery.

Aim:

This study aims to investigate the potential physiological impact of non-severe burn injuries on the brain in a paediatric burn population compared to uninjured controls using Magnetic Resonance Imaging (MRI).

Method:

The burn group includes paediatric burn patients with a burn injury ≥1% of their total body surface area (TBSA) more than 5 months postburn. The MRI scans were done in a 40-min session that included the following scans: 3D-T1-weighted scan, 3D-T2-FLAIR, resting-state functional scan, and task-based functional scan. Together, these scans measured structure, function, and metabolism in the brain.

Results:

Preliminary results suggest changes in the volume of the certain regions within the parietal lobe including a 1245 mm^2^ cluster in the superior parietal lobe as well as changes in network connectivity between the frontal lobe and the somatosensory cortex.

Conclusions:

The data in this study supported the hypothesis that there are changes to the CNS after non-severe burns. Volumetric and network data suggests both functional and structural changes were present in the brain after burns. This study will help us better understand the physiological impact of burn trauma on the CNS, providing opportunity to explore links between physiological changes and mental health postburn.

*O01.2 Engineering Fibroblast Growth Factor (FGF) Stability: The Next Frontier in Skin Regeneration*

**Lipovy B** ^1,2,3^, Pavlinakova V ^3^, Knoz M ^4^, Holoubek J ^2^, Horalkova E ^2^, Smolnicka A ^5^, Faldyna M ^6^, Jeklova E ^6^, Herudek J ^7^, Koutna I ^5,8^, Vojtova L ^3^

^1^ Department of Burns Medicine, Third Faculty of Medicine, Charles University and University Hospital Kralovske Vinohrady, Prague, Czech Republic, ^2^ Department of Burns and Plastic Surgery, Institution Shared with University Hospital Brno, Faculty of Medicine, Masaryk University, Brno, Czech Republic, ^3^ CEITEC—Central European Institute of Technology, Brno University of Technology, Brno, Czech Republic, ^4^ Clinic of Plastic and Esthetic Surgery, St Anne’s University Hospital, Brno, Czech Republic, ^5^ Department of Histology and Embryology, Faculty of Medicine, Masaryk University, Brno, Czech Republic, ^6^ Veterinary Research Institute, Brno, Czech Republic, ^7^ Enantis Ltd., Brno, Czech Republic, ^8^ Cell and Tissue Engineering Facility, International Clinical Research Center, Brno, Czech Republic

Oral presentations 01—Basic research 1, 3 September 2025, 15:45–17:15

Aim:

Fibroblast Growth Factors (FGFs), particularly FGF2, FGF7, play crucial roles in skin regeneration by promoting proliferation and angiogenesis. Our engineered stabilized variants overcome the historical instability of these factors, enabling transformative clinical applications in wound healing and tissue engineering.

Methods:

Initial research developed second-generation biomaterials for dermal substitutes using a porous collagen-chitosan foam with stabilized FGF2 (FGF2-STAB^®^, Core Biogenesis, Strasbourg, France) (0.1–10 µg/cm^2^). Neovascularization was confirmed via CAM and porcine models through histological analysis. Current work advances to fourth-generation biomaterials combining FGF2-STAB^®^, FGF7-STAB^®^, and differentiated MSCs, now undergoing functional assessment in porcine wound models to evaluate regenerative potential.

Results:

Our first-phase investigation successfully validated the biocompatibility profile of resorbable dermal substitute. Six-month follow-up data from swine models confirmed sustained tissue integration, safety, and significantly enhanced neovascularization. Optimal vascular parameters (vessel density and area) were achieved at 0.1 and 1 µg·cm^−2^ concentrations. Based on these findings, we developed fourth-generation constructs with MSCs and stabilized growth factors. Early in vivo results demonstrate excellent biocompatibility, faster wound healing, and superior regeneration. Current testing combines advanced in vitro models with animal studies to assess long-term outcomes.

Conclusions:

The therapeutic potential of FGFs has been historically limited by their transient activity. Our breakthrough in protein stabilization overcomes this critical barrier, creating new opportunities for clinical translation. We propose a synergistic platform merging tissue engineering, stabilized FGF delivery, and cellular therapy to transform skin regeneration paradigms.

Acknowledgments:

This study was supported by the Ministry of Health of the Czech Republic, grant No. NU22-08-00454. All rights reserved.

*O01.3 Bibliometric Analysis and Initial Animal Efficacy Evaluation of Top Ten Scoring Drugs to Enhance Oral Rehydration Therapy in Early Post-Burn Shock*

**Liu X** ^1^, Wu Y ^1^, Chai J ^1^, Qu Y ^1^, Zhou H ^1^, Liu T ^1^, Chi Y ^1^

^1^ Fourth Medical Center of Chinese PLA General Hospital, Beijing, China

Oral presentations 01—Basic research 1, 3 September 2025, 15:45–17:15

Aim:

Burns can cause severe physiological disturbances. Oral rehydration therapy (ORT) is an alternative to intravenous fluids. However, the World Health Organization-recommended oral rehydration solution (ORS) lacks specific components to address the critical physiological changes in burns. This study aimed to identify and evaluate several drugs that enhance the ORT efficacy in burn shock management.

Methods:

A systematic search of PubMed, Web of Science, and Scopus (1 January 2000–30 June 2024) yielded 1500 relevant studies, from which 270 were selected for bibliometric analysis. Drug candidates were prioritized based on publication frequency (≥3 mentions), journal impact factor (5-year average), impact score, and Q1 journal distribution. The top 10 drugs were tested in a rat model with 50% total body surface area full-thickness burns (*n* = 286, 22/group), comparing sham controls, un-treated controls, WHO-ORS, and drug-adjuvanted ORS groups. Primary outcomes included 48 h survival rate and blood lactate (Lac), hematocrit (HCT), malondialdehyde (MDA), and interleukin-6 (IL-6) levels.

Results:

Teprenone or vitamin C in com-bination with the WHO-ORS significantly improved survival outcomes following severe burns. They reduced blood lactate, HCT, MDA, and IL-6 levels. Glutamine and ethyl pyruvate showed beneficial effects but did not significantly improve survival. Hypertonic Saline and Dobutamine failed to demonstrate efficacy.

Conclusions:

This study demonstrated that adding teprenone or vitamin C to the WHO-recommended ORS can enhance the therapeutic efficacy of ORT in managing burn shock. These findings provide a scientific basis for further clinical trials and development of optimized ORS for patients with burns.

*O01.5 From rodents to humans—Translational aspects of burn wound models*

**Pignet A** ^1^, Hofmann E, Fink J, Prevedel M, Kamolz L, Kotzbeck P

^1^ Medical University Graz, Graz, Austria

Oral presentations 01—Basic research 1, 3 September 2025, 15:45–17:15

Aim:

Here, we aimed to establish time-resolved burn models of partial to full-thickness burns to analyze the exact sequence of local tissue reactions focusing on the inflammatory response up to 21 days post injury.

Material and Methods:

We induced contact burns on fresh human abdominal skin explants, as well as on pigs and rats. Additionally, we included a translational approach by analyzing tissue samples of patients with burns. Gene expression patterns of mediators for inflammation and angiogenesis were investigated. Additionally, we collected dermal interstitial fluid to monitor the release of eicosanoids, cytokines and metabolites.

Results:

Inflammatory markers such as IL-6, 8 or 10 were significantly up-regulated upon burn injury across all studied model organisms, including the tissue explants. Tissue explants were proven to be viable for at least 24 h after explantation. Angiogenic markers, such as HIF1A and VEGFA, on the other side, were downregulated after burn injury in rats and pigs. In human samples –ex vivo, as well as in patients- these markers were significantly up-regulated. Metabolome analysis of dISF showed a striking metabolic shift in burn injuries, linking burn injury to increased amino-acid and glucose turn over.

Conclusions:

While the inflammatory response seems to be regulated similarly across rats, pigs, ex vivo flaps and patients, markers for angiogenesis seem to be regulated differently between animal models and humans. The knowledge gained from this study might aid in understanding the complex biological dynamics in the healing process of burn wounds and might facilitate choosing an adequate model organism.

*O01.6 A novel mice model of burn with smoke inhalation injury*

**Qu Y** ^1^, Hu F ^1^, Zhou H ^1^, Liu T ^1^, Chai J ^1^, Liu X ^1^, Chi Y ^1^

^1^ Chinese People’s Liberation Army (pla) General Hospital, Beijing, China

Oral presentations 01—Basic research 1, 3 September 2025, 15:45–17:15

Aim:

To establish a stable mouse model of burns combined with smoke inhalation injury for clinical research on related diseases.

Methods:

A total of 230 female C57BL/6 mice (6–8 weeks old) were randomly assigned to four groups: Sham (*n* = 30), Smoke (*n* = 60), Burn (*n* = 60), and Burn + Smoke (S + B, *n* = 80). Mice were shaved to expose the skin. The Smoke group underwent 20-min smoke inhalation, the Burn group was immersed in 90 °C water for 9 s to induce 30% third-degree burns, and the S + B group received both smoke inhalation and burns. Fluid resuscitation was provided according to the Parkland formula. Survival was monitored over 72 h, and various clinical and pathological parameters, including blood gas analysis, platelet counts, lung function, and inflammatory cytokines (IL-1β, IL-6, IL-10, TNF-α), were measured.

Results:

The survival rate of the S + B group was significantly lower than that of the Smoke and Burn groups (*p* < 0.05). Blood gas and platelet counts worsened significantly in all injury groups, with the S + B group showing the most severe deterioration (*p* < 0.01). Lung function, including VT, MV, and F, declined significantly in the injury groups, with the S + B group showing the most pronounced decline. Inflammatory markers (IL-1β, IL-6, IL-10, TNF-α) were significantly higher in the injury groups, especially in the S + B group (*p* < 0.01).

Conclusions:

The combination of 30% third-degree burns and 20-min smoke inhalation creates a moderate to severe injury model in mice, mimicking clinical pathology, and is suitable for further research.

*O01.7 Lactate-Mediated Healing in Burns: Mechanistic Insights into the Bioactivity of PLA-Based Membranes*

**Ramirez-Garcialuna J** ^1,2^, Martinez-Jimenez M ^2^

^1^ Mcgill University, Montreal, Canada, ^2^ Universidad Autonoma de San Luis Potosi, San Luis Potosi, Mexico

Oral presentations 01—Basic research 1, 3 September 2025, 15:45–17:15

Aim:

To explore the mechanistic basis for the observed clinical efficacy of polylactic acid (PLA)-based membranes in burn wound healing, with emphasis on the role of lactate as a bioactive molecule.

Methods:

This mechanistic review synthesizes experimental, translational, and clinical evidence regarding the effects of lactate released from PLA-based epidermal membranes in burn injury patients. Peer-reviewed data was compiled from cellular, animal, and human studies addressing vascular, immune, and neurosensory responses to lactate signalling in the wound environment.

Results:

Lactate released from PLA-based membranes initiates a multifaceted cascade of bioactive effects essential for burn repair. First, lactate mimics hypoxic signalling and activates hypoxia-inducible factor-1α (HIF-1α), promoting neoangiogenesis via VEGF expression. Second, fibroblast survival, keratinocyte proliferation, and extracellular matrix deposition are enhanced. Third, lactate modulates inflammation by shifting macrophage polarization from pro-inflammatory (M1) to reparative (M2) phenotypes. Fourth, it lowers wound pH, creating an acidic microenvironment that impairs bacterial growth and favours enzymatic activity involved in healing. Lastly, lactate reduces nociceptor sensitization through TRPV1 channel desensitization, contributing to adequate pain control. Clinically, these mechanisms correlate with faster healing, lower infection rates, reduced need for grafting, and improved patient comfort.

Conclusions: 

PLA-based membranes act not only as physical wound coverings but also as bio-inductive platforms that release lactate, driving a cascade of regenerative effects. Their multifactorial action makes them uniquely suited for managing partial-thickness burns, with implications for broader use in complex wound care. Further clinical trials should continue to elucidate the optimal indications and comparative advantages of lactate-releasing dressings.

*O01.8 LncRNA-NEAT1 regulates the permeability of microvascular endothelial cells through the miR-664b-3p/MMP13 axis in sepsis*

Liu T ^1^, **Zhou H** ^2^, Chi Y ^1^

^1^ The Fourth Medical Centre of Chinese PLA General Hospital, Beijing, China, ^2^ Chinese PLA Medical School, Beijing, China

Oral presentations 01—Basic research 1, 3 September 2025, 15:45–17:15

Aim:

We investigated the effect of elevated lncRNA-NEAT1 on the highly increasing permeability of microvascular endothelium during sepsis and the underlying mechanism while exploring a new therapeutic target for sepsis.

Methods:

Human dermal microvascular endothelial cells (HDMECs) were transfected with siRNA or pcDNA3.1 plasmid to knockdown or overexpress the lncRNA-NEAT1 before being treated with 10 μg/mL LPS for 12 h to mimic sepsis. The proliferation and apoptosis of HDMECs were measured using the CCK-8 assay and flow cytometry. The expression of tight junction protein ZO-1 and cytoskeletal protein F-actin was analyzed by qPCR, Western blot, and immunofluorescence. The permeability of the confluence HDMECs liner was quantitatively characterized by measuring transendothelial resistance and FITC-dextran permeability. Further pathways were explored through bioinformatics analysis and databases including StarBase v2.0, miRWalk, ENCORI, and TargetScan. We also produced corresponding mimics, inhibitors, siRNA, and pcDNA3.1 plasmid for validation.

Results:

Compared with controls, si-lncRNA-NEAT1 treatment increased the viability (*p* < 0.05), expression of ZO-1 and F-actin, and transendothelial resistance (*p* < 0.01) of HDMECs and decreased FITC-dextran permeability (*p* < 0.01) after LPS treatment. The binding between miR-664b-3p and lncRNA-NEAT1 was predicted, and MMP-13 was identified as a target gene of miR-664b-3p. Luciferase validated the lncRNA-NEAT1/miR-664b-3p/MMP13 axis. Transfection of si-MMP-13 also improved cell permeability and protein expression.

Conclusions: 

In the LPS-induced sepsis model, elevated lncRNA-NEAT1 bound to miR-664b-3p, resulting in increasing MMP-13 expression, which damaged the permeability of microvascular endothelial cells. Knockdown of either lncRNA-NEAT1 or MMP-13 improved the protection of vascular permeability.

*O01.9 A diagnostic model for sepsis based on disulfidptosis-related genes: a bioinformatics analysis*

Liu T ^1^, **Zhou H** ^2^, Chi Y ^1^

^1^ The Fourth Medical Centre of Chinese PLA General Hospital, Beijing, China, ^2^ Chinese PLA Medical School, Beijing, China

Oral presentations 01—Basic research 1, 3 September 2025, 15:45–17:15

Aim:

We investigated the role of disulfidptosis-related genes (DRGs) in sepsis and constructed a sepsis diagnostic model, providing a new research direction for the diagnosis and treatment of sepsis.

Methods:

The GSE65682 dataset from the GEO database was analyzed to screen for DRGs. Further analyses, including immune cell infiltration, the correlation between DRGs and infiltrating immune cells, unsupervised clustering, principal component analysis, and weighted gene co-expression network analysis were performed. Four different machine learning models—the random forest model, vector machine model, generalized linear model, and extreme gradient boosting model—were constructed and analyzed with ROC curves. The diagnostic efficacy of four diagnostic models was verified by the GSE185263 dataset.

Results:

21 genes associated with disulfidptosis in sepsis were obtained. There was a correlation between 7 differentially expressed genes and immune cell infiltration. In our study, sepsis patients were divided into two subgroups, C1 and C2, according to the differentially expressed genes. We found that the vector machine model was the best classification model associated with disulfidptosis in sepsis (AUC = 0.989), and the 5 DRGs for diagnosing sepsis obtained from this model were FSTL1, SELP, PPBP, TGA2B, and PF4 (AUC = 0.871 in the validation set).

Conclusions: 

We confirmed the involvement of disulfidptosis in the psychophysiology process of sepsis, and DRGs are associated with immune cell infiltration in sepsis. A disulfidptosis-based sepsis diagnostic model was established, and five key genes were obtained by vector machine model, which showed good performance in both training and validation sets.

*O02.1 Enhancing burn care with active dressings: a comparative analysis of healing outcomes across second-degree burns*

**Alexandru A** ^1^, Mihaela P ^1,2^, Dan-Cristian M ^1,2^, Marian B ^1^, Bianca A ^1^, Irina-Andra B ^1,2^

^1^ County Emergency Hospital “St. Spiridon” Iasi, Romania, Iasi, Romania, ^2^ University of Medicine and Pharmacy “Grigore T. Popa”, Iasi, Romania

Oral presentations 02—Wounds 1, 3 September 2025, 15:45–17:15

Aim:

To evaluate the impact of active and biological dressings on wound healing, infection control, and functional recovery in second-degree burns.

Methods:

A retrospective cohort study analyzed 79 patients with second-degree burns, treated with active dressings (hydrocolloid, hydrogel, silver-impregnated) and a control group treated with conventional dressings. The active dressings were either used individually or combined in a two-layer approach (hydrocolloid and hydrogel) to optimize wound moisture balance and facilitate faster healing. Burn depth was assessed using Laser Doppler imaging. Dressings were changed every 2 days. Primary outcomes included healing time, infection rates, inflammatory response, pain scores, long-term functional and aesthetic outcomes, and the need for surgical intervention. A Bayesian network meta-analysis compared dressing effectiveness.

Results:

Active dressings significantly accelerated epithelialization, with a median healing time of 7–10 days. Combining hydrocolloid and hydrogel dressings enhanced autolytic debridement, while silver-impregnated dressings contributed to a reduction in infection rates. The combination of dressings regulated exudate, promoted a moist wound environment, and minimized fibrin deposition. Hypertrophic scarring was reduced, and dermal regeneration was improved. Secondary contracture rates were lower in the active dressing group, leading to enhanced functional outcomes, especially in areas prone to movement.

Conclusions: 

Combining active dressings optimizes wound healing in second-degree burns, reducing scarring, preventing contractures, and improving long-term functional and aesthetic outcomes. Standardized protocols incorporating active dressings could improve burn care and reduce the need for surgical interventions.

*O02.2 Assessment of the pH alteration in acute burn wounds treated with Polylactide Membrane versus those treated with silver based dressing*

**Demircan M** ^1^

^1^ İnönü University School of Medicine, Malatya, Turkey

Oral presentations 02—Wounds 1, 3 September 2025, 15:45–17:15

Aim:

We hereby present our study assessing pH changes in pediatric acute burn wounds treated by polylacticde membranes (PLM) versus silver dressings during first 14 days.

Method:

Twenty acute pediatric burn wounds with scalding and 10–20% TBSA were included in this study. The pH assessment was done using litmus paper testing on days 0,3, 7 and 14. All the wounds were dressed in a similar sequential manner according to their treatment group PLM or silver dressing). On day 14, the re-epithelialization rate was assessed in both groups.

Results:

The pH value of the fresh acute burn wounds was between 8 and 9 on day 0. On days 3 and 7, the pH value shifted to an average of 7.5 in the PLM group and an average of 5.5 in the silver group. On day 14, the pH of the PLM group was at an average of 7 whereas the silver group had an average pH value of 6.

Conclusions:

Our study shows that the application of PLM shifted the pH value of the wound to an ideal value for healing. Whereas the silver dressing shifted the pH values to a slightly lower range making it less optimal for wound healing. This was also proven by the re-epithelialization rate which was lower for the wounds treated by a silver dressing. The release of lactate from the polylactic membrane may have caused the alkaline pH to shift towards the ideal pH value between 7 and 8 for optimal acute burn wound healing.

*O02.4 Acetic acid treatment of MDR bacteria infected burn wounds*

Rantalahti R ^1^, **Ahola J** ^1^

^1^ Helsinki University Central Hospital, Helsinki, Finland

Oral presentations 02—Wounds 1, 3 September 2025, 15:45–17:15

Aim:

Aim is to present how burn wound treatment had to be re-evaluated when treating patients with multi drug resistant (MDR) bacterial wound infections.

Methods:

2022 Helsinki Burn Centre started treating Ukrainian patients. All the patients treated, had MDR infected wounds upon arrival. Wound care protocol was re-evaluated, since the standard one used, proved not to be effective enough with these wounds. There was graft loss and donor site healing problems before the change in protocol. Up to date Helsinki Burn Centre has treated 6 severely injured burn patients from Ukraine.

Results:

In the new protocol patients are treated with 2,5% acetic acid dressings 2–3 times a day. Polyhexanide/betaine solution is used with mechanical wound cleaning and acetic acid gauze is used with wet-to dry method. As wounds begin to heal, the number of dressing changes per day is reduced. Acetic acid is changed to a less traumatic wound care product as soon as possible. Strict isolation protocol is carried out by all burn unit personnel. So far none of the patients has had a systemic septic episode during hospital stay and there hasn’t been any spread of MDR infection in Helsinki Burn Centre.

Conclusions:

All patients with MDR infected wounds treated with wet-to-dry method using 2.5% acetic acid healed well. This method needs special attention to pain management and wound care environment, as it is painful and might lower the wound surface temperature unnecessarily.

*O02.5 Wound and scar outcomes of Meek micrografting versus Mesh grafting: an intra-patient randomized controlled trial*

**Rijpma D** ^1,2,3^, Claes K ^4,5^, Pijpe A ^1,2,3^, Hoeksema H ^4,5^, de Decker I ^4,5^, Verbelen J ^5^, Stoop M ^1,2^, de Mey K ^5^, Hoste F ^6^, van Zuijlen P ^1,2,7,8^, Monstrey S ^4,5^, Meij-de Vries A ^1,8,9^

^1^ Burn center, Red Cross Hospital, Amsterdam, The Netherlands, ^2^ Amsterdam UMC location Vrije Universiteit Amsterdam, Plastic, Reconstructive and Hand Surgery, Amsterdam, The Netherlands, ^3^ Amsterdam Movement Sciences, Tissue Function and Regeneration, Amsterdam, The Netherlands, ^4^ Department of Plastic Surgery, Ghent University Hospital, Ghent, Belgium, ^5^ Burn Center, Ghent University Hospital, Ghent, Belgium, ^6^ Faculty of medicine and health sciences, Ghent, Belgium, ^7^ Department of Plastic, Reconstructive and Hand Surgery, Red Cross Hospital, Beverwijk, The Netherlands, ^8^ Department of Pediatric Surgery, Amsterdam UMC location AMC, Amsterdam, The Netherlands, ^9^ Department of Surgery, Red Cross Hospital, Beverwijk, The Netherlands

Oral presentations 02—Wounds 1, 3 September 2025, 15:45–17:15

Aim:

Mesh grafting and Meek micrografting are split thickness skin graft expansion techniques. This study aimed to compare the results of Mesh and Meek expansion ratios 1:2 and 1:3 in smaller wounds.

Methods:

An intra-patient randomized, controlled trial was conducted in two burn centers (The Netherlands and Belgium). Wound outcomes e.g., donor site size, take rate, re-epithelialization rate were measured. Moreover, patient preference was measured at hospital discharge and at 3 months post-surgery. Scar quality was evaluated with the Patient and Observer Scar Assessment Scale (POSAS), Cutometer and Dermaspectrometer at 3 months post-surgery.

Results:

70 patients with an affected total body surface area of 10 ± 10% (mean ± SD) were included. Donor site size was significantly larger for Mesh compared to Meek. Take rate was 87 ± 19% vs. 79 ± 25% (*p* = 0.003), Mesh vs. Meek respectively. At follow-up, majority of observer and patient POSAS items were statistical significantly lower, corresponding with a better scar quality, for Mesh grafting compared to Meek micrografting. Scar elasticity was 0.42 ± 0.21 vs. 0.37 ± 0.20 (*p* = 0.013), mean melanin was 12.1 ± 7.7 vs. 13.3 ± 8.3 (*p* = 0.019), for Mesh vs. Meek respectively and patient preference was 49%, 32%, 19% for Mesh, Meek, and no preference. Other outcomes showed no statistically significant difference.

Conclusions:

In patients with smaller wounds, Mesh showed superiority on most wound and short-term scar results. Overall, patients preferred Mesh grafting, however the preference of patients within the 1:3 expansion ratio group and donor site size were in favor of Meek.

*O02.6 How the world manages acute burns: insights from an international survey of 106 burn units across 50 countries*

**Salemans R** ^1,2^, Simon M ^3^, van Baar M ^1,4^, Baran K ^5^, Oosterbos S ^6^, Wood F ^7,8^, Edgar D ^7,8,9,10,11^, van Uden D ^1^, van der Vlies C ^1,2,12^

^1^ Alliance of Dutch Burn Care (ADBC), Burn Centre, Maasstad Hospital, Rotterdam, The Netherlands, ^2^ Trauma Research Unit Department of Surgery, Erasmus MC, University Medical Centre Rotterdam, Rotterdam, The Netherlands, ^3^ Amsterdam UMC location VUmc, Department of Plastic, Reconstructive and Hand Surgery, Amsterdam, The Netherlands, ^4^ Erasmus MC, University Medical Centre Rotterdam, Department of Public Health, Rotterdam, The Netherlands, ^5^ Alliance of Dutch Burn Care (ADBC), Burn Centre, Red Cross Hospital, Beverwijk, The Netherlands, ^6^ Alliance of Dutch Burn Care (ADBC), Burn Centre, Martini Hospital, Groningen, The Netherlands, ^7^ Fiona Wood Foundation, Fiona Stanley Hospital, Murdoch, Western Australia, Australia, ^8^ State Adult Burn Unit, Fiona Stanley Hospital, South Metropolitan Health Service, Murdoch, Western Australia, Australia, ^9^ Institute for Health Research, Burn Injury Research Node, The University of Notre Dame Australia, Fremantle, Western Australia, Australia, ^10^ Burn Injury Research Unit, Faculty of Medicine and Dentistry, University of Western Australia, Crawley, Western Australia, Australia, ^11^ Safety and Quality Unit, Armadale Kalamunda Group Health Service, East Metropolitan Health Service, Mt Nasura, Western Australia, Australia, ^12^ Department of Trauma and Burn Surgery, Maasstad Hospital, Rotterdam, The Netherlands

Oral presentations 02—Wounds 1, 3 September 2025, 15:45–17:15

Aim:

This study aimed to explore global burn care practices through an international survey, providing insight into diagnostic, debridement, and coverage techniques and their timing.

Methods:

An online survey was developed and distributed to burn surgeons worldwide, focusing on the management of four patient cases and standard practices. The four cases represented patients with different burns of varying depth and extent. The survey was developed and tested in collaboration with burn care experts, and distributed via email. One respondent per burn centre was permitted to prevent duplication. Data were analysed using descriptive statistics, Fisher’s exact tests and Chi-square tests, with responses categorised by region and economic status.

Results:

Burn surgeons from 106 burn units across 50 countries completed the survey, achieving a 44% response rate. Early surgical debridement within 48 h was considered standard care, though the definition of early grafting varied. Low- and middle-income countries (LMIC) and high-income countries (HIC) differed in the availability of diagnostic tools, enzymatic debridement, and wound coverage techniques. Further regional differences were observed among HICs in Europe, North America, and Oceania, highlighting diverse approaches to burn care worldwide.

Conclusions: 

This study provides a global overview of acute burn management, revealing both universally common practices and significant regional variations between continents, as well as between LMICs and HICs. The lack of consensus on surgical timing and the absence of preferred techniques emphasise the need for treatment tailored to institutional resources and individual surgeon and patient preferences.

*O02.7 Artificial Intelligence-Enhanced Multispectral Imaging for Burn Wound Assessment: Insights from a Multi-Centre UK Trial*

**Tan L** ^1^, Sheikh Z, Lewis C

^1^ Royal Victoria Infirmary, Newcastle, United Kingdom

Oral presentations 02—Wounds 1, 3 September 2025, 15:45–17:15

Aim:

This study examines the application of AI-enhanced MSI for burn wound assessment in a Multi-centre UK setting.

Method

We conducted a Multicentre prospective cohort study at the Newcastle and Manchester burn centre, including patients over 18 years old with burns that did not undergo surgery. The primary outcome was the reliability and reproducibility of healing prediction, whilst the secondary outcome was the instrument’s feasibility. The AI’s prediction was compared to the clinical healing assessment by 21 days as the reference standard. Image J was used to analyse the images, and the statistical analyses were performed using R (version 4.4.1)

Results:

The study included 40 patients and 67 burn images, generating approximately 13 million data points. The mean age of the patients was 51, with an average Total Body Surface Area (TBSA) of 4.06%. The AI-enhanced multispectral imaging system demonstrated a sensitivity of 80.7% (95% CI: 51.8–100%) and a specificity of 95.5% (95% CI: 93.3–97.8%). The overall accuracy of the system was 95.3% (95% CI: 93.2–97.6%). The mean time from scan to result was 5 min, indicating the system’s high efficiency. The device was portable and utilized in clinics, operating theatres, and emergency departments.

Conclusions:

Our study demonstrates that the AI-enhanced multispectral imaging (MSI) system offers high accuracy compared to clinical healing outcomes as the ground truth. Its combined attributes of diagnostic precision, operational efficiency, and portability position this device as a transformative tool for revolutionizing current clinical practices in burn wound assessment.

*O02.8 Does the Dressing Matter in Pediatric Partial-Thickness Burns: A Systematic Review and Meta-Analysis*

**van de Warenburg M** ^1,2^, Teeuwen B ^1,2^, Hummelink S ^2^, Ulrich D ^1,2^, Vehmeijer-Heeman M ^1,2^

^1^ Amalia Centre of Expertise Pediatric Trauma and Burns, Radboud University Medical Centre, Nijmegen, The Netherlands, Nijmegen, The Netherlands, ^2^ Department of Plastic, Reconstructive and Hand Surgery, Radboud University Medical Centre, Nijmegen, The Netherlands, Nijmegen, The Netherlands.

Oral presentations 02—Wounds 1, 3 September 2025, 15:45–17:15

Superficial partial thickness burns typically receive a nonoperative treatment, whereas deep partial thickness burns, which are prone to hypertrophic scarring, are usually managed through debridement followed by autologous split-thickness skin grafting. Various therapies have been developed to prevent wound infection and to enhance wound healing in pediatric partial-thickness burns. However, the choice of dressing by the surgeon can be influenced by various factors. It is worth noting that there is no standardized approach across all burn centers, leading to variations in care practices. To optimize pediatric patient care, a systematic review was conducted following PRISMA guidelines to review existing treatment options for partial thickness burns in children. Outcomes of interest were wound healing time, dressing changes, length of hospital stay, wound infections, need for grafting despite treatment, and hypertrophic scarring. A total of 68 studies with 8199 patients were included. The mean age of the included patients was 3.1 years, and the mean total body surface area of the burns was 15.6%. Treatment groups included topical agents, bandages, skin analogues, or unclassified. Considering all treatment outcomes evaluated in this systematic review of literature, non-silver bandages and skin analogues may have some benefit over topical agents in terms of wound healing time, length of hospital stay, hypertrophic scarring, pain management, and cost saving. Dressing changes, wound infections, and need for grafting, did not significantly change between various treatments.

*O02.9 Challenges and Trends in the Management of Genital Burns: A Retrospective Study from a Major Burn Center in Germany*

**Enechukwu A** ^1^, Lorbeer L ^1^, Thielen V ^1^, Navarro L ^1^, Suhr L ^1^, Gulbis N ^1^, Obed D ^1^, Vogt P ^1^

^1^ Hannover Medical School, Hannover, Germany

Oral presentations 02—Wounds 1, 3 September 2025, 15:45–17:15

Aim:

Genital burn injuries represent a distinct clinical challenge due to the anatomical sensitivity of the region, high risk of infection, and potential long-term functional and psychological consequences. This study aimed to evaluate the etiology, clinical characteristics, and treatment approaches for genital burns, with the goal of developing a structured and reproducible management algorithm.

Methods:

A retrospective, single-center analysis was conducted over a four-year period at a major burn center in Germany. All patients presenting with genital burns were included. Collected data included demographics, burn depth and extent, etiology, treatment modality (conservative or surgical), duration of hospitalization, and complications. Treatment patterns and outcomes were evaluated to support the development of a practical algorithm.

Results:

The patient cohort showed variation in age, gender, and mechanisms of injury, with scalds being the most frequent etiology. Management included both conservative wound care and surgical interventions, such as debridement and skin grafting. The duration of inpatient treatment was influenced by burn severity and complications, including infections and delayed healing. Based on the findings, a stepwise treatment algorithm was formulated.

Conclusions:

Optimal management of genital burns requires a multidisciplinary approach involving burn surgeons, urologists, gynecologists, psychologists, and intensivists. Early recognition of surgical indications is critical to improving outcomes. Although a standardized algorithm can assist in guiding treatment, individualized patient care remains essential due to the heterogeneity of injuries.

*O2.10 The tumescent technique in local burn treatment: advantages and applications*

**Stevkovska M** ^1^

^1^ Biljana Todorovska Shapova, Skopje, RN Macedonia

Oral presentations 02—Wounds 1, 3 September 2025, 15:45—17:15

Aim:

This article presents our experience with the use of the tumescent technique in patients treated at our hospital during the burn disaster in Kochani on 16 March 2025. The objective was to evaluate the advantages and applications of the tumescent technique in patients with localized burns requiring surgical intervention.

Methods:

The study included patients with burns involving less than 15% of the total body surface area (TBSA), with 2–5% deep dermal burns, along with upper respiratory tract burns and smoke inhalation. Unlike our broader research, which focused on extensive burns, this study adopted a more targeted approach, applying the tumescent technique exclusively to donor sites during early debridement and split-thickness skin grafting.

Results:

The primary advantages observed were effective hemostasis at the donor site and the creation of a clean operative field. Additionally, the technique contributed to reduced postoperative pain at the donor site. For smaller burns, the tumescent technique improved intraoperative hemostasis, facilitated a clean operative field, and reduced postoperative pain. For larger burns and delayed surgical interventions, it further enhanced hemostasis, minimized blood loss, supported hemodynamic stability, and decreased pain at both donor and treated sites.

Conclusions:

Based on our findings, we propose that the tumescent technique can be safely employed in burn patients requiring surgical intervention, with objectives depending on burn size and severity. It is a safe procedure, without an increased risk of graft loss or infectious complications compared to other interventions.

Keywords: tumescent technique, donor site, skin grafting, burns

*O03.1 Trends in Mortality, Complications, and Resource Utilization in Elderly Burn Patients: A 15-Year Retrospective Cohort Study*

**Little D** ^1^, Ho G ^1^, Tedesco D ^2^, Jeschke M ^2,3,4^, Shahrokhi S ^3,4^, Elloso M ^2,3,4^

^1^ Division of Plastic, Reconstructive & Aesthetic Surgery, Department of Surgery, University of Toronto, Toronto, Canada, ^2^ Centre for Burn Research, Hamilton Health Sciences, Hamilton, Canada, ^3^ Burn Program at Hamilton Health Sciences, Hamilton, Canada, ^4^ Department of Surgery, McMaster University, Hamilton, Canada

Oral presentations 03—Geriatrics & Infections, 3 September 2025, 15:45–17:15

Aim:

This study examined inpatient outcomes over a 15-year period in elderly burn patients, a vulnerable group prone to complications and poor outcomes.

Methods:

A retrospective cohort study was conducted at a provincial burn center, including all patients aged ≥60 years admitted between 2007–2021. Patients were grouped into three 5-year admission cohorts: early (2007–2011), middle (2012–2016), and late (2017–2021) to evaluate trends in outcomes over time. The primary outcome was 30-day mortality. Secondary outcomes included inpatient complications, length of stay (LOS), 30-day readmission, and resource utilization. Multivariable logistic regression and negative binomial regression models were used to adjust for age, sex, and total body surface area (TBSA) affected.

Results:

Among 607 patients (mean [SD] age 72 [9] years, 63% male), 30-day mortality remained stable across cohorts (14% in late, 16% in middle, 15% in early; *p* > 0.05). However, after adjusting for patient characteristics, the odds of sepsis decreased by 71% (*p* = 0.001) and respiratory complications by 59% (*p* = 0.031) in the late cohort compared to the early cohort. Mechanical ventilation use also declined significantly (*p* = 0.02). There were no significant differences in LOS, number of surgeries, or readmission rates between cohorts.

Conclusions:

Over 15 years, elderly burn patients experienced significant reductions in sepsis, respiratory complications, and mechanical ventilation use. However, mortality and readmission rates remained unchanged, underscoring the need for continued advancements in acute burn care and post-discharge management to improve long-term outcomes in this high-risk population.

*O03.3 ICIS is an excellent marker to differentiate infectious from non-infectious causes of inflammation in patients with thermal injury*

**Bakalar B** ^1^, Zajíček R ^1^, Lahoda Brodská H ^2^, Adámková V ^2^, Tomek J ^3^, Fridrichová M ^4^

^1^ Prague Burn Centre, Charles University 3rd Faculty of Medicine and University Hospital Kralovske Vinohrady, Prague 10, Czech Republic, ^2^ Institute of Medical Biochemistry and Laboratory Diagnostics, The First Faculty of Medicine of Charles University and The General University Hospital, Prague, Czech Republic, ^3^ Department of Pharmacology of University of California, Davis, USA, ^4^ Department of Microbiology, Central Laboratories, The Third Medical Faculty, Charles University and The Kralovske Vinohrady University Hospital, Prague, Czech Republic

Oral presentations 03—Geriatrics & Infections, 3 September 2025, 15:45–17:15

Aim:

To compare Intensive Care Infection Score (ICIS) with several biomarkers of inflammation, namely White Blood Counts (WBC), C-reactive Protein (CRP), procalcitonin (PCT), lipopolysaccharide-binding protein (LBP), presepsin (PES), and calprotectin (CAL) in patients with thermal injury.

Methods:

A prospective single-centre observational study. A total of 60 patients with thermal injuries admitted to ICU were selected for the study and 181 blood samples were analyzed in the presence and absence of infection.

Results:

The highest discriminatory power for inflammation of infection or non-infection origin was observed for ICIS, with an area under the curve of 0.84 (*p* < 0.0001), while the lowest was recorded for WBC at 0.39 (*p* = NS). The ICIS cutoff for infection was determined to be 5, with a sensitivity of 75% and a specificity of 76%.

Conclusions:

The study demonstrated the non-inferiority of ICIS to CRP, PCT, CAL, PES and LBP, and its superior ability to discriminate between inflammation of infectious and non-infectious origin, as well as its ability to predict sepsis. The cost-effectiveness, speed of testing and availability make ICIS an almost ideal biomarker of infection in burns.

*O03.4 Therapeutic drug monitoring (TDM)—what are the benefits?*

**Gille J** ^1^, Vögeler S ^1^, Kremer T ^1^, Sablotzki A

^1^ Klinikum St. Georg, Leipzig, Germany

Oral presentations 03—Geriatrics & Infections, 3 September 2025, 15:45–17:15

Aim:

Therapeutic drug monitoring (TMD) is used for the individualised dosing of antibiotics. The aim of the study was to identify risk factors for both low and high serum levels and to investigate the influence on dosage.

Methods:

This prospective observational study included patients who were admitted to a regional burn center treated with beta-lactam antibiotics. Serum levels were determined and factors influencing the level of antibiotics were analyzed. In addition, it was determined how often dose adjustments were made in the context of the TDM.

Results:

A total of 105 burn patients were included in this study. A total of 173 infections occurred that were treated with: penicillins 94 (54.3%), cephalosporins 36 (20.8%), carbapenems 43 (24.9%). According to the MIC target range, the distribution of serum levels was as follows: <MIC 1.3%; >MIC ≤ 4 x MIC 6.5%; >4 x MIC ≤ 8 x MIC 9.1%; >8 x MIC < toxic 71.4%, toxic 11.7%. Gender, age, BMI and burn severity (ABSI) had no influence on over- or underdosing. Renal insufficiency was associated with the risk of overdosing (*p* = 0.005). An overdose was significantly more frequent with carbapenems (*p* < 0.001). In 53.3% of cases, a dose adjustment took place after determination of the initial level (dose increase 5.8%, dose decrease 47.5%).

Conclusions:

TDM has an influence on the dosage of antibiotics. TDM appears to be particularly useful in patients with renal insufficiency and when using carbapenems.

*O03.5 Toxic shock syndrome in paediatric burn patients*

**Phillips G** ^1^, Ibrahim A ^1^, Shahrabi Atari D ^2^, Marks M ^1^, Markeson D ^1^, Jones I ^1^, Moore L ^1^, Collins D ^1^

^1^ Chelsea and Westminster NHS Foundation Trust, London, United Kingdom, ^2^ Imperial College London, London, United Kingdom

Oral presentations 03—Geriatrics & Infections, 3 September 2025, 15:45–17:15

Aims

Toxic shock syndrome (TSS) is a rare but serious complication of burns injury in the paediatric population, studies exploring TSS in this population to date are small. We performed a retrospective observational study over a 5 year period to determine the incidence and mortality of patients admitted with potential toxic shock syndrome, predictive features in presentation, common alternative diagnosis’, microbiological findings and present our treatment algorithm.

Methods:

Clinical and laboratory data was collected from paediatric burns initially treated for TSS over a 5-year period. Patients were characterised according to their final diagnosis.

Results:

We present data on the number of patients admitted with assumed toxic shock syndrome (from preliminary data 39 patients over a 16 month period), how many retained this diagnosis and common alternative diagnosis’ made following assessment and investigation. We outline key demographic information in patients with confirmed diagnosis of toxic shock including burn% TBSA, patient age and time since burn injury. *Staphylococcus aureus* is the main microbiological cause. Methicillin resistance is a concern in this population. In addition, we compare patients presenting signs and symptoms to the abbreviated diagnostic criteria for toxic shock syndrome.

Conclusions:

This is the largest study to date of the incidence, presentation and management of children with toxic shock syndrome secondary to burn injury.

*O03.6 Challenges posed by multidrug-resistant organisms in the treatment of severely burned patients from romania in a german burn center*

**Rautenbach S** ^1^, Boos A ^1^

^1^ Universitätsklinikum Schleswig-Holstein (Campus Luebeck), Luebeck, Germany

Oral presentations 03—Geriatrics & Infections, 3 September 2025, 15:45–17:15

Aim:

The report details the experience of the Burn Center of the University Hospital Schleswig-Holstein, Campus Lübeck, with the admission and management of patients transfered from Romania, focusing on multidrug-resistant organisms and aiming to provide insights into the logistical, clinical, and infection control aspects to improve outcomes.

Methods:

We conducted a retrospective review of the transfer and treatment records of 18 patients admitted between 2022 and 2024. Our analysis focused on the incidence of MDRO contamination upon arrival, the types of resistant organisms identified, and the subsequent impact on treatment strategies and patient outcomes.

Results:

The management of severely burned patients transferred from Romania presents considerable logistical and clinical challenges, placing significant demands on the entire treatment team. Transfers often occur at a late stage in the patient’s treatment, increasing the complexity of care. A substantial proportion of patients (56%) were found to be colonized with *Acinetobacter baumannii*. This highlights the need for enhanced infection control measures and strategies to minimize the risk of cross-contamination.

Conclusions:

While the treatment of severely burned patients from Romania in Germany is formally addressed by existing EU directives, further optimization is needed through enhanced international collaboration and standardized protocols. Addressing the high rate of MDRO contamination requires a reevaluation and potential adaptation of hygiene concepts to prepare for treating these patients in the context of emerging global crises. Future strategies should focus on early detection of MDROs.

*O03.7 Fish Skin Transplantation Type Kerecis Saves Patient From A Below Knee Amputation While Preserving His Left Foot*

**Sharafi S** ^1^, Leenhouts P ^1^, Arigoni M ^1^, Schreiber V ^1^

^1^ Spital Uster Ag, Uster, Switzerland

Oral presentations 03—Geriatrics & Infections, 3 September 2025, 15:45–17:15

Aim/Background:

We saved a 62y Swiss-Serbian from a below knee amputation while preserving his left foot after trauma due to a fallen tree. The fracture of Dig1 left was initially fixed with k wires in Serbia, postop. patient developed local infection of Digit1 and 2 following amputations of Dig1 and 2 in Serbia and partial amputation of Dig3 (1OR). Postoperatively the wound developed further progress with wound contamination-infection. Patient returned to Switzerland for further treatment and presented at our ED with terrible and highly contaminated wound status. Structures like bone and tendons were seen under necrosis and palpable, clinical and radiological signs indicated MRSA pos. microbiology and osteomyelitis.

Methods:

- 22.11.2024 amputation Dig1 on Lisc Frank niveau with metatarsal amputation 2–5 left
- 02.12.2024 Transplantation 10 × 7 cm Kerecis graft (one application only)
- VAC therapy for 2 weeks
- 10.2 patient last seen, can walk, rehab will follow, orthopedic shoes individually set up for patient

Results:

Great outcome in a tricky and dangerous situation with multi resistant contamination (MRSA) in addition to diabetes, arteriosclerosis and previous operations in Serbia while dramatically progressing and worsening locally after treatment in Serbia.

Conclusions:

We saved the patients extremity and could preserve his feet and consolidated this highly contaminated situation and prevented further maceration, necrosis and wound dehiscence or further amputations.

Kerecis regenerated deeper and missing soft tissues sufficiently without further complications which shows his potency and bactericide effect and barrier against multi resistant bacterias. Overall treatment time, patients pain as well as overall costs could be reduced.

*O03.9 Challenges in blood culture diagnosis: positive results in severely burned patients without sepsis*

**Zagorac D** ^1^, Skunca A ^1^

^1^ Sestre Milosrdnice University Hospital Center, Zagreb, Croatia

Oral presentations 03—Geriatrics & Infections, 3 September 2025, 15:45–17:15

Aim:

Bloodstream infections (BSI) are a leading cause of morbidity and mortality in hospitalized patients worldwide. The detection of microorganisms in blood cultures is crucial for both diagnosis and prognosis, serving as one of the primary functions of diagnostic microbiology laboratories.

Methods:

We are presenting two cases of patients treated in the Burn Intensive Care Unit (BICU) who sustained 50% total body surface area (TBSA) burns and developed positive blood cultures during their hospitalization, despite not receiving treatment for the detected pathogens. The first case involves a 57-year-old male with Gram-negative bacteria isolated from both the central venous catheter and arterial line. The second case involves a 56-year-old female, where both Gram-positive and Gram-negative bacteria were detected from similar sites. In both instances, blood cultures were obtained while the patients were off antimicrobial therapy, had been in the BICU for more than 14 days, and showed no clinical signs of ongoing sepsis. Their laboratory results, including leukocyte count, procalcitonin, and lactate levels, were normal, and they exhibited hemodynamic stability and good overall appearance.

Results:

Both patients ultimately experienced positive outcomes, with full recovery and no further complications from the detected microorganisms.

Conclusions:

Obtaining blood cultures in severe burn patients poses significant challenges, particularly with the frequent use of vascular catheters as sampling sites, which increases the risk of contamination. Monitoring blood culture contamination is essential as contamination can lead to false-positive results, unnecessary antimicrobial therapy, prolonged hospital stays, additional diagnostic tests, and increased healthcare costs.

*O04.1 Air ambulance transfers in pediatric burn care*

**Abay A** ^1^

^1^ Ankara Bilkent City Hospital, Ankara, Türkiye

Oral presentations 04—Pediatrics 1, 4 September 2025, 08:30–10:00

Aim:

Severe burn injuries have high mortality, with 75% of deaths occurring at the scene or during transfer. This study examines the impact of transportation-related factors on clinical outcomes in pediatric burn patients.

Methods:

We retrospectively analyzed pediatric burn patients treated over the past five years, evaluating demographics, transfer routes, distances, and clinical outcomes. Patients transported by air ambulance were compared with those transferred by ground. Statistical significance was set at *p* < 0.05.

Results:

Of 676 referred patients, 12 transferred from abroad in the late post-burn period were excluded. Among the remaining 664 patients, 61.9% came from outside of our city (60 different cities and Syria). Most arrived by ground ambulance (92.6%), while 6.5% were transported by plane and 0.9% by helicopter. Of the 58 (8.7%) intubated patients, 46.6% were transported by plane and 5.2% by helicopter. Air ambulance patients had higher rates of flame (55.1%) and electrical burns (14.3%). Compared to ground transport, they had larger burn surface areas (38.4 ± 24.3%), higher inhalation injury rates (42.9%), longer hospital stays (47.4 ± 39.7 days), and increased mortality (26.5%), with all parameters showing a statistically significant difference (*p* < 0.001). Mortality also correlated with transfer distance (*p* < 0.001).

Conclusions:

Burn severity, airway status, and transport conditions greatly affect outcomes. Air ambulance is vital for critically burned patients, particularly over long distances. Rapid transfer and pre-transfer stabilization improve survival. Strengthening triage, referral networks, and air ambulance access can reduce mortality and enhance pediatric burn care. Policymakers should prioritize these improvements in transfer protocols.

*O04.2 Combination biotechnology dressings for paediatric burns: a retrospective study*

**Casas Beltran J** ^1^

^1^ Mexican Institute of Social Security, Hermosillo, Mexico

Oral presentations 04—Pediatrics 1, 4 September 2025, 08:30–10:00

Aim:

There is still debate about the use of first-line dressings in the acute management of burns. There are studies that show that recovery time is a determining factor in the functional and aesthetic prognosis of patients. Our aim is to demonstrate the benefits of the biotechnology combination for partial thickness burns in children.

Methods:

We performed a retrospective analysis of children with partial thickness burns who were treated in our burn unit between January 2023 and December 2024. A total of 67 patients were included in the study. Patients were treated with a combination of bacterial nanocellulose (Epicite hydro^®^, BIOSKINCO, Tlalnepantla de Baz, Mexico) and cultured cryoprecipitate human keratinocytes (Epifast^®^). Different areas were covered. The follow-up for long-term healing assessment was one-year post-burn.

Results:

The average length of stay in the hospital was 5 days and there were no recorded cases of infection. 93% of cases required only surgery and a single treatment. Outpatient management was performed in 34% of the cases. There were 3 cases of deepened burns, required grafting. Long term results showed an average score of 0 on the Vancouver scar scale.

Conclusions:

The use of combined biotechnology in paediatric partial thickness burns favors rapid recovery of patients with only one treatment, allowing reduction of hospital days with the option of outpatient management. This treatment prevents infection, helps prevent deepening and avoids grafting. The long-term results confirm that the reduction in recovery time has a direct impact on the aesthetic and functional outcome of patients.

*O04.3 Skin Grafting as a Preventive Strategy Against Infections in Pediatric Burns*

Cocchi E ^1,3^, Cassalia F ^2^, Melandri D ^1,3^

^1^ University of Bologna, Bologna, Italy, ^2^ University of Padova, Padova, Italy, ^3^ AUSL Romagna, Bufalini Hospital, Cesena, Italy

Oral presentations 04—Pediatrics 1, 4 September 2025, 08:30–10:00

Background: 

Infectious complications are a significant cause of morbidity and mortality in deep burn patients, especially pediatric ones. While autologous split thickness skin grafting is commonly used after escharectomy to promote wound healing, its effect on reducing infection rates and the need for antibiotics in this population is not well understood.

Objective:

This study aimed to evaluate the impact of skin grafting on the incidence of wound infectious complications, systemic inflammatory response syndrome (SIRS) development, and the need for antibiotic treatment, in pediatric patients.

Methods:

A retrospective cohort of 123 pediatric burn patients treated at Bufalini Hospital, Cesena, Italy, between 2018 and 2024 was analyzed. Inverse probability of treatment weighting (IPTW) was used to balance covariates. Cox proportional hazards models were applied to assess the influence of grafting on the time to wound infectious complications, SIRS development, and need for antibiotic treatment. Sensitivity analysis using both time-insensitive logistic models and leave-one-out approach were performed to assess robustness of findings.

Results:

Skin grafting significantly reduced the risk of wound infectious complications (Hazard Ratio = 0.12, 95% CI: 0.02–0.58, *p* < 0.01) and SIRS development (Hazard Ratio = 0.14, 95% CI: 0.03–0.73, *p* = 0.02). A non-significant trend was also identified for the need of antibiotic treatment. Sensitivity analysis confirmed the robustness of the results.

Conclusions:

Autologous split thickness grafting plays a critical role in preventing infectious complications in pediatric burn patients. These findings suggest that skin grafting should be prioritized in pediatric burn treatment protocols.

*O04.4 Deep IV sedation for pediatric burn wound dressing changes in an intermediate day care unit led by physician assistants anesthesiology: a retrospective analysis of a multidisciplinary sedation and analgesia program*

**Leeuwerik M** ^1^, Prudon I ^1^, Coenen W ^1^, van de Korput M ^1^, Bollen J ^1^

^1^ Radboud University Medical Center Amalia children’s hospital, Nijmegen, Nederland

Oral presentations 04—Pediatrics 1, 4 September 2025, 08:30–10:00

Aim:

Describing the safety, efficiency, and clinical approach of our individualized pediatric deep IV sedation program for burn wound dressing changes. The program, led by physician assistants anesthesiology, is part of a multidisciplinary team and utilizes a unique comfort care method specifically tailored for pediatric burn patients.

Methods:

We conducted a retrospective analysis of our deep sedation protocol outside the operating room, focusing on pediatric burn patients treated between Q1 2024 and Q2 2025. Inclusion criteria: age > 9 months and weight > 9 kg. We assessed the number and type of sedation sessions (scheduled and ad hoc), sedation duration, and adverse events. Also, we describe the framework of our individualized, multidisciplinary care—featuring child life specialists, parental involvement during induction and redressing, and various preparatory strategies—as part of a child- and family-centered approach. This comfort care model is unique in The Netherlands.

Results:

Preliminary data from 250 sedation sessions demonstrate that deep IV sedation, led by PA anesthesiology in an intermediate pediatric day care setting, is safe, efficient, and effective. Our comfort care method is adapted to both outpatient and inpatient needs, integrating EMLA for IV placement, language reframing, distraction techniques, child life support, and parental presence. Optional sedation with N2O or mask induction with Sevoflurane is available prior to IV placement, outside the OR.

Conclusions:

Our customized sedation and analgesia approach for pediatric burn wound care offers a safe and patient-centered alternative outside the OR. This model may serve as a framework for implementation in other pediatric burn centers.

*O04.5 Burn wounds depth evaluation in the pediatric population using laser speckle contrast imaging—retrospective study*

**Pashov I** ^1^, Argirova M ^1^, Martinov M ^1^

^1^ Umhatem “N. I. Pirogov”, Sofia, Bulgaria

Oral presentations 04—Pediatrics 1, 4 September 2025, 08:30–10:00

Aim:

The aim of this study is to present our experience evaluating burn depth in various types of burns in the pediatric population, measured with laser speckle contrast imaging (LSCI), and its relation to the time of epithelialization and the need for surgical intervention.

Methods:

A retrospective analysis was conducted, including 135 pediatric patients with burn injuries of various types and localizations, treated between November 2022 and March 2025. Demographics were collected from hospital records. The mean age of the patients, measured in months, is 28 ± 53.05. The mean TBSA is 0.03 ± 0.07. Perfusion was measured between 12- and 96-h post-injury using LSCI. On the same occasions, burn surgeons assessed the burns as healing <14 days or healing >14 days/surgery. A total of 210 regions of interest (ROI) were collected.

Results:

Significant variation in perfusion patterns was observed across different burn depths. Superficial burns demonstrated higher perfusion values, while deep partial-thickness and full-thickness burns exhibited reduced perfusion with no significant changes over time. The mean perfusion for the burn injuries that healed <14 days was 347 PU ± 166, and 207.52 PU ± 63.12 for burn injuries that healed >14 days. Mean perfusion values for wounds that underwent surgery were 133.2 PU ± 54.16.

Conclusions:

Laser speckle contrast imaging proves to be a reliable, non-invasive tool for evaluating burn wound depth and monitoring perfusion in pediatric burn patients.

*O04.7 Reconstructive surgery after hand burns in children and adolescents: a descriptive study of a Chilean cohort in outpatient rehabilitation center*

Quezada M ^1^, Espinosa M ^1^, Dìaz J ^2^

^1^ Surgery Team, Corporación De Ayuda Al Niño Quemado. COANIQUEM, Santiago, Chile, ^2^ Dirección de Extensión, Docencia e Investigación, DEDI. Corporación de Ayuda al Niño Quemado, COANIQUEM., Santiago, Chile

Oral presentations 04—Pediatrics 1, 4 September 2025, 08:30–10:00

Aim:

Hand burns in children often result in sequelae with functional consequences that may worsen during growth. This study aims to describe the demographic and clinical characteristics and reconstructive procedures performed on the hands of children and adolescents with burn sequelae in an outpatient burn center.

Methods:

A retrospective observational study was conducted, analyzing data from patients who underwent reconstructive hand surgery between 2011 and 2016. Descriptive statistics were used to summarize the population and procedures. Ethical approval was granted by the Ethics and Scientific Committee of COANIQUEM.

Results:

A total of 394 patients underwent 461 surgeries. Most patients (85.3%) were under 2 years old at the time of the burn injury, with contact burns being the primary cause (67.1%). In 85.3% of cases, total body surface area (TBSA) affected was <2%. The average interval between injury and first reconstructive surgery was 2.2 years. Common techniques included local flaps (48.2%), wing flaps (25.4%), and contracture release with skin grafting (23.1%). Combined techniques were required in 26.5% of surgeries, while complications occurred in 7.4%.

Conclusions:

Most reconstructive surgeries were performed during the growth period, emphasizing that even small burns can result in sequelae. Surgical techniques used are safe and feasible in outpatient settings. Further research is needed to identify hand areas prone to complex sequelae and greater surgical needs.

*O04.8 TEN (toxic epidermal nekrolysis)—Testimonials from a pediatric burn center*

**Wendenburg, W.** ^1^, Guarnizo Dias, S., Pohle, R., Klein, T., Boemers, T.

^1^ Childrens Hospital Cologne, Cologne, Germany

Oral presentations 04—Pediatrics 1, 4 September 2025, 08:30–10:00

Background:

Toxic epidermal necrolysis (TEN) is a severe variant of a rare immunological blistering reaction with over 30% of body surface area affected. It begins with cockade-like erythema, leading to detachment of the skin and mucous membranes and a systemic reaction (1).

It occurs with an incidence of 1–2:1 million people and is significantly less common in children. The risk of death is approximately 45% in adults, 6% in children. However, due to the rare nature of the disease, the data are difficult to classify, and most cases are presented in the literature. In this regard, we would like to present our four cases.

Materials and methods:

A retrospective presentation of the clinical reports of this condition from our Children’s Hospital Cologne from 2012 to 2025 with different clinical outcomes.

Results and Conclusions:

It is recommended that TEN be treated in a specialized burn center with a multidisciplinary team. Supportive care represents the gold standard (1). Prolonged intensive care treatment is required. The diagnosis is clinical and should be confirmed by a skin biopsy. Regarding the etiology, half of cases are drug-related; the other half infection-induced or idiopathic (1, 6, 7).

Local treatment is similar to burn therapy. The transfer to a burn center is often necessary. The short-term use of corticosteroids is recommended. In cases of progression, the use of immunomodulated therapy has been described (1,6).

The prognosis depends on the affected body surface area, accompanying symptoms, and systemic spread. Early detection of TEN is beneficial for therapeutic intervention.

*O04.9 Discrepancies in the Estimations of Burn Depth and Burned Surface Area from First Responders to Burn Surgeons: A Retrospective Study from a German Burn Center*

**Barros Navarro, L.** ^1^, Vogt, P., Enechukwu, A., Suhr, L., Milewski, M.

^1^ Medizinische Hochschule Hannover, Hannover, Germany

Oral presentations 04—Pediatrics 1, 4 September 2025, 08:30–10:00

Introduction:

Accurate assessment of total body surface area burned and burn depth is crucial for appropriate triage and referral to specialized burn centers. However, discrepancies in early evaluations by first responders and referring physicians may lead to unnecessary transfers and overburdening of the limited resources in burn centers.

Methods:

A retrospective, single-center study included 360 patients transferred to a specialized burn center between June 2020 and June 2024. Initial assessments by emergency personnel were compared with final evaluations performed by experienced burn surgeons. The analysis focused on differences in TBSA, burn depth, and suspected inhalation injury. Statistical analyses included Wilcoxon, Kruskal-Wallis, and regression models.

Results:

There was a significant overestimation of TBSA prior to referral (median 10% vs. 6%, *p* < 0.001), with greater discrepancies observed in the evaluation of both depth and TBSA in full-thickness burns compared to partial-thickness burns. Variability in burn depth classification was significantly associated with the type of initial evaluator; paramedics demonstrated the lowest deviation (mean difference: 4.13%). Anatomical regions such as the groin, feet, and legs showed the highest variability. BMI correlated positively with discrepancies in TBSA estimation Inhalation injury was suspected in 18.3% and confirmed in 17.2% of cases; 21.1% of patients were intubated upon admission.

Conclusions:

This study highlights a systematic overestimation of burn extent and inconsistencies in the assessment of burn depth. Targeted training for all levels of prehospital and emergency care providers is essential to improve evaluation accuracy and optimize referrals to burn centers, ultimately enhancing patient care.

*O05.1 The first reader study of burn wounds with predictive artificial intelligence analysis*

**Miles, V.**, Carter, J. ^1,2^, Di Maio ^3^, Chadwick, P. ^4^

^1^ Louisiana State University Health And Science Centre, New Orleans, United States, ^2^ University Medical Center (LSU Health), New Orleans, United States, ^3^ Baylor Scott & White Health, Dallas, United States, ^4^ Spectral AI, London, United Kingdom

Oral presentations 05—Wounds 2, 4 September 2025, 08:30–10:00

Aim:

Burn wounds are complex injuries that present significant challenges especially when assessing wound severity and healing potential or surgical treatment. To date, no widespread diagnostic technology effectively diagnoses or predicts burn wound healing. A potential solution exists with multispectral imaging (MSI) when processed with artificial intelligence (AI) algorithms. The primary objective of this study was to determine the impact of MSI and AI on clinicians’ treatment decisions.

Methods:

Enrolled were 54 emergency medicine and 46 burn surgeons as independent assessors in a reader study design. A mixed model predicting optimal treatment assignment incorporated 10 different healing and non-healing burn wound images (BWI). Participants independently made treatment decisions before and after AI output on BWI, which highlighted non-healing areas.

Results:

The probability of making a correct assessment was estimated at 0.61% before viewing the MSI output. After participants review of the MSI output, the probability of a correct assessment increased to 0.89%. Furthermore, the odds of making a correct treatment decision were estimated to be 3 times higher (*p* < 0.0001). The average difference in composite scores before and after MSI output was significantly improved (*p* < 0.0001). We found no significant differences between provider types (*p* = 0.5332).

Conclusions:

This study demonstrated that MSI with AI is a valuable tool for burn wound assessment, offering high reliability, improved diagnostic accuracy, and greater objectivity compared to traditional methods. This technological advancement could help standardize burn wound evaluation, reducing unnecessary transfers and hospital stays, while offering more prudent surgical intervention

*O05.2 The accuracy of indocyanine green angiography for the assessment of skin viability: a systematic review*

**de Haan, J.** ^1,2^, M.F.H. Neeter, L. ^1^, P.M. van Zuijlen, P. ^1,2,3^, Pijpe, A. ^1,2^, Meij-de Vries, A. ^1,4^

^1^ Alliance of Dutch Burn Care (ADBC), Burn Centre, Red Cross Hospital, Beverwijk, The Netherlands, ^2^ Department of Plastic, Reconstructive and Hand Surgery, Amsterdam UMC Location Vrije Universiteit, Amsterdam, The Netherlands, ^3^ Department of Plastic and Reconstructive Surgery, Red Cross Hospital, Beverwijk, The Netherlands, ^4^ Department of Surgery, Red Cross Hospital, Beverwijk, The Netherlands

Oral presentations 05—Wounds 2, 4 September 2025, 08:30–10:00

Aim:

This systematic review aims to: (1) evaluate the diagnostic accuracy of indocyanine green angiography (ICGA) for assessing skin viability, and (2) compare postoperative outcomes after skin viability assessment with ICGA versus clinical evaluation.

Methods:

This study follows the PRISMA guidelines. A comprehensive literature search was conducted in PubMed, Web of Science, and Cochrane Library, using search terms related to ICGA and skin viability. For the primary objective, studies reporting or providing sufficient data to calculate the diagnostic accuracy of ICGA for skin viability assessment were included. For the secondary objective, studies comparing ICGA with clinical evaluation and reporting postoperative skin necrosis, flap loss, or reoperation rates were included. Risk of bias will be assessed using the QUADAS-2 tool for diagnostic accuracy studies, the Newcastle-Ottawa Scale for cohort studies, and the Cochrane Risk of Bias Tool for randomized controlled trials. Screening and data extraction will be conducted independently by two reviewers.

Results:

This study was registered in PROSPERO. A total of 201 articles were identified during title and abstract screening. After full-text screening, data needed to calculate the sensitivity and specificity of ICGA will be extracted from the articles. Meta-analyses will compare ICGA with clinical evaluation in terms of postoperative skin necrosis, flap loss, and reoperation rates.

Conclusions:

Skin viability assessment is essential in debridement and reconstructive surgery. If ICGA proves to be accurate, it may have potential to improve the accuracy of skin viability assessment and guide clinical decision-making in burn and reconstructive surgery, possibly improving patient outcomes.

*O05.3 A skin to live in: a case of multistage facial reconstruction after acid assault*

Serracanta Domenech, J. ^1^, Rivas Nicolls, D. ^1^, Bulla, A. ^1^, Lopez Martinez, J. ^1^, **Gonzalez Peña, F.** ^1^, Orois Gomez, S. ^1^, Iglesias Alvarez, A. ^1^, Cuadros Ramirez, M. ^1^, Barret Nerin, J. ^1^

^1^ Hospital Universitario Vall D’hebron, Barcelona, Spain

Oral presentations 05—Wounds 2, 4 September 2025, 08:30–10:00

Aim:

To describe a multistage surgical reconstruction process followed to restore a facial chemical burn.

Methods:

A case of a 32-year-old male patient who sustained a right hemifacial burn due to an acid assault. A stepwise surgical reconstruction was performed over a 3-year period and included wound debridement, application of acellular dermal matrices, skin grafting, secondary refinement procedures and a helix root free flap.

Results:

The patient underwent a successful multistage reconstruction with stable grafts and flap integration. Functional achievements included preserved eyelid closure, maintained oral competence and acceptable facial symmetry. The patient consistently reported high satisfaction with both functional and aesthetic outcomes during follow-up evaluations.

Conclusions:

Facial chemical burns often require complex and individualized reconstructive strategies to achieve a proper restoration of the injury. The combination of the aforementioned techniques resulted in satisfactory functional and aesthetic outcomes in this patient.

*O05.5 Lessons learnt managing large burn wounds using RECELL^®^ in a single UK burns unit: A case series*

**Macleod, C.** ^1^, Murray, A. ^2^

^1^ University Of Oxford, Oxford, United Kingdom, ^2^ Buckinghamshire Healthcare NHS Trust, Aylesbury, United Kingdom

Oral presentations 05—Wounds 2, 4 September 2025, 08:30–10:00

Aim:

We present a review of RECELL^®^ (Avita Medica, Santa Clara, CA, USA) use at a single UK regional burns unit following promising cost-benefit publications.

Methods:

Patients were treated at a single UK regional burns unit. Order information for RECELL^®^ kits was used to identify patients from June 2023 to March 2025. Information collected included: patient demographics, clinical course, mechanism of burn, total body surface area (TBSA), depth of burns, number of RECELL^®^ applications, length of stay (LOS), complications and follow-up.

Results:

Four patients were identified who were treated with RECELL^®^. Average age was 39 years (range 32–52 years). Three of the patients had a Fitzpatrick skin type four or higher. All four patients suffered full thickness flame burns. Average TBSA was 30% (range 22–40%). Average LOS was 94 days (range 57–184 days). Average days per TBSA was 3.24 (range 1.43–6.13), reflecting BTM’s four-week integration period. RECELL^®^ was used in six operations. RECELL^®^ was used in isolation, with 1.5:1 split thickness skin graft(s) (SSG) or 1:3 SSG on top of integrated BTM. In all operations where a SSG was taken, RECELL^®^ was applied to the donor site(s) with noticeable benefit. All four patients survived, two have undergone scar improving procedures.

Conclusions:

Serious consideration should be given to RECELL^®^ for the management of large, full thickness burns. Our experience mirrored larger studies demonstrating reduced donor site morbidity and better skin colour matching in cases where RECELL^®^ was used. Larger datasets are necessary to specify and validate parameters for using RECELL^®^ in UK burns patients.

*O05.6 Autologous skin cell suspension without split-thickness skin graft is associated with reduced length of stay in second degree burns: Results from a US national registry*

**Miles, V.** ^1^, Schoen, J. ^1^, Phillips, B. ^2^, Carter, J. ^1^

^1^ University Medical Center New Orleans, New Orleans, Louisiana, United States, ^2^ BData, Inc, Minneapolis, Minnesota, United States

Oral presentations 05—Wounds 2, 4 September 2025, 08:30–10:00

Aim:

This study compares hospital length of stay (LOS) in adult second-degree burn patients who received meshed split-thickness skin graft (STSG) or autologous skin cell suspension (ASCS).

Methods:

The ABA Burn Care Quality Platform registry was queried for adults (≥15 years) with second-degree burns treated with STSG or ASCS from 1 Januray 2019 to 30 September 2024, excluding those with documented non-burn-related trauma, discharged as deceased or to hospice, those without burn-related etiology and non-documented or outlying LOS (above mean + 2SD). Cases were matched 2:1 on sex, age, total body surface area (TBSA), and inhalation injury. Matching was performed within TBSA bands with a width of 10%. Statistical comparisons of LOS were conducted using Welch’s *t*-test.

Results:

A total of 723 cases were matched. Insufficient sample size limited TBSA bands to <30%. Across all TBSA bands, average LOS was significantly shorter for the ASCS cohort compared to the STSG cohort (*p* < 0.05). Average LOS decreased by 4.1 days for 0–9.9% TBSA burns (*p* < 0.0001), 4.9 days for 10–19.9% TBSA burns (*p* < 0.0001), and 6.9 days for 10–29.9% TBSA burns with ASCS treatment.

Conclusions:

In addition to its known benefits of significantly reducing donor site size, pain, and scarring, independent use of ASCS significantly shortens LOS in second-degree burn adult patients, compared to STSG. Reduced LOS with ASCS may improve outcomes and lower healthcare costs for this population.

*O05.7 Innovative Approaches in Burn Treatment: Silver-Based Dressings, Smart Biomaterials, and Regenerative Solutions*

**Parlak, R.**, Bulut, E., Tekin, O., Öz, K., Karakol, P.

^1^ Başakşehir Çam and Sakura City Hospital, Istanbul, Turkey

Oral presentations 05—Wounds 2, 4 September 2025, 08:30–10:00

Aim:

This study aims to evaluate the effectiveness of contemporary wound dressings and regenerative agents used in modern burn care.

Methods:

A comprehensive literature review was conducted using PubMed, Scopus, and Web of Science databases, focusing on clinical and preclinical studies published in the past decade. Included products encompassed silver-based dressings (Acticoat, Smith & Nephew, London, UK, Aquacel Ag, Convatec, Reading, UK), polymer-based dressings (PolyMem, Fort Worth, TX, USA), epithelialization-enhancing agents (Proheal), and novel smart biomaterials.

Results:

Acticoat, containing nanocrystalline silver, demonstrated rapid bactericidal activity, while Aquacel Ag provided effective infection control via sustained ionic silver release (1). PolyMem was associated with improved exudate management and analgesic effects in painful wounds (2). Proheal was shown to promote epithelialization and accelerate wound closure (3). Emerging smart biomaterials, such as responsive hydrogels and nanofiber systems, exhibited adaptability to dynamic wound environments and supported optimized healing.

Conclusions:

Silver-based dressings and multifunctional wound care products significantly contribute to infection control and healing in burn management. Smart and regenerative approaches represent a promising future for personalized and effective burn care. Further clinical validation is essential for their integration into routine practice.

Keywords: burn treatment, smart dressings, silver-based wound care, regenerative medicine

References:

1. Leaper, D.J. Silver dressings: their role in wound management. *Int Wound J*. **2006**, *3*, 282–294.
2. Woo, K.Y.; Coutts, P.M.; Price, P.; et al. A randomized crossover investigation of pain during dressing changes comparing traditional foam and PolyMem dressings. *Adv. Skin Wound Care* **2009**, *22*, 304–310.
3. Yastrub, D.J. Clinical use of a novel, collagen-containing topical agent for hard-to-heal wounds: Proheal case series. *Wounds* **2017**, *29*, E13–E18.

*O05.8 Development and implementation of a decision aid for early versus late/no surgery for patients with deep partial-thickness burns*

**Salemans, R.** ^1,2^, Verwilligen, R. ^3,4,5^, Geelen, S. ^6,7,8^, van Baar, M. ^1,9^, The R ^10^, Joosten, K. ^1,11^, Lucas, Y. ^1^, Meij-de Vries, A. ^3,12,13^, Scholten-Jaegers, S. ^6^, van der Vlies, C. ^1,2,14^

^1^ Alliance of Dutch Burn Care (ADBC), Burn Centre, Maasstad Hospital, Rotterdam, The Netherlands, ^2^ Trauma Research Unit Department of Surgery, Erasmus MC, University Medical Centre Rotterdam, Rotterdam, The Netherlands, ^3^ Alliance of Dutch Burn Care (ADBC), Burn Centre, Red Cross Hospital, Beverwijk, Rotterdam, The Netherlands, ^4^ Amsterdam UMC location VUmc, Department of Plastic, Reconstructive and Hand Surgery, Amsterdam, The Netherlands, ^5^ Amsterdam Movement Science (AMS), Tissue Function and Regeneration, Amsterdam UMC, Amsterdam, The Netherlands, ^6^ Alliance of Dutch Burn Care (ADBC), Burn Centre, Martini Hospital, Groningen, The Netherlands, ^7^ Research group Healthy Ageing, Allied Health Care and Nursing, Hanze University of Applied Sciences, Groningen, The Netherlands, ^8^ University of Groningen, University Medical Centre Groningen, Department for Human Movement Sciences, Groningen, The Netherlands, ^9^ Erasmus MC, University Medical Centre Rotterdam, Department of Public Health, Rotterdam, The Netherlands, ^10^ Development and Implementation of Decision Aids, ZorgKeuzeLab, Delft, The Netherlands, ^11^ Alliance of Dutch Burn Care (ADBC), Dutch Burns Foundation, Beverwijk, The Netherlands, ^12^ Department of Surgery, Red Cross Hospital, Beverwijk, The Netherlands, ^13^ Amsterdam UMC location University of Amsterdam, Pediatric Surgical Centre, Emma Children’s Hospital, Amsterdam, The Netherlands, ^14^ Department of Trauma and Burn Surgery, Maasstad Hospital, Rotterdam, The Netherlands

Oral presentations 05—Wounds 2, 4 September 2025, 08:30–10:00

Aim:

To develop a decision aid for patients with deep partial-thickness burns for the decision to undergo early excision and grafting or wait and potentially avoid surgery, and to implement it into specialised burn care.

Methods:

Needs and preferences of both patients and healthcare professionals were assessed in the three Dutch burn centres. In-depth interviews were conducted with eight former patients with deep partial-thickness burns, and a survey was conducted among 40 healthcare professionals. Content and design of the decision aid were determined in five co-design sessions involving patients, healthcare professionals, and researchers. Following finalisation, a six-month pilot testing period was initiated in the three burn centres.

Results:

The majority of the healthcare professionals (92%) expressed the need for a decision aid, with 40% indicating that the current information provision is inadequate. Patients reported that the decision aid could have been useful for themselves, but mainly emphasized the importance for their relatives. The decision aid consisted of three components: (1) a paper hand-out sheet; (2) an interactive website; (3) a personal summary sheet. After four months of pilot testing, the decision aid was distributed 34 times and completed by 22 patients (participation rate 65%). Patients logged in multiple times and spent an average of one hour on the website.

Conclusions:

The decision aid was designed to facilitate shared decision-making by providing tailored information to patients and healthcare professionals. The pilot testing showed high levels of engagement, demonstrating the potential to support optimal treatment decisions for patients with deep partial-thickness burns.

*O05.9 “EDNX” vs. “SOC”—the “battle” between the two techniques in the treatment of deep dermal and full thickness burns*

**Taskov, P.** ^1,2^, Corodati, D. ^1^, Crainiceanu, Z. ^1,2^

^1^ Burn Unit—Plastic and Reconstructive Surgery Department Casa Austria, County Emergency Clinical Hospital “Pius Branzeu”, Timisoara, Romania, ^2^ University of Medicine and Pharmacy “Victor Babes”, Timisoara, Romania

Oral presentations 05—Wounds 2, 4 September 2025, 08:30–10:00

Aim:

Efficient EDNX debridement and preservation of viable tissues—effective alternative in order to avoid „SOC” tangential excision and grafting.

Methods:

In this comparative retrospective observational study were included 100 patients, divided into two groups.

First group included 33 male and 17 female patients treated with EDNX, average age 47.8 and average TBSA 30.3% from which 18.2% EDNX treated, ABSI (media) 9.1.

Second group included 27 male and 23 female patients treated with SOC, average age 57.5 and average TBSA 25.6% from which 20.2% SOC treated, ABSI (media) 8.5.

Results:

During one-year period, 100 patients were reviewed, observing following:

The EDNX protocol won the “battle” on the following “battlefields”:

- Reduce the need for additional surgical interventions
  EDNX group: 25 patients with spontaneous healing
  SOC group: 5 patients with spontaneous healing
- Reduce costs, hospitalization time and healing time
  EDNX group: 23,8 days (media) hospitalization time
  SOC group: 28,1 days (media) hospitalization time
- Superior scar quality—according to the Vancouver Scar Scale (VSS)
  EDNX group: at 1-year follow-up: 1–2
  SOC group: at 1-year follow-up: 4–6

Conclusions:

Considering the obtained aesthetic and functional results much more satisfactory, reduced costs and hospitalization time, superior quality and elasticity of scars, EDNX treatment seems to be the first option in the treatment of burns.

*O06.1 Contracture Scars and Management in Our Unit: A Case Series and Literature Review*

Guijarro Pérez, C. ^1^, **Bayo Montoliu, M.** ^1^, Heredia Alcalde, I. ^1^, Pérez del Caz, M. ^1^

^1^ H. La Fe, Valencia, Spain

Oral presentations 06—Scars 1, 4 September 2025, 08:30–10:00

Aim:

Contracture scars are a potential complication in burn patients, becoming functionally significant when they affect key areas such as periarticular regions or the face. This case series highlights the utility of dermal matrix in managing such scars.

Methods:

A series of cases with flame-induced burns leading to contracture scars are presented, where conservative measures were insufficient. Debridement of the limiting areas was performed, followed by the application of bilayer dermal matrix (BDM) on the wound bed. In one case, two different BDMs were applied, showing distinct behaviors. A literature review was conducted on PubMed using terms such as “dermal substitute,” “nevelia,” and “integra,” selecting studies that provided insights into BDM behavior on the wound bed.

Results:

Contracture scars are a significant complication in burn patients with functional limitations. BDMs resulted in substantial improvements in both macroscopic and functional quality of the scars. Moreover, differences in the body’s response were observed depending on the BDM used, as reported in the literature, which may lead to varying functional and aesthetic outcomes.

Conclusions:

BDMs are an effective alternative in resolving contracture scars as a burn complication. A systematic comparison between them is necessary to address potential differences in behavior and long-term outcomes.

*O06.2 Experience in the Management of Hypertrophic Burn Scars with Lipofilling and Corticosteroids*

Guijarro Pérez, C. ^1^, **Bayo Montoliu, M.** ^1^, Bedoya Ramirez, W. ^1^, Pérez Del Caz, M. ^1^

^1^ H. La Fe, Valencia, Spain

Oral presentations 06—Scars 1, 4 September 2025, 08:30–10:00

Introduction: Burn sequelae remain a major challenge in Burn Units due to their aesthetic, functional, and psychological impact on patients. One of the most significant complications is hypertrophic scarring, which results from deep dermal damage combined with growth factor dysregulation, leading to abnormal wound healing. This process is driven by chronic inflammation, characterized by excessive extracellular matrix synthesis, predominantly type I collagen, along with increased scar contraction, causing significant functional and aesthetic impairment in burn patients. In response, we have incorporated lipofilling (Nanofat and Microfat) as a valuable tool within the therapeutic arsenal for managing this condition.

Objective:

To describe our management protocol for hypertrophic burn scars using lipofilling (Microfat and Nanofat) and/or corticosteroid infiltration, presenting our case series over the past 13 months at the Burn Unit of Hospital Universitario y Politécnico La Fe (Valencia).

Methods:

A retrospective review was conducted on burn patients with hypertrophic scars treated with lipofilling between 2 January 2023, and 15 March 2024. Photographic evolution before and after the procedure, along with symptomatic improvement, were assessed.

Results:

A total of 25 patients underwent lipografting with Microfat, emulsified fat, and Nanofat (processed with Lipocube^©^, Istanbul, Türkiye) and/or corticosteroid infiltration, showing significant aesthetic, functional, and symptomatic improvement.

Conclusions:

In our experience, fat grafting is a highly effective tool in the treatment of hypertrophic scars, proving to be a valuable technique in burn care units.

*O06.3 Cultured dermo-epidermal skin as treatment option for scar revision surgery: 1 year results of a prospective RCT comparing denovoSkin^TM^ and split-thickness skin grafts.*

Pijpe, A. ^1,2,3^, Kemme, F. ^1,2,3^, Stoop, M. ^1,2^, van Zuijlen, P. ^1,2,3,4^, Meuli, M. ^5,6^, Schiestl, C. ^5,7,8,9^, Hartmann-Fritsch, F. ^5,6^, Kim, B. ^5,10^, Neuhaus, K. ^5,6,7,8,9^, Mujynya, K. ^6^, Ronfard, V. ^6^, Reichmann, E. ^5,7,11^, Böttcher-Haberzeth, S. ^5,7,8,9^, Farkas, M. ^5,7,8,10^, Bressan, J. ^5,7,8,10^, Zamparelli, M. ^12^, Mataro, I. ^13^, Petroccione, C. ^13^, Azzena, B. ^14^, Marino, D. ^6^, **Middelkoop, E.** ^1,2,3^

^1^ Alliance Of Dutch Burn Centres, Beverwijk, Netherlands, ^2^ Amsterdam UMC location Vrije Universiteit Amsterdam, Department of Plastic, Reconstructive and Hand Surgery, Amsterdam, Netherlands, ^3^ Amsterdam Movement Sciences (AMS), Tissue Function and Regeneration, Amsterdam UMC, Amsterdam, Netherlands, ^4^ Department of Plastic Surgery, Red Cross Hospita, Beverwijk, Netherlands, ^5^ University of Zurich, Zurich, Switserland, ^6^ Cutiss AG, Schlieren, Switserland, ^7^ Pediatric Burn Center, Children’s Skin Center, Department of Surgery, University, Children’s Hospital Zurich, University of Zurich, Zurich, Switserland, ^8^ Department of Surgery, University Children’s Hospital Zurich, University of Zurich, Zurich, Switserland, ^9^ Children’s Research Center, University Children’s Hospital Zurich, University of Zurich, Zurich, Switserland, ^10^ Department of Plastic Surgery and Hand Surgery, Burn Center, University Hospital Zurich, Zurich, Switserland, ^11^ Tissue Biology Research Unit, Department of Surgery, University Children’s Hospital Zurich, University of Zuric, Schlieren, Switserland, ^12^ Plastic Surgery and Burns Unit, Santobono Pausilipon National Children’s Hospital, Napoli, Italy, ^13^ Department of Plastic Reconstructive Surgery and BURNS, AORN A. Cardarelli, Napoli, Italy, ^14^ U.O.C Grandi Ustionati Azienda Ospedale University of Padova, Padova, Italy

Oral presentations 06—Scars 1, 4 September 2025, 08:30–10:00

Aim:

To evaluate a bio-engineered skin substitute, denovoSkinTM, in comparison to split-thickness skin grafts in patients needing scar revision surgery.

Methods:

In this intra-patient randomized phase IIb clinical trial, patients with large full-thickness skin defect were enrolled. Two comparable skin defects were randomized to either denovoSkinTM or autologous STSG. The primary endpoint was the POSAS observer score at 3 months post grafting. Secondary outcomes included other scar quality measures at 3 and 12 months post grafting (POSAS, Cutometer, Colormeter), wound healing, infection, ratio covered surface area/donor site surface, adverse events, and quality of life.

Results:

23 patients, 65% male, with a mean age of 37.4 years were enrolled; 16 had previously suffered burns. At 3 months post grafting, the median observer POSAS score was 22 for denovoSkin^TM^ areas and 27 for STSG areas (*p* = 0.019). The median time to wound closure was 63.0 days and 28.5 days in denovoSkin and STSG respectively (*p* < 0.001). Infections occurred for 6 patients (26.1%) in the area treated with denovoSkin^TM^ and for 4 patients (17.4%) in the area treated with STSG (difference ns). The mean ratio covered surface area/donor site surface for denovoSkinTM was 8.5 (SD 4.4), and for STSG 0.9 (SD 0.2) (*p* < 0.001). At 3 and 12 months, POSAS observer scores were significantly better for denovoSkinTM than for STSG for thickness (3 m), relief and pliability (3 and 12 m).

Conclusions:

denovoSkinTM offers a novel and effective treatment option for surgical scar revision, limiting donor site and offering good long term skin quality comparable to normal skin.

*O06.4 POSAS 3.0 (patient and observer scar assessment scale): Translation and cross-cultural validation for Greek-speaking patients and clinicians*

**Pipinia, A.** ^1^, Nikolaidou, E. ^1^, Tsiligeridis, S. ^1^, Petsa, A. ^1^, Joycey, A. ^2^, Papadopoulou, S^1^

^1^ General Hospital of Thessaloniki “g. Papanikolaou”, Thessaloniki, Greece, ^2^ Plastic Surgeon, Thessaloniki, Greece

Oral presentations 06—Scars 1, 4 September 2025, 08:30–10:00

Aim:

The Patient and Observer Scar Assessment Scale (POSAS) is a tool for evaluating scar quality from both patient and clinician perspectives. Our aim was to officially translate and cross-culturally validate the latest POSAS 3.0 edition into Greek, making it accessible for Greek-speaking patients and clinicians.

Methods:

Following guidelines from the Dutch Burn Foundation, the translation process involved forward translation from English to Greek by two native Greek translators, who documented challenges and synthesized a single version under supervision. This version was then back-translated into English by two native English speakers blinded to the original text. An expert committee reviewed all translations and reports to create a pre-final version, which was pilot-tested on 30 patients from the target population. We are currently conducting a cross-cultural validation study with 250 patients following COSMIN guidelines, ensuring the questionnaire’s reliability and equivalence to the original.

Results:

The final Greek version of POSAS is a comprehensive, culturally adapted tool for scar assessment from various causes. It provides a structured and standardized approach for evaluating scars, incorporating both patient-reported outcomes and clinical observations. The validation study is expected to confirm its reliability, ensuring it can be effectively used in both clinical and research settings.

Conclusions:

The Greek POSAS enables systematic scar evaluation, documentation, and comparison, supporting treatment decisions and assessing therapeutic outcomes. It is a reliable research tool for clinical and scientific applications in scar assessment before and after interventions. Its availability in Greek enhances its accessibility, contributing to improved scar management and treatment outcomes.

*O06.5 Integration of flow cytometry and single-cell RNA sequencing analysis to explore the fibroblast subpopulations in keloid that correlate with recurrence.*

**Xie, R.** ^1^, Chen, J. ^1^, Li, Z. ^1^

^1^ Department of Burn and Plastic Surgery, Sichuan University West China Hospital, Chengdu, China

Oral presentations 06—Scars 1, 4 September 2025, 08:30–10:00

Aim:

The pathogenesis of keloid (KD) remains unclear. Fibroblasts (FBs) are the cytological basis of KD formation, and are a cell population with significant heterogeneity in cell function and molecular phenotype. This study aimed to explore the influence of heterogeneous FB subpopulations on the recurrence of KD and mining key pathogenic FB subpopulations.

Methods:

Keloid fibroblasts (KFs) were comprehensively analyzed via single-cell RNA sequencing data to identify key subpopulations. Flow cytometry (FCM) was used to identify the surface molecular phenotypes of FBs that affect KD recurrence, and to explore the key pathogenic fibroblast subpopulations. Logistic regression analysis was performed, incorporating fundamental clinical information and disease remission data from the included patients to assess the predictive value of changes in FB subpopulation percentages for clinical KD recurrence.

Results:

Through clustering analysis, we obtained four subpopulations, FB1-FB4. The proportion of the FB1 subpopulation increased with KD, and functional enrichment analysis suggested that FB1 may play a greater role in extracellular matrix (ECM) collagen oversynthesis. The gene expression of CD26, CD117 and CD34 was increased significantly in FB1. In recurrent KD samples, the percentage of CD26+/CD117+/CD34+ subpopulation increased significantly, while the percentage of CD26−/CD117−/CD34− subpopulation decreased more significantly (*p* < 0.05). Regression analysis confirmed an increased risk of KD recurrence when the percentage of the CD26+/CD117+/CD34+ subpopulation increased.

Conclusions:

We identified a key pathogenic FB subpopulation that may affect KD development, which can be used as potential markers to predict recurrence and provide potential target cell populations for future clinical treatment.

*O06.6 Identify the classification pattern and immune infiltration characteristics of anoikis-related genes in keloid*

**Xie, R.** ^1^, Chen, J. ^1^, Li, Z. ^1^

^1^ Department of Burn and Plastic Surgery, Sichuan University West China Hospital, Chengdu, China

Oral presentations 06—Scars 1, 4 September 2025, 08:30–10:00

Aim:

Keloid is a pathological disease characterized by the deposition of extracellular matrix (ECM) and hyperplasia of fibroblasts. Anoikis, A kind of programmed cell death that restricts aberrant cell proliferation or the adherence of isolated cells to other substrate. However, the potential value of ARGs in keloid has not been investigated.

Methods:

We downloaded three keloid fibroblast (KF) datasets from the GEO, and obtained 338 ARGs from the GeneCards database and PubMed article. The WGCNA was used to construct the co-expression network, and obtained the KF-related ARGs. The Lasso–Cox method was used to screen the hub ARGs and construct the best prediction model. GEO single cell sequencing datasets were used to verify the expression of hub genes. Finally, we used the total RNA-sequencing technique to verify the correlation between keloid immune infiltration and anoikis.

Results:

Our study comprehensively analyzed the role of ARGs in keloid for the first time. Lasso regression analysis screened six hub ARGs (HIF1A, SEMA7A, SESN1, CASP3, LAMA3 and SIK2). A large number of miRNAs participate in the regulation of hub ARGs, 45 drugs or molecular compounds act on hub ARGs, including 4 inhibitory drugs. In addition, correlation analysis suggested that ARGs played an important role in the change of the keloid immune infiltrating state.

Conclusions:

This study provides a useful reference for revealing the role of ARGs in the pathogenesis of keloid. The hub genes we uncovered may provide potential therapeutic targets for patients. It provides new ideas for drug sensitivity screening, individualized therapy and immunotherapy.

*O06.7 Beyond z-plasty: trapeze flap plasty for post-burn contractures of the axilla and elbow*

**Yadavalli, R.**, Ganji, R.

Oral presentations 06—Scars 1, 4 September 2025, 08:30–10:00

Aim:

To evaluate the effectiveness of trapeze flap plasty in releasing post-burn contractures (PBC) of the axilla and elbow.

Methods:

A retrospective study was conducted from February 2023 to February 2024, involving 22 patients with post-burn contractures (PBC) of the elbow (*n* = 13) or axilla (*n* = 9). Patients with circumferential burns or prior joint surgery were excluded. The surgical technique followed the trapeze flap method described by Grishkevich (1). After identifying the joint’s axial line, radial incisions were marked across the contracture band, creating a central trapezoid-shaped flap that is transposed or advanced over the joint. Flaps from remaining radial incisions were approximated and sutured where possible. Residual peripheral raw areas were skin grafted. Joint angles were recorded pre- and post-operatively using a goniometer.

Results:

The mean patient age was 34.6 ± 10.4 years. Pre-operative contracture angles averaged 111.1° at the elbow and 56.1° at the axilla, improving post-operatively to 158.8° and 100.5°, respectively. Skin grafting was required in 4 (30.7%) elbow cases and 3 (33.3%) axilla cases. All patients regained functional use of the affected joint after surgery and reported good patient satisfaction. No contracture recurrence was observed during one-year follow-up.

Conclusions:

Trapeze flap plasty is a versatile and effective method for releasing axillary and elbow contractures. Its “cut-as-you-go” approach allows for tailored defect coverage using multiple flaps and grafting as needed. This technique offers a promising alternative to traditional methods like Z-plasty. Randomized control trials to compare both techniques are recommended.

*O06.8 Bromelain-based enzymatic debridement vs. standard of care in pediatric burns: long term results from a multicenter randomized controlled trial*

**Claes, K.**^1^, Shoham, Y.^2^

^1^ Ghent University Hospital, Ghent, Belgium, ^2^ Soroka University Medical Center, Ben Gurion University of the Negev, Beer Sheba, Israel

Oral presentations 06—Scars 1, 4 September 2025, 08:30–10:00

Aim:

To assess the long-term safety and efficacy of bromelain-based enzymatic debridement (BBD, NexoBrid^®^, Yavne, Israel) in pediatric patients with deep thermal burns.

Methods:

This multicenter, open-label, randomized controlled trial was conducted across 36 burn centers in Europe, the US, Israel, and India. Children (aged 0–18 years) with deep thermal burns covering up to 30% TBSA were randomized to receive either BBD or standard of care (SOC) for eschar removal. Patients were followed for over 30 months. Primary endpoints were time to complete eschar removal and percentage of wound area surgically excised. Long-term scar outcomes were assessed using the Modified Vancouver Scar Scale (MVSS) and the Patient and Observer Scar Assessment Scale (POSAS), along with safety monitoring.

Results:

Between 2015 and 2020, 145 children were enrolled (72 BBD, 73 SOC). Follow-up completion at 12, 24, and >30 months included 66/63, 58/53, and 45/37 patients (BBD/SOC), respectively. Previously reported co-primary endpoint results confirmed BBD’s superiority in time to complete eschar removal (1 vs. 6 days, *p* < 0.001) and the percentage of wound area surgically excised (1.5% vs. 48.1%, *p* < 0.001). Mean MVSS scores (BBD/SOC) at 12, 24, and >30 months were 3.8/4.9, 3.1/3.7, and 2.4/2.8, respectively. POSAS total scores (BBD/SOC) were 29.5/34.5, 22.9/27.7, and 20.5/22.8, respectively. Adverse events rates were comparable between groups through >30 months.

Conclusions:

BBD is safe and effective in pediatric burn care, significantly accelerating eschar removal, reducing excisional surgery, and achieving long-term cosmetic and functional outcomes comparable to SOC up to >30 months post-injury.

*O06.9 Modulation of the inflammatory and catabolic response after burn injury by enzymatic debridement*

Barret Nerín, J. ^1,2^, Baena, J. ^1^, Bulla, A. ^1^, Lopez Martinez, J. ^1^, Serracanta, J. ^1^

^1^ Vall d’Hebron Barcelona Hospital Campus, Barcelona, Spain, ^2^ Universitat Autònoma de Barcelona, Barcelona, Spain

Oral presentations 06—Scars 1, 4 September 2025, 08:30–10:00

Aim:

Immediate/early burn wound excision has shown to abrogate the inflammatory and catabolic response following burn injury. Enzymatic debridement with bromelain allows for early/immediate burn wound excision with excellent patient outcomes. The effect of this type of chemical excision on the inflammatory and catabolic response after burn injury is yet to be outlined. The present study aims at unraveling the effect of this wound treatment on the acute response.

Methods:

Ten consecutive patients treated with enzymatic bromelain were studied to determine the effect of this treatment on the inflammatory and catabolic response. Acute inflammatory and anabolic mediators (C3, C4, acute phase proteins, IL6, GH, IGF-1, and resting energy expenditure) were examined before treatment, 24 h after treatment and at 5 days after treatment.

Results:

TBSA burned was 28.8% ± 11 and TBSA burned treated with bromelain was 15.5 ± 2.8.

All patients exhibited a significant decrease in C3, C4, IL6, acute phase proteins, REE and a significant increase in GH and IGF-1.

Conclusions:

Enzymatic debridement with bromelain abrogates the inflammatory response and enhances anabolism by increasing levels of GH and IGF-1 following burn injury. The response follows similar patters of those observed in traditional burn wound excision.

*O07.1 Effectiveness of self-management interventions for burn survivors: a systematic review among patients with burns and two other comparable chronic conditions*

**Blok, S.** ^1,2,3^, Geelen, S. ^1,3^, Hulleman, D. ^1^, Scholten-Jaegers, S. ^1^, Nieuwenhuis, M. ^1,3,4^, Pijpe, A. ^5,6,7^, van Baar, M. ^8,9^, Bosma, E. ^1,10^, van der Sluis, C. ^2^

^1^ Alliance of Dutch Burn Care (ADBC), Burn Centre, Martini Hospital, Groningen, The Netherlands, ^2^ University of Groningen, University Medical Centre Groningen, Department of Rehabilitation Medicine, Groningen, The Netherlands, ^3^ Research group Healthy Ageing, Allied Health Care and Nursing, Hanze University of Applied Sciences, Groningen, The Netherlands, ^4^ University of Groningen, University Medical Centre Groningen, Department for Human Movement Sciences, Groningen, The Netherlands, ^5^ Alliance of Dutch Burn Care (ADBC), Burn Centre, Red Cross Hospital, Beverwijk, The Netherlands, ^6^ Amsterdam UMC location Vrije Universiteit Amsterdam, Department of Plastic, Reconstructive and Hand Surgery, Amsterdam, The Netherlands, ^7^ Amsterdam Movement Sciences (AMS), Tissue Function and Regeneration, Amsterdam, The Netherlands, ^8^ Alliance of Dutch Burn Care (ADBC), Burn Centre, Maasstad Hospital, Rotterdam, The Netherlands, ^9^ Erasmus MC, University Medical Centre Rotterdam, Department of Public Health, Rotterdam, The Netherlands, ^10^ Martini Hospital, Department of Surgery, Groningen, The Netherlands

Oral presentations 07—Rehabilitation 1, 4 September 2025, 13:45–15:15

Aim:

To describe and to report on the effectiveness of self-management interventions (SMI) in burn survivors and in two other patient groups with comparable chronic conditions.

Methods:

We conducted and reported this review according to the PRISMA guidelines. We searched the MEDLINE, CINAHL, Cochrane, EMBASE, and PsycINFO databases between January 2010 and August 2024. Eligible studies were those evaluating the effectiveness of SMI’s intended for burn survivors or for two other comparable conditions, i.e., myocardial infarction (MI) and lower extremity trauma (LET). The Downs and Black checklist was used for quality assessment.

Results:

The search yielded 6635 results, of which 16 studies met the inclusion criteria. One study targeted burn survivors, but scored poor on methodological quality. Furthermore, 8 studies on MI and 7 studies on LET were identified. There was considerable variation in intervention components, procedures, terminology, evaluated constructs, and methodological quality was found. Patients with MI demonstrated significant improvements in self-management skills one to 6 months post-intervention, and patients with LET showed a tendency towards self-management skills improvement.

Conclusions:

In conclusion, there is a lack of SMI’s that are specifically designed for burn survivors. Hence, drawing firm conclusions about the effectiveness of SMI’s in burn survivors is not possible. However, studies on other comparable chronic conditions show indications that SMI’s do improve self-management skills. Nevertheless, comparability between studies was limited due to heterogeneity in methodology. Future research should firstly focus on establishing consensus in terminology and methodology and secondly, explore the most effective SMI components in burn survivors.

*O07.2 Women’s experiences of health and well-being following hospital burn care. a scoping review*

Daltveit, S. ^1^, Almeland, S. ^2^, Beisland, E. ^1^, Mortensen, M. ^1^, **Moi, A.** ^1,3^

^1^ Western Norway University of Applied Sciences, Bergen, Norway, ^2^ Department of Plastic, Hand and Reconstructive Surgery, National Burn Centre, Haukeland University Hospital, Bergen, Norway, ^3^ Faculty of Health Sciences, VID Specialized University, Bergen, Norway

Oral presentations 07—Rehabilitation 1, 4 September 2025, 13:45–15:15

Title:

Women’s experiences of health and well-being following hospital burn care. A scoping review

Aim:

To map and describe the literature on women’s experiences of health and well-being following hospital burn care.

Methods:

A scoping review has been conducted to identify qualitative research reporting on the subjective experiences of health and well-being in burn-injured women aged ≥18 years, who report on their experience of life after discharge from a burn unit. Study selection was performed double-blinded by two pairs of reviewers. Results will be thematically and narratively summarised.

Results:

In total, 9292 scientific papers were identified in our database search, of which 50 studies were eligible according to the inclusion criteria. Of the included studies, only 12 reported findings from women exclusively. The remaining 38 studies reported quotes from interviews with both men and women, but the data were analysed and overall findings presented without taking gender into consideration. Our preliminary analysis indicates that women experience unique gender-specific challenges during the post-discharge rehabilitation period.

Conclusions:

There are few qualitative studies on women’s health and well-being after hospital burn care. Most studies report their findings without considering gender. More qualitative research is recommended to understand the unique experiences of women’s health and well-being in the aftermath of major burns.

*O07.3 Preliminary evaluation of a physical training-based rehabilitation program in severely burned patients: pilot study*

**Dávalos Yerovi, V.** ^1^, Campusano Garate, F. ^1^, Pujol Blaya, V. ^1^, Donat, I. ^1^, Sabate López, M. ^1^, Gómez Garrido, A. ^1^, Torrent, M. ^1^

^1^ Vall D’Hebron Hospital, Barcelona, Spain

Oral presentations 07—Rehabilitation 1, 4 September 2025, 13:45–15:15

Aim:

To assess clinically significant changes in aerobic exercise capacity, peripheral and respiratory muscle strength, and physical performance in severely burned patients after completing a physical training (PT) based rehabilitation program.

Methods:

Prospective pilot study, conducted from October 2023 to March 2025 in a Rehabilitation Unit. Included adults aged 18 to 65 with burns covering at least 20% of the total body surface area (TBSA). The variables assessed included aerobic exercise capacity through a cardiopulmonary exercise test, such as peak oxygen consumption (VO_2_ peak), and the first ventilatory threshold (VT1) and workload measured in watts (W); peripheral muscle strength, measured using a handgrip dynamometer; respiratory muscle strength, assessed through maximum inspiratory and expiratory pressures (MIP and MEP); and overall physical performance, evaluated using the 1-Minute Sit-to-Stand Test (1MinSTST).

Results:

Eight patients completed the PT program (75% male, mean age of 48 years). Following the program, significant improvements were observed in exercise capacity, with an increase in VO_2_ peak (69.1% to 78.3%, *p* = 0.06) and the first ventilatory threshold (VT1) (35.6% to 43.9%, *p* < 0.05). Muscle strength also showed a significant increase, rising from 25 kg to 29.7 kg (*p* < 0.05). Marginally significant improvements were recorded in the 1MinSTST, with scores increasing from 60.2% to 78.2% (*p* = 0.06). Additionally, improvements were observed in both MIP and MEP, though these changes were not statistically significant.

Conclusions:

The rehabilitation program based on physical training has proven to be effective and safe, significantly improving muscle strength and exercise capacity. Further studies are needed to confirm these findings and assess their long-term impact.

*O07.4 AVATAR: Unique combination of virtual reality and kinaesthetic illusions in rehabilitation of burn victims*

**Diakonu, A.** ^1^, Zajicek, R. ^1,2^, Bakalar, B. ^1,2^, Zimermanova, H. ^1^

^1^ Third Faculty of Medicine, Charles University, Prague, Czech Republic, ^2^ Prague Burn Centre, Faculty Hospital Kralovske Vinohrady, Prague, Czech Republic

Oral presentations 07—Rehabilitation 1, 4 September 2025, 13:45–15:15

Aim:

The Avatar project uses a combination of virtual reality (VR) and kinaesthetic illusion to provide an innovative approach to the early rehabilitation of burn victims. The combination of immersive VR and vibrotactile feedback by inducing a kinaesthetic illusion enables the perception of motion and provides non-invasive, painless muscle rehabilitation through brain activation for burn patients.

Methods:

The project was divided into three phases.

The first phase focused on developing a synchronisation of VR and vibrotactile stimulation that could be applied to the lower limbs, even in the presence of bandages and burns.

The movement represented by the illusion is a peaceful walk through a friendly landscape.

In the second phase, the system was tested on 32 healthy volunteers using a questionnaire survey to determine further configurations and the rate of immersion. In addition, the effects of the system on body and mind were assessed using neurophysiological methods and monitoring of vital functions and metabolic response using indirect calorimetry.

A clinical trial is being considered as the third phase.

Results:

Avatar was able to induce the illusion of walking in all volunteers tested, with no effect on vital signs and an increased preference for fat as a fuel source.

Conclusions:

The AVATAR project has the potential to be a unique and novel approach to the rehabilitation of patients with severe burns.

This study was supported by the Technology Agency of the Czech Republic under grant number FW06010562.

*O07.5 Examining how burn care professionals deliver self-management support to burn survivors after discharge from the burn centre: a qualitative, grounded theory study*

**Geelen, S.** ^1,2^, Niemeijer, A. ^1^, Scholten-Jaegers, S. ^1^, van Uden, D. ^3^, Verwilligen, R. ^4,5,6^, Bosma, E. ^1,7^, Nieuwenhuis, M. ^1,2,8^

^1^ Alliance of Dutch Burn Care (ADBC), Burn Centre, Martini Hospital, Groningen, The Netherlands, ^2^ Hanze University of Applied Sciences, Research group Healthy Ageing, Allied Health Care and Nursing, Groningen, The Netherlands, ^3^ Alliance of Dutch Burn Care (ADBC), Burn Centre, Maasstad Hospital, Rotterdam, Rotterdam, The Netherlands, ^4^ Alliance of Dutch Burn Care (ADBC), Burn Centre, Red Cross Hospital, Beverwijk, The Netherlands, ^5^ Amsterdam UMC location VUmc, Department of Plastic, Reconstructive and Hand Surgery, Amsterdam, The Netherlands, ^6^ Amsterdam Movement Sciences (AMS), Tissue Function and Regeneration, Amsterdam UMC, Amsterdam, The Netherlands, ^7^ Martini Hospital, Department of Surgery, Groningen, The Netherlands, ^8^ University of Groningen, University Medical Centre Groningen, Department for Human Movement Sciences, Groningen, The Netherlands.

Oral presentations 07—Rehabilitation 1, 4 September 2025, 13:45–15:15

Aim:

To examine how healthcare professionals in burn aftercare deliver self-management support to burn survivors.

Methods:

Using an inductive, interpretive qualitative methodology, interviews were conducted with 16 healthcare professionals from three specialized burn centres in The Netherlands. Participants included six burn physicians, three burn nurse specialists, three burn aftercare nurses, two occupational therapists, and two medical psychologists. Data analysis was carried out using a constant comparative method to develop a middle-range theoretical model.

Results:

The study highlights considerable variation in how healthcare professionals deliver self-management support. The middle-range theoretical model suggests that these variations are influenced by how professionals balance roles and responsibilities with burn survivors and within the multidisciplinary team. This balancing is shaped by three key factors: (1) difficulties in recognizing the support needs of burn survivors after discharge, (2) challenges in defining and managing the often complex boundaries of their professional responsibilities, and (3) issues arising from unclear team roles, conflicting goals, and time constraints.

Conclusions:

There are significant differences in how healthcare professionals approach self-management support, largely driven by their perceptions of their roles and responsibilities. Enhancing their capacity to support burn survivors may require targeted training in communication, use of comprehensive needs assessment tools, clearer role definitions within multidisciplinary teams, and improved strategies for managing shared responsibilities with patients. Furthermore, organizational adjustments—such as aligning care with burn survivors’ needs and ensuring adequate time for professional-patient interactions—are crucial to improve self-management support delivery.

*O07.6 Effect on gait pattern during robot assisted gait training (RAGT) of end-effector type in burn patients*

**Joo, S.** ^1^, Won, Y. ^2^, Lee, S. ^3^

^1^ Hallym University Hangang Sacred Heart Hospital, Seoul, Republic of Korea, ^2^ Jeonbuk National University Medical School and Research Institute of Clinical Medicine of Jeonbuk National University, Jeonju, Republic of Korea, ^3^ Soonchunhyang University Hospital, Bucheon, Bucheon, Republic of Korea

Oral presentations 07—Rehabilitation 1, 4 September 2025, 13:45–15:15

Aim:

This study aimed to investigate gait pattern and muscles power improvement of patient with gait disturbance caused by burns after end-effector type robot (Morning Walk^®^, Curexo, Seul, Republic of Korea)-assisted gait training (RAGT).

Mehtods:

This study randomly assigned 25 patients to one of two group: 30 min of Morning Walk^®^ training with 30 min conventional physiotherapy (RAGT group) or 60 min of conventional physiotherapy (CON group). 5 training sessions per week were given for 8 weeks. The primary outcomes were gait performance and muscles powers, which were assessed by the functional ambulation category (FAC) and the manual muscles test (MMT), respectively. The secondary outcomes included 6-min walking test (6 MWT), gait kinematic and spatiotemporal gait parameters.

Results:

At baseline, there was no statistically significant difference between the two groups. After 8 weeks training, the RAGT group showed significant improvement in FAC, MMT, 6 MWT, and kinematic parameters. The control group showed significant improvement in the FAC, MMT, and 6 MWT. Inter-group comparison demonstrated that the FAC, MMT of ankle and knee, gait kinematic parameters (bilateral step length, peak knee extension at single support, and peak knee flexion at swing) of the RAGT group were significantly improved than those of the control group.

Conclusions:

It was confirmed that the ROM improved in the static state as well as in the dynamic state. The results of this study suggest that the patients with gait disturbance receiving the RAGT might improve greater in gait performance and normal gait patterns that those trained with conventional physiotherapy.

*O07.7 Lymphedema in burns—incidence and influence on clinical outcomes. Assessment with real-world data*

**Kleinhapl, J.** ^1,2^, Dempsey, J. ^1^, Golovko, G. ^3^, Gainski, L. ^1^, Ekale, J. ^1^, McAuley, R. ^1^, Branski, L. ^1,4^, Finnerty, C. ^1^, Wolf, S. ^1^

^1^ Department of Surgery, University of Texas Medical Branch, Galveston, United States, ^2^ Division of Plastic, Aesthetic and Reconstructive Surgery, Department of Surgery, Medical University of Graz, Graz, Austria, ^3^ Department of Pharmacology and Toxicology, University of Texas Medical Branch, Galveston, United States, ^4^ Shriners Children’s Texas, Galveston, United States

Oral presentations 07—Rehabilitation 1, 4 September 2025, 13:45–15:15

Aim:

To investigate the incidence of lymphedema in burn patients using a real-world database. Further, to assess the influence of post-burn lymphedema on clinical outcomes.

Methods:

We conducted a study in the real-world database TriNetX. Patients were stratified into cohorts based on the affected total body surface area (TBSA): Cohort 1 (<20% TBSA burned), Cohort 2 (20–39% TBSA burned), Cohort 3 (>39% TBSA burned). We analyzed the incidence of lymphedema, treatment modalities, and the influence of lymphedema on clinical outcomes within 1 year after burn. Clinical outcomes were analyzed using TriNetX software, with significance set at *p* < 0.05.

Results:

We identified 2603 patients with burns and lymphedema diagnosis. Mean age was 65 ± 16 years. The population was 57.9% female and 37.2% male (4.9% unknown). The incidence of lymphedema was increasing with burn size, showing 0.30%, 0.49% and 0.85% in cohort 1,2, and 3, respectively. Patients with lymphedema after burn had a significantly higher risk for mortality, wound healing disorders, skin infections, thrombosis, cellulitis/lymphangitis (all *p* < 0.0001), scar pain (*p* = 0.0007) and psychological outcomes (*p* = 0.0152). The use of compression therapy or lymphatic drainage was identified in 1.53% of all patients with burns and lymphedema.

Conclusions:

Lymphedema incidence in burns increases with burn size and is associated with higher risks for clinical complications when compared to burn patients without lymphedema. The small number of therapeutic interventions indicates a potential undertreatment, underreporting of treatment, or missing coding for it. Hence, our results highlight the need for further research on this specialized topic of burn care.

*O07.8 Long-Term Evaluation of a Novel Burn Rehabilitation Concept Based on the International Classification of Functioning, Disability and Health (ICF)*

**Klimitz, F.** ^1,2^, Ritter, E. ^1^, Neubauer, H. ^1^, Stolle, A. ^1^, Hundeshagen, G. ^1^, Kneser, U. ^1^, Harhaus, L. ^3^

^1^ Department of Hand, Plastic and Reconstructive Surgery, Burn Center, BG Trauma Center Ludwigshafen, Plastic- and Hand Surgery, University of Heidelberg, Ludwigshafen, Germany, ^2^ Division of Plastic Surgery, Department of Surgery, Yale School of Medicine, New Haven, United States, ^3^ Department of Hand, Replantation, and Microsurgery, BG Trauma Center Berlin, Chair of Hand, Replantation, and Microsurgery, Charité—Universitätsmedizin Berlin, Berlin, Germany

Oral presentations 07—Rehabilitation 1, 4 September 2025, 13:45–15:15

Aim:

To evaluate the long-term outcomes of a novel ICF-based multimodal rehabilitation concept for patients with thermal injuries.

Methods:

This prospective observational study included 38 patients with thermal injuries treated at the Burn Center of BG Trauma Center Ludwigshafen as part of the project “Evaluation of an ICF-based Rehabilitation Concept for Thermal Injuries.” Patients were assessed at least three years after discharge from inpatient rehabilitation. Objective and subjective assessments aligned with the International Classification of Functioning, Disability and Health (ICF) were used to evaluate physical, psychological, and social outcomes. Physical function was assessed using grip strength (JAMAR dynamometer and pinch meter), mobility and balance (Timed Up & Go Test), physical performance (age-adjusted PWC tests on a bicycle ergometer), and range of motion (goniometer measurements). Scar quality was analyzed using the Vancouver Scar Scale and Cutometer MPA 580, Courage+Khazaka, Koln, Germany. Patient-reported outcomes were captured using validated questionnaires (DASH, LEFS, BSHS-B, SF-36, SCL-K-9, IES-R, F-SozU).

Results:

Long-term assessments demonstrated sustained improvements in grip strength, mobility, physical capacity, and joint mobility. Scar elasticity and appearance showed favorable long-term development. Patient-reported outcomes reflected stable or improved functional and psychosocial recovery compared to post-rehabilitation values.

Conclusions:

Our findings support the long-term effectiveness of an ICF-based multimodal rehabilitation approach for burn patients. The results highlight the importance of comprehensive rehabilitation strategies in achieving sustainable recovery and advocate for broader implementation in burn care.

*O07.9 Recovery of physical activity of paediatric burn patients up to 6 month post discharge*

Akkerman, M., Mouton, L., Gerrits, K., van Baar, M., Stoop, M., Scholten-Jaegers, S., Niemeijer, A., **Nieuwenhuis, M.** ^1^

^1^ Alliance Of Dutch Burn Care, Martini Hospital, Groningen, The Netherlands, ^2^ University of Groningen, University Medical Centre Groningen, Department for Human Movement Sciences, Groningen, The Netherlands, ^3^ Hanze University of Applied Sciences, Research group Healthy Ageing, Allied Health Care and Nursing, Groningen, The Netherlands

Oral presentations 07—Rehabilitation 1, 4 September 2025, 13:45–15:15

Aim To determine the recovery of physical activity (PA) in paediatric burn patients, up to 6 months after hospital discharge, and its relationship with burn severity.

Methods:

Eligible for this prospective multicentre cohort study were patients 6–18 years of age, hospitalized in one of three Dutch burn centres with burns affecting ≥ 5% of their total body surface area (TBSA), or a length of stay ≥ 2 weeks, or both. Self-reported PA was assessed with the ‘Standard Questionnaire for Activity’ with questions regarding compliance with PA guidelines added. These assessments were done at discharge, with a recall from the week before the injury, and at 6 weeks, 3 and 6 months post-discharge. Objectively monitored PA was assessed using ActiGraph accelerometers during the first week after discharge and at 3 months.

Results:

Twenty-four participants with 0.1–34% TBSA burned were included. Based on self-report, most patients (70%) did not reach the level of PA they had prior to injury. Objective activity monitoring showed that 3 months after discharge, the mean time spent in moderate-to-vigorous activity did approach 60 min per day as recommended by the PA guidelines. The self-reported PA levels at 6 weeks after hospital discharge were negatively correlated with%TBSA (*r* = −0.807, *p* < 0.001), accounting for 65% of the explained variance.

Conclusions:

Most children did not fully recover to their pre-injury PA levels. Given the importance of physical activity, it should be monitored in this vulnerable population, especially in the early phase of rehabilitation and in those with more severe burns.

*O08.1 Post-nexobrid^®^ wound bed management through a new galenic preparation based on vaseline and glucose: clinical, histological, and microbiological evaluations*

Di Gioia, G. ^1^, Tagarelli, C. ^1^, Di Turi, C. ^1^, De Cosmo, A. ^1^, Tedeschi, P. ^1^, Maggio, G. ^1^

^1^ Azienda Ospedaliero-Universitaria Policlinico Di Bari, Bari, Italy

Oral presentations 08—Enzymatic debridement, 4 September 2025, 13:45–15:15

Aim:

Identification of an effective therapeutic protocol post-Nexobrid: comparison of silver sulfadiazine and a vaseline and glucose-based galenical preparation.

Methods:

Between March 2024 and March 2025, a group of 10 patients with medium-deep and deep burns with symmetrical locations underwent selective enzymatic escarolysis within 24 h of trauma. All right-sided burns were treated with vaseline and glucose as the “study group”; all left-sided burns were treated with silver sulfadiazine as the “control group”.

Clinical, histological and microbiological parameters were evaluated to assess treatment efficacy.

Dressing changes were made every 3 days in the study areas and daily in the control areas, with photographic documentation after 6 days.

Biopsies were taken on day 6.

Culture swabs were taken immediately after enzymatic escarolysis and after 6 days.

Results:

Analysis of the data collected showed the absence of necrosis and the presence of vital tissue in the wound bed in the study group compared to the control group, which had pseudoeschar and histologically more significant residual thermal necrosis with less presence of vital epithelial elements; finally, the absence of infection was noted in all samples, both study and control.

Conclusions:

The combination of vaseline and glucose has proven to be effective in preventing bacterial contamination of wounds, creating an environment conducive to wound healing.

The galenic formulation showed other advantages, including: low cost, ease of use, better ability to control exudate and odor, and extended dressing cadence with benefits to the patient in terms of comfort and compliance.

*O08.2 Enzymatic debridement for electric arc flash burn in pediatric patients: a promising therapeutic option*

**Tatar, R.** ^1,2^, Enescu, D. ^1,2^, Alexandru, R. ^2^, Stoica, C. ^2^, Prisecaru, B. ^2^, Nacea, I. ^1,2^

^1^ Carol Davila University of Medicine and Pharmacy, Bucharest, Romania, ^2^ Plastic Reconstructive Surgery and Burns Department, Grigore Alexandrescu Emergency Hospital for Children, Bucharest, Romania

Oral presentations 08—Enzymatic debridement, 4 September 2025, 13:45–15:15

Aim:

Recently accepted by international medical authorities for use in pediatric population, enzymatic debridement has well established indications for deep burns in adult patients. Our department also included this novel therapeutic option, and we aim to present our initial experience with enzymatic debridement in children.

Methods:

Nexobrid™, Yavne, Israel, was available in our hospital in the first half of 2024. We included in this report all patients in whom we used enzymatic debridement from that point on, until February 2025. Demographic data and information about burn cause and extent, and the final outcome, were also recorded.

Results:

We applied enzymatic debridement in four cases of severe pediatric burns, involving 35% to 60% TBSA, two girls and two boys. We addressed the upper limb in tree cases (approximately 5% TBSA), and the anterior trunck (about 10% TBSA) in forth case. Nexobrid™ was applied within 24–48 h post-burn. The burns were caused evenly by flame and electric arc flash by train climbing (the boys). The latter is an off-label use, but from the local aspect we considered it an appropriate option, and the patients healed without grafting in those areas.

Conclusions:

Our initial experience with enzymatic debridement confirmed its safety and efficacy profile for pediatric burns. Moreover, for our patients, it was useful in promoting secondary epithelialization without grafting in case of burns caused by electric arc flash after train climbing, with no local or general complication. Further studies are needed for supporting this potential new indication.

*O08.3 Bromelain based enzymatic debridement for conflict areas burn and blast injuries*

**Bloom, G.** ^1^, Sabag, N. ^1^, Shoham, Y. ^1^, Harats, M. ^2^, Haik, J. ^2^

^1^ Soroka University Medical Center, Beer Sheba, Israel, ^2^ Sheba Medical Center, Tel Hashomer, Israel

Oral presentations 08—Enzymatic debridement, 4 September 2025, 13:45–15:15

Aim:

In conflict areas and mass casualty situations limited resources restrict the availability of operating rooms (OR), prompting the need for solutions to relieve surgical bottlenecks. The aim of this study is to assess the outcomes of Bromelain Based Debridement (BBD) with Nexobrid in conflict area related mass casualty incidents (MCI).

Methods:

An ongoing multicenter retrospective study, including 2 major medical centers in Israel. Eligible participants include burn and blast patients who underwent BBD from 7 October 2023 to the present date. Data collected includes patient demographics, injury characteristics, timing and setting of enzymatic debridement, anesthesia type, additional surgical interventions, and rehabilitation details.

Results:

To this date 75 patients have been included. The average age is 29.4 ± 21 years (range 0.8–75 years) and 33.3% of patients are female, 66.6% male. The majority of patients suffered flame burns covering up to 20% TBSA (53/75 patients), however the burns area ranged up to 70% TBSA. Upper extremities were the most commonly treated area.

None of the BBD treatments necessitated the use an OR. All BBD treatments were performed either in burn intensive care or plastic surgery departments. Only 41% of the patients (31/75) necessitated surgical intervention following BBD, while 59% (44/75) healed spontaneously.

Conclusions:

The use of BBD assisted in alleviating surgical bottlenecks in conflict area MCIs, allowing ORs to be used for other multitrauma patients. Its use offers advantages such as feasible application under regional anesthesia without the need for an OR, and reducing the need for surgery after debridement.

*O08.4 Long-Term Scar Outcomes After Enzymatic Debridement with NexoBrid vs. Standard of Care: A Post-Hoc Analysis of Pooled DETECT and CIDS Data*

**Schiefer, J.** ^1^, Goverman, J. ^2^, Haviv, A. ^3^, Shoham, Y. ^4^

^1^ Department of Plastic Surgery, Hand Surgery, Burn Center University of Witten/Herdecke, Cologne-Merheim Medical Center, Cologne, Germany, ^2^ Massachusetts General Hospital Burn Center, Bostonq, USA, ^3^ MediWound Ltd., Yavne, Israel, ^4^ Soroka University Medical Center, Ben Gurion University of the Negev, Beer Sheba, Israel

Oral presentations 08—Enzymatic debridement, 4 September 2025, 13:45–15:15

Aim:

Clinical trials demonstrate that bromelain-based enzymatic debridement (NexoBrid^®^) is non-inferior to standard care (SOC) in long-term scar outcomes. This analysis evaluates NexoBrid’s scar outcomes using pooled data from completed phase III studies in adult (DETECT) and pediatric (CIDS) burns.

Methods:

Pooled data from DETECT and CIDS were analyzed for total and domain scores of the Modified Vancouver Scar Scale (MVSS) and the Patient and Observer Scar Assessment Scale (POSAS) at 12 months (12M) by target wound. Scar outcomes were compared by autograft use (Y/N) for deep partial thickness (DPT) wounds. A One-way ANOVA test was applied. Additionally, MVSS outcomes from the single-arm NEXT Expanded Access Protocol with BBD in the US were compared.

Results:

160 (NexoBrid) and 140 (SOC) wounds were assessed for MVSS or POSAS at 12M, including 82 (NexoBrid) and 64 (SOC) non-autografted DPT wounds and 24 and 26 autografted (≈25% of DPT wounds). Overall, NexoBrid showed superior MVSS and POSAS scores vs. SOC (MVSS: 3.9 vs. 4.6, *p* = 0.030; POSAS: 33.9 vs. 39.9, *p* = 0.009), with the difference primarily stemming from non-grafted DPT wounds (MVSS: 3.3 vs. 4.3, *p* = 0.033; POSAS: 27.9 vs. 36.7, *p* = 0.030). MVSS and POSAS scores were worse for grafted vs. non-grafted DPT wounds for both NexoBrid and SOC. Higher incidence of autografting DPT in NEXT (48.9%) resulted in worse MVSS scores (4.6 adults, 5.6 pediatrics). Domain scores indicated less hypertrophic scarring and improved pigmentation, pliability, and surface regularity with NexoBrid vs. SOC.

Conclusions:

NexoBrid provides superior outcomes to SOC at 12M, particularly in non-grafted DPT wounds.

*O08.5 Enzymatic debridement in pediatric burn patients: nursing care and post-debridement wound coverage strategies*

**Gonzalez Gilarte, L.** ^1^, Miguel Puigbarraca, P. ^1^, Barroso Sala, M. ^1^, Roca Pascual, D. ^1^

^1^ Hospital Universitario Vall D’hebron, Barcelona, Spain

Oral presentations 08—Enzymatic debridement, 4 September 2025, 13:45–15:15

Title: Enzymatic debridement in pediatric burn patients: nursing care and post-debridement wound coverage strategies

Aim:

To describe the experience with enzymatic debridement in pediatric burn patients and to highlight the role of nursing care.

Methods:

Retrospective observational study of admitted patients. Pediatric patients with intermediate-deep burns who met clinical criteria for enzymatic debridement were included. Continuous variables were expressed as median (range), and categorical variables as number and/or percentage.

Results:

A total of 34 patients were included. Median age: 4 years (0.7–17); total body surface area burned (TBSA): 10% (1–60). In 31 patients (91%), a single enzymatic debridement was performed, with a treated TBSA ranging from 1% to 36%. The most frequent burn mechanism was scalding (50%), followed by flame burns (44%). In 20 patients (59%), debridement was performed within the first 24 h after injury. The main indication was circumferential involvement (84% vs. 26.7%). Of the 19 patients with circumferential burns, 2 (10%) required surgical escharotomy in addition. Post-debridement nursing care focused on continuous wound assessment, pain control, infection prevention, and the application of appropriate dressings based on wound characteristics. Nursing care was standardized according to the clinical evolution of the burns.

Conclusions:

Enzymatic debridement in pediatric patients is a safe and effective technique when performed in a specialized setting. The nursing role is essential for post-procedural care, including wound monitoring, pain management, and the appropriate selection of dressings.

*O08.6 Functional evaluation following enzymatic escarolysis and scar treatment outcomes: results from a single centre case-control study*

Dalla Costa, D. ^1^, Sgabussi, P. ^2^, Morello, V. ^2^, **Citterio, A.** ^2^

^1^ Rehabilitation Unit—ASST Grande Ospedale Metropolitano Niguarda, Milan, Italy, ^2^ Burn Centre—ASST Grande Ospedale Metropolitano Niguarda, Milan, Italy

Oral presentations 08—Enzymatic debridement, 4 September 2025, 13:45–15:15

Aim:

The study analyzes the efficacy of non-invasive treatment with pharmacological (Sameplast^©^ gel) and physical (silicone plaster) effects for the prevention and treatment of scarring hypertrophy in burn patients.

Methods:

This is an observational, retrospective, two-arm case-control study. Twenty patients were treated in the acute phase with enzymatic escarolysis within 72 h of the burn event. Forty days following scar healing, treatments targeting hypertrophic scar areas were initiated and maintained for 6 months.

Subjects were divided into 2 groups (*n* = 10): A, the protocol (gel applied daily and plaster nightly), and B, the control (emollient moisturizer). Pressure garments were used constantly.

Follow-ups were at 3, 6, and 12 months to assess the scar characteristics, using Vancouver Scar Scale (VSS), The Patient and Observer Scar Assessment Scale (POSAS), and functional outcomes with Burn Specific Health Scale—Brief version (BSHS-B).

Results:

At 6 and 12 months, scar progression and Quality of Life showed greater improvement for group A. Group A and B mean values, respectively, were VSS 2.6 vs 3.1; POSAS patient 3.5 vs 3.9; POSAS observer 3.6 vs 3.8; and BSHS-B 35.8 vs 33.0. No significant differences were observed between groups in VSS, POSAS observer and patient, and BSHS-B, Tukey–Duckworth test (*p* > 0.01).

Conclusions:

Limitations include small study groups, and time-sensitive for protocol implementation. However, the combined use of Sameplast^©^ gel and plaster evidences overall improvement in scar quality, additionally, reducing functional limitations. This could be considered a surgical-rehabilitative approach to be included in scar treatment protocols.

*O08.7 Comparing the Effectiveness of Single-Enzyme Slow Debridement Alternated with Hyaluronic Acid-Enriched Silver Sulfadiazine vs. Dual-Enzyme Slow Debridement Alone in Second-Degree B Burn Healing: A Randomized Controlled Trial*

Moraru, D. ^1,3^, Bulgaru Iliescu, A. ^2,3^, Băetu, M. ^3^, Avadani, B. ^3^, Amarandei, A. ^3^, Perțea, M. ^1,3^

^1^ ”Grigore T. Popa” University of Medicine And Pharmacy Iasi, Romania, Department of Plastic Surgery, Iasi, Romania, ^2^ ”Grigore T. Popa” University of Medicine and Pharmacy Iasi, Romania, Department of Anatomy, Iasi, Romania, ^3^ ”Sf. Spirion” Emergency Hospital, Iasi, Romania, Plastic Surgery and Burns Clinic, Iasi, Romania

Oral presentations 08—Enzymatic debridement, 4 September 2025, 13:45–15:15

Aim:

This study evaluates the effectiveness of single-enzyme (collagenase) slow debridement alternated with hyaluronic acid-enriched silver sulfadiazine vs. dual-enzyme slow debridement (collagenase + protease) alone in promoting healing and minimizing scarring in low TBSA second-degree B burns.

Methods:

In a randomized controlled trial, 80 patients with second-degree B burns (<10% TBSA) were randomized into two groups: Group A (single-enzyme debridement alternated with hyaluronic acid-enriched silver sulfadiazine) and Group B (dual-enzyme debridement alone). For Group A, debridement using an agent containing collagenase derived from Vibrio alginolyticus was performed for 14–21 days, alternating every two days with the application of hyaluronic acid-enriched silver sulfadiazine. Group B underwent slow debridement with a two-enzyme agent alone for 14–21 days, followerd by 10–21 days of panthenol based topicals. Silicone gel sheets were applied for both groups during the post-healing phase. The study measured re-epithelialization time, wound size reduction, pain levels, infection rates, and scarring at 3 and 6 months, using the Vancouver Scar Scale (VSS).

Results:

Preliminary findings suggest that dual-enzyme debridement alone resulted in faster debridement and re-epithelialization compared to single-enzyme debridement. However, Group A (single-enzyme + hyaluronic acid-enriched silver sulfadiazine) showed better scar pliability, pigmentation, and patient satisfaction at 6 months. Infection rates and pain levels were comparable across both groups.

Conclusions:

Single-enzyme debridement alternating with hyaluronic acid-enriched silver sulfadiazine improves long-term scar outcomes in second-degree B burns, with superior cosmetic and functional results compared to dual-enzyme debridement alone. Further studies are needed with larger sample sizes to confirm these findings.

*O08.8 Paradigm Change in Burn Care: The 42-Year NexoBrid Saga*

**Rosenberg, L.** ^1^

^1^ Mediwound, Yavne, Israel, ^2^ Ben Gurion University, Soroka University Medical Center, Beer Sheva, Israel

Oral presentations 08—Enzymatic debridement, 4 September 2025, 13:45–15:15

Aim:

It is the four-decade long development and implementation chronicles of a novel enzymatic debridement agent, the base of a paradigm shift in burn care.

Methods:

This historical account of the research and development processes of the bromelain based enzymatic debriding agent (BBDEA) started by defining the needs of burn debridement and surgical eschar excision in 1943 and realization of its limitations and costs years later. The BBDEA development started in 1982 with numerous preclinical porcine model studies. Following the assimilation of the data gained in these studies, human clinical trials started in 1985 with subsequent 11 large-scale, controlled studies in >1334 adult and children burn patients, leading to the regulatory approval of the BBDEA in Europe (2014) and the US (2023). BBDEA’s impact on over 15,000 burn patients was assessed through clinical outcomes and over 150 peer-reviewed publications.

Results:

BBDEA proved to be effective, completing a selective burn eschar removal and escharotomy in 1 day, (4-h application vs. 4–6 days in standard-of-care SOC). It reduces blood loss (14 cc vs. >814 cc), reduces surgery (4% vs. 72%) and skin grafting (9% vs. 20%). Time to wound closure and final outcome (cosmesis and function) were at least as good if not better than SOC.

Conclusions:

Nonsurgical safe, effective, selective and cost-effective enzymatic debridement is the base of the minimal invasive paradigm change of burn care of the individual burn victim and in Burn Mass Casualties Incidents.

*O08.9 Enzymatic debridement with Nexobrid^®^: protocol update for acute kidney injury prevention*

**García García, M.** ^1^, Bayo, M. ^1^, Guijarro, C. ^1^, Merino, M. ^1^, Pérez, M. ^1^, Ferre, M. ^1^, Vidal, A. ^1^

^1^ Hospital Universitario y Politécnico La Fe, Valencia, Valencia, Spain

Oral presentations 08—Enzymatic debridement, 4 September 2025, 13:45–15:15

Aim:

We present an updated protocol for the use of Nexobrid^®^ in the treatment of deep burns, aimed at preventing the risk of acute kidney injury (AKI), an important complication associated with enzymatic debridement in major burn patients.

Methods:

This protocol begins with an initial evaluation of the burns to determine the indication for treatment. A wet dressing is applied, covering no more than 15% of the total body surface area (TBSA) to prevent hypothermia. Within 24–48 h of admission, severity is assessed using SOFA scale and blood tests, including NephroCheck^®^. Then, there are three options:

(a) If SOFA score is below 6 or TIMP-2/IGFBP7 is below 0.3, enzymatic debridement is performed under prophylactic antibiotics and fluid resuscitation;
(b) If SOFA scores is above 6, reassessment occurs within 12–24 h; and
(c) If TIMP-2/IGFBP7 is above 0.3, the patient is considered at high-risk of kidney injury and require fluid adjustments and nephrotoxic withdrawal before reevaluation.

Results:

Previous studies indicate that Nexobrid^®^ reduces surgical debridement requirements by 30–40%, with comparable wound healing rates. Biomarkers like TIMP-2 (Tissue Inhibitor of Metalloproteinases-2) and IGFBP7 (Insulin-like Growth Factor Binding Protein 7) have shown strong predictive value for AKI, particularly in critically ill patients, supporting their integration into this protocol.

Conclusions:

This protocol highlights the need for assessment of renal function in patients with deep burns prior to enzymatic debridement with Nexobrid^®^. By incorporating NephroCheck^®^, a biomarker-based risk evaluation tool, the process ensures effective treatment while minimizing potential risks.

*O09.1 1-year phase II clinical study results of a bio-engineered, autologous dermo-epidermal skin graft in burn patients*

**Böttcher, S.** ^1^, Schiestl, C. ^1^, Meuli, M. ^2^, Hartmann-Fritsch, F. ^2^, Middelkoop, E. ^3^, Kim, B. ^4^, Neuhaus, K. ^1^, Marino, D. ^2^, Farkas, M. ^1^, Bressan, J. ^1^, Reichmann, E. ^2^

^1^ Pediatric Burn Center, University Children‘s Hospital Zurich, Zurich, Switzerland, ^2^ CUTISS AG, Zurich, Switzerland, ^3^ Amsterdam UMC location Vrije Universiteit Amsterdam, Department of Plastic, Reconstructive and Hand Surgery, Amsterdam, Netherlands, ^4^ Department of Plastic Surgery and Hand Surgery, Burn Center, University Hospital Zurich, Zurich, Switzerland

Oral presentations 09—Basic research 2, 4 September 2025, 13:45–15:15

Aim:

Shortage of autologous donor sites and functional impairing scarring of split thickness skin grafts are two most difficult therapeutic problems in burn surgery. Cultured skin substitutes play a realistic therapeutic role to overcome these problems.

We present a first prospective, randomized, controlled Phase II clinical trial of denovoSkin™, a laboratory cultured autologous skin analogue, comparing it to the current gold standard the split thickness skin graft (STSG).

Methods:

In the study, 21 patients, aged ≥12 years, with large burns were enrolled. Skin defects were randomized to either denovoSkin™ or autologous STSG (control) treatment. Safety assessments and efficacy endpoints were performed in all patients including the ratio of biopsy size to grafted area 4 weeks post-grafting, wound closure and scar quality. Follow-ups were done 1-year post-grafting.

Results:

After 1 year, 15 patients were fully evaluated. There were no significant differences regarding safety. denovoSkin™ showed an expansion ratio that was 7.0 times higher (*p* < 0.001) than for STSG. Experimental and control areas were fully epithelialized (ns) by month 3. 12-months post-grafting revealed no clinically relevant scarring for denovoSkin™, but mostly hypertrophic scarring for STSG.

Conclusions:

This Phase II clinical trial shows that denovoSkin™ is a novel and safe treatment option for deep burns sparing donor sites and reducing scarring.

*O09.2 Mesenchymal stem cells in fibrin glue for burn wound treatment: a comparative in vitro study of sprayed vs. cast cells*

**Jenssen, A.** ^1,2^, Mohamed-Ahmed, S. ^3^, Al-Sharabi, N. ^3^, Blokhina, D. ^4^, Winther-Waldau, V. ^2^, Mustafa, K. ^3^, Kankuri, E. ^4^, Almeland, S. ^1,2^

^1^ Norwegian National Burn Center, Department of Plastic, Hand, and Reconstructive Surgery, Haukeland University Hospital, Bergen, Norway, ^2^ Department of Clinical Medicine, Faculty of Medicine, University of Bergen, Bergen, Norway, ^3^ Center for Translational Oral Research (TOR), Tissue Engineering Group, Department of Clinical Dentistry, Faculty of Medicine, University of Bergen, Bergen, Norway, ^4^ Department of Pharmacology, Faculty of Medicine, University of Helsinki, Helsinki, Finland.

Oral presentations 09—Basic research 2, 4 September 2025, 13:45–15:15

Aim:

This study evaluates the therapeutic potential of bone marrow-derived mesenchymal stem cells (BM-MSCs) sprayed in fibrin glue for burn wound treatment compared to cast cell application.

Methods:

Human BM-MSCs were sprayed in fibrin glue at varying pressures and compared to cast cells. Flow cytometry was used to assess stem cell surface markers directly post-spraying. A scratch wound assay with conditioned medium from sprayed vs. cast MSCs was conducted to evaluate wound healing effects. Propidium iodide (PI) staining was performed to assess cell viability after spraying at different pressures.

Results:

Flow cytometry analysis showed that MSC surface marker expression remained preserved post-spraying. Conditioned medium from sprayed MSCs enhanced wound closure in the scratch assay, indicating retained paracrine activity. PI staining revealed that cell viability was affected by increasing spray pressure, highlighting the need for optimized delivery conditions.

Conclusions:

Spraying BM-MSCs in fibrin glue maintains their phenotypic and functional properties, supporting their use as a promising approach for burn wound treatment. Further studies are needed to optimize spraying parameters for maximum therapeutic efficacy.

*O09.4 The blood coagulation profile in severe frostbite injury*

**Mämmi, I.** ^1^, Pajarinen, J. ^2^, Lassila, R. ^1^, Lindford, A. ^1^

^1^ Helsinki University Hospital, Helsinki, Finland, ^2^ Päijät-Häme Central Hospital, Lahti, Finland

Oral presentations 09—Basic research 2, 4 September 2025, 13:45–15:15

Aim:

Microvascular occlusion, either from vasospasm and/or thrombus formation, plays a crucial role in the pathogenesis of severe frostbite injury. We hypothesized that severe frostbite might cause alterations in the levels of blood coagulation factors, potentially offering novel diagnostic or prognostic tools and guiding choice of therapy.

Methods:

Ten patients treated in Helsinki University Hospital between 2021 and 2023 for severe frostbite (Hennepin score 14.85 ± 20.15) were included in the study. A comprehensive blood coagulation profile (partial thromboplastin time, antithrombin, fibrinogen, D-dimer, FVIII, thrombin time and prothrombin time) was obtained on admission. Patients underwent angiography, and if severe frostbite was confirmed on angiographic findings, they received either thrombolytic or iloprost therapy. The final extent of any distal amputations were recorded and defined as the Hennepin salvage rate.

Results:

Coagulation factors D-dimer (3.6 ± 3.4) and FVIII (271.1 ± 71.49) were elevated in all the patients. FVIII was significantly higher in patients with confirmed thrombosis in angiography than in patients with vasospasm only (327.2 ± 27.26 vs. 215.0 ± 53.74, *p* = 0.0061).

However, coagulation factor levels did not correlate with the initial extent of the injury (Hennepin score on admission) nor the final Hennepin salvage rate.

Conclusions:

Severe frostbite elevates the D-dimer and FVIII levels, likely reflecting the activation of the coagulation system. The changes in coagulation factors do not directly correlate with treatment outcomes. A marked elevation of FVIII may indicate microvascular thrombotic aetiology. Further studies will determine if these coagulation factors have utility in the diagnosis and management of frostbite injuries.

*O09.5 Insights into the healing mechanisms of acellular fish skin grafts—A preclinical study*

**Pignet, A.** ^1^, Hofmann, E. ^2^, Fink, J. ^2^, Prevedel, M. ^2^, Hahn, D. ^2^, Kamolz, L. ^1^, Kotzbeck, P. ^2^

^1^ Medical University Graz, Graz, Austria, ^2^ COREMED—Joanneum Research, Graz, Austria

Oral presentations 09—Basic research 2, 4 September 2025, 13:45–15:15

Aim:

Acellular fish skin grafts (FSG) have proven to be effective in acute and chronic wound healing, as well as in burns. The aim was to investigate the effects of fish skin grafts on wound healing properties under standardized conditions in a porcine full-thickness wound model.

Materials and Methods:

FSGs were tested in a full-thickness skin defect (3 cm × 3 cm) pig model (*n* = 9, male landrace pigs) and compared to untreated control wounds over the course of 21 days. Reapplication of the dressings was carried out after 9 days post wounding. Tissue biopsies were sampled at 5, 9, 14 and 21 days after wounding and subjected to histologic (HE and Masson’s trichrome stains) and gene expression analysis. Thickness of the newly built tissue was measured histologically. QPCR was conducted for pro- and anti-inflammatory cytokines.

Results:

As soon as on day 5, fish skin grafts showed a significantly thicker regenerated tissue compared to control wounds. The difference was accelerated through day 9 and 14 after wounding. Gene expression analysis revealed a higher upregulation of pro and anti-inflammatory markers in the first days after wounding compared to the controls. In the later phases, there were no differences between the two groups.

Conclusions:

FSGs modulate the local immune response by upregulation of both, pro- and anti-inflammatory markers. The increased initial inflammatory response might drive the accelerated formation of new tissue and hence wound closure.

*O09.6 Evaluation of a topical microdosing approach in wound healing applications*

**Prevedel, M.** ^1,2^, Moshammer, M. ^1,2^, Schwingenschuh, S. ^3^, Villa-Garcia, A. ^1,2^, Carnieletto, M. ^2^, Purgstaller, S. ^3^, Eberl, A. ^3^, Kamolz, L. ^1,2^, Birngruber, T. ^3^, Kotzbeck, P. ^1,2^, Hofmann, E. ^1,2^

^1^ COREMED—Center for Regenerative and Precision Medicine, JOANNEUM RESEARCH Forschungsgesellschaft mbH, Graz, Austria, ^2^ Division of Plastic, Aesthetic and Reconstructive Surgery, Department of Surgery, Medical University of Graz, Graz, Austria, ^3^ HEALTH-Institute for Biomedicine and Health Sciences, Joanneum Research Forschungsgesellschaft mbH, Graz, Austria

Oral presentations 09—Basic research 2, 4 September 2025, 13:45–15:15

Aim:

Difficult to treat wounds, such as burns and chronic wounds, constitute a continuous challenge. One current trend is towards developing dressings that allow for targeted delivery of active substances. However, some promising substances are hormones and while exerting positive effects on wound healing, systemic distribution may have detrimental effects. We have tested a microdosing approach to evaluate the local effects of two hormones, i.e., leptin and estrogen, on wound healing progression.

Methods:

Open-flow microperfusion (OFM) technology was adapted to load and supply leptin and 17-b-estradiol via bacterial nanocellulose (BNC) dressings to superficial wounds in porcine ex vivo and in vivo models. Sampling dermal interstitial fluid (dISF) and blood allowed to determine pharmacodynamics and pharmacokinetics. Leptin- and 17-b-estradiol-loaded BNC-dressings were applied to acute superficial excision wounds and wound healing progression was observed for 6 days, with blood and tissue sample collection for analysis of substance distribution, and of local effects on wound healing parameters.

Results:

Systemic distribution was not detected for neither leptin nor 17-b-estradiol. In dISF, leptin was shown to reach maximum concentrations of 0.5–5 ng/mL after 6 h with levels decreasing after 12 h. For 17-b-estradiol, constant levels (0.1–1 ng/mL) were observed from 2 to 24 h. No differences were observed in wound healing progression, however slight differences were observed in epidermis thickness after 17-b-estradiol treatment as compared to mock treated wounds.

Conclusions:

Functionalization of existing wound dressings using microdosing of wound healing promoting substances is a promising approach for future burn wound and chronic wound treatment.

*O09.7 The unique short glycoprotein 11: experimental treatment approaches to reduce skin allograft rejection*

**Schlottmann, F.** ^1^, Strauß, S. ^1^, Bucan, V. ^1^, Vogt, P. ^1^

^1^ Hannover Medical School, Hannover, Germany

Oral presentations 09—Basic research 2, 4 September 2025, 13:45–15:15

Aim:

Given the limited availability of autologous donor sites in severely burned patients, immunomodulation of allogeneic skin grafts without altering the recipient’s immune system is of significant clinical interest. This study aims to explore the potential of the human virus derived US11 protein to reduce major histocompatibility complex (MHC) class I expression in human keratinocytes, thereby decreasing alloreactivity of allogeneic skin grafts.

Methods:

Human primary keratinocytes were transfected with US11 vectors or treated with recombinant US11 protein to assess their impact on MHC class I expression. The reduction of MHC class I was quantified using Western blot, real-time PCR, and flow cytometry. Co-culture experiments with human PBMC were conducted to analyze immune response modulation. Additionally, allogeneic skin samples were treated with recombinant US11 proteins to investigate their influence on MHC class I expression in a complex tissue model.

Results:

Keratinocytes transfected with US11 vectors exhibited a reduction in MHC class I expression at 24 h, returning to baseline at 48 h. Recombinant US11 protein treatment resulted in a similar reduction within 6 h. Co-culture experiments demonstrated a decreased interferon-gamma concentration, indicating reduced immune activation. Allogeneic skin samples treated with recombinant US11 protein showed reduced MHC class I expression after seven days.

Conclusions:

The modification of human primary keratinocytes or allogeneic skin grafts through US11 vector transfection or recombinant US11 protein treatment presents a promising approach to reducing alloreactivity. Further in vitro and in vivo studies are needed to assess the therapeutic potential and clinical translation of this strategy.

*O09.8 Effectiveness of ‘The Basics of Burn Care Course’ in Uganda*

**Simon, M.** ^1,2^, Kazibwe, S. ^3^, Kambiz, S. ^2,4^, Botman, M. ^1,2^, Kalanzi, E. ^3^, Hofland, H. ^5,7^, de Vries, A. ^2,6^, Breugem, C. ^1,7^, Hop, J. ^8^

^1^ Department of Plastic and Reconstructive Surgery, University Medical Centre, Amsterdam, The Netherlands., Amsterdam, Netherlands, ^2^ Global Surgery Amsterdam, Amsterdam, Netherlands, ^3^ Burns and Plastic Surgery Unit, Kiruddu National Referral Hospital, Republic of Uganda, Kampala, Uganda, ^4^ Department of Plastic, Reconstructive and Hand Surgery, Groene Hart Hospital, Gouda, The Netherlands, Gouda, Netherlands, ^5^ Burns Center, Maasstad Hospital, Rotterdam, The Netherlands, Rotterdam, Netherlands, ^6^ Department of Surgery, Burn Centre, Red Cross Hospital, Beverwijk, The Netherlands, Beverwijk, Netherlands, ^7^ Interplast Holland NGO, Leiden, Netherlands, ^8^ Department of Plastic, Reconstructive and Hand Surgery, Erasmus University Medical Centre, Rotterdam, The Netherlands., Rotterdam, Netherlands

Oral presentations 09—Basic research 2, 4 September 2025, 13:45–15:15

Aim:

Burn injuries cause approximately 180,000 deaths annually and are a leading cause of disability-adjusted life years in low- and middle-income countries (LMIC), where 90% of burn injuries occur. Education of healthcare professionals in these settings is essential to improve burn care outcomes. This study aimed to evaluate the effectiveness of the Basics of Burn Care course, to improve knowledge among healthcare workers involved in burn care in Uganda.

Methods:

This three-day course was conducted in 2022 in Kampala. Local Ugandan instructors collaborated with Dutch instructors to teach healthcare professionals, including nurses, medical officers and residents. Knowledge gain was assessed through identical tests administered pre-course, immediately post-course, and six months post-course.

Results:

Participants’ mean test scores improved significantly from 6.5 (SD ± 1.5) pre-course to 8.2 (SD ± 1.3) post-course (*p* < 0.001). Six-month follow-up scores remained consistent with post-course results. Participants from 12 hospitals across Uganda reported increased confidence in managing burn injuries after the training.

Conclusions:

This study demonstrates that the Basics of Burn Care course significantly enhanced long-term knowledge in primary burn care in Uganda, suggesting it may be a useful approach for strengthening burn management across LMIC settings.

*O09.9 Thrombotic and Neutrophil-Driven Mechanisms in Burn Injury: A Longitudinal Study of Post-Burn Immune and Vascular Responses*

**van der Leeden, B.** ^1,2,3^, Kemme, F. ^3,4,5^, Pijpe, A. ^3,4,5^, Vlig, M ^6^, Mulder, P. ^5,6^, van Mierlo, G. ^7^, Meij-de Vries, A. ^4,8,9^, van Zuijlen, P. ^3,4,5,8^, Gibbs, S. ^10,11^, Niessen, H. ^1,12,13^, Boekema, B. ^5,6^, Korkmaz, H. ^3,4,5,6,11^, Krijnen, P. ^1,13^

^1^ Department of Pathology, Amsterdam UMC location Vrije Universiteit Amsterdam, Amsterdam, The Netherlands, ^2^ Amsterdam Institute for Infection and Immunity, Inflammatory diseases, Amsterdam, The Netherlands, ^3^ Amsterdam Movement Sciences, Tissue Function and Regeneration, Amsterdam, The Netherlands, ^4^ Alliance of Dutch Burn Care, Burn centre, Red Cross Hospital, Beverwijk, The Netherlands, ^5^ Department of Plastic, Reconstructive and Hand Surgery, Amsterdam UMC location VUmc, Amsterdam, The Netherlands, ^6^ Alliance of Dutch Burn Care, Burn Research Lab, Beverwijk, The Netherlands, ^7^ Depertment of Immunopathology, Sanquin Blood Supply, Divison Research, Amsterdam, The Netherlands, ^8^ Pediatric Surgical Centre, Emma Children’s hospital, Amsterdam UMC, Amsterdam, The Netherlands, ^9^ Department of Surgery, Red Cross Hospital, Beverwijk, The Netherlands, ^10^ Department of Oral Cell Biology, Academic Centre for Dentistry Amsterdam, University of Amsterdam and VU, Amsterdam, The Netherlands, ^11^ Department of Molecular Cell Biology and Immunology, Amsterdam UMC location VUmc, Amsterdam, The Netherlands, ^12^ Department of Cardiac Surgery, Amsterdam UMC, Amsterdam, The Netherlands, ^13^ Amsterdam Cardiovascular Sciences, Amsterdam, The Netherlands

Oral presentations 09—Basic research 2, 4 September 2025, 13:45–15:15

Aim:

The precise pathophysiological mechanisms behind burn wound deepening remain poorly understood, therefore this study aimed to longitudinally assess local and systemic responses in burn patients, focusing on thrombotic and neutrophil-related factors, and compare them to healthy donors.

Methods:

Biopsies and blood samples were collected from 10 burn patients at post-burn days(PBD) 0–1, 2–5, and 6–16. The biopsy sites were LDI-guided target areas within partial perfused(yellow/red) zones. Blood was obtained from 10 healthy donors for comparison. Neutrophils were isolated and stimulated with phorbol 12-myristate 13-acetate (PMA) or platelet activation factor (PAF) to assess the formation of neutrophil extracellular traps (NETosis). Flow cytometry was used to evaluate neutrophil maturity (CD10) and activation status (CD66b). Plasma levels of thrombotic markers (P-selectin, D-dimer, tPA, PAI-1, FIX) and neutrophil-related markers (DNA/MPO, HNE1ATC, Nucleosomes, DNAse1) were quantified by serological analyses. Biopsies were stained to assess burn depth (Elastin von Giesson, EVG) and microvascular thrombosis (CD31).

Results:

EVG staining showed fibrosis based on loss of dermal integrity, and CD31 staining revealed significantly increased microvascular thrombosis (*p* < 0.0001) in burn biopsies. Significant increases in thrombosis- and neutrophil-related markers were observed in burn plasma and neutrophil counts were elevated in burn patients compared to controls. Burn patient neutrophils were 5× more immature than controls and exhibited significantly higher NETosis following PMA stimulation (*p* < 0.001) but significantly lower NETosis after PAF stimulation (*p* < 0.001) up to PBD 16.

Conclusions:

This study demonstrates a local and systemic increase in both thrombotic and neutrophil-related factors post-burn, revealing a potential role for immunothrombosis as one of the possible mechanisms contributing to burn wound deepening.

*O10.1 Evaluation of the influence of blood parameters on the separation of the epidermal substitute from the wound surface in the treatment of burn wounds in children.*

**Barbachowska, A.** ^1^, Górecka, Z. ^1^, Łączyk, M. ^1^, Opaliński, H. ^1^, Winiarska, A. ^1^, Piszczek, J. ^1^, Strużyna, J. ^1^, Korzeniowski, T. ^1^

^1^ Department of Plastic, Reconstructive Surgery and Burn Treatment, Medical University of Lublin, 20-093 Lublin, Poland, Lublin, Poland.

Oral presentations 10—Pediatrics 2, 5 September 2025, 08:30–10:00

Aim:

The aim of the study was to find laboratory factors that could contribute to premature epidermal substitute dressing separation from the children’s wound surface.

Methods:

The documentation of 182 children treated for acute burns at the East Centre of Burns Treatment and Reconstructive Surgery in Leczna in the years 2009–2023 was analyzed. The demographic data, etiology, area and degree of the burn as well as the methods and effects of treatment were analyzed. The group was split into three categories based on the condition of the dressing: “attached to the wound”, “partially attached to the wound” and “detached from the wound”. Laboratory tests including hemoglobin, hematocrit, leukocyte and lymphocyte levels, protein level, C-reactive protein level, procalcitonin, glucose, creatinine and urea were collected on admission and control tests after 3–5 days after injury.

Results:

The results suggest that the Leukocytes variable differs significantly depending on the dressing condition. Statistical analysis using the Welch test showed significant differences between the groups (*p* = 0.0035).

Conclusions:

Proper wound healing under the synthetic epidermal substitute occurs when it adheres to the wound. Its premature separation from its surface may negatively affect the healing process. It seems extremely important to find factors that may contribute to its premature separation from the wound bed, so as not to prolong the healing process.

*O10.2 Comparison of Wound Healing in Grafted Areas and Untreated Gaps in Pediatric Burn Patients Using Meshed Autograft with and without Acellular Dermal Matrix*

Demircan, M. ^1^, Ateş, H. ^1^, Gül, M. ^1^, Gürünlüoğlu, K. ^1^

^1^ İnönü University School of Medicine, Malatya, Turkey

Oral presentations 10—Pediatrics 2, 5 September 2025, 08:30–10:00

Aim:

This study was conducted to clinically and histopathologically evaluate wound healing in graft-covered areas and uncovered spaces in children with severe burns who underwent mesh split-thickness skin grafting (mSTSG), and to investigate the contribution of Matriderm application to this healing process.

Methods:

24 pediatric burn patients were divided into two groups; one receiving mSTSG alone, the other receiving mSTSG with ADM (Matriderm). A control group of 12 circumcised children was also included.

Patients underwent debridement and autograft application. Biopsies were taken from both grafted areas and untreated gaps on the 14th postoperative day.

Wound healing in grafted areas was compared to untreated gaps, and the effects of ADM on the healing process were evaluated.

Results:

Improved Healing:ADM significantly accelerated wound healing compared to untreated gaps.

Enhanced Dermal Regeneration: ADM promoted better collagen production, fibroblast migration, and dermal structure.

Reduced Inflammation: ADM helped to control the inflammatory response, crucial for deep burn wound healing.

Improved Graft Stability: ADM may improve graft stability and reduce donor site limitations. 

Histological Evidence: Histological analysis showed increased epithelial thickness and dermal papilla formation in areas treated with ADM.

Conclusions:

This study provides evidence that Matriderm can be a valuable adjunct in the treatment of pediatric burn injuries. It demonstrates the potential to improve both the quality and speed of wound healing by enhancing dermal regeneration and promoting a more controlled inflammatory response.

*O10.3 Longitudinal analysis of burn scar characteristics and HRQoL in pediatric patients: a five-year follow-up study*

**Meirte, J.** ^1,2^, Anthonissen, M. ^1,2^, De Cuyper, L. ^2,3^, Van Daele, U. ^1,2^, Moortgat, P. ^2^, Demarbaix, T. ^2^, Maertens, K. ^2,4^

^1^ University of Antwerp, Movant, Antwerp, Belgium, ^2^ Oscare, Organisation for Scar and Burns After-Care And Research, Antwerp, Belgium, ^3^ ZNA Cadix Antwerp Burn Centre, Antwerp, Belgium, ^4^ VUB, department of clinical and lifespan psychology, Brussels, Belgium

Oral presentations 10—Pediatrics 2, 5 September 2025, 08:30–10:00

Aim:

We investigated longitudinal changes in burn scar characteristics and HRQoL in children post-burn.

Methods:

We performed a retrospective cohort study of children with 5 year multidisciplinary follow- up starting 2 years after burn injury. Assessments included objective scar measurements and subjective reported outcome measures (PROMs), namely Patient and Observer Scar Assessment Scale assessing scar quality and Burn Outcome Questionnaire (BOQ) assessing HRQoL.

Results:

A total of 56 children were included (mean age 5.25 years old, mean TBSA 5.98%). At the start of multidisciplinary follow-up, one third of the parents reported their child’s health as excellent, another third as very good and the remaining parents scored general health as good. The least problems were found for the BOQ domains ‘upper extremity’, ‘physical functioning and sports’ and ‘transfer and mobility’. The most problems were found for the domains ‘appearance’, ‘satisfaction with current state’ and ‘parental concern’. Over time no significant changes were found, although ‘satisfaction’ increased and issues with ‘appearance’ and ‘parental concern’ over time remained. Scar quality (POSAS sum score) improved significantly (*p* < *0*.05) over time, and for the objective scar assessments no significant changes were reported.

Conclusions:

Years post-burn, parent reported optimal scores in multiple BOQ domains. However, in out-patient settings attention for appearance and concerns seems crucial, highlighting the need for multidisciplinary readiness and swift accessibility. Our findings showed scar quality improvements over time, indicating that scar characteristics and symptoms continue to change after scar maturation.

*O10.6 Epidemiological analysis of 1185 pediatric hospitalized patients with burns and scalds in multiple centers*

Qu, Y. ^1^, Lei, B. ^1^, Liu, T. ^1^, Zhou, H. ^1^, Liu, X. ^1^, Bosheng, L., Chi, Y. ^1^

^1^ Chinese People’s Liberation Army (pla) General Hospital, Beijing, China

Oral presentations 10—Pediatrics 2, 5 September 2025, 08:30–10:00

Aim:

To explore the epidemiological and clinical characteristics of pediatric burn and scald injuries in northern China and inform targeted prevention and treatment strategies.

Methods:

A retrospective study was conducted on 1185 children (0–12 years old) hospitalized in five regional burn centers from January 2016 to December 2023. Data on demographics, injury causes, locations, pre-hospital treatment, bacterial infections, hospitalization, and follow-up were collected and analyzed.

Results:

Hot liquid burns (87.8%) were the leading cause, with most injuries occurring at home (87.7%) and in winter (28.0%). The limbs (38.2%) and trunk (25.9%) were the most affected areas. 67.6% of burns involved ≤10% TBSA. *Staphylococcus aureus* (19.6%) was the most common pathogen. The average length of hospitalization was 8–14 days, with an average cost of 15,469.7 yuan. Inhalation injury correlated with prolonged stays and higher costs.

Conclusions:

Pediatric burns in northern China are primarily caused by hot liquids, with most occurring at home and affecting the limbs and trunk. A significant number of children did not receive pre-hospital first aid. Enhancing public education on burn prevention and first-aid practices is crucial to reducing incidence and improving outcomes.

*O10.7 Shifts and Milestones in the Treatment of Pediatric Burns: a 36-Year Experience from the Romanian National Referral Center*

**Enescu, D.** ^1,2^, Nacea, I. ^1,2^, Tatar, R. ^1,2^

^1^ Carol Davila University of Medicine and Pharmacy, Bucharest, Romania, ^2^ Plastic Reconstructive Surgery and Burns Department, Grigore Alexandrescu Emergency Hospital for Children, Bucharest, Romania

Oral presentations 10—Pediatrics 2, 5 September 2025, 08:30–10:00

Aim:

The aim of this study is to analyze the evolution of pediatric burn care in Romania at the national referral center, in order emphasize the main turning points and the shifts in our approach.

Methods:

This retrospective research included all pediatric patients admitted for burn injuries, from 1988 to 2024, regardless of the TBSA, cause of injury or burn depth. The analysis included epidemiological data, age and sex group distribution, and also the trends in surgical versus conservative treatment and overall mortality rates.

Results:

During the investigated timespan, a total number of 21,000 were admitted and treated in our department. The average number of patients per year was around 560, with a general trend of decreasing over time, correlated with changes in the national pediatric population, and also with some societal and medical issues, like the recent COVID-19 pandemic. However, the number of severe cases, with affected TBSA of more than 20–30%, maintained a stable evolution. The most important finding was a constant decrease of mortality rate, which was initially of 9%, reached a 0 level in 2001 and constantly stayed around 1% afterwards, with another 0 moment in 2023. More data will be presented at the congress.

Conclusions:

Adapting constantly to the most recent advances in local and general care, moving the department to a specially designed building and introducing new devices in our therapeutic armamentarium allowed us to permanently improve the care and survival chances for our patients and reach the best international standards for burn care.

*O10.8 High-voltage electrical burns in children: epidemiological patterns and management challenges*

**Tatar, R.** ^1,2^, Enescu, D. ^1,2^, Stoica, C. ^2^, Alexandru, R. ^2^, Nacea, I. ^1,2^

^1^ Carol Davila University of Medicine and Pharmacy, Bucharest, Romania, ^2^ Plastic Reconstructive Surgery and Burns Department, Grigore Alexandrescu Emergency Hospital for Children, Bucharest, Romania

Oral presentations 10—Pediatrics 2, 5 September 2025, 08:30–10:00

Aim:

High-voltage electrical injuries represent very aggressive conditions for the human body. This study aims at analyzing epidemiological patterns and treatment protocol of this challenging clinical condition.

Methods:

We retrospectively reviewed the electronic charts of all patients admitted to our burns department between January 2020 and December 2024, in order to identify the patterns of high-voltage electrical injuries and the challenges related to specific patient management.

Results:

We retrieved 35 cases of high-voltage electrical burns, accounting for 64.81% of all hospitalized electrical burns (*n* = 54). Half of cases were caused by train climbing, and 23% occurred by touching the power lines with a fishing rod. The frequency of train climbers was very low in 2020, but constantly increased afterwards. Most of patients were male with an average age of 13 years old (range 5–17). The mean TBSA was 49% (range 5–80%). Only one patient died during hospital stay. 81% of cases required surgical treatment that included escharotomies, segment amputations, excision and grafting with regular meshed grafts or Meek micrografts). We also used negative pressure wound therapy and free flap transfer for achieving complete wound closure. The mean length of hospitalization was 50.5 days.

Conclusions:

High voltage electrical burns are rare but devastating injuries, with high morbidity and mortality. Their management requires enormous human and material resources and they leave life-long physical and psychological scars. Preventing campaigns directed toward the main epidemiological patterns, especially train climbing for taking selfie pictures, might help avoiding such complex pathological conditions.

*O10.9 Hyper-inflammatory response in burn patients coincides with impaired antibacterial activity of neutrophils in blood and burn tissue*

**Mulder, P.** ^1,2,3^, van Hooren, M. ^1^, Vermij, M. ^1^, Gans, C. ^1^, Vlig, M. ^1^, Pijpe, A. ^3,4^, van Zuijlen, P. ^2,3,4,5,6^, Koenen, H. ^7^, Boekema, B. ^1,2,3^

^1^ Burn Research Lab, Alliance of Dutch Burn Care, Beverwijk, Nederland, ^2^ Department of Plastic, Reconstructive and Hand Surgery, Amsterdam UMC, Amsterdam, Netherlands, ^3^ Tissue Function and Regeneration, Amsterdam Movement Sciences, Amsterdam, Netherlands, ^4^ Burn Center, Red Cross Hospital, Alliance of Dutch Burn Care, Beverwijk, Netherlands, ^5^ Department of Plastic and Reconstructive Surgery, Red Cross Hospital, Beverwijk, Netherlands, ^6^ Department of Paediatric Surgery, Amsterdam UMC, Amsterdam, Netherlands, ^7^ Laboratory of Medical Immunology, Radboud University Medical Center, Nijmegen, Netherlands

Oral presentations 10—Pediatrics 2, 5 September 2025, 08:30–10:00

Aim:

Burn injuries trigger severe inflammation and increased susceptibility to bacterial infections, leading to higher mortality and delayed recovery. Understanding immune reactions post-burn is crucial for developing therapies that prevent complications and promote healing. We hypothesize that despite hyperinflammation, neutrophil antimicrobial function is compromised.

Methods:

Blood and skin samples from burn patients and healthy controls were collected during routine procedures. Leukocytes isolated from blood and tissue were analyzed by flow cytometry to identify immune cell subsets and activation status. Cytokines were measured using Luminex assays. Neutrophil functionality was assessed via FITC-labeled S. aureus phagocytosis, ROS production, and IL-8-induced migration.

Results:

Burn patients (*n* = 20) exhibited systemic inflammation with surges in CD10- neutrophils and monocytes, and elevated IL-6, IL-8, MCP-1, and MIP-3α for at least 30 days. Lymphocyte counts did not alter but shifted towards a pro-inflammatory phenotype. Burn tissue (*n* = 35) contained increased CD10+ neutrophils, monocytes, and lymphocytes, alongside high levels of MCP-1, IL-8, GROα, and MIP-1β, creating a pro-inflammatory environment. Functional analysis revealed impaired neutrophil phagocytosis, ROS production, and migration in both blood (*n* = 7) and burn wounds (*n* = 18).

Conclusions:

Our findings demonstrate that while burn injuries lead to a marked increase in innate immune cell numbers, the antibacterial function of neutrophils is significantly impaired, heightening infection risk in patients. Therapies must reduce inflammation while enhancing antimicrobial defenses. Moving forward, we are now simulating these neutrophil responses in animal-free full skin equivalent models, providing a novel approach to studying immune dysfunction and testing interventions.

*O11.1 The course of scar formation and predictors of scar quality in 490 children up to one year after burns: a real-world observational study*

**Kemme, F.** ^1,2,3^, Brantjes, I. ^1^, Stoop, M. ^1,2,3^, Meij-de Vries, A. ^1,4,5^, van Meijeren, R. ^6^, van Zuijlen, P. ^1,2,3,5,6^, Middelkoop, E. ^1,2,3^, Pijpe, A. ^1,2,3^

^1^ Alliance of Dutch Burn Care, Burn Center, Red Cross Hospital, Beverwijk, The Netherlands, ^2^ Amsterdam UMC location Vrije Universiteit Amsterdam, Department of Plastic, Reconstructive, and Hand Surgery, Amsterdam, The Netherlands, ^3^ Amsterdam Movement Sciences, Tissue Function and Regeneration, Amsterdam, The Netherlands, ^4^ Department of Surgery, Red Cross Hospital, Beverwijk, The Netherlands, ^5^ Amsterdam UMC location University of Amsterdam, Pediatric Surgical Center, Emma Children’s Hospital, Amsterdam, The Netherlands, ^6^ Department of Plastic Reconstructive and Hand Surgery, Red Cross Hospital, Beverwijk, The Netherlands

Oral presentations 11—Scars 2, 5 September 2025, 08:30–10:00

Aim:

To provide insights into the course of scar formation and to identify predictors of scar quality in children aged 0–17 years up to one year after burns.

Methods:

Scar quality was assessed in children (<18 years old at the time of the burn) during routine clinical follow-ups at 3, 6, and 12 months post-burn using the Patient and Observer Scar Assessment Scale (POSAS) and the Dermaspectrometer (DSM Colorimeter). Descriptive analyses were conducted to examine changes in scar characteristics over time. Multivariable linear and logistic regression analysis were performed to identify predictors of scar quality and pruritus and pain.

Results:

A total of 946 scars in 490 children were assessed. At 12 months, 83% of children’s scars differed from normal skin, with the largest difference for color. Erythema and melanin differences with normal skin significantly decreased over time. Patient and observer overall opinion significantly improved over time, however, thickness and relief appeared to worsen. Also, patients consistently rated their scars 1.5 point worse than observers. Long-term scar quality, at 12 months, was predicted by scar location, etiology, length of stay, and short-term scar quality. Chronic pruritus (60%) was more prevalent than chronic pain (12%), with (flash)flame and oil/fat burns increasing the odds of chronic pruritus.

Conclusions:

Overall, scar quality in children improves within the first year post-burn, but some characteristics worsen, and pruritus complaints remain highly prevalent. The findings of this real-world study provide valuable insights for patient counseling and clinical decision-making regarding scar management in pediatric burn patients.

*O11.3 Use of laser therapy for treatment of hypertrophic scars of burned patients: experience in a tertiary center and review of outcomes.*

**Martin-Playa, P.** ^1^, Ugedo-Alzaga, J. ^2^, Gonzalez-Alaña, I. ^1^, Estradera-Lainez, J. ^1^, Carames-Estefania, J. ^1^, Diaz-Ramon, J. ^2^

^1^ Cruces University Hospital, Bilbao, Spain, ^2^ Cruces University Hospital, Bilbao, Spain

Oral presentations 11—Scars 2, 5 September 2025, 08:30–10:00

Aim:

The aim of this study is to analyse clinical results and quality of life of patients treated with laser therapy for hypertrophic burn sequela at our center.

Methods:

We retrospectively (2021–2024) analysed patients with postburn hypertrophic scars (regardless of their TBSA and management: conservative vs skin grafting, surgical vs enzymatic debridement) treated using fractional CO2 laser with or without laser-assisted drug delivery (LADD), pulsed dye laser, combined or not with triamcinolone acetonide. Results were evaluated using modified Vancouver Scar Scale (mVSS), POSAS 2.0, SCAR-Q, and DLQI.

Results:

13 patients completed the study. Laser treatment began 10 months post-burn (5–31 months). Improvement was observed in the mVSS in 12 out of 13 patients, with a mean score of 4.2 out of 15. Improvement was found in the observer component of the POSAS scale in both items and in patient component in all three items. A mean decrease in 2 points in DLQI scale and improvement in all three subdomains of SCAR-Q with a mean of 15 points were obtained.

Conclusions:

Laser treatment has shown to be effective in hypertrophic scars, but there is little experience in postburn sequelae. In our series of burned patients, it showed improvement in the aesthetic component, but a more subtle improvement was observed in the symptomatic and psychosocial components.

Burned patients are specially complex due to the extensive area affected and significant physical, symptomatic, and psychosocial sequelae associated. We believe that more studies are needed in order to develop standarised protocols that help get better results.

*O11.4 Comparing traditional and AI-driven approaches for conducting critical appraised topics in burn care*
  **Moortgat, P.** ^1,2^, Meirte, J. ^1,3^, Demarbaix, T. ^1,3^, Anthonissen, M. ^1,3,4^, Maertens, K. ^1,5^, Van Daele, U. ^1,3^

^1^ Oscare, Organisation for Burns, Scar Aftercare and Research, Antwerp, Belgium, Antwerp, Belgium, ^2^ BridgewAI, Antwerp, Belgium, ^3^ Department of Rehabilitation Sciences and Physiotherapy REVAKI-MOVANT, Faculty of Medicine and Health Sciences, University of Antwerp, Wilrijk, Belgium, ^4^ Department of Rehabilitation Sciences, KU Leuven, Leuven, Belgium, ^5^ Department of Clinical and Lifespan Psychology, Vrije Universiteit Brussel, Brussels, Belgium.

Oral presentations 11—Scars 2, 5 September 2025, 08:30–10:00

Aim:

Critical Appraised Topics (CATs) are summaries of the best available evidence on a specific clinical case. They are essential for evidence-based burn care but can be time-consuming. This study compares the efficiency and completeness of three CAT methodologies: the traditional manual approach, a multimodal AI language-model (m-LLM), and the newly developed complex reasoning AI language-model (cr-LLM) utility.

Methods:

Three separate CATs were conducted using each method per CAT. The traditional approach involved a manual literature review and synthesis. A m-LLM was used with refined prompts to generate CATs, while a cr-LLM autonomously conducted an extensive literature review. The time required, number of papers analyzed, and completeness of results were compared.

Results:

The traditional method required an average of 4.3 h per CAT, analyzing 4 papers, with occasionally incomplete findings. The m-LLM significantly reduced completion time (3.4 min) but produced general results, analyzing 5 papers, requiring advanced prompt engineering for improved quality. The cr-LLM achieved highly detailed and transparent CATs in 10,2 min, reviewing 17 papers on average. In terms of accuracy, using the HLE-benchmark test, the cr-LLM outperforms both other methods (X2 = 4.85 *p* = 0.032). This method provided the most comprehensive and structured results.

Conclusions:

AI-driven approaches offer significant time savings over traditional CATs. A multimodal AI language model provides rapid but generalized results, requiring expert refinement. The complex reasoning AI language model strikes a balance between speed and completeness, producing the most thorough evidence synthesis. This study highlights the potential of AI-assisted CATs to optimize evidence-based decision-making in burn care.

*O11.5 Development of a reconstructive algorithm for eyelid burns through a systematic review of the literature*

**Páez Ordoño, C.** ^1^, Iglesias Huerga, J. ^2^, Marino García, M. ^3^, Yuste Benavente, V. ^4^

^1^ Hospital Universitario Miguel Servet, Zaragoza, Spain, ^2^ Hospital Universitario Miguel Servet, Zaragoza, Spain, ^3^ Hospital Universitario Miguel Servet, Zaragoza, Spain, ^4^ Hospital Universitario Miguel Servet, Zaragoza, Spain

Oral presentations 11—Scars 2, 5 September 2025, 08:30–10:00

Aim:

To review the surgical techniques used for eyelid reconstruction in facial burns, both in acute and chronic situations, in order to create a treatment algorithm to guide decision-making.

Methods:

A systematic review was conducted in MEDLINE, Web of Science, and Cochrane Library, including articles published between 2004 and 2024. After excluding duplicate articles, those with a different pathogenesis, those focused on corneal treatment, those in languages other than English or French, and those without an abstract, a total of 43 articles were included. A reconstructive algorithm was established based on the availability of viable perilesional tissue.

Results:

Of the 43 articles included in the review, 19 focus on the acute phase of eyelid burns, 19 address the chronic sequelae of eyelid contracture, and 5 describe both stages. Regarding surgical techniques, 27 articles analyze reconstruction using skin grafts, 13 focus on reconstruction with flaps (free, regional, and local), 16 explore various coverage and reconstruction procedures (tarsorrhaphy, cantopexy, lipofilling, suspension techniques), and 3 articles discuss reconstruction using skin substitutes. It is important to note that several studies investigate more than one therapeutic procedure, reflecting the variety of approaches used in the treatment of eyelid burns

Conclusions:

In the acute phase, ocular surface protection should be provided with hydration and coverage through tarsorrhaphy or grafts. Contracture release is achieved through debridement and reconstruction using local or free flaps, as well as skin grafts depending on the surrounding skin’s condition. In chronic sequelae, a similar approach is necessary to improve eyelid motility.

*O11.6 Towards standarized assessment in research and clinical practice: introducing a core outcome set for burn scar quality*

**Stoop, M.** ^1,2^, van den Bosch, A. ^1,2,3^, van Zuijlen, P. ^1,2,3,4,5^, Kemme, F. ^1,2,3^, Meij-de Vries, A. ^1,5,6^, Middelkoop, E. ^1,2,3,7^, Pijpe, A. ^1,2,3^

^1^ Alliance of Dutch Burn Care, Burn Centre, Red Cross Hospital, Beverwijk, The Netherlands, ^2^ Department of Plastic, Reconstructive & Hand Surgery, Amsterdam UMC location VUmc, Amsterdam, The Netherlands, ^3^ Amsterdam Movement Sciences, Tissue Function and Regeneration, Amsterdam UMC, Amsterdam, The Netherlands, ^4^ Department of Plastic Surgery, Red Cross Hospital, Beverwijk, The Netherlands, ^5^ Paediatric Surgical Centre, Emma Children’s Hospital, Amsterdam UMC, Amsterdam, The Netherlands, ^6^ Department of Surgery, Red Cross Hospital, Beverwijk, The Netherlands, ^7^ Alliance of Dutch Burn Care (ADBC), Burn Research Lab, Beverwijk, The Netherlands

Oral presentations 11—Scars 2, 5 September 2025, 08:30–10:00

Aim:

Implementation of a core outcome set (COS) to measure burn scars.

Method

Historically, our research has focused on the development of measurement tools, along with extensive studies into their validity and reliability. Over time, we gained valuable experience in applying various methods, which ultimately led to the creation of a comprehensive COS for burn scar assessment, designed to include not only Patient-Reported Outcome Measures and Clinical-Reported Outcome Measures, but also the use of devices. This COS encompasses comprehensive, non-invasive, and affordable measurement instruments.

Results:

The COS can be used in research and clinical practice. For routine clinical practice, we use photography and the Patient and Observer Scar Assessment Scale. For clinical trials, colorimetry and elasticity (cutometer) assessment are also applied. Time points for assessment are 3 (6, 9) and 12 months post-burn. To capture important acute phase parameters influencing scar quality, it is recommended to assess certain patient and burn characteristics as well as the time to wound healing by graft take and re-epithelialization. High-quality outcome assessment necessitates standardized measurement conditions, trained personnel, standard operating procedures, and regular calibration of devices. Specific aspects for outcome assessment in pediatric patients and remote assessment should be considered.

Conclusions:

The implementation of a COS for burn scar quality assessment enables us to evaluate the effectiveness of healthcare interventions, tracking patient progress, and for comparing results across different studies or patient populations. By sharing our experience we provide a comprehensive toolbox which can easily be adopted by professionals in burn care worldwide.

*O11.7 Bibliometric analysis of studies on management of post-burn itching*

**Tuncbilek, Z.** ^1^, Acikgoz, F. ^1^, Aktas, H. ^2^

^1^ Hacettepe University, Ankara, Turkey, ^2^ Dogus University, Istanbul, Turkey

Oral presentations 11—Scars 2, 5 September 2025, 08:30–10:00

Aim:

Itching following burn injuries is common, occurring in 87% of adult patients and 93% of pediatric patients. So many modalities that target itching through different mechanisms are used to treat post-burn pruritus. Therefore, this study is applied to reveal research trends in this field and to predict future research hot spots.

Methods:

This study was performed as a bibliometric analysis. The Web of Science Core Collection (WoSCC) database was retrieved on 28 March 2025. The search strategy was (TS = (“post-burn itch*” OR “burn-induced itch*” OR “pruritus after burn*” OR “burn injury itch*” OR “post-burn pruritus”)). This analysis included studies published between 2002 and 2024, written in English. VOSviewer software was used for mapping quantitative data on countries, journals, and keywords.

Results:

By searching the database, 40 articles were identified. Of these, 22 articles were related to management of post-burn itching. Pharmacological and non-pharmacological management, and both accounted for 72.7%, 18.2% and 9.1% of the articles, respectively. The United States of America (*n* = 7) had the highest number of publications. Moreover, India (*n* = 4) and Republic of Korea (*n* = 3) also contributed. The most popular journal in this field was Burns with 7 documents cited 205 times, and the most cited (*n* = 73) document was Murphy (2003). Of the 45 keywords, the most clustered around post-burn pruritus/pruritus, burn(s), itch and gabapentin.

Conclusions:

This bibliometric analysis reveals that research on post-burn itching management predominantly focuses on pharmacological approaches, with the USA leading in publications, and Burns being the most influential journal in the field.

*O11.8 Identification of potential therapeutic target SPP1 and related rna regulatory pathway in keloid based on bioinformatics analysis*

**Xie, R.** ^1^, Chen, J. ^1^, Li, Z. ^1^

^1^ Department of Burn and Plastic Surgery, Sichuan University West China Hospital, Chengdu, China

Oral presentations 11—Scars 2, 5 September 2025, 08:30–10:00

Aim:

To explore the complex mechanisms of keloid, new approaches have been developed by different strategies. However, conventional treatment did not significantly reduce the recurrence rate. This study aimed to identify new biomarkers and mechanisms for keloid progression through bioinformatics analyses.

Methods:

In our study, microarray datasets for keloid were downloaded from the GEO database. Differentially expressed genes (DEGs) were identified by R software. Multiple bioinformatics tools were used to identify hub genes, and reverse predict upstream miRNAs and lncRNA molecules of target hub genes. Finally, the total RNA-sequencing technique and miRNA microarray were combined to validate the identified genes.

Results:

Thirty-one DEGs were screened out and the upregulated hub gene SPP1 was finally identified, which was consistent with our RNA-sequencing analysis results and validation dataset. In addition, a ceRNA network of mRNA (SPP1)-miRNA (miR-181a-5p)-lncRNA (NEAT1, MALAT1, LINC00667, NORAD, XIST and MIR4458HG) was identified by the bioinformatics databases. The results of our miRNA microarray showed that miR-181a-5p was upregulated in keloid, also we found that the lncRNA NEAT1 could affect keloid progression by retrieving the relevant literature.

Conclusions:

We speculate that SPP1 is a potential candidate biomarker and therapeutic target for patients with keloid, and NEAT1/miR-181a-5p/SPP1 might be the RNA regulatory pathway that regulates keloid formation.

*O12.1 Burn injuries in epileptic patients: a 10-year review from a single center in Istanbul, Turkey*

**Erden, R.** ^1^, Gökçe, Y. ^1^, Dereli, Z. ^2^, Filinte, G. ^1^

^1^ Kartal Dr. Lütfi Kırdar City Hospital Plastic Reconstructive and Aesthetic Surgery Department, Istanbul, Turkey, ^2^ Kartal Dr. Lütfi Kırdar City Hospital General Surgery Department, Istanbul, Turkey

Oral presentations 12—Wounds & Psychosocial and Psychiatry, 5 September 2025, 08:30–10:00

Aim:

Epilepsy is a severe neurological dysfunction characterized by recurrent seizures accompanied by loss of consciousness. Burn injuries occurring in epilepsy patients are often deep burns due to the loss of consciousness during seizures. In this study, epileptic burn patients, the treatment process, and possible preventive measures were evaluated.

Methods:

A total of 65 patients who presented with burn injuries occurring during epileptic seizures at the Burn Center of Dr. Lütfi Kırdar City Hospital between January 2015 and January 2025 were included in the study. The patients’ mean age, gender, average follow-up periods, burn degrees, burn percentages, causes of burns, and treatment modalities were evaluated retrospectively.

Results:

Among the 65 patients, 22 were male (33.82%), and 43 were female (66.18%), with a mean age of 46.9 years. The average follow-up period was 73.08 days, and the mean total body surface area burned was 8.22%. Only 3.1% of the injuries were first-degree burns, while 40% were second-degree, and 56.9% were third-degree burns. The most common burn site was the right hand (27.9%). Scald burns were the most frequent type, observed in 53.8% of the cases. A total of 58 patients (89.2%) were followed with hospitalization. The most commonly performed surgical procedure was escharotomy and repair with STSG (69.1%).

Conclusions:

Burns resulting from epileptic seizures are often deep and full-thickness burns, and treatment frequently involves the use of dermal matrices, negative pressure wound therapy and reconstruction with grafts. Early detection of these burns, as well as timely debridement and reconstruction, is recommended.

*O12.2 Revisiting an old acquaintance—Immediate burn wound excision in practice*

**Hundeshagen, G.** ^1^, Öztürk, M. ^1^, Palackic, A. ^1^, Niederegger, T. ^1^, Schaschinger, T. ^1^, Brandt, J. ^1^, Hannmann, T. ^1^, Kneser, U. ^1^

^1^ BG Trauma Center Ludwigshafen, Ludwigshafen, Germany

Oral presentations 12—Wounds & Psychosocial and Psychiatry, 5 September 2025, 08:30–10:00

Aim:

Early excision is a mainstay of modern burn surgery. Recently, immediate excision—performed within 24 h post-trauma, often directly after admission—has reemerged as a strategy to rapidly convert burn wounds into surgical wounds. The objective of this analysis is to evaluate the safety and practicability of immediate excision in acute burn care.

Methods:

Over the course of one year, burn patients who underwent their first surgical excision within 24 h of hospital admission were retrospectively analyzed. Procedures limited to superficial debridement or escharotomy were excluded. Collected data included patient demographics, intraoperative parameters, and early complications.

Results:

Fourteen patients met inclusion criteria. The median age was 57.5 years (IQR 41.5–69.3) with a TBSA involvement of 31.5% (IQR 15.0–59.5). Median time from admission to surgery was 3.5 h (IQR 2.0–14.6), and the median operative duration was 120 min (IQR 57.5–180.0). Intraoperative minimum temperatures had a median of 36.25 °C (IQR 35.5–36.63), with a median temperature drop of 1.0 °C (IQR 0.8–1.275). Median fluid volume infused was 2642 mL (IQR 1736–4998). No major intraoperative complications were observed.

Conclusions:

Immediate excision within 24 h of admission appears feasible and safe in selected burn patients. Despite the urgency and potential physiological stress, operative parameters—including temperature control and fluid management—remained within acceptable ranges. These findings support the continued use and further study of immediate excision as part of modern burn care strategies.

*O12.3 The impact of medical robots: 3D skin printing in situ as a promising future solution in tissue regeneration*

**Maitz, P.** ^1,2,3^, Maitz, J. ^1,2,3^

^1^ Faculty of Medicine and Health, University of Sydney, Sydney, Australia, ^2^ Burns and Reconstructive Surgery Research Group, Concord Hospital, Sydney, Australia, ^3^ Burns and Reconstructive Surgery Unit, Concord Hospital, Sydney, Australia

Oral presentations 12—Wounds & Psychosocial and Psychiatry, 5 September 2025, 08:30–10:00

The term “robot” originates from the Czech word robota, meaning labor, first introduced by Josef and Karel Čapek in 1921. Since its first industrial use in 1958 by General Motors, robotics has revolutionized multiple fields, including deep-sea exploration, space travel, and medicine. In 1985, a robotic arm performed the first stereotactic brain biopsy, paving the way for complex surgical systems. The development of the Da Vinci robot in 2000 marked a milestone, enabling precise, minimally invasive surgery.

Building on this, the Verb Surgical System, a collaboration between Johnson & Johnson and Google, integrates robotics, advanced visualization, and AI-driven data analytics, signaling the next wave of intelligent surgical automation.

While most surgical robots focus on removing tissue—such as in orthopedic and oncologic procedures—the next frontier is regenerative robotic surgery. We present the world’s first robotic 3D bioprinting system capable of fabricating multilayered living skin tissue in situ within a wound. This system integrates high-speed printheads, a computer-controlled robotic arm, and advanced wound visualization for automated task execution. Preclinical studies in animal models with partial- and full-thickness skin defects have demonstrated successful integration of bioprinted tissue. The first human clinical trial is now underway. This presentation will highlight the potential of robotic soft tissue creation, automation in wound reconstruction, and the future implications for surgical innovation.

*O12.4 The future of bio-printing skin: pre-clinical and clinical outcomes of 3D printing skin in-situ*

**Maitz, J.** ^1,2^, Boyling, A. ^1,2^, Hou, L. ^2^, Dao, A. ^2^, Nieuwendyk, K.^2^, Daikuara, L. ^4^, Pei, Y. ^3^, Holt, B. ^3^, Leavens, J. ^3^, Maitz, P. ^1,2^

^1^ Faculty of Medicine & Health, University of Sydney, Sydney, Australia, ^2^ Burns & Reconstructive Surgery Research, Concord Repatriation General Hospital, Sydney, Australia, ^3^ Inventia Life Sciences, Sydney, Australia, ^4^ Australian Institute for Innovative Materials, University of Wollongong, Sydney, Australia

Oral presentations 12—Wounds & Psychosocial and Psychiatry, 5 September 2025, 08:30–10:00

Introduction:

Skin tissue engineered solutions have been adopted into clinical practice to treat all types of wounds ranging from burn injuries to chronic wounds. However, many current cell-based systems have limitations including cellular run-off and complex culturing methods. Due to these limitations, cells and their delivery systems are often adjunct treatment modalities in the management of burns. The use of highly advanced delivery systems to build constructs that represent the native human skin are at the forefront of research and in these studies we evaluate the use of a robotic 3D bio-printer in-situ to promote tissue regeneration and repair.

Methodology:

LIGO, a surgical robot capable of 3D bio-printing, can produce a cell-based construct directly on a wound, reconstructing the skin in layers in-situ. We assessed the safety and delivery of different autologous cell types derived from split thickness skin grafts on wounds generated in a porcine model and in a clinical trial.

Results:

The results demonstrated an increase in re-epithelisation rate of 3D printed skin compared to healing by secondary intention in the porcine model. The outcomes from this study have enabled the translation to a first in human clinical trial; a safety study to deliver keratinocytes within a biomaterial matrix to a surgically generated wound with promising preliminary findings.

Conclusions:

The results from this pre-clinical and clinical study demonstrated its safety and efficacy in treating controlled and non-complicated wounds. The use of this delivery system in tissue regeneration is a promising step in the advances for skin tissue engineering.

*O12.5 Multispectral Imaging and Assessment of Burns Healing Potential: A Prospective Real-World Study to assess the DeepView Snapshot Imaging Platform*

**Snelling, S.** ^1^, Abouhadid, S. ^1^, Nabil Rofaeel Ibrahim, F. ^1^, Noakes, A. ^1^, Wearn, C. ^1^

^1^ North Bristol NHS Trust, Bristol, United Kingdom

Oral presentations 12—Wounds & Psychosocial and Psychiatry, 5 September 2025, 08:30–10:00

Aim:

Assessing the predictive value of the DeepView Snapshot Imaging Platform (DVS) in evaluating burn depth and predicting healing.

Introduction:

Burns depth and subsequent healing potential is an essential part of clinical assessment and is affected by the examiner’s expertise and the dynamic nature of burn wounds. Misdiagnosis can result in unnecessary surgical or delayed definitive management. DVS combines multispectral imaging with an Al predictive model to assess burn depth and predict healing.

Methods:

Authors performed a prospective study in a single centre UK Burns Service. Adults attending with a thermal burn within 7 days of injury were included. Exclusions included specialist areas and patients undergoing excision and grafting. Clinician assessment of depth was compared to the DVS. Healing was defined as greater than 95% healed within 21 days.

Results:

55 patients with 98 regions of interest were included. DVS had a negative predictive value (NPV) of 0.85, Sensitivity of 0.58 and Specificity of 0.86 (*p* < 0.001). Clinician assessment had a NPV of 0.84, Sensitivity of 0.58 and Specificity of 0.79 (*p* = 0.001). There was no statistical different difference in NPV, Sensitivity or Specificity between DVS and clinician assessment (*p* = 0.22).

Conclusions:

The DVS is a rapid, simple, and pain-free method of assessing burns depth. DVS is as accurate in assessing burn healing potential as specialist burns clinician’s assessment. DVS has future capabilities in supporting early diagnosis of non-healing burns and subsequent management.

*O12.6 Retrospective Analysis of Kerecis Omega3 Fish Skin Grafts in Burn and Wound Management: Clinical Outcomes and Utilization Patterns*

**Sood, R.** ^1^

^1^ JMS Burn Centers, Augusta, United States

Oral presentations 12—Wounds & Psychosocial and Psychiatry, 5 September 2025, 08:30–10:00

Aim:

This study aimed to determine utilization patterns and preliminary outcomes of Kerecis Omega3 fish skin grafts (Ísafjörður, Iceland) in the treatment of burn injuries and complex wounds requiring skin substitution.

Methods:

We conducted a retrospective review of 387 unique cases involving burn injuries and complex wounds treated with Kerecis products between January 2022 and September 2024. Data was extracted from institutional surgical records documenting product usage, procedure types, and surgeon preferences. The most commonly used Kerecis products included the 540 cm^2^ Coverage Mesh (2:1), 252 cm^2^ Coverage Omega3 Graft Mesh, and 126 cm^2^ Coverage Omega3 Graft Mesh. A total of 20 different surgeons utilized these products across the study period.

Results:

Preliminary data analysis revealed that Kerecis products were applied for burn and wound grafting procedures. The most frequently performed procedures included skin substitute grafting for the face/neck/head (27%), trunk/arm/leg (35%), and full-thickness skin grafting (21%). Analysis evaluates clinical outcomes including days to definitive grafting, graft take percentages, need for additional skin treatments, and long-term scar quality assessment.

Conclusions:

This retrospective analysis provides valuable insights into the utilization patterns of Kerecis Omega3 fish skin grafts in burn and complex wound management. Complete outcome analysis will further elucidate the efficacy of these products in promoting wound healing and improving scar outcomes. These findings may help establish evidence-based protocols for the optimal application of fish skin-derived products in burn care.

*O12.7 The new multimodal approach using EDNXin the treatment of deep dermal and full thicknessfacial burns*

**Taskov, P.** ^1,2^, Corodati, D. ^1^, Crainiceanu, Z. ^1,2^

^1^ Burn Unit County Emergency Clinical Hospital “Pius Branzeu”, Timisoara, Romania, ^2^ University of Medicine and Pharmacy “Victor Babes”, Timisoara, Romania

Oral presentations 12—Wounds & Psychosocial and Psychiatry, 5 September 2025, 08:30–10:00

Aim:

New multimodal approach after selective enzymatic debridement for deep facial burns.

Methods:

In this retrospective observational study (“EDNX facies”) were included 15 patients, 10 males and 5 females, 3 of them with airways burns, average age 35.7 years old, average days for hospitalization—28, average TBSA—23.8%, ABSI (media) 7.6.

Results:

The following aspects were observed

- Safety of the treatment—(off label).
- No adverse events (hemodynamic or inflammatory effects) were detected.
- In ~2 weeks all lesions treated with this technique were healed and without the need for additional surgical interventions.
- Elasticity, quality and aesthetic appearance of the scars were superior compared to the quality of the scars after SOC.
- Reduce number of lost work days (LWDs).
- Faster psychosocial reintegration.

Conclusions:

Deep facial burns are often a challenge, especially due to the fact that they are often associated with airways burns that lead to fast destabilization of the patient.

Life satisfaction is an increasingly used outcome parameter to assess mental wellbeing after a burn injury.

Burn injuries treated by this regenerative technique were associated with decreased LWDs, STD and LTD in comparison with SOC and also an increased quality of life.

*O12.8 Unmasking Stigma: Understanding and Overcoming the Psychological Impact of Facial Burns—A Systematic Review*

Niederegger, T. ^1^, **Ashgar, M.** ^2^, Schaschinger, T. ^1^, Karakas, E. ^1^, Knoedler, L. ^4^, Klimitz, F. ^5^, Kauke-Navarro, M. ^5^, Knoedler, S. ^5^, Brandt, J. ^1^, Palackic, A. ^3^, Lellouch, A. ^6,7,8^, Panayi, A. ^3,4^, Kneser, U. ^3^, Hundeshagen, G. ^3^

^1^ University of Heidelberg, Medical Faculty, Heidelberg, Germany, ^2^ University of Saskatchewan, College of Medicine, Saskatoon, Canada, ^3^ Department of Hand, Plastic and Reconstructive Surgery, Burn Center, BG Trauma Center Ludwigshafen, University of Heidelberg, Ludwigshafen, Germany, ^4^ Charité—Universitätsmedizin Berlin, corporate member of Freie Universität Berlin, Humboldt-Universität zu Berlin, and Berlin Institute of Health, Department of Oral and Maxillofacial Surgery, Berlin, Deutschland, ^5^ Department of Surgery, Division of Plastic Surgery, Yale School of Medicine, New Haven, USA, ^6^ Division of Plastic and Reconstructive Surgery, Cedars-Sinai Hospital, Los Angeles, USA, ^7^ Vascularized Composite Allotransplantation Laboratory, Center for Transplantation Sciences, Massachusetts General Hospital, Harvard Medical School, Boston, USA, ^8^ Université Paris Cité, Inserm The Paris Cardiovascular Research Center, Team Endotheliopathy and Hemostasis Disorders, Paris, France 2- AP-HP, Hôpital Européen Georges Pompidou, Hematology department, Paris, France.

Oral presentations 12—Wounds & Psychosocial and Psychiatry, 5 September 2025, 08:30–10:00

Aim:

This systematic review provides the most comprehensive synthesis to date of the psychosocial burden of facial burns, a frequently disfiguring injury whose stigmatizing impact on mental health and social reintegration remains underexplored despite growing awareness.

Methods:

A systematic review was conducted in accordance with PRISMA 2020 guidelines, analyzing literature from PubMed/MEDLINE, EMBASE, and Web of Science databases through February 2025. Studies examining stigma, psychological distress, and social reintegration in facial burn survivors were included. A narrative synthesis was employed due to methodological heterogeneity.

Results:

Twenty-two studies from 1976 to 2024 involving 1927 patients were included. Stigmatization was most pronounced in individuals with visible facial scars, functional impairments, and lower socioeconomic status, correlating with higher rates of depression, anxiety, and social isolation. Children, women, and financially insecure individuals were at highest risk. Quantitative analyses revealed elevated stigma scores and diminished self-esteem. Interventions such as resilience training, peer support programs, reconstructive surgery, and community education significantly improved psychosocial outcomes. Additionally, structured reintegration programs and burn camps were particularly effective in improving social confidence and reducing feelings of isolation, especially among pediatric patients.

Conclusions:

Stigma following facial burns remains a critically underappreciated barrier to full recovery. A multidisciplinary approach—including psychological counseling, reconstructive surgery, and public education—is essential for reducing stigma and enhancing quality of life. Future research should prioritize standardized stigma assessment tools and explore long-term trajectories to inform targeted, culturally sensitive interventions.

*O13.1 Epidemiology and clinical characteristics of adult ICU burn patients: A 12-year review from a UK regional burn centre*

**Dixon, N.** ^1^, Thornton, M. ^2^, Sloan, B. ^3^

^1^ Dorset County Hospital, Dorset, United Kingdom, ^2^ Royal Bolton Hospital, Greater Manchester, United Kingdom, ^3^ Mid Yorkshire Teaching NHS Trust, Leeds, United Kingdom

Oral presentations 13—Critical Care and Anaesthesia, 5 September 2025, 13:45–15:15

Aim:

To analyse the epidemiological and clinical characteristics of adult ICU burn patients to guide targeted prevention and intervention strategies.

Methods:

A retrospective service evaluation was conducted on adult burn patients admitted to a UK Regional Burns Intensive Care Unit (ICU) between 2011 and 2022. Data was extracted from electronic patient records, including epidemiological factors (age, sex) and clinical characteristics (% TBSA, inhalational injury, facial injury, burn type, locality, intentional injury, and mortality). Descriptive statistical analysis was performed using Microsoft Excel data analysis tool pack.

Results:

A total of 123 patients were included (mean age: 45 years, 74% were male). The mean%TBSA was 32.5%, with 30.1% presenting with inhalational burns and 65.9% sustaining facial burns. The ICU burn population had a mortality rate of 28.0%. Flame burns, primarily from house fires, were the leading cause (53.7%), while intentional burns (self-harm or suicide) accounted for 16.3% of cases.

Conclusions:

Flame burns from house fires and intentional self-inflicted burns are major contributors to ICU admissions. These findings reinforce the need for enhanced burn prevention efforts through public education and targeted safety measures. The high prevalence of intentional burns highlights the critical need for improved mental health screening, early intervention, and sustained psychiatric support both pre- and post-burn to reduce harm and prevent readmissions. Further research is warranted to explore effective interventions for high-risk populations.

*O13.3 Developing a streamlined machine learning model for sepsis prediction in burn patients using multicenter data from the german burn registry*

**Drysch, M.** ^1^, Reinkemeier, F. ^1^, Puscz, F. ^1^, Hinzmann, J. ^1^, Lehnhardt, M. ^1^, Wallner, C. ^1^, Schmidt, S. ^1^

^1^ BG UK Bergmannsheil Bochum, Bochum, Germany

Oral presentations 13—Critical Care and Anaesthesia, 5 September 2025, 13:45–15:15

Aim:

To develop a clinically practical machine learning model for early sepsis prediction in burn patients using multicenter registry data.

Methods:

We conducted a retrospective cohort analysis of 6804 burn patients from 11 centers in the German Burn Registry (2015–2023). Sepsis was defined based on registry documentation from specialized burn ICUs. Feature selection using LASSO, ElasticNet, RFE, and RFECV identified six admission-level predictors: age, deep partial-thickness burns, full-thickness burns, burned body surface area, inhalation injury, and hypertension. Four machine learning models (Random Forest, Logistic Regression, XGBoost, LightGBM) were trained using an 80/20 split with stratified cross-validation. Model performance was evaluated using AUROC, sensitivity, specificity, and negative predictive value (NPV). SHAP analysis was employed for interpretability.

Results:

The Random Forest model achieved the highest predictive performance (AUROC 0.922, sensitivity 0.907, specificity 0.826, NPV > 0.98). SHAP analysis highlighted burned body surface area, full-thickness burns, and age as dominant predictors. The model’s streamlined input requirements distinguish it from general ICU sepsis scores, facilitating early risk stratification without the need for real-time physiological data.

Conclusions:

This streamlined machine learning model allows accurate early prediction of sepsis in burn patients using only six admission features. Its implementation may enhance antibiotic stewardship, inform wound care decisions, and optimize monitoring strategies. External validation across international burn centers is recommended to confirm generalizability and support clinical adoption.

*O13.4 Clinical outcomes and risk factors in critically ill burn patients diagnosed with Acute Kidney Injury*

Paraskevopoulou, S. ^1^, Hadzitolias, V. ^1^, Emmanouilidis, A. ^1^, Papaioannou, M. ^1^, Tzimorota, Z. ^1^, Pitsiou, G. ^2^, Papadopoulou, S. ^1^, **Lavrentieva, A.** ^1^

^1^ Papanikolaou Hospital, Burn ICU, Thessaloniki, Greece, ^2^ Papanikolaou Hospital, Pulmonary Department, ICU, Thessaloniki, Greece

Oral presentations 13—Critical Care and Anaesthesia, 5 September 2025, 13:45–15:15

Objective:

This study aims to evaluate the risk factors and clinical outcomes in critically ill burn patients diagnosed with Acute Kidney Injury (AKI).

Methods:

Data from all patients admitted to a burn ICU between 2022 and 2024 were analyzed. AKI was diagnosed based on the KDIGO criteria. The severity of burn injury, comorbidities, illness severity, presence of inhalation injury, septic complications, and vasopressor use were assessed.

Results:

A total of 43 burn patients diagnosed with AKI were included in the statistical analysis. The baseline characteristics of the study population were as follows: the mean age was 63.86 years (standard deviation [SD] 15.88), with a mean APACHE II score of 12.02 (SD 5.1), a mean Sequential Organ Failure Assessment (SOFA) score of 5.14 (SD 2.38), and a mean Charlson Comorbidity Index of 2.84 (SD 2.23). Patients diagnosed with AKI had a high mean ABSI score (9.51 [SD 1.87]). Inhalation injury was present in approximately half of the cohort (48.84%).

Most patients with AKI were considered septic. The majority of AKI patients experienced hemodynamic instability, necessitating high doses of vasopressors. Mortality rates among patients diagnosed with AKI stage I were 50%, while mortality was higher in patients with AKI stages II and III, at 71.43% and 66.67%, respectively.

Conclusions:

The diagnosis of AKI was associated with a high burden of illness at admission and a high mortality rate. The majority of AKI patients experienced septic complications and required significant vasopressor support.

*O13.5 Early-Onset Tracheostomy Does Not Facilitate Weaning in Patients with Severe Burn Injury: A Single-Center Study*

Thielmann, J. ^1^, Heitzmann, W. ^1^, Ried, M. ^2^, Akkan, J. ^1^, Fuchs, P. ^1^, Schiefer, J. ^1^, **Markowiak, T.** ^1,3^

^1^ Clinic of Plastic, Reconstructive, Hand and Burn Surgery, Hospital Cologne Merheim, University of Witten-Herdecke, Germany, Cologne, Germany, ^2^ Department of Thoracic Surgery, University Medical Center Regensburg, Regensburg, Germany, ^3^ Correspondence: MarkowiakT@kliniken-koeln.de.

Oral presentations 13—Critical Care and Anaesthesia, 5 September 2025, 13:45–15:15

Aim:

This study investigates the influence of early tracheostomy (ET; <7 days after injury) compared to late tracheostomy (LT; ≥7 days) on the weaning of severely burned patients.

Methods:

A retrospective analysis of 67 patients who underwent tracheostomy between January 2015 and June 2024 due to a burn of at least 15% of the body surface area was performed. Patients were divided into two groups according to the time of tracheostomy: ET (*n* = 42) and LT (*n* = 25). The primary endpoint of the study was the influence of tracheotomy timing on the time to reach milestones in the weaning process (CPAP ventilation, spontaneous respiration without mechanical ventilation, decannulation) and hospital mortality.

Results:

The patients had a mean age of 52.9 years (ET: 49 ± 19.7, LT: 59.7 ± 19.6). With regard to the weaning process, no significant differences were observed in the time to spontaneous respiration without mechanical ventilation (ET: median 51 days, LT: 38 days, *p* = 0.966) or in the time to decannulation (ET: 118 days, LT: 100 days, *p* = 0.770). However, patients in the ET group reached CPAP mode significantly earlier (ET: 19 days, LT: 38 days, *p* < 0.001). The overall hospital mortality rate was 43.3%

Conclusions:

This study did not demonstrate a substantial benefit of early tracheostomy in patients with severe burn injuries. Although ET led to earlier progression to CPAP mode, it did not accelerate overall weaning success or reduce the time to tracheostoma closure. These findings underscore the need for individualized approaches when considering tracheostomy timing in burn patients.

*O13.6 Burn injuries in patients with epilepsy*

Fredj, H. ^1^, Ben Messoud, S. ^1^, Gasri, B. ^1^, Messadi, A. ^1^, **Mokline, A.** ^1^

^1^ Trauma And Burn Center, Tunis, Tunisia

Oral presentations 13—Critical Care and Anaesthesia, 5 September 2025, 13:45–15:15

Introduction: Epileptic patients are at increased risk of severe burns due to unpredictable seizures. Aim of this study was to describe epidemiological, clinical and evolutionary characteristics of burns occuring in epileptic patients.

Methods:

A descriptive, retrospective study was conducted in the intensive burn care unit in Tunisia over a 10-year period (2014–2024). Epileptic patients admitted for burns occurring during seizures were included. Demographic, clinical, paraclinical, and outcome data were collected.

Results:

During the study period, 3391 patients were admitted, among which 35 patients were included (1%). Mean age of patients was 37 ± 16 years, with a sex-ratio of 0.8.Tonic-clonic seizures were observed in 71% of cases (*n* = 25) and absence seizure in 8% of cases (*n* = 3). 54% of patients (*n* = 19) had sub-therapeutic levels of antiepileptic drugs. Burns were thermal in 95% of cases and occurred in a domestic setting in 81% of cases, often involving a brazier (35%) or hot cooking water (25%). TBSA was 13 ± 9%. Burns affect mainly extremities: lower limbs in 34% of cases and upper limbs in 26% of cases, and are deep in 77% of cases. Excision of necrotic tissues was done in 17 cases and skin grafting in 9 cases. Length of ICU stay was 11 days [6–19 days]. Five patients (14%) died due to sepsis.Conclusions: Epilepsy was associated to burn injuries in 1% of cases, and closely linked to undertreated and inappropriately treated patients. So, it is important to identify epileptic patients at high-risk in terms of taking preventive measures and providing specific treatment.

*O13.7 SInhale: Validation of a novel scoring system for burn inhalation injury*

**Moran, H.** ^1^, Concannon, E. ^1^, Stallwood-Hall, C. ^1^, Shanks, L. ^1^, Zhen, E. ^2^, Solanki, N. ^1^, Wagstaff, M. ^1^

^1^ Burns Unit Royal Adelaide Hospital, Adelaide, Australia, ^2^ Burns Service Fiona Stanley Hospital, Perth, Australia

Oral presentations 13—Critical Care and Anaesthesia, 5 September 2025, 13:45–15:15

Aim:

To develop a novel scoring system to standardise description of airway changes in burn inhalation injury and stratify by severity of injury and to assess its utility in prognosticating patient outcomes following burn inhalation injury

Methods:

A retrospective study of consecutive patients with burn inhalation injury was conducted at Royal Adelaide Hospital over a three year period. A scoring system for airway assessment reported by co-authors at the Fiona Stanley Hospital, Perth, the SInhale Classification System, was prospectively developed and retrospectively applied to this burn patient cohort.

Results:

Class III injury was significantly associated with prolonged intubation (>24 h), increased mechanical ventilation duration, higher tracheostomy rates, and longer ICU and hospital stays compared to Class I (mild). Class II injury was linked to extubation failure. Full-thickness head and neck burns and soot in the oropharyngeal/nasopharyngeal regions were the only statistically significant clinical variables correlated with severe (Class II and III) injuries.

Conclusions:

The SInhale Classification System is a useful tool providing a standardised approach for airway assessment in burn inhalation injury. External and prospective multi-centre studies are underway in Australia and New Zealand as part of a burn inhalation injury special interest group established to further validate these findings.

*O13.8 Vitamin D deficiency in burn patients: is supplementation necessary?*

**Skunca, A.** ^1^, Zagorac, D. ^1^, Sabo, G. ^1^, Vinceljek, B. ^1^, Trogrlic, B. ^1^

^1^ Sestre Milosrdnice University Hospital Center, Zagreb, Croatia

Oral presentations 13—Critical Care and Anaesthesia, 5 September 2025, 13:45–15:15

Aim:

This retrospective study aimed to evaluate vitamin D levels within the first two weeks following injury in burn patients admitted to the Burn Intensive Care Unit (BICU) at the Sestre Milosrdnice University Hospital Center.

Methods:

A retrospective analysis was conducted on 50 patients, selected from a total of 130 patients admitted to the BICU over the past five years, whose vitamin D levels were measured within the first two weeks post-injury. The goal was to determine the frequency of vitamin D deficiency in this patient population.

Results:

The median age of the patients was 52.8 ± 19.1 years, with 81.7% male and 18.3% female. The mean total body surface area burned was 43.4 ± 21.8%, and the mortality rate was 50.0%. The median vitamin D level was 22.5 nmol/L (IQR 14–65) within the first 14 days post-injury. The majority of patients (94.0%) had vitamin D deficiency, with levels below 50 nmol/L, and 64.0% of patients had critically low levels below 30 nmol/L.

Conclusions:

Vitamin D deficiency is common in both the general population and critically ill patients, affecting up to 76% of those critically ill. Although literature on vitamin D in burn patients is limited, studies show that levels decrease following burn injuries and can remain low for up to one year. Vitamin D deficiency is often overlooked in acute critical illness, and its full consequences are not well understood. Our findings highlight the high prevalence of deficiency in burn patients and suggest that supplementation may be required.

*O13.9 Neuropsychological sequelae following electrical injury: Should we look for it?*

Fredj, H. ^1^, Chaabani, M. ^1^, Aloui, A. ^1^, Alouini, A. ^1^, Gasri, B. ^1^, Jami, I. ^1^, Messadi, A. ^1^, **Mokline, A.** ^1^

^1^ Burn Intensive Care Unit, Traumatology And Burn Center, Ben Arous, Tunisia

Oral presentations 13—Critical Care and Anaesthesia, 5 September 2025, 13:45–15:15

Introduction: Neurological involvement following electrical burns is not uncommon and is various. It may be either immediate or delayed, and transient or permanent. This may lead to impaired neuropsychological functions, and long term sequelae.

The aim of this study was to determine neuropsychological sequelae following electrical injury.

Methods:

A retrospective descriptive study was conducted in intensive burn care department in Tunisia over a period of 5 years, from 1 January 2018 to 31 December 2022. Patients’ victims of electrical burns requiring hospitalization were included. The survival patients were contacted post-discharge to collect data on neuropsychological disturbances.

Results:

During the study period, 2124 patients were admitted, 223 patients were victims of electrical burns (10.5%). The mean age of patients was 35 ± 15 years, with a sex ratio of 26.8. TBSA was 16.6 ± 15.4%. It was mainly a high voltage electrical injury (83.9%). Most electrical burns are work-related (58.7%). Mortality was 16,1% (*n* = 36). Most survivors (82.9%; *n* = 155) were followed up after discharge. Neuropsychological sequels were: complex regional pain syndrome (28.9%; *n* = 54), improved with analgesics in most cases (60%), anxiety (24%; *n* = 45), a post-traumatic stress disorder related to electrical currents (18%; *n* = 34); and sleep disorders (14.4%; *n* = 27).A third of the survivors (33.2%, *n* = 62) returned to work: 53% of patients undergoing professional retraining and 46.8% keeping their position.

Conclusions:

Neuropsychological sequelae were observed in 10.5% following electrical injury, leading to problems for both patients and health care workers. So, post discharge follows up of these patients and after care support are necessary.

*O14.1 Enhancing Nursing Excellence: The Impact of International Training Programs*

Gonzalez Estrella, S. ^1^, Gonzalez Fernandez, R. ^1^

^1^ Hospital Universitario La Paz, Madrid, Spain

Oral presentations 14, 5 September 2025, 13:45–15:15

Aim:

The aim of this study is to analyze the impact of international training programs on the professional development and clinical expertise of nursing professionals

Methods:

The methodology consists of an observational study conducted through the internship of a nurse from the Burn Unit of La Paz University Hospital (Madrid) at the Burn Unit of CHUV (Lausanne).

Results:

The international training experience significantly enhanced the nurse’s clinical skills, particularly in advanced burn care techniques and patient management. Exposure to different protocols and interdisciplinary teamwork in the Lausanne University Hospital provided valuable insights for improving practices in the Burn Unit of La Paz University Hospital. The exchange fostered professional growth, cultural competence, and a broader perspective on evidence-based nursing care in burn treatment. Strong connections were established between both institutions, facilitating ongoing knowledge exchange and collaboration. The study highlighted the crucial role of nursing leadership in driving innovation and improving patient care through international learning opportunities.

Conclusions:

The international training experience proved to be highly beneficial for nursing professional development, enhancing clinical expertise in burn care and fostering a more comprehensive, evidence-based approach. The exchange between the two burn units not only improved technical skills but also strengthened collaboration between institutions, promoting the continuous sharing of knowledge and best practices. Additionally, this study underscores the importance of nursing leadership in driving innovation and improving patient outcomes through international learning opportunities. Investing in these programs is essential to advancing nursing education, optimizing patient care, and strengthening global healthcare networks.

*O14.2 The journey of a patient with Toxic Epidermal Necrolysis; preliminary results from a qualitative study*

**Hofland, H.** ^1,3^, Heijblom-van Dinteren, M. ^1,3^, Trommel, N. ^1,3^, de Jong, A. ^2,3^

^1^ Burn Center Rotterdam Maasstad Hospital, Rotterdam, Netherlands, ^2^ Burn Center Beverwijk, Red Cross Hospital, Beverwijk, Netherlands, ^3^ Alliance of Dutch Burn Care, Brandwondenzorg Nederland, The Netherlands.

Oral presentations 14, 5 September 2025, 13:45–15:15

Aim:

The aim of this study was to get an in-depth encompassing account of experiences during the journey of patients living with, and the aftermath of TEN.

Introduction

Toxic Epidermal Necrolysis (TEN) is a rare disease, with severe cutaneous adverse reactions, due to a variety of medications. Because of high mortality and morbidity this disease may have a significant impact on the Health Related Quality of Life (HRQoL). As yet, the experiences of patients during the onset of the skin reactions, admission in burn centers and coping with the aftermath at home is highly under-researched.

Methods:

This study used a qualitative method. Patients with TEN admitted in one of the three burn centers in The Netherlands between 2009 and 2022 were asked to participate. Interviews were voice recorded and verbatim transcribed. Open and axial coding was used to identify themes of relevance of the experienced problems.

Results:

Twelve patients participated. Ten women and two men (mean age 51, range 20–84) were interviewed on average eight years (range 3–15) after being diagnosed with TEN. The interviews confirmed that a huge variety of problems did occur. Several themes on physical, psychological and social level have been identified that affected respondents during rehabilitation after the disease.

Conclusions:

The preliminary results of this study demonstrates that TEN has a huge impact on HRQoL and causes many problems once recuperating at home. Furthermore, there is room for improvement in preparation to discharge from the burn centre. Patients’ ideas how to improve this will be discussed.

*O14.3 Use of Burns Podcasts as part of Burns Education*

**Lee, N.** ^1^

^1^ London And South East Burns Network, London, United Kingdom

Oral presentations 14, 5 September 2025, 13:45–15:15

Aim—Introduction of digital audio educational Podcasts as part of burns education in a online academic Advanced Burns Module. With a hope to expand educationally resources catering for audio learning styles while using a online educational platform.

Methods—Creation of different Podcasts as resources for students to access during there module learning and reviewing feedback about there use. Current resource are on a range of topics set out over the burns patients journey through care.

- Pre hospital firefighter and Helicopter medic accounts.
- Case studies on unusual cases one example Elemental metal burn.
- Escharotomy.
- Patient Experience.

Results—Feedback from creators was it was fun and easy to create the Podcast. Feedback from students were 100% enjoyment of listening to them, they enjoyed this style of learning and 90% of students remembered key learning points as part of MCQ test results at the end of everyday. Feedback from students on module were they wanted more of this style of learning during the course

Conclusions—Use of audio Podcasts during burns education is well received and enjoyable to students, creating a different style of education to student and MDT. Team development of increased resources is underway looking at expanding to wider audiences as part of wider burns education within the London and South East Burns Network.

*014.4 Nutritional practice in patients with burns*

**Niemeijer, A.** ^1^, Bosch, F. ^1,2^, Lantman-Gommers, M. ^3^, Koekkoek, K. ^4^, Nieuwenhuis, M. ^1,2^

^1^ Alliance of Dutch Burn Care (ADBC), Burn Centre Martini Hospital, Groningen, The Netherlands, ^2^ Research group Healthy Ageing, Allied Health Care and Nursing, Hanze University of Applied Sciences, Groningen, The Netherlands, ^3^ Department of Dietetics, Martini Hospital, Groningen, The Netherlands, ^4^ Department Intensive Care, Martini Hospital, Groningen, The Netherlands.

Oral presentations 14, 5 September 2025, 13:45–15:15

Aim:

To describe adherence to nutritional targets in daily practice for patients with burns and to determine whether characteristics related to patient, burns, or treatment are associated with achievement of nutritional goals.

Methods:

Eligible for this longitudinal retrospective study were patients admitted with acute burns between April 2020 and February 2022, who were 18 years or older, with a length of stay of 72 h or more. We examined the amount of energy (kcal) and proteins (grams) which was prescribed based on formulas, and the amounts provided by nutrition assistants and nurses. The difference was converted into percentages, and values between 90% and 110% interpreted as good adherence.

Results:

In total, the records of 129 patients were included, of whom 92 (71.3%) were male, and 24 (18.6%) required burn intensive care (IC). These patients accounted for 3200 patient days, of which 497 (15.5%) were burn IC days. The mean provision of energy was 98% (range: 1–225%), for protein 97% (range: 0–196%). Preliminary analyses showed that good adherence was associated with a higher TBSA at admission (*p* < 0.001) and negatively affected by surgery on the day of surgery (*p* < 0.001).

Conclusions:

In practice, the mean amount of energy and proteins provided matches the prescribed amount. However, on many days, the amounts did not align, and patients were under- or overfed. Further associations will be examined to gain insights necessary to combat muscle loss and disease-related malnutrition.

*O14.5 Surgical management of ukrainian victims of war—experiences from a burn center*

**Moshammer, M.** ^1,2^, Smolle, C. ^2^, Geißler, J. ^2^, Mayrhofer, M. ^2^, Bauer, B. ^2^, Kohlhauser, M. ^2^, Carnieletto, M. ^2^, Spendel, S. ^2^, Girsch, W. ^2^, Kamolz, L. ^1,2^

^1^ COREMED—Center for Regenerative and Precision Medicine, JOANNEUM RESEARCH Forschungsgesellschaft mbH, Graz, Austria, ^2^ Division of Plastic, Aesthetic and Reconstructive Surgery, Department of Surgery, Medical University of Graz, Graz, Austria

Oral presentations 14, 5 September 2025, 13:45–15:15

Aim:

Since the onset of the Russo-Ukrainian War, wounded Ukrainian patients have been frequently transferred to European hospitals. This study provides an overview of the injury patterns and treatment approaches for war victims treated in Graz.

Methods:

All Ukrainian war victims admitted to our department between 1 March 2022, and 31 October 2024, were included in this study. Collected data included patient demographics (age, gender), injury mechanism, extent and type of injuries, wound microbiology at admission, and key treatment parameters.

Results:

A total of 13 war victims (9 women, 4 men) with a median age of 35 years were treated. The most common injuries included burns (7 cases, affecting 5–50% TBSA), fractures (9 patients with at least one fracture), nerve injuries (5 cases), extensive soft tissue defects (6 cases), and amputations (4 cases). Injuries predominantly affected the upper body, with the upper limbs involved in 11 patients and the face in 9. Multidrug-resistant organisms were frequently detected. Patients underwent a mean of 4.6 surgical procedures before discharge, with a median hospital stay of 68.7 days (range: 5–130). Eight patients required skin grafting, four had free flaps (two fibula, one serratus, and one radial forearm flap), and two received non-vascularized bone grafts.

Discussion: In this patient collective, war injuries resulted in complex and highly heterogeneous trauma. Despite varying injury patterns, victims exhibited similar bacterial colonization, with a high prevalence of multidrug-resistant organisms. Experience has shown that, even after good initial care, further treatment remains highly demanding.

*O14.6 Implementation of a Ward Round Pro Forma for Burns Patients*

**Rapier, J.** ^1^, Bennett, S. ^2^, Barnes, D. ^2^

^1^ Salisbury District Hospital, Salisbury, United Kingdom, ^2^ St Andrew’s Centre for Burns and Plastic Surgery, Chelmsford, United Kingdom

Oral presentations 14, 5 September 2025, 13:45–15:15

Aim:

St Andrew’s Centre for Plastic Surgery & Burns implemented ward round (WR) pro forma sheets to help capture important patient data (in line with national burn care standards and local best practices). The pro formas aimed to act as an aide memoire for junior doctors, make WRs more time efficient, and make documentation more legible. This audit was conducted to improve WR documentation for admitted burns injury patients and thereby facilitate the meeting of relevant clinical practice guidelines.

Method:

WR proforma sheets were distributed and then reviewed retrospectively for completeness and timely filing in the medical notes for patients admitted to the adult and paediatric burns wards. The first cycle included patients admitted from 16–29 December 2019. The results were disseminated to the team, an improved pro forma sheet was introduced and the second cycle was completed including patients admitted from 22 February to 18 March 2020.

Results:

Significant improvements were seen in the completion of 97% of WR proforma sections. The timely filing of WR pro forma sheets remained at between 99–100% across both cycles.

Conclusions:

Overall improved WR pro forma sheet completion and filing was observed between the audit cycles following dissemination of the results to the team and introduction of an improved pro forma sheet, thereby facilitating adherence to national and local clinical practice guidelines and ultimately improving patient care in this vulnerable cohort. We recommend that a similar proforma is adopted at other burns sites to improve data collection and patient care.

*O14.7 Crisis-ready by design: resilience and resource flexibility in a monospecialist burn center during mining disasters in Poland*

**Smętek, W.** ^1^, Rybka, M. ^1^, Nowak, M. ^1^

^1^ Stanislaw Sakiel Burn Treatment Center in Siemianowice Slaskie, Siemianowice Śląskie, Poland

Oral presentations 14, 5 September 2025, 13:45–15:15

Aim:

To analyze the strategic and operational flexibility of a monospecialist burn center during mining-related mass-casualty events.

Methods:

A retrospective case study was conducted at the Centre for Burn Treatment (CLO) in Siemianowice Śląskie, Poland, evaluating institutional responses to two methane explosions in coal mines (Knurów-Szczygłowice and Pniówek), involving 35 burn patients. The study includes analysis of protocols, infrastructure, digital coordination tools, and the application of management methodologies: process management, Lean Healthcare, Theory of Constraints (TOC), and Resource-Based View (RBV).

Results:

Following the Knurów-Szczygłowice incident, 10 patients were admitted (5–80% TBSA, II–III°), with 7 ICU cases. The Pniówek explosion resulted in 25 admissions (15–90% TBSA, II–IV°), with 6 ICU cases. CLO ensured continuity of care by dynamically scaling ICU capacity, optimizing patient placement via a centralized coordination system, and rapidly reallocating clinical teams. Strategic use of tissue banks and cell cultures, suspension of planned admissions, and real-time inventory control and bed-tracking supported system resilience. Management tools such as TOC and RBV enabled resource prioritization, while Lean principles enhanced staff task segmentation and patient flow during the crisis.

Conclusions:

A structured management approach integrating Lean, TOC, and RBV principles—combined with centralized coordination—allowed the burn center to maintain full operational capacity during large-scale emergencies. This model offers practical insights for other monospecialist centers aiming to strengthen preparedness and systemic resilience.

Keywords: mass casualty burns, lean Healthcare, burn center resilience

*O14.8 Cost-effectiveness in Value-Based Health Care: Development of the Burn Care Multi-Criteria Decision Analysis (BC-MCDA) model*

Thambithurai, R. ^1,2^, van Uden, D. ^1^, van der Vlegel, M. ^1^, Verwilligen, R. ^3^, van Zuijlen, P. ^3,4,5^, Nieuwenhuis, M. ^6,7,8^, van der Vlies, C. ^1,9,10^, Weel-Koenders, A. ^2,11^, Hackert, M. ^2,12^, **van Baar, M.** ^1,13^

^1^ Alliance of Dutch Burn Care (ADBC), Burn Centre, Maasstad Hospital, Rotterdam, The Netherlands, ^2^ Erasmus School of Health Policy and Management, Erasmus University Rotterdam, Rotterdam, The Netherlands, ^3^ Alliance of Dutch Burn Care (ADBC), Burn Centre, Red Cross Hospital, Beverwijk, The Netherlands, ^4^ Amsterdam UMC location Vrije Universiteit Amsterdam, Department of Plastic, Reconstructive and Hand Surgery, Amsterdam, The Netherlands, ^5^ Amsterdam Movement Sciences (AMS), Tissue Function and Regeneration, Amsterdam, The Netherlands, ^6^ Alliance of Dutch Burn Care (ADBC), Burn Centre, Martini Hospital, Groningen, The Netherlands, ^7^ Hanze University of Applied Sciences, Research group Healthy Ageing, Allied Health Care and Nursing, Groningen, The Netherlands, ^8^ University of Groningen, University Medical Center Groningen, Department for Human Movement Sciences, Groningen, The Netherlands, ^9^ Departments of Trauma and Burn Surgery, Maasstad Hospital, Rotterdam, The Netherlands, ^10^ Trauma Research Unit Department of Surgery, Erasmus MC, University Medical Centre Rotterdam, Rotterdam, The Netherlands, ^11^ Department of Rheumatology, Maasstad Hospital, Rotterdam, The Netherlands, ^12^ Department of Quality and Control, Maasstad Hospital, Rotterdam, The Netherlands, ^13^ Department of Public Health, Erasmus MC, University Medical Centre Rotterdam, Rotterdam, The Netherlands

Oral presentations 14, 5 September 2025, 13:45–15:15

Aim:

Effects of care are ideally assessed using a combination of patient-relevant outcomes. These can be integrated through a Multi-Criteria Decision Analysis (MCDA) framework. To support economic evaluation from a value-based healthcare (VBHC) perspective, we aim to develop an MCDA framework for burn care (BC-MCDA) and validate it using real-world data.

Methods:

Following guidelines, a BC-MCDA model was developed and validated with stakeholders (patients, clinicians and managers) from the three Dutch burn centres through interviews and focus groups (*n* = 14).

The VBHC burns core outcome set was used. Changes in each outcome were valued, resulting in scores. The importance of each outcome, compared to others was assessed, resulting in weights per outcome. The BC-MCDA model was validated using a cohort of adult patients receiving specialised burn care with a 12-month follow-up, during a 15-month period. Data from the Dutch Burn Repository R3 and the Burn Centres Outcomes Registry The Netherlands was used to compare BC-MCDA values and costs in patients with mild to moderate burns versus severe burns. Costs include direct medical costs from the start of treatment.

Results:

The BC-MCDA model consists of nine outcomes ranging from pain and itching to return to school/work. Scores and weights were established for 3 and 12 months post-discharge.

Validation of the model showed that, as expected, patients with severe burns showed less favourable outcomes, reflected in lower MCDA values against higher costs.

Conclusions:

The BC-MCDA model is available and can be used to assess the VBHC-cost-effectiveness of treatments throughout the patient journey.

*O14.9 Impact of Post-traumatic Stress Disorder (PTSD) on Long-Term Rehabilitation Outcomes in Burn Injury Patients*

**Klimitz, F.** ^1^, Aman, M. ^3^, Neubauer, H. ^1^, Stolle, A. ^1^, Hundeshagen, G. ^1^, Ziegenthaler, H. ^4^, Kneser U, ^1^, Harhaus L, ^3^

^1^ Department of Hand, Plastic and Reconstructive Surgery, Burn Center, BG Trauma Center Ludwigshafen, Plastic- and Hand Surgery, University of Heidelberg, Ludwigshafen, Germany, ^2^ Division of Plastic Surgery, Department of Surgery, Yale School of Medicine, New Haven, USA, ^3^ Department of Hand, Replantation, and Microsurgery, BG Trauma Center Berlin, Chair of Hand, Replantation, and Microsurgery, Charité—Universitätsmedizin Berlin, Berlin, Germany, ^4^ Graefliche Kliniken—Moritz Klinik GmbH, Bad Klosterlausnitz, Germany

Oral presentations 14, 5 September 2025, 13:45–15:15

Aim:

To investigate the influence of post-traumatic stress disorder (PTSD) on long-term physical and psychosocial rehabilitation outcomes in burn injury patients.

Methods:

This prospective cohort study included burn survivors treated at the Burn Center Ludwigshafen and the Rehabilitation Center of Moritz Klinik, Bad Klosterlausnitz (Germany) as part of the project “Evaluation of an ICF-based Rehabilitation Concept for Thermal Injuries.” Patients were stratified into two groups: with PTSD and without PTSD. Longitudinal data were collected at the beginning of inpatient rehabilitation and at discharge as well as at follow-up intervals of 3- and 12-months post-discharge, including physical performance measures and validated self-report instruments. Statistical comparisons were performed using non-parametric Kruskal-Wallis tests.

Results:

A total of *n* = 92 were included in the analysis. Patients with PTSD (*n* = 38) showed significantly worse outcomes in multiple domains. Pain levels—both at rest and during physical activity—were consistently higher in the PTSD group. In particular, itching and joint pain were notably higher in PTSD patients both at the beginning of inpatient rehabilitation and 12-months post-discharge. Furthermore, PTSD patients exhibited delayed improvements in skin condition including dryness, vulnerability, elasticity, and sensitivity to heat or cold. These findings indicate a broad and sustained negative impact of PTSD on recovery after thermal trauma.

Conclusions:

PTSD substantially impairs physical rehabilitation outcomes in burn patients. These results emphasize the need for early identification and targeted treatment of PTSD in burn survivors, suggesting that integrated psychological support should be a central component of post-burn rehabilitation protocols.

*O14.10 Thank you Europe!—Triage and early assessment of intoxication levels and burn surface*

**Todorovska Shapova, B.** ^1^

^1^ Biljana Todorovska Shapova, Skopje, North Macedonia, ^2^ Elizabeta Obocki Lukovska, Skopje, North Macedonia, ^3^ Mare Stevkovska, Skopje, North Macedonia, ^4^ Emilija Atanasova, Skopje, North Macedonia, ^5^ Vladislav Gruev, Skopje, North Macedonia.

Oral presentations 14, 5 September 2025, 13:45–15:15

Aim:

The aim of this paper is to present primary assessment and triage of burn and intoxication patients due to a massive disaster.

Method

On the 16th of March 2025, 30 patients were admitted at JZU City Surgical Clinic St” N.Ohridski”-Skopje. The patients were treated with primary assessment which concluded that there is intoxication, smoke inhalation, and burns on the head, neck and upper arms. Primary triage showed that 17 patients needed intensive unit care and immediate operations. Six patients were with TBSA with above 20%, 7 patients with TBSA 10–20% and 4 patients with TBSA less than 10%.

Results:

Five of 17 patients required immediate intubation, two patients had a fasciotomy and additionally on 9 patients we performed escharotomy. Immediate resuscitation was done as well as CVC and UC, and one thoracic drainage. An ophthalmologic assessment showed injury of the sclera and cornea which was treated by artificial tear drops. A fiber laryngoscopy was performed on all of the patients, it was positive and treated. A computed tomography showed inhalation of smoke in the lungs. A toxicological assessment showed disturbances in gas analysis and was treated with oxygen supply. Primary psychological assessment was done on all patients to conclude their mental well being. In our hospital 11 patients remained to be treated and one was transferred to a toxicology clinic for further treatment.

Conclusions:

Due to primary assessment it was concluded that the patients need intensive unit care treatment and were sent abroad.

Keywords: disaster, triage, early assessment

*O15.1 Pediatric burns during seasonal food preparations*

**Abay, A.** ^1^, Bostancı, S. ^2^, Erten, E. ^1^, Ertürk, A. ^2^, Demir, S. ^1^

^1^ Ankara Bilkent City Hospital, Ankara, Turkey, ^2^ Ankara Yildirim Beyazit University, Ankara, Türkiye.

Oral presentations 15—Prevention, 5 September 2025, 13:45–15:15

Aim:

Scald burns are the most common childhood burns, often caused by spills or splashes. In our country, seasonal food (e.g., molasses, jam, tomato paste) preparations involving large cauldrons of boiling high volume thick liquids create a significant burn risk for children. This study aims to analyze the characteristics and outcomes of pediatric seasonal food preparation burnsn and highlight the need for targeted prevention strategies.

Methods:

We retrospectively analyzed pediatric patients treated for scalds in our pediatric burn center over the last five years. Burns were categorized based on the cause (seasonal preparations, hot water, tea, milk, oil, food). Demographic and clinical data were compared in those groups, with *p* < 0.05 considered significant.

Results:

Among 910 patients with hot liquid burns, 33 resulted from seasonal preparations. The majority of these burns occurred in the autumn (48.4%), particularly in September (42%). Seasonal preparation burns had a significantly higher mean total burn surface area (29.6%, *p* < 0.001), higher grafting rate (51.5%, *p* < 0.001), and a longer hospital stay (32.1 ± 26 days, *p* < 0.001). Two patients in seasonal preparation group died from sepsis and multiorgan failure, with a mortality rate similar to other groups (*p* = 0.778).

Conclusions:

While seasonal food preparation is culturally significant, it poses one of the most severe scald injury risks for children, often leading to large burn surface areas, prolonged hospitalizations, and high grafting rates. Urgent public awareness campaigns through television and social media, especially during peak preparation periods, are essential to prevent these dangerous accidents and protect children from severe and potentially fatal burns.

*O15.2 Electrical Burn Injuries: An over 15-Year Period Multicenter Retrospective Review of High-Voltage and Low-Voltage Cases*

**Aydogan, C.** ^1^, Erkent, M. ^2^, Abalı, A. ^1^, Albayati, A. ^1^, Gojajev, A. ^2^, Avcı, T. ^3^, Aydogan, H. ^4^, Haberal, M. ^1^

^1^ Baskent University Burn Center, Ankara, Türkiye, ^2^ Baskent University Department of General Surgery, Ankara, Türkiye, ^3^ Baskent University Turgut Noyan Trainig and Research Center, General Surgery, Adana, Adana, Türkiye, ^4^ Baskent University School of Medicine, Ankara, Türkiye.

Oral presentations 15—Prevention, 5 September 2025, 13:45–15:15

Aim:

Electrical burns can be severe due to complex tissue damage. This study aimed to analyze the epidemiology, risk factors, clinical presentation, management, and outcomes of electrical burn patients in multiple Baskent University burn centers.

Methods:

We retrospectively reviewed records of 361 patients with electrical burns from January 2008 to January 2025. Collected data included demographics, burn characteristics (voltage level, total body surface area, depth), clinical variables (CPK, myoglobin), management (fasciotomy, debridement, grafting), complications, and outcomes.

Results:

Among 361 patients, 87% were male, and 76% had high-voltage injuries. Most accidents occurred at workplaces (47%) or near power lines in residential areas (19%). The mean age was 28.9 years, and the mean total body surface area involved was 21%. Average serum CPK and myoglobin levels were 8722.63 U/L and 1924.43 ng/mL, respectively. Common complications included invasive wound infections (67.1%), deep muscle involvement (43.4%), sepsis (34.6%), and ARDS(13.8%). Debridement (62.2%) and skin grafting (54.6%) were the most frequent surgical interventions, with fasciotomy (33.7%), amputation (15.1%), and flaps (14.2%) contributing to limb salvage or definitive closure. Delayed hospital admission (mean 2.5 days) correlated with higher rates of tissue loss. Mortality was 8.92%.

Conclusions:

Electrical burn injuries present unique challenges due to deep tissue damage and associated systemic complications. Prompt resuscitation, early fasciotomy/escharotomy, and comprehensive debridement are critical to minimize amputations and preserve function. Public awareness campaigns, workplace regulations, and safety education can reduce incidence. When injuries occur, specialized burn centers ensure optimal outcomes through timely surgery, aggressive infection control, and multidisciplinary rehabilitation.

*O15.3 Ethnic Disparities in Burn Injuries in the UK: A Systematic Review*

Badakhshan, U. ^1^, Zamani, R. ^1^, Martins, T. ^1^

^1^ University Of Exeter, Exeter, United Kingdom

Oral presentations 15—Prevention, 5 September 2025, 13:45–15:15

Aim:

Burn injuries are among the most physically and emotionally distressing injuries, affecting approximately 250,000 people annually in the UK. While extensive research has explored how gender and socioeconomic factors influence burns, ethnic disparities have received less attention. This study aimed to address this by evaluating potential healthcare barriers affecting ethnic communities in the UK.

Method:

The review followed the PRISMA framework for accessing journal databases and search terms included concepts of ethnicity and burn injuries in the UK. The quality of the studies was assessed with the Critical Appraisal Skills Programme.

Results:

Out of the initial search result of 3339, 11 studies were selected following the eligibility screening. White British made up 52.4% of admissions, whereas Asians and Africans made up 24.7% and 6.46%, respectively. Trends showed a decline in Whites’ admissions and a rise in Africans’ admissions. Children aged 1 to 5 years were most affected, particularly in the Asian and African groups. Males constituted 57.9% of admissions, while females accounted for 42.1%. Most burns occurred at home, with scalds, particularly among children. Ethnic minorities were more likely to apply unsuitable topical treatments. The duration of hospital stay was longer for ethnic minorities, with flame burns leading to longer stays. Ethnic minority patients had higher rates of psychological referrals.

Conclusions:

The review underscores significant ethnic disparities in burn injuries and outcomes, with specific characteristics. Targeting policies to address them could result in a more equitable healthcare system and improved outcomes for burn patients in similar healthcare settings worldwide.

*O15.4 From injury to recovery: analyzing treatment pathways in adult hand burn patients*

**Bartkova, J.** ^1,2^, Petrásšková, L. ^2^, Selecká, D. ^3^, Rucki, J. ^2^, Kotradyová, M. ^2^, Cederna, P. ^4^

^1^ Department of Burns and Plastic Surgery, The University Hospital, Brno, Czech Republic, ^2^ Faculty of Medicine, Masaryk University, Brno, Czech Republic, ^3^ Department of Dermatology, Šumperk Hospital, Šumperk, Czech Republic, ^4^ Department of Surgery, Section of Plastic Surgery, University of Michigan Health System, Ann Arbor, Michigan, USA

Oral presentations 15—Prevention, 5 September 2025, 13:45–15:15

Aim:

This study aimed to assess the demographics, causes, and treatment outcomes of adult hand burn patients to identify risk factors and improve management strategies.

Methods:

A retrospective analysis was conducted on 507 adult patients with hand burns admitted to a specialized burn unit between 2016 and 2022. Data were collected from medical records, including demographics, burn etiology, injury severity, treatment interventions, hospitalization outcomes, and ICU admissions.

Results:

Among 507 patients analyzed from a cohort of 1378 burn patients, 72.78% were male, with a mean age of 45.64 years (IQR 31–59). Burns affected one hand in 54.64% of cases, while 45.36% sustained bilateral injuries. Most patients (59.4%) were from urban areas, with injuries occurring mainly at home (51.6%) and the workplace (19.4%). Transport to the burn center varied, with 35.6% arriving by ambulance, 15% by air, and 21.7% transferred from other hospitals. Flame burns were the most common cause (61.34%). Surgery was required in 53.45% of cases, including escharotomy in 11.05%, and skin grafts in 37%. Chemical necrectomy was performed in 87.03% of cases, while surgical debridement was necessary in 12.97%. Reconstructive surgery for scars and contractures was required in 1.58% of patients. ICU admission was necessary for 35.7%, while 24.5% required immediate surgery. The mean hospital stay was 18.82 days (IQR 7–26).

Conclusions:

The findings emphasize the importance of early specialized care, surgical management, and preventive measures to reduce the impact of hand burns.

*O15.5 The impact of socioeconomic deprivation on first aid adequacy for burn injuries: A retrospective cohort study*

**Kong, M.** ^1^, Baldwin, A. ^2^, Kerstein, R. ^2^

^1^ University of Buckingham, Buckingham, United Kingdom, ^2^ Department of Burns and Plastic Surgeon, Stoke Mandeville Hospital, Aylesbury, United Kingdom.

Oral presentations 15—Prevention, 5 September 2025, 13:45–15:15

Aim:

To examine the relationship between socioeconomic status (Index of Multiple Deprivation IMD) and adequacy of first aid for burn injuries.

Methods:

A retrospective cohort study included all burn patients (paediatric and adult) treated at a regional burn unit over a three-month period (1 May 2024—31 July 2024). Data were extracted from the International Burn Injury Database (IBID). IMD deciles were determined from postcodes. First aid was classified as “adequate” (cooling with water for ≥20 min) or “inadequate” (all other methods), with the latter subcategorised as “damaging” or “not damaging.” Logistic regression analyses assessed the relationship between IMD decile and first aid adequacy, adjusting for age, sex, burn aetiology, size, and depth.

Results:

Among 269 patients (median age 28.3 years, IQR 6.0–49.5; 66.9% adults; 50.9% male), scald injuries were most common (59.5%), followed by contact (24.9%) and flame burns (15.6%). Median IMD decile was 7 (IQR 5–9). Binomial logistic regression indicated IMD decile as a significant predictor of inadequate first aid (OR 0.898, 95% CI 0.807–0.998, *p* = 0.046), with a 10.2% increased likelihood of inadequate first aid per decile decrease. Multinomial regression showed an association between lower IMD deciles and harmful first aid practices (OR 0.836, 95% CI 0.737–0.948, *p* = 0.005).

Conclusions:

Socioeconomic deprivation correlates with inadequate and harmful first aid. Targeted public health interventions in disadvantaged communities are crucial to improving first aid knowledge and adherence to guidelines, potentially reducing burn severity and improving outcomes.

Key words: burn injuries, first aid, socioeconomic status

*O15.6 Five-year-period changes in epidemiology of severe burns—experience from a university tertiary burn center in Serbia*

**Petrovic Popovic, D.** ^1^, Petrović Elbaz, M. ^1^, Novaković, M. ^1^

^1^ Clinic for burns, plastic and reconstructive surgery, University Clinical Center of Serbia, Beograd, Serbia

Oral presentations 15—Prevention, 5 September 2025, 13:45–15:15

Aim:

Aim of this study was to monitor epidemiological changes in severe burns’ incidence, dominant causes, number of ICU days, and patients’ sex, age and mortality in 2011. compared to 2015, when a new antiseptic regimen was implemented based on octenidine-based products for prevention of infection and/or local treatment of infected burns.

Methods:

Following data of a university tertiary burn center were retrospectively analysed for both periods: age, gender, main cause of burn (flames, scalds, electricity…), total body surface area (TBSA), length of stay in ICU and mortality.

Results:

In total 327 patients were identified. We found no significant difference in male/female ratio in those years (64.2/35.8% vs. 63.6/36.4%), age (50.9 ± 19.8 vs. 53.5 ± 19.9 years) and the dominant cause of burn wounds (flame 45.5 vs. 44%, scalds 35 vs. 37%), respectively.

Noteworthy, mortality decreased (2.3 vs 1.8%), despite slight increase in TBSA (17.2 ± 18.7 vs. 18.3 ± 19.1%) and significant increase in length of stay in ICU (17.9 ± 16.5 vs. 21.7 ± 19.6 days). In general, incidence of severe burns decreased by 11% (173 vs. 154 patients).

Conclusions:

In line with published literature in other countries, we also found a steady decrease of severe burns’ incidence in our region whereas dominant causes of severe burns remained the same.

From clinical perspective, octenidine-based antisepsis enables a good wound assessment and represents safe and effective first-line therapy of burns. Its probable effect on reduced mortality and increased patient safety through prevention of subsequent infections should further be evaluated and thoroughly described.

*O15.7 Impact following implementation of Ultraviolet Light (UVC) decontamination system into a regional burn centre with overview of the literature on UVC technology in healthcare.*

**Singh, V.** ^1^, Shibu, S. ^1^, Cappuyns, L. ^1^, Philip, L. ^1^, Gurusinghe, D. ^1^, Shokrollahi, K. ^1^

^1^ Mersey Burn Centre, Liverpool, United Kingdom

Oral presentations 15—Prevention, 5 September 2025, 13:45–15:15

Aim:

A: To evaluate the impact of installing UVC systems in our burn centre

B: To review existing literature on UV decontamination in healthcare and identify future research

Methods:

A structured literature review on UV decontamination in healthcare settings was performed. Additionally, interviews were held with staff and physicians at our burn-care centre to assess the impact of UVC installation on physiotherapy schedules, patient throughput, and recovery.

Results:

The installation of UVC systems in the therapy/gym area improved staff productivity and patient access. Previously, treating a single infective patient rendered the gym unusable for the remainder of the day. Whereas now, multiple patients with infections can be seen consecutively, enhancing rehabilitation opportunities. UVC cleaning also reduced time and cost compared to traditional methods such as fumigation. Interviews suggested improved flexibility and efficiency in care delivery. Literature review of 170 studies revealed that UV irradiation significantly reduces bacterial load and healthcare-associated infections. Emerging UV technologies demonstrate potential for safe use in occupied environments, including surgical site and skin disinfection, with minimal harm to human tissue.

Conclusions:

The use of UVC systems in operating rooms has shown to lead to a 58.5% greater reduction in colony-forming-units compared to traditional chemical cleaning [1]. Utilising this information in addition to our interviews, which show increased efficacy and ease of use, reveals an opportunity for expansion of UV decontamination technology into other critical areas to enhance safety and efficiency, and potentially making UV decontamination a new standard in infection control.

*O15.8 Behavioural and environmental factors related to burn accidents in young children; a qualitative study*

**van Zoonen, E.** ^1^, Soek, A. ^1^, van Baar, M. ^2^, Pijpe, A. ^3^, Nieuwenhuis, M. ^4^, Stoker, K. ^3^, Heijblom, M. ^2^, Bijker, G. ^4^, van Schie, C. ^1^, Meij-de Vries, A. ^3^

^1^ Dutch Burns Foundation, Beverwijk, The Netherlands, ^2^ Maasstad Hospital, Rotterdam, The Netherlands, ^3^ Red Cross Hospital, Beverwijk, The Netherlands, ^4^ Martini Hospital, Groningen, The Netherlands

Oral presentations 15—Prevention, 5 September 2025, 13:45–15:15

Aim:

This follow-up study aimed to identify factors influencing the behaviour and environment of parents in The Netherlands, whose children under the age of 5 were involved in a burn accident and classified as increased risk of burn injury.

Methods:

In this qualitative study, phase 3 of the PRECEDE-PROCEED model was completed. Interviews were carried out until saturation of topics was reached. Eligible were parents of children who were treated in a Dutch burn centre at the age of 0–4 years and who were identified in a previous study as a high-risk group, that is; when both parents were born outside The Netherlands, they lived in a low SES neighbourhood or they had two or more siblings living at home. Interviews were transcribed and coded.

Results:

A total of 27 interview were conducted. Important predisposing factors were a lack of knowledge and risk awareness, underestimated abilities of the child and a knowledge driven adequate response. Important enabling factors were distraction, stress and fatigue, difficulties in securing the environment, poor Dutch language skills, an unfamiliar environment, permissive parenting style and an outgoing character of the child. Reinforcing factors included thresholds in addressing others on unsafe behaviour and a lack of support.

Conclusions:

The outcomes of this study provided a deeper understanding of the aetiology in burn accidents in children under 5 years of age in The Netherlands. The identified factors will be used to design effective prevention programs for the population at risk.

*O15.9 Dermal substitutes as a carrier for mechanical SVF*

**Kim, B.** ^1^, Reid, G. ^1^

^1^ University Hospital Zürich, Zürich, Switzerland

Oral presentations 15—Prevention, 5 September 2025, 13:45–15:15

Aim:

Contemporary management of full-thickness burn defects frequently incorporates dermal substitutes, with clinicians selecting from a wide array of commercially available options that differ in composition, structure, and biological properties. Cell-based therapies, particularly those utilizing adipose tissue-derived progenitor cells, have emerged as promising adjuncts recently. Mechanical isolation methods, such as mechanical stromal vascular fraction (mSVF), offer a practical, regulatory-compliant approach for obtaining regenerative cells. This study evaluated the feasibility of seeding mSVF onto various commercially available dermal substitutes in vitro to their biocompatibility.

Methods:

Mechanical SVF was isolated from human donors obtained during elective surgeries using a standardized protocol involving emulsification and centrifugation steps. The mSVF was then seeded onto multiple commercial dermal substitutes under standard culture conditions. Supernatants were analyzed for total protein content and growth factor concentrations. Cellular integration was assessed via histological examination and electron microscopy.

Results:

Significant variability was observed in mSVF ingrowth among the tested dermal substitutes. Rigid substitutes with dense surfaces exhibited poor cellular infiltration, while others with a greater pore size fostered integration within 1–2 weeks. Biological dermal substitutes demonstrated higher protein and growth factor levels in supernatants.

Conclusions:

The interaction between mSVF and commercial dermal substitutes varies substantially, influencing cellular engraftment and secretory profiles. These findings highlight the importance of scaffold selection in tissue engineering strategies with mSVF and suggest potential clinical implications for optimizing regenerative outcomes in burn reconstruction.

## 7. Poster Presentations

P001

Skin substitutes and homologous grafts following enzymatic debridement in deep burns: an ideal dressing

**Mangia, A.** ^1^, Di Lonardo, A. ^1^, Bacci, B. ^1^, Tommasi, R. ^1^

^1^ Emergency Department Burn Center, Pisa, Italy

Poster presentations 1, 3 September 2025, 15:00–15:30

Aim:

To identify an ideal dressing following enzymatic debridement in burn patients.

Method:

This study retrospectively analyzed data from 20 patients with intermediate and deep burns affecting the trunk and limbs, with a mean age of 42 years. The mean total body surface area affected was 15%. None of the patients presented significant comorbidities. After enzymatic debridement, burn wounds were treated according to their depth: intermediate partial-thickness burns were conservatively managed with a synthetic absorbable skin substitute and treated as outpatients without the need for surgical intervention, while deep partial-thickness burns received homologous split-thickness skin grafts. Among the patients, 10 had intermediate partial-thickness burns, and 10 had deep partial-thickness burns. Days of hospitalization, healing times and scar assessment using the Vancouver Scar Assessment Scale for both burn categories were compared.

Conclusions:

Currently, there is no ideal dressing applicable to all burn areas following treatment with enzymatic debridement. The ideal dressing should maintain adequate moisture for the residual dermis, preventing potential formation of pseudo-eschar. It should also ensure patient comfort facilitating outpatient management, while promoting effective re-epithelialization. Based on our observations, a thorough assessment of the uniformity and integrity of the residual dermis after enzymatic treatment allows for optimal and definitive coverage with the polylactic acid epidermal substitute. This approach enables early hospital discharge and outpatient management enhanced patient comfort, reducing painful dressing changes with excellent aesthetic results.

P002

Use of a synthetic dermal regeneration matrix as an alternative for wound closure in a patient with severe alkali burns at a national referral center in Mexico.

Martínez, R. ^1^, Velez, M., Elnecavé, A., Ferreira, F., Estrada, J., Ochoa, A., Pérez, A.

^1^ Instituto Nacional de Rehabilitación “luis Guillermo Ibarra Ibarra”, Ciudad De México, Mexico

Poster presentations 1, 3 September 2025, 15:00–15:30

Aim:

To demonstrate the surgical success achieved with the use of a polylactic acid-based synthetic dermal regeneration matrix to provide skin coverage in an exposed osteotendinous area.

Methods:

A 54-year-old male with no history of chronic degenerative diseases suffered an alkali burn (caustic soda NaOH) affecting 35% of his total body surface area.

As part of the surgical treatment, a tangential excision was performed with harvesting and application of a meshed split-thickness skin graft on the right thoracic extremity and abdomen; a tangential excision and reverse circumcision were performed on the genitals; and, due to the severity of the injuries, a transfemoral amputation of the right pelvic extremity and a transtibial amputation of the left pelvic extremity were performed on the pelvic extremities.

During the skin coverage process, the patient presented exposure of the patella and the left infapatellar ligament. Because the surrounding structures were in suboptimal condition, it was decided to place a synthetic polylactic acid-based dermal regeneration matrix with a negative pressure system as a clamp, followed by a split-thickness graft to provide skin coverage and thus preserve the left knee joint.

Results:

The patient was discharged from the hospital after 40 days with the left knee joint preserved.

Conclusions:

The use of a synthetic polylactic acid-based dermal regeneration matrix allows for the closure of difficult-to-cover wounds, which is very beneficial in patients with severe alkali burns.

P003

Impact of a newly implemented protocol on surgically managed large full thickness burns at clinic of burns and plastic surgery in umhatem “N. I. Pirogov”

Argirova, M. ^1^, **Martinov, M.** ^1^, Pashov, I. ^1^

^1^ Umhatem “n. I. Pirogov”, Sofia, Bulgaria

Poster presentations 1, 3 September 2025, 15:00–15:30

Early excision and autografting of burn patients is one of the fundamental principles of acute burn care. In cases with large burns there are limited donor sites which causes the need for temporary coverage over the excised areas. The biosynthetic temporary skin substitute Biobrane™ provide the ideal wound coverage in these situations.

Aim:

To determine the efficacy of Biobrane^TM^ (Smith & Nephew, London, UK) application, as a temporary wound cover of tangentially excised full-thickness burns, donor sites and widely meshed autografts in the surgical treatment of severe burns.

Material and Methods:

Between February 2019–February 2024, 28 patients with large burns of various areas and localization were enrolled. The Biobrane™ was placed immediately on the excised wounds, donor site and widely perforated skin grafts when are available. The efficacy of this temporary coverage was analyzed according to the following parameters: adherence, fluid collection, rejection, infection, wound healing time, and follow up wound evaluation.

Results:

The mean age of the patients was 18.39 ± 12.30. The mean TBSA was 55.84 ± 10.70. In the areas of application there was firmly adhered Biobrane without rejection. Upon removal the underlying wound surfaces are viable, covered with granulation tissue, ready for grafting and the widely meshed autografts are stable with advanced epithelialization. The areas covered with Biobrane^TM^ were without or low frequency of bacterial contamination. Only three patients require regrafting. There was no mortality.

Conclusions:

The introduction of Biobrane^TM^ as a temporary coverage in the treatment protocol in operative behavior of large deep burns has shown excellent clinical results.

P004

Ten Years of Experience with Nexobrid: Evolution and Optimization of Enzymatic Debridement in Burn Treatment

**Mataro, I.** ^1^, Avvedimento, S. ^1^, Del Torto, G. ^1^, Alberico, A. ^1^, d’Angelo, D. ^1^, Petroccione, C. ^1^

^1^ Hospital A. Cardarelli, Naples, Italy

Poster presentations 1, 3 September 2025, 15:00—15:30

Aim:

To evaluate the clinical use of Nexobrid over a ten-year period and how its progressive optimization has impacted outcomes in burn care.

Methods:

We conducted a retrospective analysis of burn patients treated with Nexobrid from May 2015 to January 2025.

Patients with second- and third-degree burns and a minimum 6-month follow-up were included. They were grouped into three time periods:

- Initial phase (Years 1–3): early use, protocol development
- Intermediate phase (Years 4–6): refined indications and techniques
- Advanced phase (Years 7–10): standardized, optimized use

Analyzed parameters included: burn characteristics, timing and number of applications, treated surface area, pain management, post-treatment dressings, need for escharotomy or surgery, and hospital stay duration.

Results:

820 patients were included. Over time, treated surface areas increased and application timing extended up to 72 h. In patients with more extensive burns, the number of applications grew. Pain control improved through multimodal strategies, particularly local/regional anesthesia. Wound care protocols became standardized. A notable outcome was the reduced need for escharotomies and surgical interventions, alongside shorter hospital stays.

Conclusions:

A decade of Nexobrid use highlights the importance of experience in optimizing enzymatic debridement. Improvements in timing, application strategies, pain control, and wound care have positively influenced clinical outcomes, reducing surgical needs and hospitalization length.

P005

Evaluation of the Use of Flaminal^®^ after Treatment with Nexobrid^®^ in the Management of Intermediate Burns: Our Preliminary Experience

Mataro, I. ^1^, **Avvedimento, S.** ^1^, Manfellotto, V. ^1^, Di nola, G. ^1^, Manzi, E. ^1^, La Torre, F. ^1^, Razzano, S. ^1^, D’angelo, D. ^1^, Petroccione, C. ^1^

^1^ Hospital A. Cardarelli, Naples, Italy

Poster presentations 1, 3 September 2025, 15:00–15:30

Aim:

To describe the preliminary experience using Flaminal^®^, an enzyme alginogel with antimicrobial action, in the treatment of patients with moderate burns (10–25% of total body surface area burned), assessing its role in pain management, exudate control, infection prevention, and facilitation of wound healing.

Methods:

A retrospective observation of 15 patients admitted to our unit from September 2024 to January 2025.

Inclusion criteria: patients with superficial and deep second-degree burns, treated with Nexobrid^®^, with potential for spontaneous healing.

Treatment protocol: Enzymatic debridement within the first 48–72 h, followed by dressings with paraffin gauze and Flaminal^®^ until complete healing.

Parameters evaluated: healing time, pain, need for surgical intervention, infectious complications, and dressing change frequency.

Results:

- Average treated burn surface: 8% (range 5–12%).
- Dressing changes: every 3 days on average (range 1–4).
- Average healing time: 20 days (range 15–37).
- One patient required surgical skin grafting after 21 days due to high risk of hypertrophic scarring.
- No major infections occurred.

Conclusions:

The use of Flaminal^®^ in combination with Nexobrid^®^ and paraffin gauze proved to be a promising strategy in the management of moderate burns with potential for spontaneous healing, facilitating the healing process.

The protocol allowed effective exudate control thanks to Flaminal^®^’s properties, which support autolytic debridement and wound edge protection.

The reduced frequency of dressing changes represents an additional benefit in terms of patient comfort and pain management compared to traditional dressings.

Larger studies are needed to confirm these preliminary findings.

P006

Comprehensive Review of 550 Applications of Fish Skin Graft

**Mcgroarty, C.** ^1^

^1^ Wakemed Health and Hospitals, Raleigh, United States

Poster presentations 1, 3 September 2025, 15:00–15:30

Aim:

The management of wounds varies significantly in clinical practice. The use of xenograft to assist or complete wound closure is standard or care, however selection of the xenograft varies in clinical practice. This retrospective review of 550 applications of fish skin graft 8/2022 to 3/2025 demonstrates successful closure of partial to full thickness thermal, chemical and friction burn wounds, wounds from infectious causes and traumatic wounds.

Methods:

This is a retrospective review of 550 operating room fish skin graft applications. The cases were reviewed for wound type, median days to closure, additional graft requirement, length of hospital stay post application, wound care requirements post application, outcome and complications.

Results:

Fish skin graft application is an effective treatment to diverse wound types demonstrating reduced median days to closure, decreased additional graft requirement, decreased length of hospital stay post application, reduced wound care requirements post application, excellent wound healing and outcomes, and less a than 1% complication rate.

Conclusions:

This retrospective review of fish skin graft application demonstrates that fish skin graft is a valuable, safe and effective treatment for successful closure of partial to full thickness thermal, chemical and friction burn wounds, wounds from infectious causes and traumatic wounds with minimal post application complications.

P007

Complex abdominal wall wound closure using a staged closure with early application of fish skin graft followed by autograft

**Mcgroarty, C.** ^1^

^1^ Wakemed Health and Hospitals, Raleigh, United States

Poster presentations 1, 3 September 2025, 15:00–15:30

Aim:

The management of large anterior abdominal wall tissue defects varies in clinical practice. Some patients may not be appropriate or medically cleared for component separation. This case series presents three patients that were managed with early application of fish skin graft followed by autograft with successful closure.

Methods:

This is a series of three cases with large abdominal wall tissue defects requiring complex abdominal wall wound closure. The first patient had a necrotizing soft tissue infection that caused the abdominal wall wound. The second patient had a complex abdominal wall wound with exposed bowel from a seatbelt in an MVC. The third patient was a gunshot to the back and required multiple abdominal surgeries leaving exposed bowel in a large anterior abdominal wall defect. The patients were managed with early application of fish skin graft and then autografted.

Results:

Three patients were successfully stabilized and then the complex abdominal wall wounds were closed using fish skin graft to prepare the wound for autograft.

Conclusions:

This case series illustrates that complex abdominal wall wound closure and autografting is an option for early management of complex abdominal wall wounds. Complex abdominal wall wound closure can be achieved in acute traumatic and post necrotizing infection wounds with staged fish skin graft and autograft.

P008

A Side-by-Side Comparison of Bovine Collagen Tissue Matrix and Acellular Fish Skin Grafts Applied to a Full-Thickness Traumatic Avulsion injury to the Left Upper Extremity.

**Mcgroarty, C.** ^1^

^1^ Wakemed Health and Hospitals, Raleigh, United States

Poster presentations 1, 3 September 2025, 15:00–15:30

Aim:

Full-thickness wound management often includes the use of biologic substitutes after debridement to expedite healing, reduce pain, lower infection risk, prevent fluid loss, and simplify wound care. This case study compares bovine collagen tissue matrix and acellular fish skin graft. The patient sustained a full thickness traumatic avulsion with exposed tendon and muscle to the left upper extremity in a motorcycle collision. This case study illustrates the timing of product incorporation and granulation tissue formation via photograph analysis, and tracks wound re-epithelialization through images and wound size measurements.

Methods:

This study depicts one patient with a full thickness traumatic avulsion injury to the left upper extremity and the results noted in wound closure with a side-by-side application of bovine collagen tissue matrix and acellular fish skin graft. Post application of the xenografts, negative pressure therapy was applied and maintained until postoperative day (POD) 7.

Results/Conclusions:

In this side by side comparison the acellular fish skin graft application reduced the wound depth and resulted in more granulation tissue covering the exposed structures allowing more rapid definitive closure with autograft. The wound area of fish skin graft demonstrated less wound contracture during wound healing and remodeling.

P009

Why we use polylactic dressings in burns?

**Miranda, A.** ^1^

^1^ Hospital Civil De Guadalajara, Guadalajara, Mexico

Poster presentations 1, 3 September 2025, 15:00–15:30

In the last 5 decades the management of Burns has evolved dramatically.

The search for other treatment options for the coverage of the breakaway using synthetic dermal substitutes that can supplement autologous dermal grafts but also help heal areas with chronic lesions.

There is a very complex network of physiological changes enabling wound healing. The immunological process enables the body to distinguish damaged cells (homing) and begin a cleaning mechanism by separating damaged proteins and cells with matrix metalloproteinases, a complement reaction, and free radicals. One of the earliest investigated local factors is the pH of wounds, studied in close relation to the local perfusion, oxygen tension, and lactate concentration. Neovascularization depends on oxygen provision and lactate, signaling hypoxic conditions even under normoxic conditions. An appropriate pH is necessary for successful skin grafting; hypoxia can change the pH of wounds.

Since 2011 we have been using polylactic acid dressings to treat pediatric burns. 2257 patients were received in Pediatric Burn Unit of the Civil Hospital of Guadalajara, Mexico. 73.23% (1653) of these were treated with the polylactic acid dressing. In 1074 patients we used it as a dermal substitute. Only 89 infections (5.38%). 7.80% of patients required two applications (129).

579 donor sites treated. Average area affected 22.5% TBA

Less pain. 1.2% mortality rate

Hospital stays 0.8 per percentage point

Of these lesions, including 3rd degree lesions, small lesions epithelialize correctly, and fewer autografts are required (only 27.5% 415 patients)

P010

Preliminary Experience Using Artificial Intelligence and Multispectral Imaging for Burn Depth Assessment.

**Moran, H.** ^1^, Concannon, E. ^1^, Solanki, N. ^1^, Wagstaff, M. ^1^

^1^ Burns Unit Royal Adelaide Hospital, Adelaide, Australia

Poster presentations 1, 3 September 2025, 15:00–15:30

Aim:

To evaluate the accuracy of a combined artificial intelligence (AI) and multispectral imaging (MSI) device in predicting burn healing within three weeks, compared to clinical predication with expert consultant consensus.

Methods:

A study was conducted in a major tertiary burn centre including adult patients with partial-thickness burns. All patients underwent burn wound imaging using the Spectral AI Device at the time of their initial presentation and predictions were compared with blinded consensus assessments from specialist burn surgery consultants. The primary outcome was device accuracy compared with clinician opinion in correctly predicting burn wound healing within three weeks, validated against clinical assessment of healing time.

Results:

The combined AI and MSI device demonstrated acceptable levels of accuracy in predicting burn healing, correctly correlating with clinical outcomes in the majority of cases. Comparative analysis of AI-generated predictions with expert clinical assessments revealed areas for improvement in image acquisition. Specific techniques for improving the device’s reliability were identified.

Conclusions:

AI and MSI technology shows promise in burn depth assessment and healing prediction. Further multi-centre validation with larger patient cohorts is needed to refine accuracy and reliability. The integration of AI-based tools with clinical expertise could enhance burn care decision-making.

P012

Treatment of 2a-b degree burns of the face with a alloplastic nanocellulose-based epidermal substitute (epicite hydro).

**Nietzschmann, I.** ^1^

^1^ BG-Klinik, Halle, Germany

Poster presentations 1, 3 September 2025, 15:00–15:30

Aim:

The treatment of 2a-b degree facial burns is carried out conservatively in our burn centre. We have treated more than 100 patients with Epicite Hydro (BioSkinCo, Tlalnepantla, Mexico) to determine the feasibility and potential benefits compared to our previous treatment procedure.

Methods:

On the day of admission, after the usual admission procedure in the form of a full body wash/shave and dermabrasion, epicite hydro was immediately applied to the burnt area instead of the previously used antiseptic moist dressings.

The applied epicite hydro face mask remained on the face and was moisturised with an antiseptic solution daily for up to 3 days for large 2b degree areas. For 2a degree burns, the epicite hydro remained on the face without further action.

The Epicite Hydro was only changed if there was a strong feeling of tightness.

In most cases, it remained unchanged until completely dry after more than 10 days.

Results:

No further dressing changes were necessary with this treatment regime. Later surgery was not necessary in any patient.

Conclusions:

With the right indication, patients benefit from a reduction in pain and significantly fewer dressing changes with a good functional and aesthetic result.

P013

Application of a synthetic, resorbable dermis replacement material for severe soft tissue injuries.

**Nietzschmann, I.** ^1^

^1^ BG-Klinik Halle/Saale, Halle, Germany

Poster presentations 1, 3 September 2025, 15:00–15:30

Aim:

Severe soft tissue defects, especially after necrotizing fasciitis and décollement injuries, represent a challenge in surgical treatment.

Methods:

We would like to present 2 cases of different genesis that we treated with a synthetic, resorbable dermis substitute.

Results:

We transferred the patients from external clinics for further treatment after initial surgery had failed to achieve primary wound healing.

Conclusions:

Using a synthetic skin substitute, all soft tissue defects were healed and definitive wound closure was achieved without significant functional restrictions. as achieved.

P014

Initial Experience and Pitfalls in the Use of NovoSorb BTM for Burn Reconstruction

**Norling, A.** ^1^, Lang, C. ^1^, Holmgaard, R. ^1^, Drejøe, J. ^1^

^1^ Rigshospitalet, Copenhagen, Denmark

Poster presentations 1, 3 September 2025, 15:00–15:30

Aim:

To evaluate our early clinical experience and challenges with NovoSorb BTM (PolyNovo, Melbourne, VIC, Australia) in complex burn injuries.

Methods:

We performed a retrospective single-center case series at a specialized burn unit, including all burn patients treated with NovoSorb BTM since its introduction in our department. Data included demographics, indication, anatomical site, time to BTM integration and skin grafting, as well as complications such as BTM failure.

Results:

14 patients were included. NovoSorb BTM facilitated reconstruction of full-thickness burns with exposed tendon, bone, or other vital structures where direct grafting was not feasible. When successful, it promoted a stable, dermis-like granulation tissue, allowing for rapid and durable graft integration. Initial BTM failure occurred in six patients, though two were successfully re-treated with a second BTM application. Failure was mainly linked to infection or poor adherence due to friction or suboptimal contact. Some in the failure group were critically ill with a very poor healing potential. Four patients died—three from their burn injuries and one following a suicidal act—none of the deaths were related to BTM.

Conclusions:

NovoSorb BTM is a valuable tool in burn reconstruction. Success requires infection control and close contact between matrix and wound bed. In severely ill patients, BTM may be used as a last resort despite the risk of failure. Recognizing these limitations can guide case selection and set realistic expectations.

P015

“Use of a resorbable microporous synthetic membrane (Suprathel^®^) as a useful alternative for reducing recovery time and minimizing complications in donor sites of severely burned patients.”

**Ochoa Rodriguez, A. **^1^, Vélez Palafox, M.

^1^ Instituto Nacional De Rehabilitación, Mexico City, Mexico

Poster presentations 1, 3 September 2025, 15:00–15:30

Aim:

To evaluate the efficacy of Suprathel^®^ in reducing recovery time of donor sites in severely burned patients, compared to conventional treatments.

Methods:

A prospective study was conducted in patients with severe burns who required skin grafts. The cohort was divided into two groups: one received Suprathel^®^, and the other received conventional treatment (paraffin gauze dressings). Pain, epithelialization time, and infection risk were evaluated over a 21-day period.

Results:

Suprathel^®^ demonstrated a shorter healing time, reduced pain, and a lower incidence of infections compared to the control group, which used paraffin-impregnated dressings.

Conclusions:

Suprathel^®^ represents an effective alternative to optimize recovery of donor sites in severely burned patients, reducing complications and improving the patient’s quality of life.

P016

Cost-effectiveness of native collagen-elastin dermal regeneration template in chronic and acute wounds

**Oehlbauer, M.** ^1^

^1^ BG Trauma Center Murnau, Murnau, Germany

Poster presentations 1, 3 September 2025, 15:00–15:30

Aim:

An acellular single-layer dermal substitute composed of native bovine collagen (types I, III, and V) and elastin hydrolysate can be used in single-step and two-step reconstruction surgeries in several skin defects. The objective of this study is to assess the cost-effectiveness of native collagen-elastin dermal regeneration template in the treatment of both acute and chronic wounds from the perspective of the healthcare systems.

Methods:

The analyses were performed comparing native collagen-elastin dermal regeneration template with split-thickness skin graft (STSG) alone and other dermal templates. Diabetic foot ulcer (DFU) and burns were the indications selected to represent chronic and acute wounds, respectively. Separate mathematical models were developed for each indication: a Markov model for DFU and a decision tree model for burns.

Results:

Native collagen-elastin dermal regeneration template was cost-effective in all comparisons, when compared to other dermal templates in acute wounds with marginal quality-adjusted life-year (QALY) gains). In chronic wounds native collagen-elastin dermal regeneration template led to savings and QALY gains against all comparators with incremental QALYs and cost savings compared to STSG, other dermal templates and outpatient templates, respectively.

Conclusions:

Highlighting the importance of a holistic economic approach considering cost factors as material costs, hospitalization time and complications native collagen-elastin dermal regeneration template has proven to be more effective and less costly (i.e., dominant) than all comparators. As the clinical data is obtained from heterogeneous populations and varying wound characteristics, the obtained can be seen as an indicator.

P017

Key players in physiological tissue repair

Optimizing the complex cell dialogue during tissue repair processes by using rapid vascularized collagen/elastin matrix.

**Oehlbauer, M.** ^1^

^1^ BG Trauma Center Murnau, Murnau, Germany

Poster presentations 1, 3 September 2025, 15:00–15:30

Aim:

Fibroblasts are key players for maintaining skin homeostasis and for orchestrating physiological tissue repair.

Keratinocytes play an important role in cutaneous cell-cell communication for wound healing outcome.

A rapid vascularized tissue matrix, consisting of native collagen (collagen type I, III and V) supplemented by an elastin hydolyzate is used since more than 20 years especially as dermal template.

We here report our—after 20-years experience—optimized setting using this tissue matrix for defect coverage especially in complex wounds and its impact in keraticocyte-fibroblast crosstalk.

Methods:

Different settings of wound bed preparation with and without using tissue matrix were compared intra-individual and to patients treated by these different settings in our level I trauma center since 2003. Outcome quality of the scar tissue was assessed using electron mikoskope.

Results:

Human fibroblasts showed best cell proliferation and collagen synthesis when precise debridement including complete removal of granulation tissue was performed just before application of collagen/elastin matrix.

Long follow up of these collagen-elastin matrix procedures in defect coverage showed much faster maturation of the scar and excellent functional outcome without occurence of unstable scaring or requirement of scar revision.

Conclusions:

The intimate dialogue between fibroblasts and collagen-elastin matrix represents a fascinating domain in order to characterize the therapeutic targets especially to prevent pathological developments of myofibroblasts but also to interfere with keraticocytes to induce cutaneous cell-cell communication.

In clinical settings precise wound bed preparartion has shown to be the crucial key point to maximize quality and function of reconstructed tissue.

P018

What to do when surgical choice brings the worst complication? Boldly aggressive surgical debridement or patiently chemical debridement?

**Oral, A.** ^1,2^, Sevinc, D. ^2^, Yavuz, M., Aydin, I. ^2^, Hepguler, I. ^2^

^1^ University of Health Sciences, Izmir Faculty of Medicine, Izmir, Turkey, ^2^ Izmir City Hospital, Department of Pediatric Surgery, Izmir, Turkey.

Poster presentations 1, 3 September 2025, 15:00–15:30

Aim:

Treatment of skin necrosis on the back of a newborn born with a diagnosis of meningomyelocele without aggressive surgical excision due to the risk of perforation and meningitis.

Method:

A 3700 g girl, born at 37 weeks of gestation, was operated by pediatric neurosurgeons on the second after the delivery. The meningomyelocele sac and its contents were excised. The dura mater defect was repaired using Duragen^®^ bovine “Collagen dural graft matrix” patch (Integra LifeSciences, Princeton, NJ, USA), and the dorsal skin was primarily sutured on the Duragen^®^ in an obligatory plane. Relaxation incisions were made in both flank areas withoutany flap displacement, and ischemia observed in the skin flaps during the operation.

On the 6th postoperative day, a consultation was requested by the pediatric surgery department to assess the skin, and it was detected a widespread skin necrosis up to the flank area where the relaxation incisions were made. Chemical debridement with enzymealginogel was administered instead of surgical debridement due to the risk of dura mater perforation and meningitis. Flaminal^®^ was applied to the field every other day, and the patient was given systemic antibiotics. Although grafting was planned in case of granulation tissue development, the treatment was continued as the wound started to contract very well.

Result: Complete epithelialization was achieved, and the patient was discharged on the 38th day.

Conclusions:

In cases where surgical debridement is threatening, applying enzymealginogel using conservative methods may provide a safer treatment for the patient.

P019

Management of Scalp Necrosis Following Hair Transplantation: A Case Report

**Ozkan, B.** ^1^, Tatar, B. ^1^

^1^ Medipol University, İstanbul, Turkey

Poster presentations 1, 3 September 2025, 15:00–15:30

Title:

Management of Scalp Necrosis Following Hair Transplantation: A Case Report

Aim:

To present a case of post-hair transplant scalp necrosis managed with Flaminal^®^ Forte, Biodegradable Temporizing Matrix (BTM), and full-thickness skin grafting for optimal reconstruction.

Methods:

A 52-year-old man presented with necrotic scalp tissue and severe forehead edema five days after a hair transplant. Examination revealed two 4 × 4 cm necrotic islands in the frontoparietal region, fluctuance, and purulent drainage. Debridement exposed full-thickness defects, including periosteum. The wounds were irrigated with hydrogen peroxide and treated with daily Flaminal^®^ Forte. Broad-spectrum antibiotics were administered based on wound cultures. After two weeks, a cavitary wound with granulation tissue formed, and NovoSorb^®^ BTM (PolyNovo, Melbourne, VIC, Australia) was applied for contour restoration. After five weeks, the BTM outer layer was removed, revealing healthy granulation tissue. Two 4 × 3 cm defects were reconstructed with full-thickness, unmeshed en bloc skin grafts containing hair follicles from the occipital scalp.

Results:

The patient showed progressive wound healing with complete graft take. Hair follicle growth was observed by postoperative week four. There were no complications, and scalp contour was successfully restored.

Conclusions:

Flaminal^®^ Forte effectively prepared the wound bed, while BTM helped correct cavitation and maintain scalp contour. Full-thickness scalp grafting should be considered for aesthetic scalp defect reconstruction.

P020

Strategic Application of BTM in Free Flap Surgery: Indications and Timing

**Ozkan, B.** ^1^, Tatar, B. ^1^

^1^ Medipol University Faculty of Medicine, Department of Plastic, Reconstructive and Aesthetic Surgery, İstanbul, Turkey.

Poster presentations 1, 3 September 2025, 15:00–15:30

Title:

Strategic Application of BTM in Free Flap Surgery: Indications and Timing

Aim:

Free flap surgery is a cornerstone of complex defect reconstruction in microsurgery. Despite high success rates, challenges such as donor-site morbidity, partial flap necrosis, and delayed wound healing persist. PolyNovo NovoSorb^®^ Biodegradable Temporizing Matrix (BTM) has emerged as a valuable tool in complex wound management. This study evaluates its role in free flap surgery, particularly in cases of partial flap failure and donor site complications, focusing on optimal application sites and timing.

Methods:

Between August 2024 and February 2025, 15 patients underwent free flap surgery where BTM was utilized in cases where conventional closure or grafting was not feasible. Indications included unreliable muscle flaps, marginal necrosis, secondary dehiscence, graft loss, pedicle sites, and donor sites. BTM was applied either immediately postoperatively or during secondary wound management.

Results:

BTM application yielded positive outcomes across multiple scenarios. Where? BTM was applied to muscle flaps, marginal necrosis sites, pedicle sites, and donor sites. When? In cases of unreliable muscle flaps, BTM provided a stable wound bed for delayed grafting. For marginal necrosis and pedicle sites, BTM was applied after debridement, promoting secondary healing. When used over muscle flaps, it reduced the grafting requirement. At donor sites, BTM facilitated satisfactory wound closure.

Conclusions:

BTM effectively supports wound healing in free flap surgery, reducing the need for additional interventions. Its strategic application, based on defect location and healing phase, enhances secondary and delayed healing.

P021

Reconstruction of Severe Neck Contracture with Super-thin ALT Flap in a Pediatric Patient: Case Report.

**Ozkan, B.** ^1^, Tatar, B. ^1^

^1^ Medipol University Department of Plastic, Reconstructive and Aesthetic Surgery, İstanbul, Turkey.

Poster presentations 1, 3 September 2025, 15:00–15:30

Title:

Reconstruction of Severe Neck Contracture with Super-thin ALT Flap in a Pediatric Patient: Case Report.

Aim:

To present a case of severe neck contracture in a pediatric patient, managed with super-thin anterolateral thigh (ALT) flap for reconstruction, aiming to restore both function and aesthetics.

Methods:

A 7-year-old male presented with severe neck contracture due to a deep second-degree scald burn affecting the lower face, anterior neck, and chest. After six months of secondary healing, the contracture caused downward displacement of the lower lip, difficulty speaking and retaining saliva, restricted neck extension, and left eye ectropion. A surgical plan was made for scar excision and reconstruction using a super-thin ALT flap. Fiberoptic-assisted intubation was performed, and scar excision was carried out in a W-plasty fashion to redefine the mandibular border and cervicomental angle. The defect measured 16 × 12 cm post-excision. A 16 × 10 cm ALT flap was harvested and adapted to the defect. Microvascular anastomosis was performed using 10–0 nylon sutures, and the donor site was covered with a skin graft.

Results:

Postoperative imaging demonstrated significant improvements in both function and aesthetics. The ectropion and lower lip malposition were corrected, and the patient regained full neck mobility. The patient was able to speak, laugh, and eat without restriction.

Conclusions:

This case demonstrates the effectiveness of super-thin ALT flaps in pediatric post-burn neck contracture reconstruction, achieving excellent functional and aesthetic outcomes.

P023

Establishment of operative procedure for management of enzymatic debridement

**Pensa, A.** ^1^, Romeo, M. ^1^, Sciarrillo, A. ^1^, Depetris, N. ^2^, Casalis, C. ^1^, Bommarito, C. ^1^, Navissano, M. ^1^

^1^ Burn Center, AOU Città della Salute e della Scienza, Torino, Italy, ^2^ Anesthesia and Intensive Care 3, Department of Anesthesia and Intensive Care, City of Health and Science, CTO Hospital, Turin, Italy.

Poster presentations 1, 3 September 2025, 15:00–15:30

Aim:

In the Burn centre of Turin we create an interdisciplinary group for writing an operative procedure for enzymatic debridement to standardize the application.

Methods:

During two months after the review of literature we draw up the operative procedure describing the indications, contraindications, precautions, preoperative preparation (consent, wound assessment, cleaning) materials needed for all phases, procedure steps, the covering medication, the anesthesiology care, the multidisciplinary staff, the postoperative care, documentation.

Results:

The multidisciplinary group draws up the operative procedure in all the fields and an evaluation patient’s sheet.

Conclusions:

The organisation and developing of a new procedure between all the health professionals can be a key for a more correct application of the enzymatic debridement.

P024

The effectiveness of enzymatic debridement (NexobridR) in the treatment of post-burn compartment syndrome of the upper limb.

**Pertea, M.** ^1,2^, Bulgaru-Iliescu, A. ^1,2^, Amarandei, A. ^2^, Benamor, M. ^2^, Avadani, B. ^2^, Moraru, D. ^1,2^

^1^ Grigore T Popa University of Medicine and Pharmacy Iasi, Romania/Sf Spiridon Emergency Hospital Burn Unit Iasi, Romania, Iasi, Romania, ^2^ Sf Spiridon Emergency County Hospital Burn Unit Iasi Romania, Iasi, Romania.

Poster presentations 1, 3 September 2025, 15:00–15:30

Aim:

Enzymatic debridement represents a viable alternative to surgical debridement, providing rapid action, selective tissue removal, and an improved safety profile. This study aims to evaluate the efficacy of NexobridR (Yavne, Israel) as a first-line treatment for compartment syndrome in circumferential burns of the upper limb. By employing NexobridR as an initial intervention in such cases, the need for decompressive incisions may be avoided.

Methods:

We studied a cohort of 27 patients with burns of various etiologies and surface areas, all presenting with deep burns (IIB and III degree). Circumferential burn injuries affected one or both upper limbs, with compartment syndrome either diagnosed or in progression. NexobridR was applied as a first-line treatment in all cases, following the manufacturer’s guidelines in every instance.

Results:

The study cohort comprised 8 female and 19 male patients. Thermal injuries were predominantly caused by scalds from hot liquids (74%), while the remaining cases resulted from flame burns. Following NexobridR application, none of the patients required decompressive fasciotomy for compartment syndrome management. In 35% of cases, skin grafting was indicated, utilizing the micrografting Meek technique to optimize wound coverage. In the remaining cases, a strategy of guided spontaneous epithelialization was pursued to facilitate tissue regeneration.

Conclusions:

The use of bromelain demonstrates both safety and efficacy in wound care. The application of NexobridR eliminates the need for surgical treatment of compartment syndrome and, in many cases, even the necessity for skin grafting, allowing for guided spontaneous epithelialization with superior functional and aesthetic outcomes compared to grafting.

P025

Enzymatic debridement, the “gold-standard” in the therapeutic protocol for hand burns

**Pertea, M.** ^1,2^

^1^ Grigore T Popa University of Medicine and Pharmacy Iasi, Romania, Iasi, Romania, ^2^ Sf Spiridon Emergency Hospital Burn Unit Iasi, Romania, Iasi, Romania

Poster presentations 1, 3 September 2025, 15:00–15:30

Aim:

Enzymatic debridement represents an alternative to surgical debridement, offering speed, tissue selectivity and increased safety. The aim of the study is to demonstrate the need to introduce enzymatic debridement as the “gold-standard” in the therapeutic protocol of IIB and III degree burns on the hand.

Methods:

We studied a group of 40 patients with burns of different etiologies, deep (degree IIB and III), localized on the hands (including fingers), with surgical indication considering the depth of the burn. Since an excision on the dorsal surface of the fingers and hand would have left the extensor tendons exposed with reduced possibilities of coverage, the first intention use of enzymatic debridement was chosen in all cases.

Results:

Of the studied group, 24 cases were female patients (two of them being surgeons). The application of enzymatic debridement was followed by applications of moist and topical hyaluronic acid-based dressings. In none of the cases was it necessary to use split free skin grafts, all cases benefiting from spontaneous epithelialization. The functional and aesthetic results were good with full satisfaction from the patients. In none of the cases was the presence of retractions recorded and the aesthetic appearance including only moderate hyperchromia of the scars.

Conclusions:

The use of enzymatic debridement in deep burns of the hands and fingers avoids tendon exposure (from surgical excisions), most often does not require grafting and the aesthetic and functional results are clearly superior to surgical ones, to which is added the absence of a graft donor site.

P026

Dermal skin substitutes in burn care: the perspective of scarless wound healing

**Pijpe, A.** ^1,2,3^, van den Bosch, A. ^1,2,3^, Verwilligen, R. ^1,2,3^, van der Vlies, C. ^4,5,6^, Bosma, E. ^7^, van Zuijlen, P. ^1,2,3,8^, Middelkoop, E. ^1,2,3^, National Burn Care, Education & Research Group The Netherlands

^1^ Alliance of Dutch Burn Care, Burn Centre, Red Cross Hospital, Beverwijk, The Netherlands, ^2^ Amsterdam UMC location Vrije Universiteit Amsterdam, Department of Plastic Reconstructive and Hand Surgery, Amsterdam, The Netherlands, ^3^ Amsterdam Movement Sciences, Tissue Function and Regeneration, Amsterdam, The Netherlands, ^4^ Alliance of Dutch Burn Care, Burn Centre, Maasstad Hospital, Rotterdam, The Netherlands, ^5^ Department of Trauma and Burn Surgery, Maasstad Hospital, Rotterdam, The Netherlands, ^6^ Trauma Research Unit, Department of Surgery, Erasmus MC, University Medical Centre, Rotterdam, The Netherlands, ^7^ Alliance of Dutch Burn Care, Burn Centre, Martini Hospital, Groningen, The Netherlands, ^8^ Department of Plastic, Reconstructive and Hand Surgery, Red Cross Hospital, Beverwijk, The Netherlands

Poster presentations 1, 3 September 2025, 15:00–15:30

Aim:

To determine the present and future position of dermal skin substitutes in burn care, and identify challenges and opportunities to further enhance this field.

Methods:

A mixed-method study was performed and included an international survey, a systematic review and meta-analyses, and two innovation projects targeted at developing a treatment algorithm and decision aid for the application of dermal substitutes in acute burn care.

Results:

Two thirds of 148 included international experts acknowledged the efficacy of dermal substitutes. However, they reported experienced barriers in terms of costs and contamination risk, and emphasised the absence of clear indications and treatment protocols. Efficacy was confirmed by a meta-analyses on 31 comparative trials which highlighted improved scar quality in burn patients despite a slightly delayed wound healing. Extraction of indications from 190 studies revealed that wound depth was the primary indication for dermal substitute use. No age or burn/scar location thresholds were identified. Contraindications include wound infections and allergies to matrix components. Limited data exist on use in patients with comorbidities. Based on these data, a treatment algorithm was developed. To enhance implementation and shared-decision making, a decision aid was created in co-design with patients and clinical professionals.

Conclusions:

Despite proven efficacy in trials, dermal substitutes lack a firm footing in treatment protocols, with clear indications still undefined. Opportunities lie in the use of science-based implementation strategies, real-world data, cost-effectiveness studies, and patient perspectives. Lessons from the biomaterial journey could be applied in future developments such as bioengineered skin products.

P028

A new dermal substitute—our experience in multitrauma patients

**Sabag, N.** ^1^, Bloom, G. ^1^, Ron, O. ^1^, Hakrush, S. ^1^, Grinberg, R. ^1^, Jacobi, E. ^1^, Halpern, D. ^1^, Silberstein, E. ^1^, Shoham, Y. ^1^

^1^ Soroka medical center, Beer Sheba, Israel

Poster presentations 1, 3 September 2025, 15:00–15:30

Aim:

To evaluate the efficacy of NovoSorb BTM (Biodegradable Temporizing Matrix) as a dermal substitute for patients with complex soft tissue injuries requiring staged reconstruction.

Methods:

This retrospective study examines the use of BTM in 20 multitrauma patients with extensive soft tissue loss due to various etiologies, including burns, fasciotomies, surgical interventions, and traumatic injuries. BTM, a synthetic dermal scaffold composed of polyurethane open-cell foam with a sealed silicone layer, was applied to promote neo-dermis formation while maintaining a controlled wound environment. Vacuum-assisted closure (VAC) therapy was used over BTM to enhance wound bed preparation. Skin grafting was performed upon achieving complete integration, indicated by capillary refill.

Results:

The mean time from BTM implantation to grafting was three weeks. All patients demonstrated full integration of the matrix, allowing for successful skin grafting. Wound healing was achieved with minimal complications, and no graft loss was observed. The patient series also included one patient where an 18% total body surface area (TBSA) burn was completely covered with BTM, and resulted in complete graft take. BTM facilitated vascularization and improved wound bed quality, supporting its role as a reliable alternative to complex reconstruction.

Conclusions:

BTM provides an effective solution for soft tissue coverage in multitrauma patients, particularly when immediate complex reconstruction is not feasible due to the patient’s general condition or wound characteristics. Its ability to create a vascularized wound bed while minimizing donor site morbidity makes it a valuable option in staged reconstruction. Further research should assess long-term functional and aesthetic outcomes.

P031

Use of Manuka honey in the burned unit in a third level hospital

Senosiain Sancho, L. ^1^, González Díez, M. ^1^, Rogel Arenas, L. ^1^, **Sinovas Rodríguez, M.** ^1^

^1^ Unidad de Grandes Quemados—OSI Ezkerraldea-Enkarterri-Cruces. Osakidetza, Barakaldo, Spain

Poster presentations 1, 3 September 2025, 15:00–15:30

Aim:

Review the properties of manuka honey and describe our experience with the different application formats of the market.

Methods:

The composition, mechanisms of action and indications of Manuka honey are described in patients with second degree burns. The advantages and disadvantages found after the observation of the application are detailed in the patients that meet the inclusion criteria.

Results:

After reviewing patients in whom the protocol of Application of manuka honey has been applied, the following results are exposed:

Some of the main advantages are the easy application that helps to short up the time of curing process, the decrease in the infection rate, the reduction of inflammation and pain thanks to its properties anti-inflammatory, the great debridement capacity and the acceleration of the healing process.

With regard to the inconveniences found, it stands out that they are not applicable in very exudative and/or bleeding wounds because it involves an increase of the dressings replacement frequency.

Conclusions:

In our experience, the use of Manuka honey facilitates the cures with good results in terms of epithelialization and infection rate.

The good results observed in healing make its use suitable in areas that require a certain level of aesthetics. Its wide range of formats and easy application makes possible to use Manuka honey in burns of any extent, wich is a benefit for the clinical nursing practice as well as for the patient’s comfort.

P032

Initial experience closing burns and wounds with the next-generation autologous cell harvesting device in a US regional burn center

**Sood, R.** ^1^, Craft-Coffman, B. ^1^

^1^ JMS Burn Centers, Augusta, Georgia, USA

Poster presentations 1, 3 September 2025, 15:00–15:30

Aim:

Approved in May 2024, the next-generation autologous cell harvesting device (NG-ACHD) enhances workflow efficiency, optimizes enzyme incubation time, and simplifies device operation; this study evaluates its initial clinical outcomes and effectiveness in closing burns and complex wounds in a US regional burn center.

Methods:

A retrospective chart review was conducted on patients who received treatment with autologous skin cell suspension (ASCS) prepared by NG-ACHD between 31 May 2024, and 12 November 2024. Demographics, burn/wound etiology, percent total body surface area (TBSA), treatment approach, and clinical outcomes were analyzed. 

Results:

Of 56 patient charts reviewed, 36 met the inclusion criteria. The cohort contained 29 males (81%) and 7 females (19%) with an average age of 50 years. Injuries included thermal burns (66%), chemical burns (6%), electrical burns (6%), and various wounds (22%), such as hidradenitis, degloving, necrotizing fasciitis, purpura fulminans, and pyoderma gangrenosum. Average TBSA was 22%. Nineteen patients (53%) required burn ICU admission, while 17 (47%) were treated in non-ICU settings. Average time to ASCS application was 11 days. Treatments included ASCS alone, ASCS over autograft, and ASCS over widely meshed autograft. Consistent documentation of percent graft take was unavailable; however, only one patient required ASCS reapplication to the same treatment area, yielding a success rate of 97% for wound closure.

Conclusions:

This study demonstrates that the NG-ACHD is effective for managing burns and complex wounds. Its streamlined workflow and reliable outcomes support its integration into burn and wound care treatment protocols.

P033

White phosphorus: analysis of devastating cutaneous injuries and systemic toxicity

**Stonová, C.** ^1,2^, Zajíček, R. ^1,2^, Valenta, J. ^3^, Adam, L. ^4^

^1^ University Hospital Královské Vinohrady, Prague, Czech Republic, ^2^ Charles University, Third Faculty of Medicine, Prague, Czech Repulic, ^3^ Brno University of Technology, Faculty of Electrical Engineering and Communication, Brno, Czech Republic, ^4^ Adizo iMedia production, Prague, Czech Republic.

Poster presentations 1, 3 September 2025, 15:00–15:30

Aim:

White phosphorus (WP) is a highly reactive substance that poses significant risks to human health due to its incendiary and toxic properties.

Methods:

This presentation provides a detailed analysis of the devastating effects of white phosphorus (WP) on the human organism, focusing on the specific characteristics of deep penetrating burns. Unique high-speed camera footage will be used to demonstrate the penetration of WP into the human skin, revealing the mechanism of this deep and difficult-to-treat burn injury. Thermal imaging will then visualize the auto-ignition effect of WP and the associated complications in extinguishing the burning substance, a critical challenge in acute care management.

Conclusions:

White phosphorus is a highly dangerous chemical agent that can cause severe burns. As the global security situation continues to deteriorate, the likelihood of medical professionals being exposed to these types of injuries is increasing. By combining high-speed imaging, thermal analysis and toxicological data, we aim to improve understanding of the effects of white phosphorus on human health and contribute to improved treatment strategies.

P036

Deep burns treatment using enzymatic debridement with bromelain in combination with other modern devices—A clinical study.

**Tamas, C.** ^1^, Tecuceanu, A. ^1^, Hreniuc Jemnoschi, I. ^1^, Moraru, D. ^1^, Tataru, R. ^1^, Tamas, I.^1^, Stanescu, C. ^2^, Pintilie, C. ^1^

^1^ Grigore T Popa University of Medicine and Pharmacy, Iasi, Romania, ^2^ Dunarea de Jos University of Medicine and Pharmacy, Galati, Romania.

Poster presentations 1, 3 September 2025, 15:00–15:30

Aim:

To evaluate the clinical performance of enzymatic debridement with bromelain, in deep burns, in combination with negative pressure wound therapy (NPWT), or other devices (bacterial nanno-cellulose dressing, hydroactive compresses, dialkylcarbamoyl –chloride dressing).

Methods:

30 patients with 2ndB and 3rd degree thermal burns on 15–45% of total body surface area (TBSA) underwent enzymatic debridement; then, 15 patients received NPWT and the other 15 were treated with devices able to stimulate the healing process (bacterial nannocellulose dressing, hydroactive compresses, dialkylcarbamoyl –chloride dressing). We evaluated the thickness of burn wound, before and after enzymatic debridement, and the progression of the healing process using a Periscan Laser Doppler system.

Results:

Enzymatic debridement was efficient in all the cases. 14 patients with burns located on 20–45% TBSA needed skin grafts (micrografts in 12 cases, meshgrafts in 2 cases). The use of NPWT over the skin grafts stimulated the wound healing, reduced hospitalization days and the infection risk, as confirmed by bacteriological examination. This approach was more effective (healing daily rate of 4.54–3.98% from the initial burn area) compared to enzymatic debridement followed by other devices (healing daily rate of 2.67–2.38% from the initial burn area).

Conclusions:

The combination of bromelain with NPWT is an efficient alternative to surgical treatment, improving healing, reducing the need for skin grafts and pain level associated with dressing changes. Bromelain proved to be useful in extensive burns treatment, where skin-grafts donor zones reserves are limited.

P039

Follow-up treatment of alkali chemical burns with an enzyme-algino-gel containing an otolytic debridement pomade: Case Report.

**Tan Başer, N.** ^1^

^1^ University of Health Science, Ankara, Turkey

Poster presentations 1, 3 September 2025, 15:00–15:30

Aim:

Many industrial or domestic substances have the potential to cause chemical burns. Here we report a patient admitted to our centre with a chemical burn after contact with a cleaning product containing potassium hydroxide.

Methods:

A 71-year-old man was admitted to our centre 1 week ago after a cleaning product containing potassium hydroxide was spilled on his foot and the burns developed and the wounds deepened. The patient’s 3rd degree burn areas with 1% fibrinous fibrin on the right foot dorsum of the 2nd–5th toes were detected. Since the patient refused surgical debridement, daily dressing with enzyme algino-gel pomade with autolytic debridement was started.

Results:

In the first week, it was observed that the fibrin tissue on the wound surface started to decrease and granulation tissue from the base started to fill the cavity. At week 2, it was observed that the fibrin tissue in the wound had disappeared and healthy granulation tissue had formed. In a third-degree burn, the defect was repaired with a partial-thickness skin graft to prevent scar tissue formation.

Conclusions:

Alkalis tend to cause more damage than acids. Acids typically cause coagulation necrosis, which produces an eschar that limits further damage. Alkalis, on the other hand, cause liquefaction necrosis, which allows deeper damage to occur In the patient presented, the depth of the alkali burn was such that the tendons of the finger were exposed. Application of an enzyme-algino-gel pomade provided antimicrobial protection and created healthy granulation tissue in the wound ready for grafting.

P040

Post-operative wound care of micro-skin grafts

**Tantula, S.** ^1^, Laine, S. ^1^, Toivanen, I. ^1^, Ilmarinen, S. ^1^, Schepel, V. ^1^

^1^ Helsinki Burn Centre, Espoo, Finland

Poster presentations 1, 3 September 2025, 15:00–15:30

Post-operative wound care of micro-skin grafts

Tantula, S., Laine, S., Toivanen, I., Ilmarinen, S. and Schepel, V.

Aim:

Micro-skin grafting treatment with Meek technique is used among burn and plastic surgery patients who have limited donor sites. We herein describe the process of wound care performed by burn nurses for a patient who has undergone micro-skin grafting with the Meek technique. The purpose of this poster is to describe this process.

Methods:

During 2016–2023 severely burned patients received treatment with Meek technique microskin grafting in Helsinki Burn Centre. Meek technique method has seen a revival in recent years instead of traditional meshed skin grafting.

Micro-skin grafts are placed in the operating theatre. They are glued to the silk dressing that is fixed on the wound bed with surgical staples. Wound is covered with non-adherent absorbant wound dressings, which is changed daily.

The first dressing take down is carried out between the days 7–10 post-surgery. The burn nurse performs wound care by softening the wound bandage dressilk to avoid the risk of shearing the micro-skin graft that can cling tightly to the dressilk.

Results:

The presented patient case illustrates that with proper guidance burn nurses can perform appropriate wound care for micro-skin grafted patients without the risk of shearing of the microskin graft.

Conclusions:

Micro-skin grafting is a useful method for patients who have limited donor sites. With proper guidance and training of burn nurses the result can lead to a successful outcome.

P041

The novel concept using EDNX in delayed and fractional treatment for extensive burns.

**Taskov, P.** ^1,2^, Corodati, D. ^1^, Crainiceanu, Z. ^1,2^

^1^ Burn Unit County Emergency Clinical Hospital “Pius Branzeu”, Timisoara, Romania, ^2^ University of Medicine and Pharmacy “Victor Babes”, Timisoara, Romania.

Poster presentations 1, 3 September 2025, 15:00–15:30

Aim:

Delayed and fractional EDNX is a feasible and safe procedure for major burns.

Methods:

The retrospective comparative observational study included 50 patients, divided into two groups, first group 25 patients treated with EDNX < 72 h, medium 43.2% TBSA—13% TBSA EDNX treated and second group 25 patients treated with EDNX > 72 h, medium 40.2% TBSA—25.2% TBSA EDNX treated.

This novel concept was applied:

- up to ~ 50% TBSA
- up to 7 days after burn injury

Results:

The treatment was evaluated by monitoring effectiveness of debridement, spontaneous healing, primary coverage, surgical revisions after spontaneous healing or primary coverage, number of additional secondary surgeries, time required until complete epithelialization, hospitalization period.

Conclusions:

The availability of a reliable and complication-free enzymatic debridement without significant systemic effects could open new horizons in the treatment of severe burns.

This novel concept has shown promising results in burn healing process, effectiveness of debridement, reduce need for SOC, reduce hospitalization and healing time, earlier rehabilitation and improves quality of life.

Delayed and fractional EDNX is a real CHALLENGE as part of personalized care in burn surgery for major burns.

This concept will show its efficacy when higher standardization and experience is achieved.

P042

Our Experience in Burn Patients with Flaminal

**Turan, M.** ^1^

^1^ Istanbul City Hospital, Basaksehir. Istanbul, Turkey

Poster presentations 1, 3 September 2025, 15:00–15:30

Aim:

The purpose of this study was to assess the performance of Flaminal^®^ which consists of hydrated alginates polymers in a polyethyleneglycol matrix embedded with a biologic enzyme system based on glucose oxidase and lactoperoxidase. Enzyme system of this gel forms free radicals which destroy the cell wall of bacteria.

Methods:

Thirthy two patients with second degree burn participated in this study. involving 14 women and 18 men with a mean age of 46 years (range from 18 to 81).

Every day burn wounds of the patients were washed and cleaned with Actolind^®^ solution (Wietzen-Holte, German) and then Flaminal was applied to the wound of the patients and covered with a paraffin gauze dressing who were treated through a scheduled protocol and assessed at 3rd, 6th, 10th, 15th, 20th and 25th days.

Results:

Improvements to wound bed condition were reported after commencing initial treatment, with decreases in exudate, eschar, malodour and pain reported across the study. After 15 days, debritement of all the wounds finished. Eight of 32 (25%) of the patients needed surgical excision. 4 (12%) of the patients needed split thickness skin greft.

After 25 days, a pronounced healing of all treated wounds was noted. None of the patients experienced any adverse events related to the use of this agent.

Conclusions:

The Flaminal can be a suitable choice in the second degree burn wounds to enhance debridement and maintain healing and support granulation.

P043

Suprathel’s Usability and Effectiveness for the Treatment of Paediatric Partial-Thickness Burns: a 10 Year Retrospective Cohort Study.

**van de Warenburg, M.** ^1,2^, El Yadari, S. ^1,2^, Hummelink, S. ^2^, Ulrich, D. ^1,2^, Vehmeijer-Heeman, M. ^1,2^

^1^ Amalia Centre of Expertise Paediatric Trauma and Burns, Radboud University Medical Centre, Nijmegen, The Netherlands, Nijmegen, The Netherlands, ^2^ Department of Plastic, Reconstructive and Hand Surgery, Radboud University Medical Centre, Nijmegen, The Netherlands, Nijmegen, The Netherlands.

Poster presentations 1, 3 September 2025, 15:00–15:30

Suprathel has been introduced in our centre since 2014 for superficial to deep partial thickness burns. To evaluate healing outcomes and usability of Suprathel in our paediatric population, we conducted a retrospective cohort study, including all paediatric patients treated with Suprathel for burn wounds since its introduction. The studied population consisted of 92 patients, with a mean age of 4 years [3 months–17 years]. The median wound healing time for the entire cohort was 12.5 days, with no significant differences between the age groups. If wounds were found to be colonized during treatment, the majority of pathogens were *Staphylococcus aureus* (55.8%). Seven percent of the patients required a split-thickness skin graft after approximately ten days.

The use of Suprathel as epidermal dressing for treatment of partial thickness burns in the paediatric population demonstrates favourable adherence to the wound bed, reduces the need for frequent dressing changes, and the lack of increased wound infections underscore its benefits in paediatric burn care. Our findings suggest no significant differences in outcomes based on the timing of application, wound location, or wound bed preparation, and confirms its efficacy in mobile areas such as the hands.

P044

Reconstruction of various complex full-thickness skin defects with a biodegradable temporising matrix: a case series

**van Durme, J.** ^1^

^1^ Burn Center, University Hospital Ghent, Gent, Belgium

Poster presentations 1, 3 September 2025, 15:00–15:30

Aim:

BTM NovoSorb is a highly effective dermal substitute for complex full-thickness skin defects (FTSD) that warrants greater recognition and adoption across Europe.

Method:

This case series focused on treating complex FTSDs with BTM. After wound debridement, BTM was applied according to a defined protocol. Once adequate vascularization observed, the sealing membrane was removed and the neodermis was covered with split-thickness skin grafts (STSGs). Patient demographics, comorbidities, wound localization, etiology, wound bed preparations, BTM application and removal time, complete wound healing time after STSG, complications, and HTS formation were recorded.

Results:

BTM was used in six patients to treat complex FTSDs from degloving (3), burns (1), ulcerations (1), and necrotizing fasciitis (1). Successful integration occurred in five cases (83%), with one partial integration. BTM remained in situ for an average of 20.7 days before delamination and STSG coverage. No major complications occurred, though one case had hypergranulation with secondary STSG infection. Two patients were lost to follow-up, while the remaining four had excellent aesthetic and functional outcomes.

Conclusions:

This case series demonstrates that BTM is an effective and versatile solution for complex FTSD reconstruction, regardless of etiology. High integration success and minimal complications underscore its reliability and clinical utility. Consistent results with a standardized protocol support its reproducibility. These findings reinforce BTM as a safe, efficient option for high-quality neodermis formation, with potential long-term benefits. Further studies are needed to assess long-term outcomes and broader applications.

Keywords: wound reconstruction, full thickness skin defect, biodegradable temporising matrix, synthetic dermal substitute

P045

Does Enzymatic Debridement Reduce the Occurrence of Hypertrophic Scarring in Intermediate Depth Burns?

**van Durme, J.** ^1^

^1^ University Hospital Ghent, Gent, Belgium

Poster presentations 1, 3 September 2025, 15:00–15:30

Aim:

Hypertrophic scars (HTS) are a major concern after burns. While conservative treatment is the standard treatment for superficial burns and Nexobrid^®^ (EDNX) for deep burns, the optimal approach for intermediate depth burns remains unclear. This study aims to assess whether EDNX can reduce HTS risk in intermediate depth burns.

Methods:

Patients with intermediate depth burns (healing potential (HP) 14–21 days), assessed by LDI within 48 h to five days post-injury, were retrospectively analysed for HTS following either conservative treatment or EDNX. Regions of interest (ROIs) were analysed for flux values and surface area. Wound closure time and HTS formation at three different time frames: 3–6 months, 6–12 months, and 12–24 months post-injury, within the ROI were independently evaluated by two burn specialists.

Results:

In total, 98 ROIs were analyzed in 67 patients, with 46 ROIs treated conservatively and 52 ROIs treated with EDNX. HTS developed in 32.6% (15/46) of ROIs in the conservative group and 32.7% (17/53) in the EDNX group, showing no statistically significant difference between the two groups (*p* = 0.993). A statistically significant correlation was found between HTS formation and wound closure time (*p* = 0.001); however, no significant correlation was observed between HTS formation and flux values (*p* = 0.203).

Conclusions:

There was no significant difference in HTS prevalence between intermediate depth burns treated conservatively or with EDNX, indicating that conservative treatment remains the preferred approach.

P046

Enhanced Regeneration and Scar Modulation in Deep Dermal Burns via Fish-Derived Omega-3 Matrix Post-Enzymatic Debridement.

**Wallner, C.** ^1^, Holtermann, J. ^1^, Lehnhardt, M. ^1^

^1^ Bergmannsheil Bochum, Bochum, Germany

Poster presentations 1, 3 September 2025, 15:00–15:30

Aim:

This retrospective investigation evaluates the clinical efficacy of decellularized fish skin graft applied after enzymatic debridement in patients with deep dermal burns. The study compares healing time, scar quality, and functional skin outcomes with standard treatments, namely lactic acid membrane and split-thickness skin grafts (STSG).

Methods:

Fourteen patients with deep dermal burn injuries underwent enzymatic debridement followed by wound coverage with either decellularized fish skin graft, lactic acid membrane, or STSG. Wound healing progress was monitored for 12 months using both objective parameters (skin hydration, elasticity, sebum production) and subjective assessments (POSAS for scar evaluation, pain, and itch scores). Statistical significance was determined using ANOVA and Student’s *t*-tests.

Results:

Treatment with decellularized fish skin graft significantly accelerated reepithelialization (mean 22 days) compared to STSG (34.7 days) and lactic acid membrane (45.6 days). Scars in the decellularized fish skin graft group exhibited superior aesthetic and functional properties, including improved pliability, pigmentation, and thickness. Moreover, enhanced skin hydration and normalized sebum production were observed, indicating improved skin barrier restoration.

Conclusions:

The use of decellularized fish skin graft following enzymatic debridement leads to faster wound closure and superior scar outcomes compared to conventional treatments. These findings highlight the potential of Omega-3-rich matrices as a valuable adjunct in the treatment of deep dermal burns and support their broader integration into clinical practice. Further studies with larger cohorts are recommended to validate these results across diverse patient populations and wound types.

P049

Enzymatic Debridement (Nexobrid^®^) Enhances Epithelization of a Chronic Deep Burn Wound Unhealing for One Year: A Case Report

**Wu, C.** ^1^

^1^ Burn Center, Department of Surgery, China Medical University Hospital, Taichung, Taiwan

Poster presentations 1, 3 September 2025, 15:00–15:30

Aim:

To evaluate whether enzymatic debridement using Nexobrid^®^ can enhance epithelization in a delayed deep burn wound that remained unhealed for one year.

Methods:

A 26-year-old Asian male sustained severe burns following an explosion, resulting in 90% TBSA deep burn. Skin grafts were applied to high-priority areas, including the chest, neck, and bilateral groin, to prevent infection at central venous catheter insertion sites. The back skin defect was left unaddressed initially. In the ninth and tenth months following the injury, scalp skin grafts were applied to the back wound; however, both grafts failed to take, and the wound remained unhealed. Persistent MRSA infection was identified, which did not resolve even with teicoplanin treatment and continuous sodium hypochlorite irrigation. In the twelfth month post-injury, Nexobrid^®^ was applied for 2 h to debride the superficial slough and biofilm. Daily photographs of the wound were taken to monitor progress.

Results:

Enzymatic debridement effectively removed superficial slough, even though it was not visibly prominent. Although the wound size temporarily increased following debridement, noticeable improvements in epithelization were observed, and the wound began healing progressively over the following weeks.

Conclusions:

Biofilm, slough, and scar tissue serve as significant barriers to wound epithelization in chronic burn wounds. This case demonstrates that enzymatic debridement is a promising treatment option for promoting the healing of chronic burn wounds that have been unhealed for an extended period. The findings suggest that enzymatic debridement could offer a valuable alternative for challenging cases that fail to progress with conventional therapies.

P050

Comparative Study Between Meshed Grafts and MEEK Grafting for Small Surface Burns—Aesthetic and Functional Outcomes

Baetu-Dumitrascu, M., Pertea, M., **Alexandru, A.**, Benamor, M., Avadani, B.

^1^ ”Sf. Spiridon” Emergency Clinical County Hospital, Iasi, Romania

Poster presentations 1, 3 September 2025, 15:00–15:30

Aim:

Skin grafting is a key reconstructive method for burn injuries, with the goal of achieving optimal healing while maintaining functionality and aesthetic appearance. This study evaluates the aesthetic and functional outcomes of meshed grafting versus MEEK grafting in small surface burns (<10% total body surface area, TBSA).

Methods:

The study was conducted on 36 patients. Twenty patients received meshed split-thickness skin grafts (STSG), while 16 were treated with MEEK grafting. Outcomes were assessed at 3, 6, and 12 months postoperatively using: The Vancouver Scar Scale (VSS) for aesthetic outcomes, range of motion measurements for functional recovery, time to complete re-epithelialization.

Results:

The mean re-epithelialization time was significantly shorter in the meshed graft group (14 ± 3 days) compared to MEEK group (18 ± 4 days). Scar evaluation using the VSS at 6 months showed comparable pigmentation and vascularity between groups, but MEEK group exhibited a higher mean score for pliability and texture irregularity. Functional assessment demonstrated no significant differences across anatomical sites, although MEEK technique has a lower risk of scar contracture with more uniform healing and better sensitivity. Patient satisfaction was slightly higher in the meshed graft group mainly due to scar appearance concerns in MEEK group.

Conclusions:

Both meshed and MEEK grafting are effective techniques for small burns, offering comparable functional recovery and aesthetic. MEEK technique provides advantages in donor site preservation, uniform healing, reduced secondary contraction although meshed STSG might be better in areas requiring flexibility and in highly mobile areas.

P053

Reconstruction of Burn Contractures with Free and Perforator Flaps

**Canlar, S.** ^1^, Tapki, M. ^1^, Oz, K. ^1^, Karakol, P. ^1^

^1^ Health Sciences University, Basaksehir Cam and Sakura City Hospital, Istanbul, Turkey

Poster presentations 1, 3 September 2025, 15:00–15:30

Reconstruction of Burn Contractures with Free and Perforator Flaps

Aim:

This study aimed to highlight different free flap and perforator flap options in the treatment of burn contractures and to evaluate their functional outcomes.

Methods:

A retrospective review was conducted on 28 patients with burn contractures treated between January 2023–February 2024. Data collected included demographic characteristics, flap type used, duration of surgery and hospitalization, postoperative complications, and patient satisfaction during follow-up.

Results:

Twelve patients them reconstructed with free anterolateral thigh flap, ten with free superficial iliac artery perforatory flap, two of them with free profunda artery perforator flap, three of them thoracodorsal artery perforatory flap and one of them with ulnar artery perforatory flap. No major complications occurred. Minor complications such as wound dehiscence and hematoma were managed conservatively with close monitoring and secondary healing. All patients received postoperative physiotherapy for at least one month. Functional outcomes were assessed by comparing pre- and postoperative passive and active range of motion (ROM). A statistically significant improvement in joint mobility was observed in all patients (*p* < 0.05). Patient-reported satisfaction was high based on postoperative quality of life assessments.

Conclusions:

The reconstructive ladder remains a guiding principle in managing complex contractures, starting with less invasive techniques. However, advanced cases may require more complex reconstructive approaches to achieve optimal functional and aesthetic outcomes. Surgical technique selection should be individualized, considering factors such as contracture size and location, tissue condition after release, and the patient’s overall health status.

P054

CO_2_ laser treatment for burn scar sequelae: A prospective intrapatient pre-post intervention study

**Cerón Molina, F.** ^1^, Romero López, I. ^1^, Álvarez Hernández, P. ^1^, Anaya Pérez, P. ^1^, Martínez Méndez, J. ^1^, Leyva Rodríguez, F. ^1^

^1^ Hospital Universitario La Paz, Madrid, Spain

Poster presentations 1, 3 September 2025, 15:00–15:30

Aim:

To assess the clinical effectiveness of fractional CO2 laser therapy in the treatment of hypertrophic burn scars through a prospective intrapatient pre- and post-intervention design.

Methods:

This prospective study includes patients with hypertrophic scarring secondary to burns who are treated with fractional CO2 laser therapy. Each patient undergoes multiple sessions, with clinical evaluations performed before and after the intervention. Scar assessment tools such as the Vancouver Scar Scale (VSS) and patient-reported outcome measures are used to evaluate changes in scar appearance, texture, and function.

Results:

The study is expected to reveal improvements in scar quality following laser treatment. Changes in pigmentation, pliability, thickness, and overall appearance will be analyzed. Patient satisfaction and tolerance to the procedure will also be assessed to determine the impact of treatment on quality of life.

Conclusions:

CO_2_ laser therapy represents a promising tool for improving burn scar sequelae. This study aims to contribute valuable data to support its incorporation into multidisciplinary burn rehabilitation strategies. Final results may guide future protocols for individualized scar management.

P055

Evaluation of Facial Burns Treatment: Alloplastic Epidermal Substitute Mask for Recovery Without Aesthetic and Functional Sequelae.

**Covarrubias Rodriguez, J.** ^1^

^1^ Centro Nacional de Investigación y Cuidado de Quemaduras. Instituto Nacional de Rehabilitación, Ciudad de México, México.

Poster presentations 1, 3 September 2025, 15:00–15:30

Background: 

Facial burns are one of the most difficult challenges for plastic surgeons; the outcome of facial burns is integral to positive self-esteem. Therefore, acute and reconstructive treatment of facial burns requires methodical management with the ultimate goal of achieving optimal functional and aesthetic results. The psychological effects of facial scarring secondary to severe burns are alarming for patients. Although numerous reconstructive surgical procedures can reduce deformity and sequelae, patients with facial burns ultimately realize that they will not recover their original facial appearance.

Objective: 

To propose an alternative definitive treatment for facial burns to promote early epithelialization and reduce aesthetic and functional sequelae through the use of the Suprathel^®^ dressing.

Methods:

An observational, descriptive, retrospective study was conducted in patients with facial burns. Forty-two patients with severe facial burn injuries were analyzed from January 2024 to January 2025. The degree and duration of epithelialization, need for surgical intervention, and self-perception were assessed.

Results:

Reduction in the rate of surgical interventions in patients with facial burns treated with Suprathel^®^, reduction in epithelialization time and sequelae associated with facial burns, favoring self-perception and early social adaptation.

Discussion: 

Facial burns, due to their depth, require surgical interventions associated with the subsequent development of aesthetic and functional sequelae. This negatively impacts the physical, psychological, and social health of patients. Currently, with the use of new dressings such as Suprathel^®^ in facial burns, they promote early epithelialization, a lower rate of sequelae, interventions, and with better short-term aesthetic results.

P056

AI-powered chat-assistant vs. traditional FAQ pages: which provides more effective scar care guidance?

**Demarbaix, T.** ^1,3^, Maertens, K. ^1,2^, Anthonissen, M. ^1,4^, Van Daele, U. ^1,3^, Meirte, J. ^1,3^, Moortgat, P. ^1^

^1^ Oscare Npo, Merksem, Belgium, ^2^ Vrije Universiteit Brussel, Brussels, Belgium, ^3^ Universiteit Antwerpen, Antwerp, Belgium, ^4^ Katholieke Universiteit Leuven, Leuven, Belgium

Poster presentations 1, 3 September 2025, 15:00–15:30

Aim:

To evaluate whether an AI-powered chat-assistant provides scar care guidance more effectively regarding efficiency, accuracy, and user satisfaction than traditional FAQ-pages on a specialized scar aftercare platform.

Method:

In this cross-sectional study, 10 participants were asked to find information on a specialized scar aftercare website regarding topics such as scar types, microneedling, or post-surgical care. They were randomly assigned to an AI or traditional search group. The AI group interacted with an AI-powered chat-assistant utilizing the website’s content and a generative pretrained transformer model, whereas the traditional group manually searched FAQ-pages. Outcome measures included time taken to obtain satisfactory answers for 6 questions, accuracy and relevance of information, and user satisfaction rated on a 5-point Likert scale. Demographic data, such as age, education level, technological proficiency, frequency of technology use, and previous chatbot experience, were collected.

Results:

The sample (*n* = 10) consisted of 30% aged 18–24, 40% aged 25–34, 20% aged 45–54, and 10% aged 55–64. Educational background showed 30% with a high-school diploma and 70% with higher education. Technological experience varied: 30% reported limited tech-skills without previous chatbot experience, while 70% demonstrated moderate-to-high proficiency with prior chatbot exposure. Participants using the AI chat-assistant completed tasks faster (mean time: 603.8 s vs. 874.0 s), though accuracy was comparable between groups. The AI group reported significantly greater ease of use (*p* = 0.028). Higher technological proficiency and prior chatbot experience correlated significantly with improved performance (*p* = 0.015).

Conclusions:

These preliminary findings suggest potential advantages of an AI chat-assistant for scar aftercare guidance.

P058

Fat grafting for hypertrophic scars: evolution and our experience

**García García, M.** ^1^, Bayo, M. ^1^, Guijarro, C. ^1^, Pérez, M. ^1^

^1^ Hospital Universitario Y Politécnico La Fe, Valencia, Valencia, Spain

Poster presentations 1, 3 September 2025, 15:00–15:30

Aim:

Hypertrophic scars are one of the most common and potentially devastating sequelae of burns. Currently, fat grafting is considered a useful treatment for scars.

Methods:

We reviewed recent literature on fat grafting for hypertrophic scars and the effects of adipose stromal vascular fraction cells. Additionally, we revised our case series and present a sample of patients who sustained burns and developed hypertrophic scars, all of whom were treated with fat grafting.

Results:

The initial treatment of hypertrophic scars is conservative. As a second-line treatment, autologous fat grafting (lipofilling) is commonly used, often in combination with intralesional corticosteroids (triamcinolone). Adipose tissue is one of the largest sources of adult stem cells, which have shown multiple beneficial effects, such as inhibiting fibroblast proliferation and migration to the hypertrophic scar as well as reducing the expression of inflammatory cytokines, among others. In our Burns Center’s case series over the last 10 years, fat grafting was used in 45 patients. Microfat (decantation and centrifugation) was used in 25 patients, while nanofat (Lipocube^®^) was used in 20 patients. 93.3% of all patients suffered thermal burns (75.5% from fire and 17.7% from liquids). Only 3 patients needed reintervention to improve scar appearance and alleviate negative symptoms, such as itching and tightness.

Conclusions:

Fat grafting significantly improves hypertrophic scars by promoting healing, reducing size, and enhancing skin texture and appearance. In recent years, nanofat has trended over microfat, although both are effective and offer distinct benefits for scar appearance and related effects.

P059

Improved cosmetic appearance of a modified cultured epidermal autograft compared to traditional skin autograft: A case report.

**Kleintjes, W.** ^1,2^, Prinsloo, T. ^3,4^

^1^ Department of Surgical Sciences, Stellenbosch University (Medical School), Cape Town, South Africa, ^2^ Western Cape Provincial Adult Tertiary Burns Centre, Tygerberg Hospital, Cape Town, South Africa, ^3^ Department of Biomedical Sciences, Cape Peninsula University of Technology, Cape Town, South Africa, ^4^ Department of Emergency Medical Sciences, Cape Peninsula University of Technology, Cape Town, South Africa.

Poster presentations 1, 3 September 2025, 15:00–15:30

Aim:

Cultured epidermal autografts (CEA) face logistical, cost, and handling challenges; limiting their use to life-saving burn treatment. Consequently, cases for cosmesis remain scarce despite its superior aesthetic outcomes. A modified, low-cost CEA technique was developed in a resource-limited setting to treat burn patients with poor prognoses, potentially surpassing traditional autografts for those prioritizing wound appearance. This study compares the cosmetic outcomes of conventional autografts with the modified Kleintjes cultured skin (KCS).

Methods:

The KCS technique included immersing epidermal fragments, initially separated from a 3 × 2 cm full-thickness skin biopsy, in trypsin for 2 h. The resultant keratinocytes were seeded onto routinely-used dressing and incubated in pediatric incubators at 37 °C for 2 weeks until confluence. Supplementation included daily application of fresh autologous plasma and Intrasite Gel^®^ (Smith & Nephew, London, UK) every 3–4 days. Xenografts, used during the culture period, were removed and the KCS-containing dressing was transplanted directly onto the debrided wounds of the right forearm and the traditional autografts onto the left. Both forearms initially had deep partial-thickness flame burns.

Results:

Compared to skin autografts, short-term healing (2 weeks post-transplant) with KCS demonstrated less scabbing and, after 1 month, appeared similar to the autograft. However, long-term KCS healing (1 year post-transplant) demonstrated more pliability, secondary hair growth and a smoother appearance.

Conclusions:

These favourable outcomes related to CEA appearance aligned with previous limited reports, supporting KCS’s potential for broader cosmetic applications. Further studies are needed to validate findings across different populations, burn depths, and comorbidities, as well as to comparing with other CEAs.

P060

Use of enzymatic alginogel for optimizing surgical area delimitation in second-degree intermediate burns after enzymatic debridement.

Minguez Abia, S. ^1^, **Santin Perez, N.** ^1^, Martin Playa, P. ^1^

^1^ Osakidetza—Hospital De Cruces, Cruces—Barakaldo, Spain

Poster presentations 1, 3 September 2025, 15:00–15:30

Aim:

To evaluate the use of enzymatic alginogel after selective enzymatic debridement in second-degree intermediate burns to better define the burn areas requiring grafts, thus optimizing surgical coverage.

Methods:

An observational study was conducted in patients with burns treated with enzymatic debridement. In cases with uncertainty regarding the burn’s ability to epithelialize, enzymatic alginogel was applied every 48 h during the first week and every 72 to 96 h during the second week. Parameters such as burn extension, grafted area, epithelialization time, and potential complications were analyzed.

Results:

In deeper burn areas, after 4 days of enzymatic alginogel application, a pseudoeschar formed, which was easily identifiable and began to detach spontaneously from the 7th day, facilitating the delimitation of the deeper zone and reducing the grafted area compared to the initial assessment. In previously doubtful areas that did not form a pseudoeschar, favorable evolution was observed with spontaneous epithelialization and a lower complication rate.

Conclusions:

In our experience, the use of Enzymatic Alginogel after Enzymatic Debridement allows for better identification of areas requiring surgical intervention, avoiding grafting areas with epithelialization potential, and promoting epithelialization in viable areas with a lower complication rate.

P061

Pure Skin Perforator (PSP) Flaps in Burn Reconstruction

**Orois Gómez, S.** ^1^, Rivas Nicolls, D. ^1^, Serracanta Domenech, J. ^1^, Bulla, A. ^1^, López Martínez, J. ^1^, Iglesias Álvarez, A. ^1^, González Peña, F. ^1^, Cuadros Ramírez, M. ^1^, Barret Nerin, J. ^1,2^

^1^ Vall d’Hebron Barcelona Hospital Campus, Barcelona, Spain, ^2^ Department of Surgery, School of Medicine, Universitat Autónoma de Barcelona, Barcelona, Spain

Poster presentations 1, 3 September 2025, 15:00–15:30

Aim:

To collect the data from the reconstructive surgeries based on PSP flaps for burn patients conducted in the past five years in Vall d’Hebron Burn Unit. To propose PSP flaps as an alternative for said interventions.

Methods:

Twenty-three PSP flaps were performed on 21 patients at our Unit from October 2020 to February 2025. Eight patients were treated for acute burns while the other 15 were scheduled to treat sequelae. Ages ranged between 15 and 75. The data collected were as follows: demography, flap design and surgical details, as well as postoperative outcomes and following surgeries.

Results:

Regarding the 23 flaps harvested, 21 of them were based on perforators from the SCIA, one was an ALT PSP and one was an MSAP PSP. Four needed urgent exploration during recovery: three were fully restored—two showed arterial thrombosis and one suffered an underlying haematoma-, whereas one experienced total necrosis and loss of the flap due to venous thrombosis. Moreover, the reported medical complications include four cases of venous congestion (two improved spontaneously, one with serial local injections of heparin and the latter suffered partial necrosis despite the local heparin injections), one case of transitory facial palsy and one case of wound dehiscence.

Conclusions:

PSP flaps could be proposed as a reliable one-step procedure for treating acute small deep burns and their sequelae in demanding areas since they provide pliable and elastic skin.

P062

Introducing the modified Meek grafting technique in the burn service: a paradigm shift for (not only) extensive burns.

**Ligomenou, T.** ^1^, Tsilingeridis, S. ^1^, Petsa, A. ^1^, Nikolaidou, E. ^1^, Tzimorota, Z. ^1^, Papadopoulou, S. ^1^

^1^ Department of Plastic and Hand Surgery & Burns ICU, “G. Papanikolaou” Hospital, Thessaloniki, Greece.

Poster presentations 1, 3 September 2025, 15:00–15:30

Aim:

Purpose of this study is to present our recent experience and learning curve during the first year of implementation of Meek technique in our Burn Center in patients with extensive burns and skin defects.

Methods:

We conducted a retrospective analysis of patients who underwent Meek grafting during 2024. Demographics, burn characteristics, clinical course, operative management and surgery duration were retrieved and analyzed from patient records. Outcome measures, including graft take rate, complications and need for further surgery, were recorded.

Results:

Ten patients with a mean age of 57 years (range 25–92) and mean TBSA 44% (range 7–85%) underwent Meek grafting in 13 surgical procedures. In 5 cases the micrografts followed enzymatic debridement, whereas in 2 cases they covered areas with dermal substitutes. In 2 cases the donor area was also “boosted” with Meek grafts. Approximately 90% of the transplanted areas healed satisfactorily and did not require re-grafting. In the remaining 10% infection was the main cause of graft failure.

Conclusions:

Meshed autologous split-thickness skin grafts remain the standard for extensive skin defect coverage, but their use is limited by donor site availability. The Meek technique offers an effective alternative, allowing up to 1:9 skin expansion while requiring a smaller donor area. Moreover, it may benefit smaller burns by minimizing donor site morbidity. To optimize outcomes, surgical team coordination and workflow adjustments are essential to prevent prolonged procedures.

P064

Burn injury of thorax—aspects of a surgery

**Stritar, A.** ^1^, Fortuna, K. ^1^, Lovšin, K. ^1^

^1^ UMC Ljubljana, Ljubljana, Slovenia

Poster presentations 1, 3 September 2025, 15:00–15:30

Burn injury of thorax—aspects of a surgery

Aim:

Circumferential burn injuries of the thorax can disrupt respiration and blood circulation. Therefore deep burns of thoracic wall require escharotomy.

Methods:

The pressure of eschara is released by several incisions. Later, in females burn excisions must be very careful and conservative. The defects should be covered with as large grafts as possible to reduce scar tissue to a minimum. Following these principles, the breast will grow almost normally if the gland is not damaged by the original trauma. In any case, abdominal pressure must be monitored.

Results:

When the breast is fully grown, secondary scar revision will be necessary if needed. In case of a breast deformity it can be corrected by a later reconstruction with regard to scar and gland. In females, disfigurement of a breast is remodelled by the principles of mammary breast reconstruction. In males, nipple areola reconstruction is sometimes demanded.

Conclusions:

Modern technique of breast expander and successive breast implant maintain good results as symmetrization and lipofilling, too. Our approach for removing the hypertrophic scarring in males is with the use of Integra dermal matrix followed by skin grafting.

P065

Microneedling treatment at the Helsinki Burn Centre

**Tiimo, P.** ^1^

^1^ HUS, Espoo, Finland

Poster presentations 1, 3 September 2025, 15:00–15:30

Aim:

The purpose of this work is to present microneedling as part of scar treatment at the Helsinki Burn Centre since 2023.

Methods:

The microneedling decision is made together with the patient and doctor at the reception.

The treatment process includes vitamin A-E-C oil, which is used three weeks before and throughout the treatment.

Needling is done approximately every 1–4 weeks, depending on the length of the needle and the recovery of the scar between needling. The needles used are 1–3 mm in length.

Continuation of needling is based, among other things, on the patient’s experience of an increase in the elasticity of the scar, a decrease in possible itching and active redness. During treatment visits, photos are also taken, from which the situation can be compared with the previous one.

Results:

Since 2023, there have been 7 burn scar patients, and 5 other scar patients that have been treated with microneedling. Each patient has received 1–7 treatments. For 7 patients the treatment is still ongoing.

Conclusions:

In two years, microneedling has become a viable alternative in the treatment of burn or other plastic surgery scars as part of other scar treatment or as an independent method. Needling is provided mainly by a nurse. POSAS and SCAR-Q are planned to be added to the pre- and post-assessment of scars in the future.

P100

Clinical Experience with Electrical Burns: A Retrospective Analysis from a Tertiary Burn Center

**Akın, M.** ^1^, Sarı, A. ^1^, Akgün, A. ^1^

^1^ Ankara Bilkent City Hospital, Ankara, Turkey

Poster presentations 2, 4 September 2025, 10:00–10:30

Aim:

Electrical injuries represent a complex form of trauma, often leading to deep tissue damage, amputations, and multi-organ complications. This study aims to present our clinical experience with patients treated for electrical injuries in a tertiary burn center.

Methods:

A retrospective review was conducted of patients admitted to the Burn Treatment Center of Ankara Bilkent City Hospital between 2020 and 2024.Data including age, sex, TBSA, surgical interventions, and mortality were collected from electronic records of patients with electrical injuries.

Results:

Of 426 burn patients admitted to the ICU, 88 (20.6%) had electrical injuries. High-voltage exposure was present in 62 (70.4%) patients. The mean age was 34.8 years, and the average TBSA was 10.7%. A total of 532 surgical procedures were performed. Amputations were required in 18 patients, including 8 below-knee, 4 forearm, and 1 above-knee amputation, in addition to various finger and toe amputations, totaling 20 amputations. Skin grafting was performed in 56 patients. Two patients (2.3%) died. The average length of hospital stay was 29 days (range: 1–170 days).

Conclusions:

Electrical injuries, particularly from high-voltage sources, often require multiple surgical interventions and prolonged hospitalization. The mean TBSA affected in our series was 10.7%, yet the severity of electrical injuries often extends beyond superficial estimations of burn surface area. Deep tissue involvement, compartment syndromes, and vascular compromise frequently necessitate aggressive surgical management. Despite the severity of the injuries, the mortality rate in our series was relatively low (2.3%), which may reflect the benefit of early intensive care unit (ICU) management and multidisciplinary burn care, including timely surgical intervention and infection control. In light of these findings, early multidisciplinary management is crucial to reduce morbidity and improve functional outcomes.

P101

Factors affecting burn injury outcomes in a Hispanic cohort: a retrospective study

**Aponte Rivera, H.** ^1,2^, Davila Perez, P. ^1^, Serpa Irizarry, M. ^1,2^, Ramos Sanchez, R. ^1^, Pomales Diaz, G. ^1^, Sotomayor Rivera, C. ^1^, Aymat Sanchez-Vahamonde, S. ^3^, Bonilla Robles, J. ^1,2^, Lopez Torres, G. ^1^, Rivera Barrios, A. ^1,2,4^

^1^ University of Puerto Rico (UPR)—Medical Sciences Campus, San Juan, Puerto Rico, ^2^ UPR—Department of Surgery, San Juan, Puerto Rico, ^3^ Universidad Central del Caribe—School of Medicine, Bayamon, Puerto Rico, ^4^ UPR—Plastic Surgery Division, San Juan, Puerto Rico.

Poster presentations 2, 4 September 2025, 10:00–10:30

Aim:

This study aims to explore the relationships between key clinical variables and health outcomes in patients treated for burn injuries in the acute care setting of the only trauma center in Puerto Rico.

Methods:

A retrospective analysis was conducted on a dataset of 116 burn patients from Jan 2018 to May 2022. Statistical tests, including Chi-Square analysis, T-tests, and ANOVA, were used to examine associations between patient demographics, TBSA, timing of first surgical intervention, need for mechanical ventilation, COVID-19 status, and survival outcomes, among others.

Results:

Our findings indicate that TBSA percentage has a statistically significant relationship with patient mortality (*p* < 0.01), suggesting that larger burn surface areas correlate with poorer outcomes. However, timing of first surgery did not show a statistically significant impact on survival rates. Additionally, no significant association was found between COVID-19 status and mortality. Nonetheless, the presence of inhalation injury and mechanical ventilation use were associated with increased mortality, highlighting their role as critical factors in burn patient prognosis.

Conclusions:

This study underscores the importance of TBSA, inhalation injury, and mechanical ventilation use as predictors of burn patient outcomes, while also questioning the assumed impact of early surgical intervention on survival. The lack of a strong association between COVID-19 status and mortality suggests that burn care outcomes may not have been significantly affected by the pandemic in this cohort. Further research with larger, multi-center datasets is recommended to confirm these findings and improve burn management strategies.

P102

Three years of “BUMP” Score in Clinical Practice: Evaluating Its Applicability in German Burn Intensive Care Units.

**Bagheri, M.** ^1,3^, Schiefer, J. ^2^, Lefering, R. ^3^, Fuchs, P. ^2^

^1^ Helios Klinikum Emil von Behring, Berlin, Germany, ^2^ Clinic of Plastic, Reconstructive, Hand and Burn Surgery, Hospital Cologne Merheim, Cologne, Germany, ^3^ Institute for Research in Operative Medicine (IFOM), Faculty of Health, Witten/Herdecke University, Cologne, Germany.

Poster presentations 2, 4 September 2025, 10:00–10:30

Aim:

Accurate mortality prediction for burn patients is crucial for optimizing treatment strategies in intensive care settings. We developed the Burn Mortality Prediction (BUMP) Score to improve prognostic accuracy in 2022. This invesstigation aims to assess its applicability in burn intensive care units (ICUs) in Germany.

Methods:

A survey was conducted among burn centers in Germany to evaluate the implementation and clinical use of the BUMP Score. Additionally, existing literature on the BUMP Score was analyzed, including its citation history and comparative performance against other scoring systems.

Results:

The survey results provide insights into the current utilization of the BUMP Score in clinical practice, highlighting its perceived advantages and limitations. Preliminary findings indicate that the majority of centers are familiar with the BUMP Score, while only a few actively implement it in clinical decision-making. The literature review confirms the superior predictive accuracy of the BUMP Score compared to traditional burn mortality scoring systems, with one study (PMID: 36231617) explicitly concluding that it outperforms traditional burn mortality prediction models.

Conclusions:

The BUMP Score is a promising tool for mortality prediction in burn patients. Its widespread adoption and acceptance in burn ICUs could enhance clinical decision-making and improve patient care. However, further evaluation is needed to assess its applicability in different settings.

P103

Teaching for the future: developing a weekly, cross-site, multidisciplinary, hybrid burns teaching programme.

**Beese, M.** ^1^, Liu, X. ^1^, Ma, Y. ^1^, Park, J. ^1^, Jackowski, A. ^1^, D’Asta, F. ^2^, Bache, S. ^1,2^

^1^ Burns Centre, Queen Elizabeth Hospital, Birmingham, United Kingdom, ^2^ Paediatric Burns Centre, Birmingham Children’s Hospital, Birmingham, United Kingdom.

Poster presentations 2, 4 September 2025, 10:00–10:30

Aim:

To describe the design and implementation of a multidisciplinary team (MDT), hybrid format, curriculum-based teaching programme suitable for cross-site burns education.

Methods:

A weekly teaching session was devised to facilitate meeting and learning between the adult and paediatric burns centres in Birmingham. This was both face-to-face and online. A dedicated e-mail address was used to distribute weekly emails. Topics were based on fifty topics from the Intercollegiate Surgical Curriculum Programme (ISCP) Burns section of the Plastic surgery curriculum, with special interest topics. A QR code for feedback surveys facilitated development of the programme.

Results:

Over a 12-month period, 42 teaching sessions were delivered and attended by MDT members, including consultants (7%), resident doctors (14%), nurses (36%), therapists (21.4%) and other allied health professionals (10.7%). The programme was well-received, with 96.4% finding the teaching well organised and engaging. Additionally, 85.7% agreed that the teaching programme fostered team building across sites, 85.7% felt it positively contributed to their work, 82.1% reported increased confidence in burns management, and 100% recommended the programme for its multidisciplinary relevance and impact.

Conclusions:

The development of a hybrid teaching programme provided all members of staff teaching time, self-development and an opportunity to learn from each other. Using technologies including online platforms, QR code links and live feedback forms, this has created a modern programme for today’s burns MDT. We offer tips for those wishing to set up similar initiatives.

P104

Therapeutic Effects of Propranolol on Post-Burn Cardiac Dysfunction in a Rat Scald Model

**Brandt, J.** ^1,2^, Niederegger, T. ^1^, Mertin, V. ^1^, Ritterhoff, J. ^1^, Trogisch, F. ^3^, Heineke, J. ^3^, Most, P. ^1^, Thiele, P. ^1^, Schulte, M. ^2^, Foundas, H. ^1^, Schaschinger, T. ^2^, Panayi, A. ^2^, Palackic, A. ^2^, Kneser, U.^2^, Hundeshagen, G. ^2^

^1^ Molecular and Translational Cardiology Division, University Hospital Heidelberg, Heidelberg, Germany, ^2^ Department of Hand, Plastic and Reconstruction Surgery, BGU Ludwigshafen, Ludwigshafen, Germany, ^3^ Department of Cardiovascular Physiology, Medical Faculty Mannheim, Heidelberg University, Mannheim, Germany

Poster presentations 2, 4 September 2025, 10:00–10:30

Aim:

To investigate the mechanistic effects of the non-selective beta-blocker propranolol on hypermetabolism and post-burn cardiac dysfunction using a rat scald model.

Methods:

Adolescent male Sprague-Dawley rats received a 60% TBSA full-thickness scald injury. Animals were randomly assigned to sham/vehicle (SV), burn/vehicle (BV), sham/propranolol (SP), and burn/propranolol (BP) groups.

Propranolol (c = 0.125 mg/hr) or vehicle (saline) was administered via intraperitoneal osmotic pumps starting on postoperative day 3. Analyses were performed on days 3, 7, and 30 (*n* = 6–8 per group per timepoint).

Results:

Burn injury (BV, day 30) caused significant weight loss (SV vs. BV: –21.26%) and muscle atrophy (–19.87%), indicating a hypermetabolic state. Heart rate was reduced in BP vs. BV (–12.57%).

Echocardiography showed reduced global longitudinal strain in BV, which improved in BP (BV vs. BP: +47.83%).

Expression of inflammatory (S100A8: 39.2-fold increase in BV vs. SV) and fibrotic markers (Col1: 1.71-fold higher in SV vs. BV) was altered, with no significant effects of propranolol at the transcript level.

At the protein level, troponin I Ser23/24 phosphorylation was elevated in BV vs. SV (+2.89-fold) and reduced in BP vs. BV (–0.37-fold). Phospholamban phosphorylation at Ser16 was 1.5-fold higher in BV vs. SV and decreased to 0.1-fold in BP vs. BV, suggesting normalized β-adrenergic signaling after propranolol administration.

Conclusions:

Propranolol attenuates post-burn cardiac dysfunction by improving myocardial strain and modulating β-adrenergic signaling, supporting its potential as a therapeutic strategy in burn care.

P105

When the liver fails: A study of coagulopathy and burn wound outcomes in chronic alcohol consumers.

**Bulgaru Iliescu, A.** ^1,2^, Amarandei, A. ^1^, Benamor, M. ^1^, Moraru, D. ^1,2^, Susanu, S. ^3^, Pertea, M ^1,2^

^1^ St. Spiridon Emergency Hospital Iasi, Iasi, Romania, ^2^ University of Medicine and Pharmacy Grigore T.Popa Iasi, Iasi, Iasi, ^3^ St Mary Emergency Children Hospital, Iasi.

Poster presentations 2, 4 September 2025, 10:00–10:30

Aim:

This study aims to evaluate how coagulopathy resulting from hepatic dysfunction in chronic alcohol consumers affects burn wound healing, focusing on hemostatic disturbances, inflammatory responses, and clinical outcomes.

Methods:

A retrospective observational study was conducted on 50 burn patients with second and third degree burns, admitted between January 2020 and December 2024. Inclusion criteria: age between 18–65 years, confirmed history of chronic alcohol consumption (>40 g/day for >5 years), TBSA of 10–40%. Exclusion criteria: patients with pre-existing coagulopathies unrelated to hepatic dysfunction, immunosuppressive therapy, or other comorbidities (diabetes mellitus, chronic kidney disease). Patients were divided in Group A (*n* = 25): Chronic alcohol consumers with hepatic dysfunction (based on liver function tests, imaging, and clinical diagnosis) and Group B (*n* = 25): Non-alcoholic burn patients without hepatic dysfunction. Outcomes measured included coagulation profiles (INR, fibrinogen, platelet count), post-burn sepsis, wound healing time, and 30-day mortality.

Results:

The study group included both male and female patients, with a ratio of 3:1. Results showed that Group A had significantly higher INR levels (2.1 vs. 1.1; *p* < 0.001), lower fibrinogen levels (130 mg/dL vs. 240 mg/dL; *p* < 0.01), and thrombocytopenia (95,000/μL vs. 175,000/μL; *p* < 0.001). Infection rates were higher in Group A (60% vs. 28%; *p* = 0.02), and wound healing time was prolonged (36 days vs. 24 days; *p* < 0.001). Group A also demonstrated higher 30-day mortality (20% vs. 4%; *p* = 0.04).

Conclusions:

Hepatic dysfunction-related coagulopathy significantly hinders burn wound healing in chronic alcohol consumers. Early intervention focusing on hemostatic optimization, nutritional support, and infection control is critical in managing these patients effectively.

P106

Profile of Chemical Burn Injuries in the UAE: Insights from a Regional Tertiary Centre

Curtis, E. ^1^, **Debal, B.** ^1^, Browne, R. ^1^, Pierce, D. ^1^, Afrooz, I. ^1^

^1^ Sheikh Shakhbout Medical City, Abu Dhabi, United Arab Emirates

Poster presentations 2, 4 September 2025, 10:00–10:30

Aim:

Chemical burns are a clinically complex subset of soft tissue injuries, often requiring prolonged hospitalization and surgical intervention. This study evaluates the demographic and clinical profile of chemical burn patients managed at Sheikh Shakhbout Medical City; a tertiary referral burns centre for burns in the UAE.

Methods:

A retrospective review was conducted of all patients admitted with chemical injury between 1 January 2023, and 31 December 2024 (*n* = 46). Data on age, gender, TBSA, causative agents, burn depth, and grafting procedures were analysed.

Results:

Of 46 patients, 89.1% were male. The mean TBSA was 6.88%, with adult males showing the highest TBSA (8.13%) and adult females the lowest (3.83%). Paediatric patients (<16 years) comprised 10.8%, with a mean TBSA of 6%. Occupational injuries (*n* = 10) had significantly greater TBSA (15.1%) than domestic burns (4.74%). Acid agents, mainly drain cleaners, accounted for 69.5% of burns. Second-degree burns were most common (72%). Allografts were used in 13% of cases, primarily in adult males. Autografts (split-thickness skin grafts) were performed in 50% of patients, most frequently in adult males (39%) and paediatric males (33%); none were performed in adult females. Greater TBSA correlated with higher grafting needs.

Conclusions:

Chemical burns primarily affected adult males and were more extensive in occupational settings. The pattern of TBSA distribution and surgical intervention aligns with international literature. Continued work in education recommendations regarding protective equipment in high-risk occupations and when utilizing drain cleaners in domestic settings may limit these injuries in UAE.

P107

Nexobrid Versus Tangential Escariectomy: Mortality Rates and Overall Survival in Severely Burned Patients Admitted over the Last Ten Years at the Bari Burn Center

**Di Turi, C.** ^1^, Bruno, S. ^1^, Di Gioia, G. ^1^, Tedeschi, P. ^1^, De Cosmo, A. ^1^, Maggio, G. ^1^

^1^ UOSD Burn Center, Department of Precision and Regenerative Medicine, University of Bari Aldo Moro, AOC Policlinico Bari, Bari, Italy

Poster presentations 2, 4 September 2025, 10:00–10:30

Aim:

Deep and intermediate-deep burn treatment has significantly evolved due to the introduction of modern, less invasive techniques. Although tangential escarectomy remains widely used, early selective enzymatic escarolysis with bromelain “Nexobrid” has revolutionized the treatment of severely burned patients. Nexobrid uses an enzymatic gel that selectively dissolves necrotic tissue while preserving viable dermal layers, promoting spontaneous healing. This study aimed to compare mortality rates and overall survival (primary outcomes) and identify factors influencing hospital stay duration (secondary outcome) between patients treated with traditional surgery and those treated with early selective enzymatic escarolysis (Nexobrid).

Methods:

A retrospective cohort study was conducted on 179 patients admitted to the Bari Burn Center between 2014 and 2023, divided into two cohorts: 101 patients treated with surgery (2014–2018) and 78 treated with Nexobrid (2019–2023). Statistical analysis was performed to compare mortality rates, overall survival, and hospital stay duration between the groups.

Results:

The analysis revealed lower mortality rates and higher survival in the Nexobrid group. No significant difference in hospital stay was found between the two treatments.

Conclusions:

Nexobrid demonstrated significant improvements in survival and mortality rates over traditional surgery, without extending hospital stay. It represents a vital innovation in burn management, offering early, selective necrosis removal, reduced surgical interventions, and improved healing.

P108

Physical Findings as Early Diagnostic Measures for Inhalation Injury in Burn Patients to Indicate Early Intubation: A Systematic Review and Meta-Analysis.

**Farhana, N.** ^1^, Wardhana, A. ^1^

^1^ Division Of Plastic Reconstructive and Aesthetic Surgery, Jakarta, Indonesia

Poster presentations 2, 4 September 2025, 10:00–10:30

Aim:

The need of early intubation in burn patients with suspected inhalation injury remains uncertain. However, unnecessary intubated patient can lead to ventilator-associated-respiratory complications. Bronchoscopy, as the gold standard for diagnosing inhalation injury in burn patients, is inaccessible in some hospitals in Jakarta, posing a challenge to diagnosing inhalation injuries in developing countries, such as Indonesia. This paper aims to review the early diagnosis of inhalation injury in burn patients to determine the indication of early intubation.

Methods:

Literature review was conducted using the terms of “inhalation injury”, “burns”, and “intubation”. Pubmed, Cohrane, and Science Direct were the online data bases utilized. We identified four studies evaluating the indication of early intubation in burn patients with suspected inhalation injury. The risk of bias was assessed using QUADAS-2 tool. Pooled sensitivity, specificity, and diagnostic odds ratios, along with their corresponding 95% confidence intervals, were calculated for each physical finding and the reported clinical decision aids.

Results:

Ten physical findings for inhalation injury were analyzed and ranked by diagnostic odds ratio (DOR). Most findings showed high specificity and low sensitivity. The pooled DOR ranged from 0.93 to 7.50. Dyspneu had the highest DOR of 7.50 [0.48; 119.17] (sensitivity 0.20, specificity 0.97), while singed nasal hair had the lowest DOR of 0.93 [0.09; 9.71]. Dyspneu, stridor, and hoarseness exhibited the highest DOR among other physical findings.

Conclusions:

Physical findings with the highest diagnostic accuracy for inhalation injury may help determine the need for early intubation.

P109

Hypercalcemia and Renal Failure in Patients with Severe Burns: An Overlooked Medical Conditions. Electrolyte Imbalances in Burn Patients

**Filaj, V.** ^1,2^, Bazo, M. ^2^, Tabaku, E. ^1^, Kola, I., Tafilaj, V. ^1^

^1^ University of Medicine, Tirana, Albania, Tirana, Albania, ^2^ University Hospital Center “Mother Teresa”, Tirana, Albania

Poster presentations 2, 4 September 2025, 10:00–10:30

Introduction: A 16-month review at the burn unit of Mother Teresa Hospital in Tirana, Albania, revealed an unexpected occurrence of hypercalcemia among burn patients, particularly those hospitalized for more than 3 weeks. This condition was more common in patients with renal failure and those with prolonged immobilization, and higher calcium levels were linked to increased mortality rates.

Purpose: This study explores the relationship between hypercalcemia, renal failure, and electrolyte imbalances in burn patients, advocating for better recognition and management of these conditions to improve patient outcomes.

Methods:

A retrospective cohort study of 217 patients from the Department of Burns and Plastic Surgery at Mother Teresa Hospital, Tirana, analyzed calcium and electrolyte levels from January 2023 to April 2024. The study included patients with burns covering >20% of body surface area (BSA) or hospital stays >10 days. Hypercalcemia was defined as ionized calcium >1.22 mmol/L.

Results:

Of 61 patients (age: 1–95 years, burns: 20–85% BSA), 20 (32.7%) developed hypercalcemia. There was a significant correlation between calcium levels and immobilization (*p* < 0.05), and a positive correlation between longer stays and mortality. Hypercalcemia was more common in patients with acute renal failure (14% vs. 6%) and was associated with higher mortality. Initial electrolyte imbalances included hyponatremia (37%), hyperkalemia (16.3%), and hyperchloremia (45%).

Conclusions:

Hypercalcemia and acute renal failure occur earlier and more frequently than previously reported in burn patients. Bisphosphonates are an effective treatment for severe hypercalcemia and can help prevent bone loss.

P110

Robot portrait of tunisian electrical burn patient: Analysis of 322 cases

Fredj, H. ^1^, Chaabani, M. ^1^, Aloui, A. ^1^, Alouini, A. ^1^, Jami, I. ^1^, Gasri, B. ^1^, Messadi, A. ^1^, **Mokline, A.** ^1^

^1^ Burn Intensive Care Unit, Traumatology and Burn Center, Ben Arous, Tunisia

Poster presentations 2, 4 September 2025, 10:00–10:30

Introduction: Electrical burns were associated with high morbi-mortality.

The aim of our study was to determine the profile of the patient victim of electrical burns in Tunisia through an epidemiological and clinical study.

Methods:

A descriptive, retrospective study was conducted in the intensive burn care unit in Tunisia over an 8-year period (January 2017–December 2024). Patients admitted for electrical burns were included. Epidemiological, clinical, and outcome data were collected and analyzed.

Results:

During the study period, 3460 patients were admitted, 322 were included (incidence of 9.4%). The average age of patients was 34 ± 12 years, with a male predominance (97.1%). Most cases involved high-voltage electrical accident (85%). Work related accidents represented the most common circumstance (62%), affecting mainly construction workers (45%) and painter (20%). TBSA was 17.3 ± 16.3%. Burns were deep in 65% of cases, affecting both upper limbs (42%) and lower limbs (42%). Escharotmy was done in 21% of cases and amputation was performed in 23.3% of cases. Mechanical ventilation was required in 18% of cases. Biological abnormalities included rhabdomyolysis (78%), elevated troponins (57.5%), hemoconcentration (43.5%), metabolic acidosis (23.3%), and acute renal failure (15.5%) with renal replacement therapy requirement in 6% in cases. Length of ICU stay was 9.2 (6–15) days, and mortality was 17.3%.

Conclusions:

Tunisian electrical burn victim is a 34-year-old male, construction worker, in 45% of cases, burned by high-voltage due to work related accident. TBSA was about 17% affecting extremities, leading to amputation of at least one limb (23.3%).

P111

Correlation of O2C-Device with Cutometer and scar scales in evaluation of burn scars: a retrospective cohort study of scar elasticity and perfusion.

**Galati, V.** ^1^, Kisch, T. ^2^, Harhaus-Wähner, L. ^3^, Vonthein, R. ^4^, Hoenning, A. ^5^

^1^ Centre for Severe Burn Injuries with Plastic Surgery, BG Klinikum Unfallkrankenhaus Berlin, email: galativirginia@gmail.com, Berlin, Germany, ^2^ Praxisklinik Kronshagen, Kronshagen, Germany, ^3^ Centre for Hand, Replantation and Microsurgery BG Klinikum Unfallkrankenhaus Berlin, Charité Universitätsmedizin, Berlin, Germany, ^4^ Institute of Medical Biometry and Statistics, University of Luebeck, Luebeck, Germany, ^5^ Centre for Clinical Research, BG Klinikum Unfallkrankenhaus Berlin, Berlin, Germany.

Poster presentations 2, 4 September 2025, 10:00–10:30

Aim:

The O2C device (LEA Medizintechnik GmbH, Giessen, Germany) provides non-invasive monitoring of microcirculatory parameters in tissues and organs. Blood flow, one of the four parameters measured, has been used to assess burn scars, with higher values observed in hypertrophic scars. However, limited research exists on the O2C device’s role in evaluating burn scars. This secondary analysis aims to explore the correlation between O2C measurements, Cutometer data, and scar scale scores (VSS, POSAS) in burn scar evaluation.

Methods:

A secondary analysis was conducted on the patient cohort from our previous retrospective clinical study. This study assessed the elasticity and perfusion of burn scars after autologous split-thickness skin grafting or the application of the temporary dressing Suprathel, at least one year after treatment. Pearson’s correlation coefficient, canonical correlations, and stepwise regression analysis were used to assess relationships between O2C measurements Cutometer data and scar scale scores.

Results:

Statistical analysis revealed a lack of correlation between between O2C measurements, Cutometer data and scar scale scores. Among microcirculatory parameters, only weak correlations were found, with the exception of blood flow and velocity.

Conclusions:

Our analysis showed no correlation between O2C measurements, Cutometer data, and scar scale scores assessments. The O2C device provides objective, reproducible measurements of microcirculation and perfusion in burn scars, offering valuable data for evaluating scar healing and functionality. While it may serve as an additional tool in burn scar assessment, further research is needed to determine its clinical relevance.

P112

A 5-year retrospective review of the management and outcomes of facial burns at a major burns centre in London

Gashi, V. ^2^, Chendriah, J. ^1^, Elmitwalli, D. ^3^, Essam, M. ^1^, Li, H. ^1^, Mann, A. ^1^, Seyone, D. ^1^, Akyol, E. ^1^, Markeson, D. ^1^

^1^ Chelsea & Westminster Hospital, London, United Kingdom, ^2^ University Clinical Center of Kosovo, Pristina, Kosovo, ^3^ Theodor Bilharz Research Institute, Giza, Egypt.

Poster presentations 2, 4 September 2025, 10:00–10:30

Aim:

The aim of this study was to evaluate the ophthalmic and oculoplastic implications of facial burns, focusing on the management and clinical outcomes.

Methods:

We used the International Burn Injury database (IBID) to retrieve electronic records of facial burn patients presenting to Chelsea and Westminster Hospital between 2019 and 2024. 360 patients met the inclusion criteria and we recorded patient demographics, burn characteristics, timing of ophthalmology review, intervention(s) and complication(s). Clinical data was retrospectively collected from institutional electronic medical records.

Results:

227 patients (63.05%) were male, 132 (36.67%) female and 1 (0.28%) unspecified gender with a mean age of 30 (range 0–92). Thermal burns were most common (89.72%), followed by chemical (6.95%) and electrical burns (3.33%). 314 (87.22%) of burns were accidental, 27 (7.50%) were assault, 16 (4.45%) self-inflicted and 3 (0.83%) were unknown. Periocular burns were observed in 38.33% of cases, with bilateral involvement in 64.49%. A total of 78.7% patients were reviewed within 48–72 h, and 7.22% underwent early inpatient surgical management. Ophthalmic sequelae were documented in 6.11% of cases (59% eyelid-related, 22.72% corneal, 9.09% affecting vision, 4.54% surgery-related complications).

Conclusions:

This large retrospective analysis highlights the risk of ocular complications in periocular burns, mainly affecting the eyelid, cornea and vision. Most patients were conscious, benefiting from blink reflex protection. Early ophthalmology review reduced severe complications and need for surgery, whilst chemical burns, though less common, required specialised intervention. This research provides a risk stratification algorithm to guide ophthalmology involvement and assess periocular prognosis.

P113

An innovative simulation-based course for severe burn management in undergraduate medical education.

**Górecka, Z.** ^1,2^, Surowiecka, A. ^1,2^, Majewski, M. ^1^, Wilhelm, G. ^1^, Barbachowska, A. ^2^, Antonov, S. ^1^, Torres, K. ^1,4^, Strużyna, J. ^1,3^, Korzeniowski, T. ^1,3^

^1^ Department of Plastic and Reconstructive Surgery and Microsurgery, Medical University of Lublin, Lublin, Poland, ^2^ East Centre of Burns Treatment and Reconstructive Surgery, Medical University of Lublin, Łęczna, Poland, ^3^ Department of Plastic and Reconstructive Surgery and Burns Unit, Medical University of Lublin, Lublin, Poland, ^4^ Chair and Department of Didactics and Medical Simulation, Medical University of Lublin, Lublin, Poland.

Poster presentations 2, 4 September 2025, 10:00–10:30

Aim:

We aim to share our experience with an innovative approach to burns education using simulation scenarios at the Medical University of Lublin, Poland.

Methods:

An evaluation was conducted on the burns teaching methodology at the Medical University of Lublin.

Results:

Each year, 360 sixth-year Polish medical students, along with 50 students from the international program, take part in a burn treatment course. Teaching is carried out at Centre of Medical Simulation through the high-fidelity simulation session and consists of prebriefing, a simulation scenario, and debriefing. This simulation session is designed to teach the management of a patient admitted to the Emergency Department with a thermal burn. The primary educational goal is to develop the necessary skills for handling such cases in safe environment. The scenario focuses on four specific medical objectives: stabilizing a trauma patient with extensive burns across multiple body regions, conducting a detailed medical interview along with a comprehensive general and trauma-focused physical examination, accurately calculating the burn area and evaluating the depth of burn injuries and implementing appropriate diagnostic and treatment procedures.

Conclusions:

The burn scenario implemented by the Medical University of Lublin effectively prepares future doctors for decision-making and management of burn cases. Incorporating similar training into medical curricula at other universities could significantly improve and expand undergraduate education in burn care.

P115

Compared Results of Conventional Dressing and Spincare Treatment for Burns

**Khuvkhuu, T.** ^1^

^1^ National Traumatology and Orthopedics Research Center, Ulaanbaatar, Mongolia

Poster presentations 2, 4 September 2025, 10:00–10:30

Compared Results of Conventional Dressing and Spincare Treatment for Burns

Tuya.Kh1, Nyamdavaa.V 2, Monohtsatsral.G3

Burn Center of Mongolia

tuya.hk@gmail.com

Keywords: II-IIIA degree burns, painless dressing, rapid healing

Introduction:

The Spincare system is an advanced wound dressing that supports primary healing, maintains wound freshness, and prevents infection. It creates a nanofibrous epidermis with a single spray, adhering directly to the wound, allowing breathability, water permeability, and movement flexibility. The dressing remains on the wound until full healing, then peels off naturally.

Objective:

To compare the efficacy of traditional wound dressings with the Spincare treatment system.

Methods:

This study included 168 patients with II-IIIA degree burns admitted to the National Trauma and Orthopedic Center (October 2022–December 2024). Participants were randomized into two groups: Spincare and conventional dressing. The mean dressing time per patient was significantly longer for Spincare (28.5 min) compared to conventional methods (10.2 min) (*p* < 0.05).

Results:

Wound healing rates were 95.8% for Spincare and 94.5% for traditional dressings (*p* = 0.85), showing no significant difference. However, pain relief and patient satisfaction were significantly higher with Spincare (*p* < 0.01). Spincare required fewer dressing changes, reducing material use and staff workload, while minimizing patient discomfort, especially in children.

Conclusions:

Both methods demonstrated similar healing outcomes, but Spincare offered superior pain relief, higher patient satisfaction, and reduced dressing frequency, making it a beneficial alternative for burn management.

1. Burn surgeon of Burn department; tuya.hk@gmail.com; +976 95091591
2. Burn surgeon of Burn department; +976 89057704
3. Pediatrician of ICU; +976 99817309

P116

Influence of magnetic field arrangements on the growth of cultured epidermal autografts derived from a modified South African-based technique: A pilot study.

**Kleintjes, W.** ^1,2^, Prinsloo, T. ^3,4^

^1^ Department of Surgical Sciences, Stellenbosch University, Cape Town, South Africa, ^2^ Western Cape Provincial Adult Tertiary Burns Centre, Tygerberg Hospital, Cape Town, South Africa, ^3^ Department of Biomedical Sciences, Cape Peninsula University of Technology, Cape Town, South Africa, ^4^ Department of Emergency Medical Sciences, Cape Peninsula University of Technology, Cape Town, South Africa.

Poster presentations 2, 4 September 2025, 10:00–10:30

Aim:

Cellular electric fields (EFs) influence molecular and biological mechanisms that regulate behaviours such as cellular migration, proliferation, and growth; and varying magnetic fields (MFs) may influence these processes in cultured epidermal autografts (CEA). This study aimed to report on CEA growth patterns using a modified culture technique and different MF arrangements.

Methods:

Epidermal fragments were isolated from biopsies and the keratinocytes retrieved with trypsin were then seeded onto Cutimed^®^ (Stockholm, Sweden) Sorbact dressings with autologous platelet-rich plasma (PRP). These were placed directly on varying magnet arrangements (MF strength of magnets were relatively low) and incubated in pediatric incubators at 37 °C for 1 week to observe growth progression. CEA was supplemented daily with fresh PRP, and Intrasite Gel^®^ every 3–4 days. Light microscopy (100×) was used to visually assess cell growth in terms of viscosity, density, and potential pigmentation as primary outcomes.

Results:

The overall CEA cell growth was more viscous and dense with darker pigmentation on the Sorbact sheets that had magnetic arrangements with continuous MFs compared to those arrangements with gaps.

Conclusions:

CEA cultivated under continuous MFs exhibited favourable cell growth and further suggests that even low MF strength can influence cellular EFs and proliferation. This pilot evidence supports the potential for enhanced growth with MF stimulation, warranting further investigation for definitive conclusions.

P117

Investigating Pyrexia in Paediatric Patients with <10%TBSA Burns in Alder Hey

**Lam, D.** ^1^, Ryles, N. ^2^, Falder, S. ^1,2^

^1^ University of Liverpool, Liverpool, United Kingdom, ^2^ Alder Hey Children’s Hospital, Liverpool, United Kingdom.

Poster presentations 2, 4 September 2025, 10:00–10:30

Aim:

Our study aims to investigate pyrexia <38 °C in paediatric burns, <10% TBSA, a poorly understood but frequent clinical sign.

Methods:

This is a prospective study, commenced in November 2024. All patients <14 years admitted for >24 h with burns <10% TBSA were included. Demographic and clinical information was collected from the electronic record with additional information from ward staff. Statistical analysis was carried out using SPSS.

Results:

To date, 47 patients (16 female, 31 male) are included; median age 21 months (range 1–159 months); median TBSA 2.25% (SD 1.95).

18 patients (38%) experienced at least one pyrexia, of whom 11 (61%) experienced multiple episodes.

Time from injury to first pyrexia: median 22.17 h, mean 26.85 h (SD 17.95).

Time from first cleaning to first pyrexia: mean 17.15 h, median 11.32 h (SD17.04).

Time from any cleaning to pyrexia: mean 10.26 h, median 6.61 h (SD11.7).

17 patients (36%) had concurrent viral infection. Of 18 patients with pyrexia, 15 (83%) had a viral infection.

5 patients received antibiotics for spiking temperatures, of which 3 (60%) had concurrent viral infection. Only 2 patients had features of burn wound infection.

Conclusions:

Children who developed a pyrexia did so with a mean of 26 h, which aligns with a previous study. However, most of our children (83%) had a concurrent viral infection which could have caused the pyrexia. There was a trend towards pyrexia developing within 12 h of cleaning. This may represent cleaning contributing towards an inflammatory response.

P118

Impact of Total Body Surface Area (TBSA) on Effective Surgical Treatment and Mortality in Adult Burn Patients: A 5-Year Retrospective Analysis in an Indonesian National Referral Hospital

Leksono, T. ^1^, Wardhana, A. ^1^, Sanjaya, I. ^1^, Handiansyah, B. ^1^, **Farhana, N.** ^1^

^1^ Division of Plastic Reconstructive and Aesthetic Surgery, Department of Surgery, Faculty of Medicine, Universitas Indonesia, Cipto Mangunkusumo Hospital, Jakarta, Indonesia, Kota Jakarta Pusat, Daerah Khusus Ibukota Jakarta, Indonesia.

Poster presentations 2, 4 September 2025, 10:00–10:30

Aims: This study evaluates the impact of total body surface area (TBSA) on surgical treatment outcomes and mortality rates in burn patients.

Methods:

A retrospective cohort study was conducted on adult burn patients admitted to an Indonesian National Referral Hospital from January 2020 to December 2024. Patients were classified into TBSA categories and surgery count. Factors associated with mortality, including age, TBSA and surgery count were analyzed using SPSS version 26.

Results:

This study included 595 patients, with a mean age of 41.77 ± 14.94 years and a male predominance (*n* = 406, 68.2%). Flame or explosion-related burns were the most common cause of hospitalization (*n* = 473, 79.35%). Mortality was significantly linked to age, TBSA, surgery count, and hospital stay (*p* < 0.05). While increasing TBSA correlated with more surgeries, patients with TBSA >40% underwent fewer procedures. Higher TBSA also markedly increased mortality risk, with TBSA >40% showing the highest odds (OR 12.253, 95% CI: 4.1–36.5, *p* < 0.05). General escalation in TBSA was found to significantly heighten the risk of mortality (OR 2.57, 95% CI: 2.1–3.0, *p* < 0.05). The predominant cause of mortality identified in this study was sepsis (*n* = 161, 33.82%).

Conclusions:

A greater TBSA is closely associated with higher mortality and the need for more surgical interventions. However, patients with extensive burns (TBSA > 40%) underwent fewer procedures, possibly due to the limitations of surgical management.

P120

Statistical analysis of burn victims aiming to improve clinical decision-making, optimize triage protocols and produce targeted prevention strategies

**Tremma, P.** ^1^, Lyssiotis, F. ^2^

^1^ School of Mechanical Engineering, Aristotle University, Thessaloniki, Greece, ^2^ KAT Hospital, Clinic for Plastic, Reconstructive Surgery and Burn Unit, Athens, Greece.

Poster presentations 2, 4 September 2025, 10:00–10:30

Aim:

This study presents an analysis of factors associated with burn injuries in Greece, aiming to identify clinically significant patterns and predictive factors related to patient outcomes such as the hospitalization time of patients, as well as in the development of predictive models for patient outcomes upon hospital admission.

Methods:

Data were retrospectively collected the past five years from the General Hospital KAT in Athens, Greece. KAT Hospital, being a major Trauma Center of Greece, receives burn victims from all Greek territory, thus presents a representative population of burn victims for data collection in our country. Data include variables such as age, gender, percentage of total burned body surface area (TBSA), presence of suspected inhalation injury, mechanism of trauma, number of surgeries performed, and final outcome (length of stay or death).

Various statistical analysis techniques were used to describe the data and to develop predictive models.

Results:

Predictive models created can be applied to estimate clinical outcome early in the treatment process.

Preliminary analyses highlight strong associations between TBSA, age, and the presence of inhalation injury with both prolonged hospitalization and fatality. Additional analysis explored the distribution of trauma mechanisms across demographics, the influence of surgical intervention, and the relationship between the number of surgeries and survival outcomes.

Conclusions:

These findings contribute to improved clinical decision-making, optimized triage protocols, and targeted prevention strategies. Future research will focus on external validation and the integration of comorbidity data, preferably within a larger, multi-center dataset to enhance generalizability.

P121

SPECTRABURN: Towards a Comprehensive Hyperspectral Burn Wound Analysis Registry

**Martin, R.** ^1^, Roos, J. ^2^, Besch, J. ^1^, Hecker, A. ^1^, Lambert, S. ^1^, Weber, S. ^1^, Siemers, F. ^1^, Kaczmarczyk, R. ^3^

^1^ BG Klinikum Bergmannstrost Halle, Clinic for Plastic and Hand Surgery, Burn Care Center, Halle (Saale), Germany, ^2^ University Hospital of Bonn, Department of Orthopedics and Trauma Surgery, Bonn, Germany, ^3^ Technical University of Munich, Department of Dermatology and Allergy.

Poster presentations 2, 4 September 2025, 10:00–10:30

Aim:

Objective burn depth assessment remains a key challenge in burn care, typically relying on subjective clinical judgment. Hyperspectral imaging (HSI) enables detailed analysis of tissue structures via spectral signatures and offers strong potential for automated diagnostic tools. The “SPECTRABURN” initiative aims to establish a comprehensive registry of hyperspectral burn wound images to support standardized diagnostics. Advanced dimensionality reduction techniques such as UMAP and PaCMAP are applied to enhance data analysis and enable objective burn depth classification.

Methods:

Since February 2024, HSI data from burn patients have been systematically collected at the Burn Center of BG Klinikum Bergmannstrost during emergency care. The TIVITA^®^ Tissue System (Diaspective Vision, 640 × 480 px, 500–1000 nm) (Protex Healthcare, Roeselare, Belgium) is used for imaging. Patient- and wound-specific metrics are recorded continuously. Data processing is performed using Python (Pandas, Matplotlib).

Results:

As of the end of March 2025, data from 75 patients (59 male, 16 female; mean age: 51.2 ± 20.1 years) had been collected, yielding 153 HSI recordings across all burn depths. The most common locations were forearm and hand. Initial results demonstrate that dimensionality reduction effectively preserves key data structures and enhances visualization. The applied algorithms show strong potential for developing automated classification tools.

Conclusions:

A hyperspectral imaging registry offers a valuable basis for developing and validating automated burn depth assessment methods. Dimensionality reduction represents a critical first step toward achieving this goal.

Keywords: hyperspectral imaging, burn depth assessment, dimensionality reduction

P122

A standardized catalog of burn depths based on temperature and duration in a pig model.

**Moshammer, M.** ^1,2^, Prevedel, M. ^1,2^, Villa-Garcia, A. ^1,2^, Kamolz, L.^1,2^, Kotzbeck, P. ^1,2^, Hofmann, E. ^1,2^, Foessl, I. ^1,3^

^1^ COREMED—Center for Regenerative and Precision Medicine, JOANNEUM RESEARCH Forschungsgesellschaft mbH, Graz, Austria, ^2^ Division of Plastic, Aesthetic and Reconstructive Surgery, Department of Surgery, Medical University of Graz, Graz, Austria, ^3^ Division of Endocrinology and Diabetology, Department of Internal Medicine, Medical University of Graz, Graz, Austria.

Poster presentations 2, 4 September 2025, 10:00–10:30

Aim:

We present the first standardized catalog of burn depths resulting from various combinations of burn temperatures and durations in pigs. This catalog is intended as a reference for researchers in the field, providing guidance for their experiments and ultimately helping to save resources and reduce the use of animals by minimizing pre-testing.

Methods:

In four Landrace pigs weighing approximately 25 kg, burn wounds were inflicted using standardized stainless steel blocks (3 × 3 cm, 836 g) immediately after narcotic sacrification, with burn temperatures ranging from 70 °C to 80 °C and durations from 2 to 30 s. Histological assessment of burn wound depth was performed by experienced observers and measured using Aperio ImageScope. Evaluated factors included flattening of the rete ridges, epidermal detachment, nuclear damage, loosening of the dermal structure, collagen condensation, and damage to subcutaneous adipose tissue.

Results:

Burn depths resulting from various burn temperatures and durations were compiled into a table. Representative examples are burns at 80 °C for 10 s, which resulted in an average burn depth of 1.899 mm, indicating a deep dermal burn, whereas burns at 70 °C for 25 s led to an average burn depth of 0.903 mm, corresponding to a more superficial partial-thickness burn.

Conclusions:

The findings suggest that higher temperatures result in deeper tissue damage more rapidly, whereas prolonged exposure at lower temperatures leads to less severe burn penetration. This systematic burn depth analysis serves as a reference for researchers in the field, aiding in the optimization of burn conditions for experimental studies.

P124

Patient experience of using artificial intelligence-enhanced multispectral imaging to measure burn depth: a regional burns centre review.

**Nyeko-Lacek, M.** ^1^, Walsh, K. ^1^, Sheikh, Z. ^1^

^1^ Manchester University NHS Foundation Trust, Manchester, United Kingdom

Poster presentations 2, 4 September 2025, 10:00–10:30

Aim:

To evaluate patient perceptions of the accuracy, comfort, and overall acceptance of using an AI-powered multispectral imaging device to assess burn depth during a burns consultation, and to determine its acceptability as part of the clinical consultation process.

Method:

Adult patients with full mental capacity presenting to a regional burns centre following burn injuries between 30/08/24 and 24/01/25 underwent both a clinical assessment and an AI-based imaging assessment. After reviewing their images on the monitor, patients completed a 4-item questionnaire to rate their experience of the device on a 10-point Likert scale. Feedback was gathered on speed, comfort, and overall acceptability.

Results:

Fifty-five patients completed the questionnaire. The median score for device speed was 10, with an interquartile range (IQR) of 1. In terms of comfort, 85% of patients rated the device as 10, with a median of 10 and an IQR of 0 (range 8–10). For satisfaction with further imaging, 80% rated it 10 (range 7–10). Regarding how much the device helped them understand their burn depth, 80% rated it 8 or higher, yielding a median score of 10 with an IQR of 2.

Conclusions:

Patients were highly receptive to the technology, reporting that it improved their understanding of burn depth. They found the device comfortable and efficient, indicating its potential for use in paediatric settings. This study supports the acceptability of AI-powered multispectral imaging in clinical consultations for burn assessments.

P125

Accuracy of burn size estimation in patients referred to a UK regional burns centre

**Nyeko-Lacek, M.** ^1^, Bodapati, V. ^1^, Sheikh, Z. ^1^

^1^ Manchester University NHS Foundation Trust, Manchester, United Kingdom

Poster presentations 2, 4 September 2025, 10:00–10:30

Aim:

This study aimed to evaluate potential discrepancies between the total body surface area (TBSA) calculations made by the referring institutions and those assessed at a UK regional burns centre.

Methods:

Data from all referrals to a UK regional burns centre over a one-year period were analysed using the International Burn Injury Database (IBID).

Results:

A total of 705 patients were referred to the centre during the study period (31 January 2024–31 january 2025). In 40.6% of cases, the TBSA calculated by the referral site and the burns centre were the same. In 10.1% of cases, the TBSA was underestimated, while in 49.4% it was overestimated. A discrepancy of ≥5% was found in 11.8% of referrals. Among the 36 referrals categorized as ≥20% TBSA (adult resuscitation burns according to the latest EMSB guidelines), 22 were overestimated by >10%, and 3 were underestimated by >10%.

Conclusions:

Significant discrepancies in TBSA estimation occurred during the study period, particularly among patients referred as resuscitation burns to the regional burns centre. Overestimations were more common than underestimations. These discrepancies have important implications for the initial management of burns, including fluid resuscitation calculations and the decision to transfer patients to a specialised burns centre.

P126

Cd42-expressing extracellular vesicles in burn patients complicated by sepsis as potential diagnostic and prognostic biomarkers.

Schiavello, M. ^1^, **Pensa, A.** ^2^, Vizio, B. ^1^, Bruno, S. ^1^, Bosco, O. ^1^, Dini, C. ^1^, Sciarrillo, A. ^2^, Risso, D. ^2^, Navissano, M. ^2^, Camussi, G. ^1^, Montrucchio, G. ^1^, Mariano, F. ^1^, Lupia, E. ^1^

^1^ Department of Medical Sciences, University of Turin, Turin, Italy, Torino, Italy, ^2^ Burn Centre, CTO Hospital, A.O.U. Città della Salute e della Scienza, Turin, Italy.

Poster presentations 2, 4 September 2025, 10:00–10:30

Introduction. Sepsis is the leading cause of death in patients with burn injury. Extracellular vesicles (EVs) have emerged as novel cell-to-cell mediators and promising non-invasive biomarkers that may aid in the diagnosis and prognostic stratification of patients with sepsis. Here, we studied EVs released during sepsis in burn patients by multiplexed phenotyping.

Methods:

A prospective observational study was conducted at the Burn Centre of CTO Hospital, Turin, Italy. We enrolled 33 patients with burn injuries: 23 patients developed sepsis (Burn Septic Patients—BSP), whereas 10 burn patients showed no evidence of infection (Burn Non-Septic Patients—BnSP). Sepsis was diagnosed according to Sepsis-3 guidelines plus positivity of blood cultures. Ten healthy subjects were used as an additional control group. EVs were isolated from plasma by ultracentrifugation or precipitation-based methods, and characterized by Transmission Electron Microscopy and flow cytometry. EV surface antigens were studied by bead-based multiplex flow cytometry.

Results:

EVs from BSP expressed a specific pattern of epitopes distinct from those from BnSP and HS. Specifically, EVs from BSP showed an increase in CD42a expression compared to BnSP- derived EVs. ROC analysis of CD42a-EVs showed an AUC of 0.947 (95% CI, 0.855–1.000; *p* = 0.003) for the diagnosis of sepsis in burn patients. Of interest, CD42a-EVs expression was significantly increased in BSP with the highest SOFA score (≥ 11) compared to those with SOFA score 4–6 (*p*= 0.008) or 7–10 (*p* = 0.014).

Conclusions:

Our results suggest the potential of CD42a-expressing EVs as diagnostic and prognostic biomarkers of sepsis in burn patients.

P127

The use of alloplastic skin in burn injuries from 1999 to 2024: experience from the burn center in Turin

**Pensa, A.** ^1^, Risso, D. ^1^, Cambieri, I. ^2^, Alotto, D. ^2^, Fumagalli, M. ^2^, Castagnoli, C. ^2^, Navissano, M. ^1^

^1^ Burn Center, AOU Città della Salute e della Scienza, Torino, Italy, ^2^ Bank Tissue; AOU Città della Salute e della Scienza, Turin, Italy.

Poster presentations 2, 4 September 2025, 10:00–10:30

Aim:

This paper describes the applications of alloplastic skin in our Burn Center in synergy with our Tissue Bank.

Methods:

At Skin Bank of Turin between 1999 to 2024, a total of 2,511,685 cm^2^ of skin was harvested from 1285 donors (median 1954.70 cm^2^ for donor). The skin was processed using standardized disinfection techniques and preserved in glycerol or cryopreserved.

The unsuitable skin flaps were discarded in the presence of positive microbiological or donor exclusion criteria.

Results:

Over 25 years, 569 patients with burns (21 patients/year) were treated for a total of 1,679,614 cm^2^ of alloplastic skin used in 849 surgical procedures. The main use was temporary covering in severe burns (602 surgeries), followed by permanent covering through the techniques of Alexander (223 surgeries) and Cuono (24 surgeries). Mortality in severely burned patients from the moment that the Skin Bank began to distribute alloplastic skin becoming the “gold standard” of treatment, has decreased from 23.4% to 14%, confirming the usefulness of this therapeutic remedy as a life-saving.

Conclusions:

The introduction and continued use of alloplastic skin at Burn Centre in Turin has represented a significant advance in the management of medium and large-scale burns. The immediate availability of this resource has allowed timely interventions and life saving, optimizing surgical treatment strategies.

P128

Analysis of epidemiological, clinical and microbiology characteristic and surgical treatment of burn patient—single tertial center experiences.

**Petrovic Popovic, D.** ^1^, Petrović Elbaz, M. ^1^, Novaković, M. ^1^

^1^ Clinic for burns, plastic and reconstructive surgery, University Clinical Center of Serbia, Beograd, Serbia, ^2^ Barens Jewish Hospital, Washington University, USA, St Louis, USA.

Poster presentations 2, 4 September 2025, 10:00–10:30

Aim:

The aim of this study is to analyze the summarized epidemiological and clinical data of severe burn patients and surgical management during the five year period in order to acquire an accurate and recent picture of this pathology.

Methods:

The study retrospectively analyzes the data of burn patients admitted in the Clinic for Burns, Plastic and Reconstructive Surgery, University Clinical Center of Serbia, Belgrade, Serbia, Europe during the five year period. The following data were collected: age, sex, comorbidities, hospital of primary treatment, mechanism of injury (flames, scalds, electrical injury), total body surface area (TBSA) burned, presence of inhalation injury, comorbidities, infection, season of burn injury and outcome.

Results:

Total number of patients were 790 during the five year period, male patients were 558 (70,6%)-and female 232 (29,4%) with average age 52.52 ± 19.26, age range was 16–97 years. The most frequent causes of burns were flame (45%), followed by scalds (36%), contact (10%), electrical (5%), chemical (2%) and unknown cause (2%). Average number of days in ICU was 19 days ± 17.2 days and 27.8% of burned patients had one or more infections, average TBSA of patients was 16.36 ± 10%. Comorbidities were present 36.9% CVD, 14.2% endocrinology, 10.5 psychiatry leading etilismus, 10.5% GIT and 27.9% more than two comorbidities.

Conclusions:

Epidemiology and etiology has changed through decades, and in this study showed decrease in the number of patients with severe burns injuries every year.

P129

Dynamics of Tissue Regeneration After Deep Dermal Burns

**Prevedel, M.** ^1,2^, Pignet, A. ^1,2^, Fink, J. ^1,2^, Funk, M. ^3^, Bernardelli de Mattos, I. ^3,4^, Madl, T. ^5^, Habisch, H. ^5^, Einsiedler, J. ^1,2^, Hahn, D. ^1^, Eberl, A. ^6^, Hecker, A. ^1,2^, Birngruber, T. ^6^, Kamolz, L. ^1,2^, Kotzbeck, P. ^1,2^, Hofmann, E. ^1,2^

^1^ COREMED—Center for Regenerative and Precision Medicine, JOANNEUM RESEARCH Forschungsgesellschaft mbH, Graz, Austria, ^2^ Division of Plastic, Aesthetic and Reconstructive Surgery, Department of Surgery, Medical University of Graz, Graz, Austria, ^3^ EVOMEDIS GmbH, Graz, Austria, ^4^ Department of Tissue Engineering & Regenerative Medicine, University Hospital Würzburg, Würzburg, Germany, ^5^ Gottfried Schatz Research Center for Cell Signaling, Metabolism and Aging, Molecular Biology and Biochemistry, Medical University of Graz, Graz, Austria, ^6^ HEALTH-Institute for Biomedicine and Health Sciences, JOANNEUM RESEARCH Forschungsgesellschaft mbH, Graz, Austria.

Poster presentations 2, 4 September 2025, 10:00–10:30

Aim:

Thermal injuries initiate a cellular cascade that extend beyond the site of injury, often resulting in significant tissue damage and systemic effects. Burn wounds are not static, as they undergo a complex progression in the first few days after injury, leading to deeper tissue involvement. This study investigates the dynamic progression of burn wounds, focusing on the histological features, inflammatory markers, and metabolic responses over time.

Methods:

Contact burns (80 °C for 20 s) were induced on pigs (*n* = 4) at four different occasions. Biopsies and dermal interstitial fluid were obtained from wounds in different healing stages, i.e., 8 h, 4, 11, and 21 days post burn, with unburned areas as controls. Histology, immunohistochemistry and gene expression analyses were performed on tissue biopsies. Metabolomics of tissue biopsies and the dermal interstitial fluid were assessed via NMR spectroscopy.

Results:

Histological analysis revealed deep dermal burns at 8 h post-injury, progressing to full-thickness burns. Re-epithelialization began at 4 days, with complete epithelialization at 21 days. Inflammation peaked at 8 h. Pro-inflammatory markers (IL-6, PGE2) and angiogenesis markers (VEGFA) showed a regressive dynamic. Metabolomics analysis revealed that myo-inositol, ketoleucine, and phosphorylcholine peaked at 8 h but returned to baseline by day 4. Mannose decreased at 8 h and 4 days, returning to baseline at 21 days.

Conclusions:

Our detailed analyses of burn wound dynamics in the present porcine in vivo study provide profound knowledge on the temporal dysregulation of key mediators such as cytokines and metabolites in the progress of burn wound healing.

P130

Identification and functional profiling of potential prognostic miRNA biomarkers for the presence and severity of inhalation injury in burn patients.

**Prinsloo, T.** ^1^, Kleintjes, W. ^2,3^, Najaar, K. ^4,5^

^1^ Department of Biomedical Sciences, Cape Peninsula University of Technology, Cape Town, South Africa, ^2^ Department of Surgical Sciences, Stellenbosch University (Medical School), Cape Town, South Africa, ^3^ Western Cape Provincial Adult Tertiary Burns Centre, Tygerberg Hospital, Cape Town, South Africa, ^4^ Department of Emergency Medical Sciences, Cape Peninsula University of Technology, Cape Town, South Africa, ^5^ Department of Wellness Sciences, Cape Peninsula University of Technology, Cape Town, South Africa.

Poster presentations 2, 4 September 2025, 10:00–10:30

Aim:

This study aimed to identify potential differentially expressed micro(mi)RNA profiles and their functionally enriched pathways for inhalation injury presence and/or severity prognosis.

Methods:

Blood samples (*n* = 59) were collected from burn patients shortly after admission to the Western Cape Provincial Adult Tertiary Burns Centre, Tygerberg Hospital, Cape Town, South Africa, over an 18-month period. Total RNA was extracted, with quality (Bioanalyzer, Agilent, Santa Clara, CA, USA) and quantity (Nanodrop, Waltham, MA, USA) assessed. The miRNA profiling of exemplar samples (mild and severe inhalation injury cases) was performed using high-throughput sequencing (Illumina NextSeq 550, San Diego, CA, USA). Differential expression (DE) analysis was conducted with EdgeR in R and corroborated by DESeq2, with the Fisher’s Exact test comparing DE miRNAs between groups. Overlapping miRNAs meeting *p*-adjusted <0.05 and fold change >1.5 values were subjected to target gene (miRNet), protein-protein interaction (STRING), and hub gene (cytoHubba) analyses. The top 10 functionally enriched pathways (*p* < 0.05) of the top 10 hub genes were the determined with EnrichR-integrated platforms (GO, KEGG, Reactome, PANTHER).

Results:

Ten DE miRNAs overlapped and met threshold cut-off values, including nine upregulated (miR-143-3p, -200b-3p, -148b-5p, -10b-5p, -30a-5p, -15a-5p, -374a-5p, -21-5p, -144-5p) in severe injury and one downregulated (miR-504-5p) in mild injury samples. Target gene and subsequent protein-protein interaction network analysis identified key hub genes that were predominantly enriched in inflammatory and apoptotic signalling pathways.

Conclusions:

These findings suggest a regulatory role for the DE miRNAs and their target genes in pathways central to inhalation injury. The preliminary results provide a strong foundation for their prognostic potential for inhalation injury presence and/or degree.

P131

Incidence of burns and their anatomical distribution—a nationwide register study in Finland 2011–2015

**Purola, L.** ^1^, Vuola, J. ^1,2^, Kavola, H. ^1^, Palmu, R. ^3^, Hulkkonen, S. ^4^

^1^ Department of Plastic Surgery, Helsinki University Hospital, Helsinki, Finland, ^2^ Department of Plastic Surgery, Helsinki Burn Centre, Helsinki University Hospital, and University of Helsinki, Helsinki, Finland, ^3^ Department of Psychiatry, Helsinki University Hospital, and University of Helsinki, Helsinki, Finland, ^4^ Department of Hand Surgery, Helsinki University Hospital and University of Helsinki, Helsinki, Finland.

Poster presentations 2, 4 September 2025, 10:00–10:30

Aim:

To provide a register-based analysis of the incidence, anatomical distribution, and associated operative care of all burn injuries treated by specialists.

Methods:

The study covers the population of Finland from 2011–2015. Data includes all burn patients registered in the Care Register for Health Care. We utilized the International Classification of Diagnoses, the tenth revision code for burn injuries, and the Nordic Medico-Statistical Committee Classifications of Surgical Procedures for procedure codes. We calculated the mean crude and adjusted (to European Standard Population 2013) incidence rates of burns as cases per 100.000 person-years, assuming Poisson distribution of cases for calculating 95% confidence intervals.

Results:

The sample included 16,575 patients (63% men). The mean standardized annual incidence rate per 100.000 person-years of any burn injury was 60.8 (95% CI, 59.9–61.7). The corresponding rates were 76.3 (95% CI, 74.9–77.8) for men and 45.2 (95% CI, 44.1–46.3) for women. Among patients admitted for two or more days, the incidence rate per 100.000 was 10.9. The mean (SD) age was 32 (23) years. Hand injuries were the most common. The most frequently operated regions were the upper limbs. A total of 18% of patients were admitted for two or more days, with the mean inpatient time of 12 (SD 29) days for women and 11 (19) days for men.

Conclusions:

Men experienced more burn injuries and were admitted to specialized care more frequently. While hand and wrist injuries were prevalent, they were seldom operated on. Women had a longer mean LOS than men aged 11–30 years.

P134

Burn care without borders—an evaluation of the basics of burn care website

**Simon, M.** ^1,6^, Nuwass, E. ^2^, Kalanzi, E. ^3^, Smit, L. ^4^, Rahmee, C. ^1,6^, de Vries, A. ^5,6^, Breugem, C. ^1^, Botman, M. ^1,6^

^1^ Department of Plastic and Reconstructive Surgery, University Medical Centre, Amsterdam, The Netherlands, Amsterdam, Netherlands, ^2^ Department of Surgery, Haydom Lutheran Hospital, Tanzania, Haydom, Tanzania, ^3^ Burns and Plastic Surgery Unit, Kiruddu National Referral Hospital, Republic of Uganda, Kampala, Tanzania, ^4^ The Online Scientist, Netherlands, ^5^ Department of Surgery, Burn Centre, Red Cross Hospital, Beverwijk, The Netherlands, Beverwijk, Netherlands, ^6^ Global Surgery Amsterdam, Amsterdam, Netherlands.

Poster presentations 2, 4 September 2025, 10:00–10:30

Aim:

Burn injuries cause an estimated 180,000 deaths annually and often result in prolonged hospital stays and disability. In low- and middle-income countries (LMICs), access to quality burn care is frequently limited by economic and geographic barriers. To address these challenges, we developed basicsofburncare.org, a freely accessible online platform offering up-to-date burn care education for healthcare professionals worldwide. This study evaluates the platform’s reach, user demographics, and feedback.

Methods:

From September 2024 to January 2025, the website attracted 12,536 visitors and 21,922 page views from 170 countries, with highest traffic from the United States, United Kingdom, Netherlands, and India. Most users (56%) accessed the site via smartphone. From January to March 2025, 37 users from 21 countries submitted feedback forms. Respondents included surgeons, nurses, physiotherapists, and students. Most were involved in clinical burn care or teaching. The majority (89%) indicated they would use the website in clinical practice and 86% would use it in training. Users rated the platform highly for satisfaction and content quality. Suggestions included more step-by-step tutorials, practical tips, downloadable teaching materials, and video demonstrations.

Conclusions:

The platform basicsofburncare.org has shown early success in delivering accessible, global burn education. Continued user feedback will guide further development to support frontline burn care providers worldwide.

P136

Augmented Reality for Medical Education in the Primary Survey of Burns.

**Vehmeijer-Heeman, M.** ^1^, van de Warenburg, M. ^1^

^1^ Radboud University Medical Center, Nijmegen, Nederland

Poster presentations 2, 4 September 2025, 10:00–10:30

Augmented reality (AR) technology is rapidly evolving, and is finding an increasing application in education, including medical training. This study aimed to explore the usability and didactic potential of AR with the HoloLens 2 (Microsoft, Redmond, WA, USA) for medical students, teaching the primary survey of burn wounds. The application was developed in collaboration with 3D Lab. 3D Photos of burn patients were assembled into a virtual model. Questionnaires were developed with the help of educational specialists.

Test groups consisted of medical students or nurses, residents or nurse specialists, and plastic surgeons, who filled in a short questionnaire after training with the HoloLens. Furthermore, 4 students or nurses, and 2 residents filled in a long questionnaire.

Outcomes of the questionnaires show AR to be a significant promising technology for educating medical students in the primary survey of burn wounds. Most medical students found AR to be realistic, motivating, and educational (78%). All students agreed that AR provides unique opportunities to practice scenarios that would otherwise be inaccessible, and experienced a safe learning environment using this headset. Furthermore, students expressed a wish for more AR-based education. However, there is a further need for development in usability and image quality. The ability to simulate realistic scenarios in a safe and scalable environment could pave the way for a new era for medical education, where AR becomes a valuable supplement of even replacement for traditional learning methods.

P137

Spray-delivered adipose-derived mesenchymal stem cells for severe burn wound treatment: an in vitro study.

**Winther-Waldau, V.** ^1,3^, Jenssen, A. ^1,3^, Mohamed-Ahmed, S. ^2^, Kankuri, E. ^4^, Mustafa, K. ^2^, Kreken Almeland, S. ^1,3^

^1^ Research Group for Burn Care and Reconstructive Surgery, Department of Clinical Medicine, Faculty of Medicine, University of Bergen, Bergen, Norway, ^2^ Center for Translational Oral Research (TOR), Tissue Engineering Group, Department of Clinical Dentistry, Faculty of Medicine, University of Bergen, Bergen, Norway, ^3^ Norwegian National Burn Center, Department of Plastic, Hand, and Reconstructive Surgery, Haukeland University Hospital, Bergen, Norway, ^4^ Department of Pharmacology, Faculty of Medicine, University of Helsinki, Helsinki, Finland.

Poster presentations 2, 4 September 2025, 10:00–10:30

Aim:

This study aims to evaluate viability, proliferation and immunophenotype of adipose-derived mesenchymal stem cells (ASC) when sprayed using a fibrin glue spray system.

Background:

Major burns are serious injuries requiring specialized care, but advancements in wound care are still needed to improve outcomes. Mesenchymal stem cells, particularly ASC show promising therapeutic potential due to their regenerative properties. Additionally, spraying ASC on large burn wounds may provide an efficient delivery method.

Methods: 

Human ASC were encapsulated in fibrinogen and sprayed using a fibrin glue spray system, with 1 or 1.8 bar pressure, at 10 or 20 cm distance, before adding thrombin. Casted ASC without spraying were used as control. During culture for 7 days, viability, morphology and proliferation of ASC were assessed using Live/Dead staining, metabolic activity assay, actin-cytoskeleton staining, and DNA quantification. Cellular immunophenotype was examined using flow cytometry.

Results: 

Like casted ASC, sprayed ASC maintained high viability with good distribution and increasing metabolic activity during culture up to day 7. Spraying has not influenced ASC ability to spread within the fibrin glue, having a similar morphology to casted ASC. Sprayed ASC demonstrated a comparable proliferation rate to casted ASC, confirmed by higher DNA quantity from day 1 to day 7. No changes were observed in ASC immunophenotype after spraying. Generally, increasing spraying pressure and distance did not negatively impact the ASC.

Conclusions: 

ASC can be efficiently sprayed on large burn wounds using fibrin glue spray system without compromising their viability and characteristics.

P143

Family satisfaction with the burn center: A qualitative analysis.

**Falk, J.** ^1^, Lindblad, M. ^1,2^, Leander, K. ^1^, Huss, F. ^1,2^

^1^ Uppsala Burn Center, Uppsala University Hospital, Uppsala, Sweden, ^2^ Department of Surgical Sciences, Plastic Surgery, Uppsala University, Uppsala, Sweden.

Poster presentations 2, 4 September 2025, 10:00–10:30

Aim:

To gain an increased understanding of how relatives of burn patients experience the care at Uppsala Burn Center.

Methods:

The method used for the study was the questionnaire ‘Family Satisfaction in the Intensive Care Unit 24‘ (FS-ICU 24R). The questionnaire consisted of Likert scale questions and open-ended questions. The answers to the open-ended questions were selected for a qualitative content analysis. The family members who were asked to participate in this study were ICU or StepDownUnit/ward patients. Data was collected from the next of kin to patients admitted to the burn center from September 2016 to December 2024. The result presented is an interim analysis.

Results:

Eighty-four questionnaires from sixty-three patients were collected for the results. The qualitative content analysis resulted in three main themes based on the answers: Communication, environment, and professionalism. The negative comments concerned transfers from the burn unit to their remitting hospital when the burn care no longer needed the plastic surgery specialty. Positive comments included praise for the professional treatment the patient received from the health care staff.

Conclusions:

The care at the burn center was of high quality with professional staff. However, more consistent information from the doctors and nurses is needed to improve satisfaction.

P144

Implementation and management of the keratinocyte process in the burn patient

**Gonzalez Estrella, S.** ^1^, Braña Campos, S. ^1^, Torrecilla Rosado, G. ^1^, Manrique, G. ^1^, Pereira Gonzalez, J. ^1^

^1^ Hospital Universitario La Paz, Madrid, Spain

Poster presentations 2, 4 September 2025, 10:00–10:30

Title: Implementation and management of the keratinocyte process in the major burns unit of the Hospital Universitario La Paz.

Aim:

The aim of this case report is to implement through the nursing staff a protocol on the use and management of keratinocytes in the major burns and polytrauma unit of the Hospital Universitario La Paz.

Methods:

This case report is a descriptive observational study based on the implementation of keratinocytes in a patient with a total burned body surface area of 45%.

Results:

The standardized protocol ensures greater consistency in treatment application, leading to increased effectiveness and fewer complications. The role of nursing is crucial, as their expertise ensures proper implementation of the protocol, enhances patient care and optimizes treatment outcomes. The implementation of keratinocyte therapy presents several challenges, including its complex technique, high cost, and prolonged treatment duration. These factors emphasize the need for strict protocol adherence and specialized nursing care to optimize outcomes and reduce inconsistencies in treatment effectiveness.

Conclusions:

The lack of ideal skin coverage in the burn patient is a reality. Cell-based therapeutic strategies are becoming increasingly important in the treatment of burns by creating an environment that allows rapid re-epithelialisation and accelerates cell proliferation, minimises pain sensation in the patient, improves aesthetic outcome and prevents infection. The use of this type of therapy may be a solution for earlier intervention, as it decreases complications associated with the donor site, as well as reducing morbidity.

P145

Clinical outcomes after the implementation of a dynamic alginogel-based protocol in partial-thickness burns post enzymatic debridement.

**Gonzalez Gilarte, L.** ^1^, Barroso Sala, M., Miguel Puigbarraca, P., Rubio Garcia, J.

^1^ Hospital Universitario Vall D’hebron, Barcelona, Spain

Poster presentations 2, 4 September 2025, 10:00–10:30

Aim:

To evaluate the clinical outcomes following the implementation of a dynamic wound care protocol using Flaminal^®^ in patients with partial-thickness burns after enzymatic debridement.

Methods:

A retrospective analysis was carried out on patients treated with Flaminal^®^ Hydro or Flaminal^®^ Forte following enzymatic debridement. The dynamic protocol was developed by the nursing team of the Burn Unit, allowing for the adaptation of local treatment based on the clinical evolution of the wound. Variables analyzed included the number of dressing changes required, infection rate, exudate progression, burn depth, need for surgical intervention, time to complete epithelialization, and pain reported during dressing changes.

Results:

The implementation of the protocol led to a reduction in the number of required dressing changes per patient, with adequate control of exudate and a low rate of infection. Most 2B burns epithelialized without the need for skin grafting. The protocol supported individualized, structured, and effective care, allowing for consistent clinical decision-making.

Conclusions:

A dynamic protocol based on Flaminal^®^ significantly improves clinical outcomes in partial-thickness burns post enzymatic debridement, reducing the need for frequent dressing changes, minimizing infections, and enhancing patient comfort. These results support its integration into specialized burn nursing practice as a valuable and adaptable care tool.

P146

Enhancing Burn Care through a standardized wound protocol

**Batist, M.** ^1^, Hofland, H. ^1^, Jansen, K. ^1^, van der Vlies, K. ^1,2^

^1^ Burn Center Rotterdam Maasstad Hospital, The Netherlands, ^2^ Trauma Research Unit Department of Surgery, Erasmus MC, University Medical Center Rotterdam, The Netherlands.

Poster presentations 2, 4 September 2025, 10:00–10:30

Aim:

The aim of this project was to develop and implement standardized wound care protocols to enhance decision-making processes.

Introduction:

At the Burn Center of the Maasstad Hospital in Rotterdam, a range of topical agents is utilized based on wound depth, affected total body surface area (TBSA), and patient preference. Recently, the enzyme alginogel (Flaminal^®^) was introduced due to its safety profile, debriding capability, and exudate management properties, becoming the preferred treatment for partial and deep partial-thickness burns. However, its implementation was met with resistance from some staff accustomed to prior treatment routines, causing inconsistencies in wound care practices and confusion among burn care nurses.

Method:

To address this, the Expert Wound Care Team (EWCT)—comprising burn center and outpatient nurses, management, and medical staff—conducts bi-monthly evaluations of treatment strategies. Recognizing inconsistencies in topical agent use, the team developed standardized wound care protocols, incorporating two structured flowcharts: one for hospitalized patients and another for outpatient care.

Results:

The implementation of these protocols streamlined decision-making regarding topical agent selection, ensuring a systematic approach based on %TBSA, burn depth, and infection status. Additionally, patient preferences were integrated into care decisions. The structured methodology improved consistency and was well received by the burn care team.

Conclusions:

The introduction of standardized wound care protocols has enhanced decision-making processes and provided clarity for both nursing and medical staff. This structured approach has optimized burn treatment strategies, and future research should evaluate its long-term impact on clinical outcomes and patient satisfaction.

P147

Enzyme Alginogel (Flaminal^®^) treatment in burn injuries: the Rotterdam Burn Center experience.

**Hofland, H.** ^1^, Batist, M. ^1^, Lucas, Y. ^1^, van der Vlies, C. ^1,2^

^1^ Burn Center Rotterdam Maasstad Hospital, The Netherlands, ^2^ Trauma Research Unit Department of Surgery, Erasmus MC, University Medical Center Rotterdam, The Netherlands.

Poster presentations 2, 4 September 2025, 10:00–10:30

Aim:

The aim of this project was the implementation of a new topical agent in the Rotterdam Burn center.

Introduction: Enzyme Alginogel (Flaminal^®^) was introduced in the Rotterdam Burn Center in 2019. Whereas Silversulphadiazine (Flamazine^®^) was the preferred choice Flaminal^®^ was slowly initiated for treatment of partial and deep partial thickness burns. Flaminal^®^ is a topical agent that is safe for skin and wound tissue, can debride and remove necrotic tissue and manage wound exudate. It has two products: Flaminal^®^ Hydro and Flaminal Forte that is indicated for moderate to high-exudating wounds.

Methods:

To instruct staff how to use Flaminal^®^ was difficult. Complaints were that it would stuck to the wound, patients experienced more pain or it was smelly. However, proper instructions were not always followed or caused misunderstanding. Instruction lessons and bed- side teaching was necessary to get familiar with how to apply this topical agent and when to remove the crusts on the edges of the wounds.

Results:In time the benefits of using this topical agent became apparent. Patients are dressed every other day instead of daily and it is easy to apply even in difficult areas. Flaminal^®^ became the standard choice.

Conclusions:

Flaminal^®^ is now the preferred topical agent for (deep) partial thickness burn injuries and is used in the clinic as well as the Out Patient Department with good results and to full satisfaction of staff. With proper implementation staff will learn how to apply both Flaminal^®^ Hydro and Forte, and how to assess the indication.

P148

Gunpowder Burns: Updates, Treatments, and Our Experience.

**Marín Tebar, S.** ^1^, Ivañez Mari, B. ^2^, Roig Roig, G. ^3^, Pérez del Caz María, M. ^4^

^1^ Hospital Universitari i Politècnic La Fe, Valencia, Spain, ^2^ Hospital Universitari i Politècnic La Fe, Valencia, Spain, ^3^ Hospital Universitari i Politècnic La Fe, Valencia, Spain, ^4^ Hospital Universitari i Politècnic La Fe, Valencia, Spain.

Poster presentations 2, 4 September 2025, 10:00–10:30

Title: Gunpowder Burns: Updates, Treatments, and Our Experience.

Aim:

The aim of this presentation is to provide a comprehensive guide on the first aid and treatment of gunpowder burns, which are common injuries during fireworks-related celebrations and festivals.

Methods:

The Presentation is based on reviews of existing literature on the management of gunpowder burns based on our annual cases and focuses on the first aid measures, treatment protocols, and the assessment of burn severity.

Results:

The presentation identifies key first aid practices such as cooling the burn under running cold water, removing pressure-inducing objects, and using sterile dressings. In addition it also highlights the importance of proper wound cleaning and the need for professional medical intervention in severe cases. The research underscores the need for prompt treatment, particularly for deep or extensive burns, and recommends monitoring for signs of infection.

Conclusions:

The presentation concludes that immediate first aid and appropriate aftercare are essential in managing gunpowder burns. Preventive measures, such as proper supervision during firework displays, are critical in reducing injury risk. Further research into the long-term outcomes of severe burns and the effectiveness of specific treatments is warranted.

P149

One Family—Two Burn Centers. A Comparison of Treatment of Adults and Children

**Rosch, C.** ^1^, Beck, K. ^2^, Thurnheer, A. ^2^

^1^ Universitätsspital Zürich, Zuerich, Switzerland, ^2^ Kinderspital Zürich, Zuerich, Schweiz

Poster presentations 2, 4 September 2025, 10:00–10:30

Aim and Methods:

This case report compares the physical and psychosocial treatment of adults and children with burns at two specialized centers in Zürich. The case report involves a family of four who sustained severe burn injuries and were treated at the University Hospital Zürich (USZ) and the Children’s Hospital. The treatment teams faced significant challenges due to severe inter-familial conflicts.

Results:

- Wound care management for adults and children is similar, involving regular dressing changes and surgical interventions. However, adult care is more constrained by financial and usability considerations.
- The mother’s treatment was complicated by chronic wound infections and multi-organ dysfunction, leading to severe weight loss and prolonged unconsciousness.
- Professional exchange between the two centers facilitated the adaptation of treatment concepts.
- Inter-familial conflicts consumed substantial time and energy, particularly affecting children when conflicts led to visitor restrictions and information gaps.
- Confidentiality rules and legal uncertainties constrained information exchange between the centers.
- The adult burn center’s family care system was insufficiently adapted to extreme inter-familial conflicts, making it challenging to inform and accompany children visiting their mother in the ICU.

Conclusions:

- Both treatment teams’ sense of responsibility and commitment helped overcome cooperation obstacles.
- Coordinated cooperation and common goals were lacking, despite prioritizing children’s wellbeing.
- Systematic information exchange and cooperation from admission could prevent information gaps and protect children from familial conflict effects.
- Coordinated family meetings and immediate involvement of hospital legal services would enhance legal certainty and support for the family.

P150

The relationship between health literacy and attitudes towards the use of complementary therapies in patients with burns in Turkiye.

Tuncbilek, Z. ^1^, **Acikgoz, F.** ^1^, Yalaniz, B. ^1^, Karahan, S. ^2^, Cennet, O. ^1^

^1^ Hacettepe University, Ankara, Turkey, ^2^ Medizinische Hochschule Hannover, Hannover, Germany.

Poster presentations 2, 4 September 2025, 10:00–10:30

Aim:

The aim of this study is to determine the relationship between the attitudes toward using complementary therapies and the health literacy of patients with burns.

Methods:

This study was conducted as a descriptive and correlational study, including adult patients treated in the burn unit of a university hospital in Turkiye. Study sample consisted of 131 patients. After obtaining ethical approval, patient data were collected through face-to-face interviews or online via Google Forms using the Patient Identification Form, the Attitude Scale Toward Using Complementary Therapies and the Health Literacy Scale.

Results:

41 patients (31.3% of the sample) had been reached between 25 November 2024–28 March 2025 in this ongoing study. The mean age of the patients was 37.24 ± 11.50 (18–64) years, with 61% being female and 41.5% being high school graduates. Many patients had 1–15% TBSA (95.1%), second-degree burns (90.2%), and 46.4% had scald burns. Burns were most located on the hands (39%) and arms (31.7%). Only 12.2% of the patients used complementary therapy. The mean attitude score toward using complementary therapies was 15.93 ± 6.48 (5–36), while the mean health literacy score was 110.32 ± 15.24 (55–125). A weak and statistically insignificant negative correlation was found between these two variables (r = −0.072, *p* = 0.654).

Conclusions:

Based on the existing findings, it has been determined that patients with burn have a negative attitude toward using complementary therapies and have an adequate level of health literacy.

P151

Retrospective study of cultured epithelial autografts (CEAs) in severely burned patients.

**Abdel-Sayed, P.** ^1^, Georges, M. ^1^, Al-Dourobi, K. ^1^, Guichoud, Y. ^1^, Bruhlmann, J. ^1^, Applegate, L., de Buys Roessingh, A. ^1^

^1^ Lausanne University Hospital (chuv), Lausanne, Switzerland

Poster presentations 2, 4 September 2025, 10:00–10:30

Aim:

Retrospective evaluation of safety of cultured epithelial autografts (CEAs) in a cohort of patients treated for deep burns at our University hospital (CHUV) between 2003 and 2021.

Methods:

Following approval from the State Ethics Committee (CER-VD), clinical data were collected using a REDCap database. The inclusion criteria were: (i) Male or female adults (≥18 years) with ≥20% total body surface area (TBSA) burns, or equivalent injuries as defined by EBA guidelines; (ii) Male or female children (<18 years) with ≥10% TBSA burns, or equivalent injuries; (iii) Treatment with CEAs; (iv) Availability of relevant clinical data. Exclusion criterion was a documented refusal to participate by the patient or their legal representative.

Results:

A total of 106 patients treated with CEAs at the CHUV were included. Clinical data have been collected for 26 patients to date. Mean patient age was 33.71 ± 16.83 years. Mean TBSA of deep burns was 43.10 ± 26.17%. Survival rate during hospital stay was 96.0%. No skin tumors associated with CEA grafting were reported to date and the most common complication was scar contraction in the extremities (40.7%). Acute phase adverse events were primarily infections (total 51% to date), for which skin infection events represented only 13.3%.

Conclusions:

Preliminary findings confirm the ongoing safety assessment of CEAs in the surgical treatment of severe burns which contribute valuable evidence to support safe continued use and further investigation of efficacy for the ongoing market authorization process for CEAs for burn treatment in Switzerland.

P152

Study on The Correlation Between Epidemiological Characteristics of Burn Patients and The Bacteriological Profile

**Akın, M.** ^1^, Gürler, M. ^2^, Akgün, A. ^1^, Çamöz, M. ^1^, Yurdakul, Z. ^1^, Kırca, F. ^2^, Çöplü, N. ^2^, Dinç, B. ^2^

^1^ Ankara Bilkent City Hospital-General Surgery, Ankara, Türkiye, ^2^ Ankara Bilkent City Hospital—Medical Microbiology, Ankara, Türkiye.

Poster presentations 2, 4 September 2025, 10:00–10:30

Aim:

Infection in burn patients remains the significant source of morbidity/mortality.The objective of this research was to assess the distribution of microorganisms isolated from samples of patients hospitalized in Burn Units and to explore how demographic characteristics and causes of burns in patients influence this distribution.

Methods:

We retrospectively analyzed all clinical specimens received at laboratory from patients at BU between January 2020 and June 2024.Demographic, clinical and epidemiological data of 179 patients from whom microorganisms were isolated in any sample were obtained from the system.

Results:

The study included a total of 685 samples collected from 179 patients. Most of the patients with microorganisms detected in their samples were those in the burn intensive care unit. 68.7% of the included patients were male. Fire (57.5%) was the main etiological agent for burn injuries. The majority of patients had 3rd degree burns (50.8%). The mean hospitalization time of the patients 38.3 days. Overall mortality rate is 33.5%. The most frequently sent sample type was the blood/catheter sample (60.4%). The most frequently isolated bacteria were *Pseudomonas aeruginosa* (26.6%) and *Staphylococcus aureus* (19.3%). It is noteworthy that the rates of *Candida* spp increased after 2022 and that *Acinetobacter baumannii* was not detected in 2024. There was no significant difference in the types of microorganisms found between patient groups with varying degrees of burns or length of hospital stay.

Conclusions:

Determining the most common bacteria in infections in burn patients and revealing the infection-related risk factors of these patients are necessary to prevent serious complications and to manage the situation in the best way.

P153

Therapeutic Drug Monitoring of Antimicrobials in Burn Patients: Are We Reaching the Targets?

**Arévalo Bernabé, Á.** ^1^, Baena Caparrós, J. ^1^, Rodríguez Pardo, D. ^1^, Pau Parra, A. ^1^, García García, S. ^1^, Lalueza Broto, P. ^1^, Marquina Escoz, S. ^1^, Bulla, A. ^1^, Serracanta Doménech, J. ^1^

^1^ Hospital Universitari Vall D’hebron, Barcelona, Spain

Poster presentations 2, 4 September 2025, 10:00–10:30

Aim:

To evaluate plasma antimicrobial concentrations in a cohort of burn patients to assess their compliance with established pharmacokinetic targets and support the optimization of anti-infective therapy, given that infections are the leading cause of morbidity and mortality in this population.

Methods:

This single-center ambispective observational study included all adult patients admitted for burn injuries to the Burn Unit of a tertiary care hospital between 03/2022 and 03/2025, with at least one plasma antimicrobial concentration measured during hospitalization. Biodemographic, clinical, and pharmacological data were obtained from the SAP^®^ electronic medical record, and laboratory data from the Modulab^®^ system. A univariable descriptive statistical analysis was performed using Microsoft Excel^®^.

Results:

Thirteen patients (84.6% male; 76.9% classified as major burns) were included. Median age was 55 years (IQR: 44–60). Thermal burns were most common (69.2%: deflagration 67%, flame 22%, scald 11%), followed by electrical (23.1%) and chemical (7.7%) burns. Median%TBSA was 60% (IQR: 27–70). A total of 34 plasma determinations (mean number of determinations per patient: 2.6 ± 0.4) were performed: 17 (50%) showed subtherapeutic levels (16 linked to augmented renal clearance, defined by a creatinine clearance of >130 mL/min/1.73 m^2^), 15 (44%) were therapeutic, and 2 (6%) were potentially toxic. Teicoplanin (*n* = 10), meropenem (*n* = 9), and isavuconazole (*n* = 5) were most frequently monitored.

Conclusions:

Therapeutic Drug Monitoring (TDM) in burn patients revealed frequent subtherapeutic antimicrobial exposures, often related to augmented renal clearance. Standardized TDM emerges as a key strategy to optimize efficacy and safety of anti-infective therapy in this high-risk population.

P154

Evaluating Infection Control in Burn Intensive Care Unit: A 7-Year Assessment of the ZERO Programs

**Arévalo Bernabé, Á.** ^1^, Jacinto, B. ^1^, Marquina Escoz, S. ^1^, Selva Armadans, I. ^1^, Palmada Ibars, C. ^1^, Lalueza Broto, P. ^1^, González Gilarte, L. ^1^, Serracanta Doménech, J. ^1^

^1^ Hospital Universitari Vall D’hebron, Barcelona, Spain

Poster presentations 2, 4 September 2025, 10:00–10:30

Aim:

To evaluate the incidence and trends of catheter-related bloodstream infections (CRBSI), catheter-associated urinary tract infections (CAUTI), and ventilator-associated pneumonia (VAP) in critically burned patients to identify potential areas for safety improvement.

Methods:

A retrospective observational study was conducted including all adult critically burned patients admitted to the Burn Intensive Care Unit (ICU) of a tertiary referral hospital from June 2018 to December 2024. Data from 232 patients were analyzed following national ZERO project protocols and extracted from the ENVIN registry (National Nosocomial Infection Surveillance Study in Intensive Care Units). A univariate descriptive analysis was performed. Incidence rates are expressed as means ± standard deviation.

Results:

The mean incidence rates per 100 admissions were 8.19 for CRBSI (*n* = 19), 9.48 for CAUTI (*n* = 22), and 25.43 for VAP (*n* = 59). These rates were significantly higher than the Catalonian (CRBSI: 0.86; CAUTI: 1.02; VAP: 1.50) and national general ICU averages (CRBSI: 1.29; CAUTI: 1.44; VAP: 1.82), attributed to the unique complexity of burn patients. No comparative data from other burn ICUs were available. Over time, CRBSI rates improved, while VAP showed an increasing trend in the last two years. CAUTI rates improved notably post-ITU ZERO accreditation in 2019, although a slight rebound was observed in 2024.

Conclusions:

Despite partial improvements in device-associated infection rates, these remain high due to the distinct pathophysiological vulnerability and complex clinical course characteristic of critically burned patients. The data indicate a need for ongoing education and reinforcement of preventive measures. Continuous implementation, evaluation and reinforcement of ZERO protocols are essential to improve patient safety in this population.

P155

Characteristics, treatments, and outcomes of patients with Necrotizing Soft Tissue Infections: A Dutch multicentre cohort study

**de Haan, J.** ^1,2^, M.F.H. Neeter, L.^1^, Suijker, J. ^1,2^, P.M. van Zuijlen, P. ^1,2,3^, Pijpe, A. ^1,2^, Meij-de Vries, A. ^1,4^, NSTI Knowledge Collaborative Group

^1^ Alliance of Dutch Burn Care (ADBC), Burn Centre, Red Cross Hospital, Beverwijk, The Netherlands, ^2^ Department of Plastic, Reconstructive and Hand Surgery, Amsterdam UMC Location Vrije Universiteit, Amsterdam, The Netherlands, ^3^ Department of Plastic and Reconstructive Surgery, Red Cross Hospital, Beverwijk, The Netherlands, ^4^ Department of Surgery, Red Cross Hospital, Beverwijk, The Netherlands

Poster presentations 2, 4 September 2025, 10:00–10:30

Aim:

This study aimed to gain insights into patient-, disease-, and treatment characteristics, as well as clinical outcomes of patients with necrotizing soft tissue infections (NSTI) in The Netherlands, thereby contributing to our global knowledge of NSTI.

Methods:

The NSTI Knowledge Project cohort, which includes retrospectively collected data from 271 patients treated for acute NSTI in one of 11 Dutch hospitals between 2013 and 2017, was analysed descriptively.

Results:

Most patients (61%) presented with symptoms non-specific for NSTI, such as pain, erythema, and swelling. These patients had significantly higher misdiagnosis rates (*p* = 0.019) and longer debridement delays (*p* = 0.009) compared to those presenting with specific symptoms, such as skin necrosis. Group A Streptococcus was cultured in 41% of patients. Intensive care unit admission was required in 83%, with a median stay of 5 days (IQR 2–11). The median time from hospital admission to the first debridement was 8 h (IQR 4–23). The in-hospital mortality rate was 21%, and 12% of patients ultimately required extremity amputation.

Conclusions:

In The Netherlands, NSTI patient- and disease characteristics vary considerably. With one in five patients dying, and one in eight patients undergoing a major amputation, interventions leading to rapid diagnosis and optimal treatment are urgently needed.

P158

Our experience in treating military burn injuries.

**Kovalenko, O.** ^1^, Kovalenko, A. ^2^, Linnik, A. ^3^

^1^ Bogomolets National Medical University, Kyiv, Ukraine, ^2^ KNP Kyiv City Clinical Hospital No. 2 Burn Clinic, Kyiv, Ukraine, ^3^ Shupyk National Healthcare University of Ukraine, Kyiv, Ukraine.

Poster presentations 2, 4 September 2025, 10:00–10:30

Purpose:

To analyze the treatment of severe military burns in 2024

Materials and methods:

88 patients were under observation who were treated during 2024 in the burn center, who were delivered from the stages of evacuation from the battlefield. The burn center was a third hospital for these patients.

Results:

the wounded were admitted on 5th on 5.9± 2.1 (3–10) days after the injury. The age was 37.5 ± 7.3 (25–52). The lesion area was 48.6 ± 7.9% of the TVSA (40–70). The wounds of all patients were infected with polymorphic trench and hospital microflora from the previous stages. The wet necrosis did not allow for excision. *Acinetobacter baumannii* was mainly found—30% In the wounds, *Pseudomonas aeruginosa*—32.3%., also Enterococcus faecium, Klebsiella pneumonia and others. Microbial colonization was lg (7.05 ± 0.4) and lg (8.05 ± 0.5) CFU/g. Patients underwent treatment using hyperosmolar antibacterial ointments. Wet necrosis was converted to dry necrosis then was removed.

Antibacterial therapy was carried out taking into account the sensitivity and synergism of antibiotics (two antibiotics at the same time). Autograft was started on 22.5 ± 3.1 days. Excision of necrosis was started on 5.1 ± 1.3 days after hospitalization in the burn center and on 12.1 ± 2.8 days after injury. Wounds were temporarily closed with a xenograft.

Conclusions:

The number of septic complications increased twice in the group of patients more than 40% TBSA, mortality increased 1.5 times. Hyperosmolar ointments and two antibiotics prescribed simultaneously and delayed surgery allowed to stabilization of these patients.

Key words: military burns, infection, delayed surgery

P159

Effects of bromelain enzymatic debridement on colonization rates and sepsis in burn patients: a cohort study.

**Minic, J.** ^1^, Pezzani, M. ^2^, Morra, M. ^2^, Tacconelli, E. ^2^, Governa, M. ^1^

^1^ Burn Unit, Ospedale Civile Maggiore, Verona, Italy, ^2^ Infectious Diseases Section, Department of Diagnostics and Public Health, University of Verona, Verona, Italy

Poster presentations 2, 4 September 2025, 10:00–10:30

Aim:

This study aimed to evaluate the association between bromelain-based enzymatic debridement (ED) and the risk of bloodstream infections (BSIs) in critically ill burn patients compared to surgical debridement (SD) at Verona University Hospital’s Burn Center.

Methods:

We included patients with deep partial or full-thickness burns admitted between January 2020 and December 2023. Data on demographics, total body surface area (TBSA), treatment type (ED/SD), and colonization status (via weekly swabs) were collected. Propensity score was applied to estimate the likelihood of receiving ED and to control for confounding variables. The primary analysis compared BSI rates between the two groups, once adjusting for residual imbalance due to length of stay (LOS).

Results:

Of 238 patients, 85 (36%) received ED, and 153 (64%) underwent SD. Median age was 54 years; ED patients had higher median TBSA (20% vs. 12%, *p* < 0.001) and longer LOS (31 vs. 16 days, *p* < 0.001). BSIs occurred in 21 patients (17 in ED), with 38% linked to multidrug-resistant (MDR) bacteria matching skin colonization. MDR colonization affected 12.6% of patients. Mortality was 7% (no significant ED vs. SD difference). After LOS adjustment, ED showed no significant BSI risk increase (OR = 2.29, 95% CI: 0.5–10.0, *p* = 0.269), while LOS was a key predictor (OR = 1.06, 95% CI: 1.03–1.09, *p* < 0.001). Colonization patterns were similar, shifting from Gram-positive to Gram-negative bacteria over time.

Conclusions:

Bromelain-based ED was not significantly associated with increased BSI risk in burn patients, suggesting it remains a safe alternative to SD.

P160

Necrotizing soft tissue infections in The Netherlands: preliminary results of a snapshot study.

**Neeter, L.** ^1^, Pijpe, A. ^1,2^, de Haan, J. ^1,2^, van Zuijlen, P. ^1,2,3^, Meij-de Vries, A. ^1,4^, Core group NSTI Network Netherlands, NSTI snapshot study group

^1^ Alliance of Dutch Burn Care (ADBC), Red Cross Hospital, Beverwijk, The Netherlands, ^2^ Amsterdam Movement Sciences, Tissue Function and Regeneration, Amsterdam UMC, Amsterdam, The Netherlands, ^3^ Department of Plastic and Reconstructive Surgery, Red Cross Hospital, Beverwijk, The Netherlands, ^4^ Department of Surgery, Red Cross Hospital, Beverwijk, The Netherlands.

Poster presentations 2, 4 September 2025, 10:00–10:30

Aim:

The aim of the necrotizing soft tissue infection (NSTI) snapshot study was to retrospectively register NSTI patients in The Netherlands over the year 2023 and to gain insight into patient, disease, referral, and treatment characteristics.

Methods:

This retrospective snapshot study was held among eighteen Dutch hospitals. The core group of the NSTI Network Netherlands determined the minimal important dataset: age, sex, location of NSTI, pathogen(s) inducing NSTI, mortality, length of (ICU) stay (LOS), sepsis, amputation/enucleation, skin-sparing debridement, and referral information. Data of all patients admitted in 2023 were collected from their patient files.

Results:

Results from ten hospitals so far show that 61 of the 101 (60%) patients were male with mean age of 60 (SD ± 14) years. NSTI was mainly induced by GAS and localization of NSTI was most common in the legs (36%) and anogenital area (44%). Median LOS (admissions = 104) and LOS ICU (admissions = 68) were 17 (IQR: 11–32) and 4 days (IQR: 3–8), respectively. (Complete) skin-sparing debridement was often performed (62%). Two patients (2%) required amputation of toes and leg. Most patients (59%) came in via the emergency room, 28 patients (28%) were referred from another hospital of which three to another participating hospital, and 50 patients (50%) were discharged to their own home. By the end of 2024, 22 patients (21%) were deceased.

Conclusions:

Skin-sparing debridement was performed in the majority of NSTI patients in 2023. The mortality rate, was with 21% substantial among NSTI patients. More data is expected in Q2 2025.

P161

The characteristics of Indonesian local honey compared to manuka honey in terms of physical-chemical components, unique manuka factor, and antibacterial effects

**Putri, N.** ^1^, Dewi, F. ^1^, Kreshanti, P. ^1^, Tjampakasari, C. ^2^, Tunjung, N. ^3^, Oklia, S. ^4^

^1^ Universitas Indonesia, Jakarta, Indonesia, ^2^ Department of Microbiology, Universitas Indonesia, Jakarta, Indonesia, ^3^ Universitas Indonesia hospital, Depok, Indonesia, ^4^ Ciptomangunkusumo national hospital, Jakarta, Indonesia

Poster presentations 2, 4 September 2025, 10:00–10:30

Honey has been used for its medicinal properties. Manuka honey and Medihoney™ (Comvita, Te Puke, New Zealand) have been widely recognized for their effectiveness in treating ulcers, infected wounds, and burns. Their high cost and availability make them difficult to use in Indonesia. Previous studies have evaluated in physiochemical activity between Nusantara (local) honey and Manuka Honey. However, this study expands the analysis by including more local honey varieties and assessing their antimicrobial potential. Aims: This descriptive analytical study aimed to compare Indonesian local honeys and Manuka honey antimicrobial potential activity. Methods:

We compared local honeys and manuka honey in terms of their physicochemical characteristics, methylglyoxal (MGO) content as the Unique Manuka Factor, and antimicrobial effect against *Kelbsiella pneumoniae* ATCC 13883, *Pseudomonas aeruginosa* ATCC 27853, *Staphylococcus aureus* ATCC 25923, *Enterobacter cloacae* ATCC 23355, and *Escherichia coli* ATCC 25922. Results:

The results showed that New Zealand manuka honey had a lower pH, higher acidity, viscosity, and sugar content compared to Indonesian local honey. Manuka honey also had a higher MGO and NPA. Between the local honeys, Nusantara honey showed a higher MGO levels than Java honey. In terms of antibacterial activity, Manuka honey demonstrated stronger effect against *P. aeruginosa*, *K. Pneumonia*, and *S. aureus* compared with Indonesian local honey. Despite this, Nusantara honey showed a comparable antibacterial effect to New Zealand Manuka honey UMF 5 +. Conclusions: This study provides baseline data to help develop local medical-grade honey that can be competitive with international medical-grade products, offering a more accessible option for Indonesia.

P162

Large area cold plasma patch therapy as a new approach in the treatment of burns with multi-resistant pathogen colonization.

**Schlottmann, F.** ^1^, Milewksi, M. ^1^, Kloss, F. ^1^, Lorbeer, L. ^1^, Wellkamp, L. ^1^, Dieck, T. ^1^, Vogt, P. ^1^

^1^ Hannover Medical School, Hannover, Germany

Poster presentations 2, 4 September 2025, 10:00–10:30

Aim:

This study aims to evaluate the efficacy of atmospheric cold plasma therapy (ACPT) in the treatment of complex wounds colonized with multidrug-resistant (MDR) pathogens. We investigated its potential for treating burn wounds with MDR colonization, addressing a critical need in burn care management.

Methods:

This case series included patients with extensive burn wounds colonized by MDR pathogens, who underwent a multimodal treatment approach consisting of topical antisepsis, surgical debridement, systemic anti-infective therapy, and ACPT for microbial reduction and elimination. The therapeutic effect of ACPT is based on the interaction of reactive oxygen and nitrogen species with cells and tissues, exhibiting antimicrobial effects and promoting wound healing. The CPT^®^Cube device (COLDPLASMATECH, Greifswald, Germany) enable automated, large-area cold plasma application, ensuring reproducible treatment conditions.

Results:

All patients exhibited MDR pathogen-colonized wounds, where topical or systemic anti-infective measures alone were insufficient for pathogen control. Large-area ACPT application resulted in a significant microbial load reduction, enabling satisfactory wound healing. The combination of ACPT with existing therapeutic measures ensured successful skin graft integration and prevented infections. As a result of the acquired expertise, our department was certified as the first German center for cold plasma treatment of burn wounds.

Conclusions:

Large-area ACPT represents a novel and effective approach for treating complex wounds, combining potent antimicrobial effects with enhanced wound healing. This dual mechanism makes ACPT a valuable addition to the current therapeutic arsenal in plastic surgery. Larger case series and randomized controlled trials are needed to further assess ACPT’s role in future clinical practice.

P163

Fish Skin Graft Transplantation (Type Kerecis) In A Progressive *Pseudomonas aeruginosa* Contaminated Distal Wound of the Lower Right Extremity.

**Sharafi, S.** ^1^, Leenhouts, P. ^1^, Arigoni, M. ^1^, Schreiber, V. ^1^

^1^ Spital Uster Ag, Uster, Switzerland

Poster presentations 2, 4 September 2025, 10:00–10:30

Aim:

With this study case, we proofed the sufficiancy and regenerative power of Kerecis Fish Skin Grafts in Pseudomonas contaminated wounds by speeding up consolidation time, preventing superinfection, reducing pain and wound progress.

Methods:

- PTA initially
- Surgical debridement
- Kerecis Graft 3 × 7 cm (Initial local infiltration, bedside application, partiall graft stacking in wound depth, rest graft on top of stacks, fixation of top graft piece with stiches, Mepitel application, fixation with steri strips, absorber on top)
- Wound controll postoperatively after 4 days
- 2nd wound control plus complete dressing change 7 days p.o.
- Weekly changes till final consolidation
- Second grafting after four weeks approx.

Conclusions:

This case underlines and puts emphasis on the major consolidation effect of Kerecis on contaminated and progressive wounds, such as MRSA or Pseudomonas contaminated wounds. In contrast to conservative wound management, Kerecis sufficiently manages to control and reduce patient’s pain by reducing wound depth and size continuously up to final closure with a pain free patient at the end.

Kerecis has shown it’s “bacteriostatic” potential. Our study proves the capability of Kerecis to close wounds faster plus reduce pain by wound closure. It indicates its potential even in burn wounds when contaminated as well. In Addition, patients treatment time as well as overall costs can be successfully reduced. Further studies will be necessary to provide more evidence and show further advantages of Kerecis in comparison to regular wound treatment.

P200

Early Enzymatic Debridement Enhances Survival and Recovery in Elderly Burn Patients: A Retrospective Study

**Dalla Pozza, E.** ^1^, Vigato, E. ^1^, Minic, J. ^1^, Governa, M. ^1^

^1^ A.O.U.I. Verona, Department of Plastic Surgery and Burn Centre, Verona, Italy

Poster presentations 3, 4 September 2025, 15:15–15:45

Aim:

The impact of early enzymatic debridement on survival in elderly burn patients remains underexplored. This study aims to assess the efficacy of early enzymatic debridement in improving outcomes in elderly patients with moderate to severe burns.

Methods:

A retrospective cohort study was conducted on 70 elderly patients (≥65 years) with comparable burn surface areas and second- to third-degree burns, treated at a burn center between 2020 and 2024. Patients were divided into two groups: Group A (*n* = 35) received enzymatic debridement within 72 h of injury, followed by collagenase dressings for 48–72 h and subsequent homograft coverage. Group B (*n* = 35) received conventional treatment without enzymatic debridement due to contraindications or comorbidities. Outcomes included 90-day survival, wound infection rate, need and extent of surgical autografting/burnt TBSA, and time of hospitalization.

Results:

Group A demonstrated higher 90-day survival compared to Group B (95% vs. 86%). Wound infections were less frequent in Group A (24% vs. 42%, *p* < 0.05), and patients required fewer and less extensive surgical interventions. Hospitalization was similar in both groups (32 vs. 35 days).

Conclusions:

Early enzymatic debridement in elderly burn patients is associated with improved survival, fewer infectious complications, and reduced burnt areas requiring surgical intervention. These findings support the integration of enzymatic debridement into early treatment protocols for geriatric burn care. Prospective studies are warranted to validate these results.

P201

Over Intubation During Disasters

**Akgün, A.** ^1^, Akın, M. ^1^

^1^ Türkiye Ministiry of Health, Ankara Bilkent City Hospital, Burn Treatment Center, Ankara, Turkey

Poster presentations 3, 4 September 2025, 15:15–15:45

Aim:

If first responder physicians over react to burn patients during disasters.

Methods:

We briefly reviewed 5 Macedonia Nightclub Fire Disaster patients’ diaries retrospectively at Burn ICU (Intensive Care Unit). Especially about intubation and extubation time.

Results:

Five patients were admitted as intubated 24 h after the Macedonia Nightclub Fire Disaster. These were “young” casualties, 1 female and 4 male, aged between 18 and 27. Two of the patients were extubated after 3 h, the other two after 12 h, and one after 15 h of admission to the Burn Intensive Care Unit. None of them were assessed as having upper respiratory tract thermal injury or inhalation injury. However, due to the lack of a deep history during patient transport in the disasters, they received bronchodilator medication, cold steam, and respiratory physiotherapy as a precaution. None of them were reintubated or had respiratory problems during their hospitalization and were transferred to the ward after 7–10 days, respectively.

Conclusions:

We believe that it is important to further explain thermal injuries and inhalation injuries of the upper respiratory tract to general practitioner candidates in medical schools for both diagnostic and differential diagnosis purposes, in order to prevent unnecessary intubations.

Key words: burn disasters, over intubation, İnhalation injury

P203

Meta-analysis of hydrofluoric acid burns: developing a life-saving protocol

**González-Alaña, I.** ^1^, Martín-Playá, P. ^1^, Rodríguez-Ruiz, P. ^1^, Caramés-Estefanía, J. ^1^

^1^ Cruces University Hospital, Barakaldo, Spain, ^2^ Cruces University Hospital, Barakaldo, Spain, ^3^ Cruces University Hospital, Barakaldo, Spain, ^4^ Cruces University Hospital, Barakaldo, Spain.

Poster presentations 3, 4 September 2025, 15:15–15:45

Aim:

Hydrofluoric acid (HF) burns, though uncommon and primarily work-related, pose a significant risk due to their high lethality. This study reviews existing literature to develop a protocol that reduces mortality related to HF burns.

Method:

HF is widely used in industry and household cleaners. Due to its rapid skin penetration, it causes severe burns and systemic toxicity within minutes. A systematic review of 32 references from PubMed, UpToDate, and other sources was conducted to establish a therapeutic algorithm.

Results:

Of the reviewed literature, 62.5% were case reports, 31.25% systematic reviews, and 6.2% protocols. 85% of patients had burns covering <30% of body surface, 50% were exposed to >20% HF concentration and mortality was 35%. Treatment recommendations included washing with water (53.12%) or Hexafluorine (12.5%) and ocular exposure was treated with saline rinsing (9.37%). Painful burns were managed with 10% calcium gluconate gel (25%) and intravenous infusion of 10% calcium gluconate (25%), although 37.5% preferred intra-arterial infusion. Systemic toxicity risk increased with >50% HF concentration or >5% body surface affected. Urgent debridement (28.12%), ECMO (12.5%), and hemofiltration (43.75%) were proposed for severe cases.

Conclusions:

Prehospital washing with water or Hexafluorine is essential. Severe cases require continuous ECG monitoring, serum calcium control, IV calcium gluconate, and possibly ECMO or hemofiltration. Local treatment includes calcium gluconate gel, subcutaneous infiltration, or intra-arterial infusion. When other treatments prove insufficient, early debridement down to the fascia with delayed coverage should be considered.

P205

Is your burn center prepared for CBRN incidents ?

**Hannmann, T.** ^1,3^, Kreinest, M. ^3^, Frambach, D. ^2^, Kneser, U. ^1^, Palackic, A. ^1^, Hundeshagen, G. ^1^

^1^ BGU Burn Center, Ludwigshafen, Germany, ^2^ BASF Corporate Health Management, Ludwigshafen, Deutschland, ^3^ BGU Disaster Medicine Unit, Ludwigshafen, Deutschland

Poster presentations 3, 4 September 2025, 15:15–15:45

Aim:

To assess the preparedness of burn centers for treating patients with CBRN (Chemical, Biological, Radiological, and Nuclear) injuries and to present a specialized treatment and decontamination approach implemented at BGU Ludwigshafen.

Methods:

- Establishment of a specialized treatment and decontamination team at BGU Ludwigshafen.
- Collaboration with BASF to develop standardized procedures for handling contaminated patients.
- Implementation of safety measures to ensure both effective patient decontamination and staff protection before medical treatment begins.

Results:

- A structured and trained decontamination team and facilities have been established.
- Standardized procedures have been developed to manage contaminated patients safely.
- Effective coordination between emergency teams and burn centers improves response to CBRN injuries.

Conclusions:

Burn centers play a critical role in handling CBRN injuries, but specialized preparation is essential. The approach at BGU Ludwigshafen, in collaboration with BASF, demonstrates an effective model for safe decontamination and treatment, ensuring both patient care and staff protection.

P207

Designing a national burns assessment, management and referral system—A systems engineering approach.

**Hussain, Z.** ^1,2,3,4^, Montoya, M. ^2^, Waleed, T. ^5,6^

^1^ International Health Systems Group, Department of Engineering, University of Cambridge, Cambridge, United Kingdom, ^2^ Whiting School of Engineering, Johns Hopkins University, Baltimore, USA, ^3^ Norfolk and Norwich University Hospitals NHS Foundation Trust, Norwich, United Kingdom, ^4^ AUBMC, American University of Beirut, Beirut, Lebanon, ^5^ Department of Planning, Iraq Ministry of Health, Baghdad, Iraq, ^6^ Iraq Specialized Burns Hospital, Baghdad, Iraq.

Poster presentations 3, 4 September 2025, 15:15–15:45

Aim:

As healthcare needs change dynamically across the globe, little is available to support healthcare leaders, by way of approaches to system design and implementation, in addressing those needs in a timely and effective manner. This work seeks to demonstrate the effective application of systems engineering principles in addressing the need for more coordinated, consistent and effective burns care in Iraq.

Method:

Data provided by the Ministry of Health (MoH) highlighted compromises in the assessment, referral and, access to specialised care of burns patients, resulting in fragmented, inconsistent care pathways and high mortality rates. The systems engineering V model was adopted to address the national burns need. A needs analysis was conducted through structured stakeholder interviews including burns surgeons, patients, MoH officials, the WHO and healthcare auditors. This informed the system’s requirements and functional analysis as well as the system’s concept of operations.

Results:

122 requirements were derived and categorised as 4 mission needs, 50 capability, 14 performance, 53 functional and, 1 constraint requirement. Each requirement was assigned a test and evaluation verification method. 6 key performance parameters were also identified. Requirements were linked to 85 system functions and 39 physical components with 61 interfaces. Relationships were demonstrated using a verification matrix and GeneSys software. 4 clinical scenarios were used to ensure system functionality and a context diagram demonstrated system interactions. 6 major risks were identified and mitigated against in the design phase.

Conclusions:

Systems engineering principles can offer a framework for design and implementation of effective burns services.

P208

Medical Countermeasures Against Nuclear Strike Now Successfully Deployed: Reflections on Next Evolutionary Steps.

**Jeng, J.** ^1^

^1^ University of California Irvine, Orange, United States

Poster presentations 3, 4 September 2025, 15:15–15:45

Aim:

Describe the development and deployment of medical countermeasures against a nuclear strike, and subsequent evolution and refinements planned for the future.

Methods:

Memorializing the two-year work-up from initial call for help, Ukrainian Ministry of Health (MoH); to physical deployment of the kits in-country in the first quarter of 2025.

Results:

As of February 2025, medical countermeasures against nuclear strike have been successfully deployed to Ukraine. The developed concept of operations assumes the most impossibly austere conditions in a post-apocalyptic environment completely devoid of regular order health care. Enough materials have been provided to treat ~7500 major IR flash burns, based on oral resuscitation and dry, flat-packed silver-based burn dressings. Companion countermeasures for Acute Radiation Syndrome (bone marrow) have been provided for ~7500 victims with 2–4 Gray total body exposure, centered on life-saving G-CSF injections and a rapid field method to estimate dosimetry. Direct interface with Ukraine MoH personnel occurred on multiple occasions in Poland and in Ukraine, focused intensely on implementation science challenges. We now turn towards evolutionary development of these v.1 operational concepts and implementation challenges to the needs of neighboring European sovereignties, well aware that every locale will need specific tailoring.

Conclusions:

This government-academia collaboration was achieved under great time pressure and is the first-ever iteration of comprehensive countermeasures against a nuclear detonation. Implementation science is equally important to the original development of concept. Evolutionary refinements come naturally with greater experience and differing needs of other host nations.

P210

The stock of human allogeneic skin grafts in a view of a mass disaster

**Łabuś, W.** ^1^, Słaboń, A. ^1^, Ziółkowska, K. ^1^, Czerny, K. ^1^

^1^ Stanislaw Sakiel Burn Treatment Center In Siemianowice Slaskie, Siemianowice Śląskie, Poland

Poster presentations 3, 4 September 2025, 15:15–15:45

Aim:

To achieve and manage the stock of human allogeneic skin grafts in case of a mass disaster that may involve large numbers of severely burned patients.

Methods:

This study involved an analysis of selected mass disasters. From this an estimate was made from a verified casualty profile of the necessary minimum stock of human allogeneic skin graft materials. A proposal has been made for the organizational, legal and systemic changes required to improve the situation in Polish transplantology, with particular emphasis on skin donation. In order to achieve and sustain strategic reserves we implemented industry management methods, such as kanban and DBM (dynamic buffer management).

Results:

A government program has been established to create a strategic bank of biological dressings. In order to achieve a strategic stock of human skin grafts, a tissue collecting transplantation team was organized. The rights and obligations of the non-physician transplant team member should be extended. Proposals have been made for awareness campaigns (adverts, posters etc.) and educational schemes (educational video, lectures during transplant coordinator training, etc.). Finally, a proposal has been made for possible methods to deal with the logistic management of the allogeneic skin stock, especially constraint management methods.

Conclusions:

The required, essential stock of human allogeneic skin in the event of a mass disaster has been estimated at 600,000 cm^2^.

P211

From constraint-management to result-base outcome treatment—how we achieved major breakthrough thanks to industry-level management methods in public hospital

**Ludyga, M.** ^1^, Nowak, M. ^1^, Ambroziak-Łabuś, J. ^1^

^1^ Stanislaw Sakiel Burn Treatment Center, Siemianowice Slaskie, Poland

Poster presentations 3, 4 September 2025, 15:15–15:45

Aim:

The delivery of highly specialized care—especially in wound management—remains undervalued and fragmented. Despite clinical advances, patients often face long delays between appointments, leading to poorer outcomes, prolonged hospital stays, and reduced system-wide capacity.

We identified a key constraint in patient flow and care delivery. Without disciplined, time-sensitive management, clinical results plateaued. To break this limitation, a strategic shift was needed—from reactive care to results-based treatment planning. This meant embedding proven, industry-level management methods into hospital operations to drive clinical and organizational breakthroughs.

Methods:

Using sophisticated industry-level management methods, we redesigned the treatment process around measurable outcomes.

The care pathway was reengineered to improve flow efficiency, reduce waiting time, and eliminate bottlenecks. Steps included system constraint identification, creation of a focused improvement plan, lead-time monitoring, and outcome validation. The validated model was then scaled across other departments through structured dissemination and training, establishing a broader, systemic approach.

Results:

The initiative became a nation-wide, legislated program for complex wound care—a key milestone in expanding access and improving outcomes.

In one year, more patients were treated within the same infrastructure, without additional bed capacity. The ratio of fully healed wounds (100%) to partially healed wounds (≥60%) reached 4:1.

Conclusions:

This program demonstrates how constraint-based and outcome-focused management can transform healthcare. Hospitals must adopt systems thinking, shifting from siloed models to industrial-level operational excellence—focused on flow, capacity, and measurable clinical results.

P212

Kyiv burn centre: our experiences as uk-based burn surgeon and burn nurse working with the red cross.

**Macdonald, M.** ^1^, Jeffery, S. ^1^

^1^ Red Cross, Liverpool, United Kingdom

Poster presentations 3, 4 September 2025, 15:15–15:45

Aim:

To provide a comprehensive insight into the experiences of a UK-based burn surgeon and burn nurse working with the British Red Cross (BRC) and International Committee of the Red Cross (ICRC) to enhance care in Kyiv burn centre.

Methods:

The observational study was conducted for six weeks in Kyiv City Hospital 2. Staff were observed in areas like the operating theatre, dressing room and intensive care unit.

Results:

The ongoing conflict has led to a rise in burn injuries associated with antimicrobial resistance, largely due to prophylactic broad-spectrum antibiotic use. Direct betadine soaks are the dressing applied to all burns in the unit, requiring renewal every 24–48 h. Most burns are infected by multidrug-resistant pseudomonas and/or klebsiella. The lack of modern antimicrobial dressings hampers early burn excision. The effective honey or silver/copper dressings were advised as alternatives. Moreover, two patients were in the operating theatre simultaneously. It is essential to recommend and provide education on infection prevention and control measures. Additionally, staff were unaware of the importance of monitoring a patient’s body temperature. Dietician support is advised to address the lack of nutritional knowledge and ensure appropriate use of nasogastric tubes for patients with ≥30–40% burns.

Conclusions:

It was evident the importance of providing education and resources to address the gaps in care, ultimately improving burn treatment for patients. The team is to return in April 2025 to provide education on the identified topics and train staff on the newly acquired equipment.

P213

Artificial intelligence in burn care: defining the gaps and barriers

**Moortgat, P.** ^1,2^, Demarbaix, T. ^1,3^, Meirte, J. ^1,3^, Anthonissen, M. ^1,3,4^, Van Daele, U. ^1,3^, Maertens, K. ^1,5^

^1^ Oscare, Organisation for Burns, Scar Aftercare and Research, Antwerp, Belgium, Antwerp, Belgium, ^2^ BridgewAI, Antwerp, Belgium, ^3^ Department of Rehabilitation Sciences and Physiotherapy REVAKI-MOVANT, Faculty of Medicine and Health Sciences, University of Antwerp, Wilrijk, Belgium, ^4^ Department of Rehabilitation Sciences, KU Leuven, Leuven, Belgium, ^5^ Department of Clinical and Lifespan Psychology, Vrije Universiteit Brussel, Brussels, Belgium.

Poster presentations 3, 4 September 2025, 15:15–15:45

Aim:

Artificial intelligence (AI) is transforming burn care through applications in diagnostics, imaging, patient-monitoring, and predictive modeling. Despite significant advancements, challenges remain in optimizing AI’s effectiveness and expanding its role beyond acute care. This review aims to evaluate the accuracy of AI-models, identify barriers to optimal performance, and highlight underexplored domains in AI-driven burn care.

Methods:

A narrative literature review was conducted on AI-applications in burn care, focusing on accuracy, implementation challenges, and research gaps. Studies were analyzed to assess model performance, common barriers, and areas where AI remains underutilized.

Results:

The literature search resulted in 54 retrieved papers of which only 8 mentioned accuracy figures. AI-applications in burn care have demonstrated high accuracy, with reported figures ranging from 76% to 96% for mortality prediction, 88% for burn depth classification, and 80% for image classification. However, several barriers hinder AI’s optimal performance, including data quality limitations, lack of external validation, high computational demands, ethical concerns, and clinician skepticism. Underexplored areas include virtual simulation research for training, robotics in burn rehabilitation, AI-driven psychological aftercare, and AI for burn prevention strategies. Additionally, long-term scar monitoring remains insufficiently studied.

Conclusions:

AI has the potential to enhance acute burn care, rehabilitation, and long-term patient management, but significant challenges persist. Addressing data-limitations, improving clinical integration, expanding research into underexplored domains, and fostering clinician-trust are critical for maximizing AI’s impact in burn care. Future research should prioritize multi-center validation and seamless clinical integration to foster trust, facilitate adoption, and maximize AI’s impact in burn care.

P214

Impact of burn surgery on hospital theatre time and resources: a retrospective study of the relationship between burn characteristics and number and duration of surgical procedures.

**Petsa, A.** ^1^, Nikolaidou, E. ^1^, Ligomenou, T. ^1^, Michopoulos, A. ^1^, Makarona, K. ^1^, Papadopoulou, S. ^1^

^1^ Department of Plastic and Hand Surgery & Burns ICU, “G. Papanikolaou” Hospital, Thessaloniki, Greece.

Poster presentations 3, 4 September 2025, 15:15–15:45

Aim:

We studied operative time needed for burn patients and its relation to factors such as burn surface area and depth and specific areas being treated. Further aim was to predict the theatre time per patient for better planning and optimization of resources allocation.

Methods:

Operative records and registry data of burn patients hospitalized in our Burn Center between January 2023 and December 2024 were retrospectively analyzed. Data fields included age, gender, TBSA and localization of burn, number of procedures and their duration, number of surgeons involved, time of surgery (working time or on-calls). 85 patients were included in the study, 62 male and 23 female with average age 56 ± 14 (range 16–88) years.

Results:

64 of the patients had at least one surgical procedure (75%). Their average Total Burn Surface Area (TBSA) was 25.5 ± 19% and Abbreviated Burn Severity Score (ABSI) 7.5 ± 2.2 with 49 survivors and 15 non-survivors. A total of 177 operations were carried out (2.8 ± 1.5 per patient, range 1–10) with a total 319 h of allocated theatre time. A great percentage of surgeries were performed out of regular working hours and 2–6 surgeons were participating. The mean hospital stay was 35 ± 24 days.

Conclusions:

Access to theater is essential for the management of deep burns. Availability is not always given, as personnel shortages often occur. Burn Unit patients bring a huge workload and surgical burden in the Plastic Surgery Department and theatre. Resources are needed in order to achieve better outcomes, shorter hospital stays and to avoid associated complications.

P215

Ten-year experience (2014–2023) of burn treatment in Poland and eastern-located burn centre—retrospective analysis, COVID-19 pandemic (COVID-19) impact and lessons for the future.

**Winiarska, A.** ^1^, Korzeniowski, T. ^2^, Surowiecka, A. ^3^, Piszczek, J. ^1^, Tobiasz, M. ^1^, Opaliński, H. ^1^, Barbachowska, A. ^1^, Strużyna, J. ^2^

^1^ East Centre of Burns Treatment and Reconstructive Surgery, Independent Public District Hospital in Leczna, Poland, ^2^ East Centre of Burns Treatment and Reconstructive Surgery, Independent Public District Hospital in Leczna, Poland; Department of Plastic Surgery, Reconstructive Surgery and Burn Treatment, Medical University of Lublin, Lublin, Poland ^3^ East Centre of Burns Treatment and Reconstructive Surgery, Independent Public District Hospital in Leczna, Poland; Department of Plastic, Reconstructive Surgery and Microsurgery, Medical University of Lublin, Lublin, Poland.

Poster presentations 3, 4 September 2025, 15:15–15:45

Aim:

Burns are an important public health problem worldwide. The majority of injuries occur in developing countries; the number is decreasing in higher-income countries. Analysis of epidemiological data allows the development of burn prevention systems and effective healthcare organisation. We aimed to analyse data on burn care in our centre (the only burn centre in the macroregion) and to describe the profile of burn injury and care, including during COVID-19.

Methods:

Data were collected on patients admitted to the East Burns Centre and Reconstructive Surgery between 2014 and 2023. A retrospective analysis was performed using Statistica software.

Results:

Nearly 2150 hospital admissions were documented, with an average annual caseload of 215 patients. More than 70% were male. The median length of hospital stay was 16 days (longer during COVID-19: 19 days [*p* = 0.0004]). The mean burn area was 33% of the total body surface area (TBSA), median TBSA 8%, mode TBSA 5%. 2.79% of the hospitalisations were fatal. 53.37% of patients required surgical treatment, e.g., split-thickness skin graft or enzymatic debridement. Injuries were more severe in COVID-19 (75.4% vs 68.1% of full-thickness burns, *p* = 0.0005).

Conclusions:

The number of burns requiring hospitalisation is decreasing, both worldwide and in Poland. Therefore, it is worth considering how to focus burn care in specialised centres. Treatment of burns requires specialised knowledge and unique skills. The right clinical decisions must be made at the right time (especially in challenging times, such as a pandemic) to prevent death and long-term sequelae.

P216

Historical Mass Disasters in Silesia Poland Since 1994: The Role of Methane Explosions in Coal Mines

**Ziółkowska, K. **^1^, Słaboń, A. ^1^

^1^ Stanislaw Sakiel Burn Treatment Center in Siemianowice Slaskie, Siemianowice Śląskie, Poland

Poster presentations 3, 4 September 2025, 15:15–15:45

Aim:

This study analyzes the epidemiology, causes, and medical responses associated with methane explosions in Polish coal mines. By assessing past incidents and their impact on healthcare, the study underscores the importance of continuous medical innovation and emergency preparedness in mitigating mass disaster effects.

Methods:

A retrospective analysis was conducted on major methane explosions in Polish coal mines, e.g Halemba (2006), Wujek-Śląsk (2009), and Pniówek (2022). Data sources comprised official reports, medical records, and academic literature. The study examines injury patterns, emergency responses, and advancements in disaster medicine.

Results:

The most recurrent and devastating mass disasters in Poland involve methane explosions in coal mines, predominantly in the Silesian Voivodeship. Methane explosions in coal mines are among the deadliest industrial accidents, characterized by high mortality rates and severe injuries. These incidents cause immediate casualties, extensive burns, and long-term health complications. Over the past three decades, medical responses have evolved, improving pre-hospital care, burn treatment, and interdisciplinary cooperation between emergency services and specialized burn centers.

Conclusions:

Lessons from these disasters have driven advancements in mining safety protocols and disaster preparedness. The findings emphasize the need for continuous improvements in industrial safety regulations and emergency medical infrastructure to maximize survival rates, continuous investment is needed—systemic application of process optimization industry-level management methods that ensure preparedness, coordination, and resilience during mass casualty events.

P217

Versatile applications of negative pressure wound therapy in pediatric burns

**Abay, A.** ^1^, Çayhan, V. ^1^, Öztorun, C. ^2^, Demir, S. ^1^, Şenel, E. ^2^, Azılı, M. ^2^

^1^ Ankara Bilkent City Hospital, Ankara, Türkiye, ^2^ Ankara Yildirim Beyazit University, Ankara, Türkiye.

Poster presentations 3, 4 September 2025, 15:15–15:45

Aim:

Negative Pressure Wound Therapy (NPWT) was initially developed for chronic and traumatic wounds, helping reduce edema, lower infection risk, and promote granulation tissue formation. Recently, its potential benefits in burns have gained attention. This study shares our experience with NPWT use in pediatric burns for various indications.

Methods:

We retrospectively analyzed the patients treated with NPWT in our pediatric burn center over the past five years, evaluating clinical data and treatment outcomes.

Results:

A total of 41 NPWT applications were performed in 32 patients. Based on NPWT indication, patients were classified into two groups: burns in difficult-to-manage areas (e.g., neck, axilla, ankle) and deep burns. NPWT was used in 11 cases in these challenging areas (63% in the axilla) and after contracture release (11.7 ± 5.6 days, 3 cases) or over grafting (4.75 ± 1.3 days, 8 cases). In five of the 30 deep burn cases, NPWT followed facial excision for invasive infection. Among 25 patients with 4th-degree burns, 68% were due to electrical injuries. In this group NPWT was applied after burr hole procedures (5 cases), amputations (7 cases), or fasciotomies (2 cases). Treatment was discontinued upon granulation tissue formation, with closure via autologous split-thickness grafts. No NPWT-related complications were observed.

Conclusions:

NPWT is a safe and effective treatment for pediatric burns, particularly in deep and high-risk areas. Its use may enhance healing and reduce complications, supporting its role as a valuable adjunct in burn management.

P218

Viral infections in burn wounds

**Abay, A.** ^1^, Şenel, E. ^2^, Demir, S. ^1^

^1^ Ankara Bilkent City Hospital, Ankara, Türkiye, ^2^ Ankara Yildirim Beyazit University, Ankara, Türkiye.

Poster presentations 3, 4 September 2025, 15:15–15:45

Aim:

Although bacterial infections are most common in burn patients, viral infections can also occur. This study aims to share our center’s experience with Herpes Simplex Virus (HSV) and Human Papilloma Virus (HPV) infections in pediatric burn patients.

Methods:

We retrospectively reviewed the files of patients treated at our burn center who developed viral infections in burn wounds. Demographic and clinical data were analyzed.

Results:

Viral infections developed in the burn areas of six patients treated at our burn center. In four male patients aged 1.5–2 years, vesicular rashes appeared on days 7–9 of treatment, limited to newly epithelialized burn sites. Diagnosis was made clinically in collaboration with dermatology and infectious diseases. One patient had positive HSV-1 serology; another showed viral cytopathic changes (Tzanck cells) on punch biopsy, supporting the diagnosis. All received topical and systemic antiviral therapy, resulting in resolution of lesions.

The remaining two patients presented with verrucous lesions over healed burns at 3 months and 3 years post-injury. In one, biopsy confirmed verruca vulgaris. Both were treated with cryotherapy and a topical ointment containing urea and salicylic acid.

Conclusions:

Viral infections, though less common, should be considered during both early and late burn recovery. Lesions confined to burn areas may delay diagnosis due to atypical presentation. HPV-related lesions can appear long after initial healing and may require prolonged follow-up due to their association with chronic inflammation and potential malignancy. Clinical findings, supported by serology and histopathology when needed, are essential for timely diagnosis and management.

P219

Non-burn cases in a pediatric burn center: Stevens-Johnson syndrome and toxic epidermal necrolysis.

**Abay, A.** ^1^, Demir, S. ^1^, Azılı, M. ^2^, Şenel, E. ^2^

^1^ Ankara Bilkent City Hospital, Ankara, Türkiye, ^2^ Ankara Yildirim Beyazit University, Ankara, Türkiye

Poster presentations 3, 4 September 2025, 15:15–15:45

Aim:

Stevens-Johnson Syndrome (SJS) and Toxic Epidermal Necrolysis (TEN) are rare, drug-induced dermatological emergencies in children. Despite their non-burn origin, they require intensive wound and critical care which make them relevant cases for pediatric burn teams.

Methods:

This retrospective study evaluated pediatric patients treated for SJS/TEN over six years. Two were excluded due to biopsy findings of erythema multiforme. Patients were grouped based on skin involvement: SJS (<10%), TEN (>30%), and overlap (10–30%). Demographics, clinical features, treatment, and outcomes were analyzed.

Results:

Eleven patients were included: 2 with SJS, 8 with TEN, and 1 with overlap. The average age was 12.2 years; 63.6% were female. Medication history included antibiotics (7 patients), anti-inflammatories (3 patients), and antiepileptics (2 patients). All had oral mucosal involvement; genital and ocular mucosa were affected in 72.7% and 18.1% of cases, respectively. The mean affected body surface area was 55.5%. Wound care involved nanocrystalline silver dressings (3 patients) or paraffin gauze (8 patients). All received IV corticosteroids and immunoglobulin; additionally, 7 received cyclosporine, and 2 were treated with hydrocortisone. Despite presenting early, SJS/TEN-specific treatment began after an average delay of 4.5 days. Two required mechanical ventilation, and one patient (90% involvement) died.

Conclusions:

SJS/TEN often begin with non-specific symptoms, leading to delayed diagnosis. The extent of skin and mucosal damage significantly impacts prognosis. Pediatric burn teams play a crucial role in wound management and coordination of multidisciplinary care (especially with pediatric intensive care units) is crucial for improving outcomes.

P220

Enzymatic Debridement in the Management of Burns in the Paediatric Population: A Systematic Review.

**Abdul, A.** ^1^, Pastor González, M. ^2^, Herrera Renaud, L. ^3^, Frew, Q. ^4^, Fernandez-Diaz, O. ^5^

^1^ King’s College London, London, United Kingdom, ^2^ Universidad Europea de Madrid, Madrid, Spain, ^3^ University of Barcelona Campus Hospital Clínic, Barcelona, Spain, ^4^ St Andrew’s Centre for Plastic Surgery and Burns, Broomfield Hospital, Chelmsford, United Kingdom, ^5^ Department of Clinical Sciences, CUTLAJOMULCO, University of Guadalajara, Guadalajara, Mexico.

Poster presentations 3, 4 September 2025, 15:15–15:45

Aim:

Burn debridement is vital in paediatric plastic surgery to mitigate infection risks and foster healthy tissue regeneration. While traditional methods like surgical excision are effective, they often remove healthy tissue, a significant concern for paediatric patients with smaller body surface areas. Enzymatic debridement offers a minimally invasive alternative, selectively targeting necrotic tissue while preserving healthy tissue. This systematic review evaluates the efficacy and safety of enzymatic debridement in managing paediatric burns.

Methods:

A systematic review was conducted per PRISMA guidelines using PubMed, Scopus, Medline, and Embase databases. Studies in English or Spanish involving enzymatic debridement in children (<18 years) were included. Outcomes assessed included infection rates, bleeding, length of hospital stay, scar formation, and complications.

Results:

From 58 studies, 8 involving 555 paediatric patients met inclusion criteria. Agents such as Nexobrid (bromelain-based) and collagenase were evaluated. Most burns were thermal (fire 46%, scald 43%). Nexobrid demonstrated significantly shorter debridement times (1.9 ± 0.8 days vs. 8.1 ± 6.3 days, *p* < 0.001) and reduced hospital stays (29.9 ± 14.3 days vs. 32.1 ± 18.9 days). Infection rates were 17.7%, with minimal bleeding. Enzymatic debridement required fewer transfusions (2% vs. 73%, *p* < 0.01) and less frequent skin grafting (2% vs. 80.5%).

Conclusions:

Enzymatic debridement, particularly Nexobrid, is a safe, effective alternative to surgical excision for paediatric burns, reducing debridement time, transfusion needs, and skin grafting. Future multicentre studies should explore long-term outcomes.

P221

Questionnaires assessing activity limitations and participation restrictions in children post-burn: a systematic review.

**Anthonissen, M.** ^1,2^, Meirte, J. ^1,2^, Moortgat, P. ^1^, Maertens, K. ^1,3^

^1^ Oscare, Organisation for Burns, Scar After-Care and Research, Antwerp, Belgium, ^2^ University of Antwerp, Rehabilitation Sciences and Physiotherapy, MOVANT, Antwerp, Belgium, ^3^ Vrije Universiteit Brussel, Department of Clinical and Lifespan Psychology, Brussels, Belgium.

Poster presentations 3, 4 September 2025, 15:15–15:45

Aim:

To identify and evaluate questionnaires that assess ICF domains activities of daily living and participation in society in children post-burn, considering both child’s and parents’ perspectives.

Methods:

A systematic literature review was conducted in Pubmed and Web of Science. Studies examining activity limitations or participation restrictions in burn patients aged 0–18 years were included.

Results:

We included 23 studies describing 19 outcome measures to evaluate activity limitations or participation restrictions or both: 6 (burn) specific and 13 generic questionnaires. The Burn Outcome Questionnaire (BOQ) was most often applied (*n* = 8). For the generic questionnaires, the Pediatric Quality of Life inventory (PedsQL) was the most widely used (*n* = 3). From these 19 outcome measures, the majority (*n* = 15) assessed both activities of daily living and societal participation. Three questionnaires solely investigated participation: Satisfaction with Life Scale (SWLS), Community Integration Questionnaire (CIQ) and Youth Quality of Life Instrument (YQOL). One questionnaire focused on activities: Functional Independence Measure (FIM).

Twelve questionnaires were child-reported, seven were parent/proxy-reported, and one was clinician-reported (FIM). Some studies compared questionnaire responses provided by parents with those directly reported by children.

Due to variations in study design, study population and type of outcome measures, pooling data was difficult.

Conclusions:

Multiple questionnaires are at hand to evaluate activities of daily living and participation. There seems to be a difference in patient self-reported and parent-reported data. Although various studies included child-reported questionnaires there seems to be no unique/universal questionnaire and clinimetric properties need to be better explored.

P222

Smaller Surface, Deeper Damage: Surgical and Clinical Implications of Pediatric Hand Burns

**Bartkova, J.** ^1,2^, Szabo ^3^, Miklisova, D. ^4^, Kanuscak, K. ^5^, Jarosova, K. ^6^, Hrizova, S. ^7^, Buganova, B. ^8^, Cederna, P. ^9^

^1^ Department of Burns and Plastic Surgery, The University Hospital Brno, Czech Republic, Brno, Czech Republic, ^2^ Faculty of Medicine, Masaryk University Brno, Czech Republic, Brno, Czech Republic, ^3^ Institute of Medical Physics and Biophysics, Faculty of Medicine, Comenius University, Bratislava, Slovakia, Bratislava, Slovakia, ^4^ Department of Traumatology, Faculty of Medicine, Masaryk University, Brno, Czech Republic, ^5^ 2nd Department of Surgery, Motol University Hospital, Prague, Czech Republic., Prague, Czech Republic, ^6^ St. Anne’s University Hospital Brno, Department of Urology, Brno, Czech Republic, Brno, Czech Republic, ^7^ Faculty of Medicine, Comenius University Bratislava, Slovak Republic, Bratislava, Slovak Republic, ^8^ Faculty of Medicine of the Slovak Medical University, Bratislava, Slovak Republic, Bratislava, Slovak Republic, ^9^ Department of Surgery, Section of Plastic Surgery, University of Michigan Health System, Ann Arbor, Michigan, Michigan, USA.

Poster presentations 3, 4 September 2025, 15:15–15:45

Aim: 

This study aimed to describe the epidemiological and clinical profile of pediatric hand burn patients, focusing on age patterns, setting, treatment, and outcomes.

Methods:

A retrospective cohort study was conducted on pediatric hand burn patients admitted to a tertiary burn center between 2005 and 2022.

Results: 

Of 2601 pediatric burn patients, 584 (22.5%) sustained hand burns. Younger children (≤5 years) had a lower prevalence (*p* = 0.003), with a slight age-related increase (rpb = 0.08, *p* < 0.001). Most injuries (82%) occurred at home (*p* < 0.001). Although associated with a lower average TBSA (5.3% vs. 6.5%, *p* < 0.001), hand burns showed a higher incidence of deep (grade III) burns (1 in 3.6 vs. 1 in 5.6, *p* < 0.001). Affected children required twice as many surgeries on average (1.52 vs. 0.84; *p* < 0.001) and were more likely to undergo surgical treatment (*p* = 0.006). No association was found with the need for acute surgery on admission (*p* = 0.8). Chemical debridement was used significantly more than surgical (96% vs. 37%; *p* < 0.001). Hospital stays were approximately 30% longer in the hand burn group (9.8 ± 1.9 days vs. 6 ± 0.33 days; *p* = 0.05). There was no significant association with ICU admission or infection (*p* = 0.263; *p* = 0.357).

Conclusions: 

Pediatric hand burns are predominantly domestic, and associated with deeper injuries, more surgeries, and longer hospitalizations. These findings highlight the importance of preventive measures at home and the need for individualized surgical care in pediatric hand burn management.

P224

Paediatric Burns Admissions at Mater Dei Hospital: A Comprehensive Audit

**Bugeja, K.** ^1^, Grima, T. ^1^, Nasser, Z. ^1^, Borg, D. ^1^, Aquilina, D. ^1^

^1^ Mater Dei Hospital, Malta, Msida, Malta

Poster presentations 3, 4 September 2025, 15:15–15:45

Aim:

Paediatric burns pose significant healthcare challenges due to complex treatment needs and long-term complications. This retrospective audit examines five years of paediatric burn admissions at Mater Dei Hospital, aiming to enhance the quality of care provided.

Methods:

The study included patients under 16 years of age who were admitted between March 2019 and March 2024. Collected data covered patient demographics, nationality, administration of fluid resuscitation, burn type, hospital stay duration, requirement for surgical procedures, infections, and use of antibiotic treatment.

Results:

The study involved 62 paediatric patients. The study included a relatively even gender distribution (54.8% male, 45.2% female) and patients’ ages ranged from 2 months to 13 years, with an average age of 3.35 years, indicating a predominance of very young children.

The majority of patients were Maltese (64.5%) and Syrian (12.9%), with smaller groups from other countries, whereas 58.1% were from Europe, 17.7% from Asia, and 6.5% from Africa, reflecting the diverse international population at Mater Dei Hospital.

Thermal burns were the most common (94.3%), followed by chemical (3.8%) and electrical burns (1.9%), with 55.6% of patients having second-degree burns; fluid resuscitation was required in 24.2% of cases. Hospital stays averaged 8.51 days, with a maximum of 30 days, and 14.5% required surgery. Infection rates were low (11.3%), and 12.9% received antibiotics.

Conclusions:

The findings offer valuable insights into the management of such patients, emphasizing key factors like burn types, treatment approaches, and outcomes, which will help guide improvements in care and management for this vulnerable population.

P225

Successful Non-Surgical Treatment of a Pediatric Scald Burn with Enzyme Alginogel

Yalaniz, B. ^1^, Alkan, I. ^1^, **Cennet, O.** ^1^, Konan, A. ^1^

^1^ Hacettepe University, Dept of Surgery, Burn Unit, Ankara, Turkey

Poster presentations 3, 4 September 2025, 15:15–15:45

Aim:

To report a pediatric scald burn case successfully treated with enzyme alginogel, emphasizing its non-surgical use and pain relief benefits.

Methods:

A 14-year-old male patient presented with a partial-thickness scald burn (9% TBSA) on the right leg after hot water kettle spillage. Conservative treatment with Flaminal Hydro, a hydrated alginate gel embedded with glucose oxidase, lactoperoxidase, and guaiacol, was initiated. No surgical intervention was required. Informed consent were obtained.

Results:

Wound healing was achieved without grafting, and the patient had a full functional recovery with minimal scarring (Picture 1). Pain during dressing changes was notably low, allowing outpatient management and improved compliance. Literature supports that enzyme alginogel provides a moist wound environment with autolytic debridement and intrinsic antimicrobial activity, which contributes to decreased dressing frequency and significantly reduced pain scores compared to traditional silver-based dressings

Conclusions:

Enzyme alginogel enabled a safe, effective, and pain-minimizing non-surgical approach for a pediatric partial-thickness burn. Its favorable pain profile, alongside antimicrobial and healing-promoting effects, makes it a valuable option in pediatric burn management. This case aligns with the FLAM study, which reported reduced dressing pain and fewer dressing changes in patients treated with enzyme alginogel.

P226

Tiny Patients, Big Impact: Nexobrid takeson Pediatric Burns

**Citterio, A.** ^1^

^1^ Niguarda Hospital, Milan, Italy

Poster presentations 3, 4 September 2025, 15:15–15:45

Aim:

Child’s burns has a major impact on the young patient and the family. It is important to limit, as much as possible, the hospitalization and trauma resulting from the burn treatment.

Methods:

We evaluated the length of hospital stay, the number of procedures, the pain, the need for drugs in a group of 12 children from 1 to 12 years treated with enzymatic escarolysis.

Results:

Comparing the results with a sample of 12 children treated in the previous year, we verified a similar number of grafts, less transusions and hospitalization days.

Conclusions:

The best result was the improved impact on the early treatment and the reduction of medications. The reduction of the hospital stay was associated with better satisfaction of the patients and family. Is also in evalutation the quality of scars compared to the standard of care. The limit of the comparison is due to the different age of the children in the two groups. In the two groups the age range is from 1 to 15 years.

P227

Recalling a traumatic event: The use of event freeze-framing and guided questioning after a burn injury in a child enhances data harvesting

Dalan, R. ^1^, **Shah, M.** ^1^

^1^ Royal Manchester Children’s Hospital, UK, Manchester, United Kingdom

Poster presentations 3, 4 September 2025, 15:15–15:45

Aim:

We describe the use of event freeze-framing and guided questioning to obtain a detailed history surrounding a burn injury in children.

Methodology:

When taking the details of the burn injury from the parent/carer, the doctor/nurse is advised to freeze frame the moment of the injury to the child. The parent is asked to “visualise” the actual moment and the clinician asks guided questions to understand the mechanism of the injury and the surrounding circumstances as it happened. This will be demonstrated in the descriptive presentation.

Results:

This technique has aided clinicians to obtain details about the injury which better explain whether it is feasible/accidental/deliberate. It has also helped identify risks and safety measures that can be advised to parents/carers to help prevent further injuries.

Conclusions:

A detailed, data-rich history forms the backbone of the management of burn injuries and is especially crucial in informing the safeguarding status within a paediatric setting. This technique empowers the parent/carer to provide details which they may not have felt to be of relevance for the clinician. A rethinking of how information is gathered in a paediatric burn history is proposed. This allows for a smoother flow of information amongst multidisciplinary agencies in an often fraught and complex paediatric burns safeguarding process.

Applicability to Clinical Practice:

Currently taught and used as part of the paediatric burns induction for new clinicians at Royal Manchester Children’s Hospital.

P228

Reconstructive surgery on children and adolescents with burn sequelae in an outpatient rehabilitation center in Santiago-Chile: 6 years’ experience

**Espinosa, M.** ^1^, Quezada, M. ^1^, Díaz, J. ^2^

^1^ Surgery Team, Corporación De Ayuda Al Niño Quemado. COANIQUEM, Santiago, Chile, ^2^ Dirección de Extensión, Docencia e Investigación, DEDI. Corporación de Ayuda al Niño Quemado, COANIQUEM., Santiago, Chile.

Poster presentations 3, 4 September 2025, 15:15–15:45

Aim:

Reconstructive surgery is relevant for the treatment of burn sequelae after burns, but the evidence describing it as an outpatient procedure is limited. This study aims to describe the demographic and clinical profile of patients receiving reconstructive surgery in an outpatient burn center.

Methodology:

An observational retrospective study was conducted. Demographic, clinical, and surgical data of patients who underwent reconstructive surgeries between January 2011 and December 2016 were analyzed using descriptive statistics. The study has ethical approval from COANIQUEM.

Results:

During the study period, 1109 surgeries were performed on 882 patients under 20 years of age. The mean age at the time of the injury was 2.3 years, with scalds as the predominant cause of burns (53.2%). Burn extension was less than 6% TBSA in 50% of the sample. The most frequently injured location was the upper extremities (46.8%). The mean time from injury to the first reconstructive surgery was 1.3 years, with the hand being the most frequently reconstructed anatomical site (38.9%). Scar release plus flaps was the most commonly used surgical technique (42.7%). Complications requiring reintervention occurred in 7.5% of surgeries performed.

Conclusions:

The study described the patient profile and techniques used in reconstructive surgeries in children and youth with burn sequelae. Consistent with published evidence, most surgeries are performed on the burned hand. The complication rate is similar to that observed in surgeries performed on hospitalized patients. Future studies are warranted to identify factors that predict the need for reconstructive surgery after burns in children.

P229

Application Options for SpinCare© in Children—An Alternative to Challenging Dressing Procedures?

**Flach, A.** ^1^, Hartmann, B. ^2^, Noatnick, M. ^1^

^1^ Pediatric Burn Center, Department of Pediatric Surgery, Sana Klinikum Lichtenberg, Berlin, Germany, ^2^ Center for Severe Burn Injuries, Department of Plastic Surgery, Unfallkrankenhaus, Berlin, Germany.

Poster presentations 3, 4 September 2025, 15:15–15:45

Aim:

The use of EHF-based technologies such as SpinCare© (Lod, Israel) presents a promising alternative to conventional dressings, particularly for burn injuries located in difficult-to-treat areas such as the face, head, and genital region in children.

Methods:

This study is a retrospective in-house evaluation of SpinCare© application in 73 pediatric patients (average age: 14 months) treated within the past 12 months. We defined “difficult-to-treat areas” as the head, face, genitals, and buttocks—regions where standard dressing applications pose specific challenges in terms of hygiene, infection risk, and patient (or parental) compliance.

Results:

For second-degree burn wounds, SpinCare© proved to be a practical and significantly better-tolerated alternative to traditional dressings. This was especially evident in burns located on the head and face, where SpinCare© led to reduced pain and itching, while also supporting favorable cosmetic outcomes.

Conclusions:

Very young children (particularly under 18 months of age) benefit greatly from SpinCare© treatment, as it supports a child-friendly daily routine—including activities such as breastfeeding and bottle feeding. Our evaluation demonstrates that SpinCare© is an effective and well-tolerated alternative for treating burns in anatomically sensitive or hard-to-dress areas.

P230

Development of the pediatric patient scale of the Patient and Observer Scar Assessment Scale (POSAS) for burn scars: an international qualitative study.

**Kemme, F.** ^1,2,3^, Stoop, M. ^1^, Parry, I. ^4^, Meij-de Vries, A. ^1,5,6^, Palmieri, T. ^4,7^, van Zuijlen, P. ^1,2,3,5,8^, Pijpe, A. ^1,2,3^

^1^ Alliance of Dutch Burn Care, Burn Center, Red Cross Hospital, Beverwijk, The Netherlands, ^2^ Amsterdam UMC location Vrije Universiteit Amsterdam, Department of Plastic, Reconstructive, and Hand Surgery, Amsterdam, The Netherlands, ^3^ Amsterdam Movement Sciences, Tissue Function and Regeneration, Amsterdam, The Netherlands, ^4^ Neil Reitman Pediatric Burn Institute at Shriners Children’s Northern California, Sacramento, United States of America, ^5^ Amsterdam UMC location University of Amsterdam, Pediatric Surgical Center, Emma Children’s Hospital, Amsterdam, The Netherlands, ^6^ Department of Surgery, Red Cross Hospital, Beverwijk, The Netherlands, ^7^ Department of Surgery, University of California, Davis, Regional Burn Center, Davis, United States of America, ^8^ Department of Plastic Reconstructive and Hand Surgery, Red Cross Hospital, Beverwijk, The Netherlands.

Poster presentations 3, 4 September 2025, 15:15–15:45

Aim:

To develop the pediatric patient scale of the Patient and Observer Scar Assessment Scale (POSAS) for assessing burn wound scars in children.

Methods:

In this international qualitative study, semi-structured interviews with children with burn wound scars and focus group sessions with parents were conducted in The Netherlands (Burn Centre, Red Cross Hospital, Beverwijk) and the United States (Neil Reitman Pediatric Burn Institute, Shriners Children’s Northern California). We aimed to identify key aspects of scar quality for children, as well as the most suitable response options. Interviews and focus groups were audio-recorded, transcribed, and thematically analyzed using MAXQDA. The relevant themes were formulated into items for a draft patient scale which was pretested with children and parents for further refinement.

Results:

We conducted 21 interviews and 5 focus groups involving 20 parents. The resulting Dutch and English scale includes 12 items: satisfaction with the scar, color, bumpiness, pain, thickness, tightness, itch, hardness, cords/lines, numbness, hair growth, and burning sensation. Based on children’s preference, literature, and multidisciplinary team discussions, a 5-point emoticon response scale is used. Additionally, a proxy-patient version was developed. Pretesting the scale in children and parents resulted in minor refinements.

Conclusions:

This study marks a significant advancement in scar quality assessment by and for children. The scale was developed with input from children and parents, making it the first patient-reported outcome measurement instrument specifically designed for assessing scar quality in pediatric burn patients. Ongoing field tests and validation aim to establish the scale’s measurement properties.

P231

Comparing parent and child assessments of burn scar quality: reliability of the pediatric patient scale of the POSAS by proxy.

**Kemme, F.** ^1,2,3,4^, Lelifeld, P. ^1^, Anthonissen, M. ^5,6^, Meij-de Vries, A. ^1,2,8,9^, de Cuyper, L. ^5,7^, van Zuijlen, P. ^1,2,3,4,9,10^, Pijpe, A. ^1,2,3,4^

^1^ Burn Center, Red Cross Hospital, Beverwijk, The Netherlands, ^2^ Alliance of Dutch Burn Care (ADBC), Burn Center, Red Cross Hospital, Beverwijk, The Netherlands, ^3^ Amsterdam UMC location Vrije Universiteit van Amsterdam, Department of Plastic Reconstructive and Hand Surgery, Amsterdam, The Netherlands, ^4^ Amsterdam Movement Sciences, Tissue Function and Regeneration, Amsterdam UMC, Amsterdam, The Netherlands, ^5^ OSCARE, Organisation for Burns, Scar After-care and Research, Antwerp, Belgium, ^6^ University of Antwerp, Antwerp, Belgium, ^7^ Burn Center, ZAS CADIX, Antwerp, Belgium, ^8^ Department of Surgery, Red Cross Hospital, Beverwijk, The Netherlands, ^9^ Amsterdam UMC location University of Amsterdam, Paediatric Surgical Center, Emma Children’s Hospital, Amsterdam, The Netherlands, ^10^ Department of Plastic Reconstructive and Hand Surgery, Red Cross Hospital, Beverwijk, The Netherlands.

Poster presentations 3, 4 September 2025, 15:15–15:45

Aim:

To evaluate the agreement between parent- and child-reported scar quality using the pediatric patient scale of the POSAS for burn scar assessment in children.

Methods:

Children aged 8–17 years with burn scars and their parents were recruited from the burn center Beverwijk, The Netherlands, and the aftercare center OSCARE, Belgium. Both the child and one of the parents independently completed the pediatric patient scale of the POSAS, with parents instructed to report from the child’s perspective. Agreement between parent- and child-reports was analyzed by the proportion agreement, kappa, and intraclass correlation coefficients (ICC).

Results:

Preliminary results based on 36 child-parent pairs included between January and March 2025 revealed several discrepancies across different scar characteristics. We observed the highest proportion absolute agreement between parent- and child-reported scores for the item burning sensation (75%), followed by pain (60%) and itch (49%). The lowest agreement is observed for bumpiness and hair growth (both 31%), followed by numbness (34%). The target is to include 50 child-parent pairs. Data collection is ongoing and will reveal to what extent parent-reported scar quality scores deviate from child-reported scores, and if this is particularly pronounced in specific scar characteristics. Final results will be available in September 2025.

Conclusions:

While the use of proxy reports in pediatric burn care is unavoidable, the preliminary findings of our study underscore the necessity to identify the interpretability and reliability of proxy reports in pediatric burn scar assessments, to improve treatment decisions, and to allow for potential calibration in research settings.

P232

Surgical management of pediatric electrical burn injuries—university tertiary center experience in cohort of 52 children.

**Kravljanac, D.** ^1,2^

^1^ Institut for Mother and Child Healtcare of Sebia, Clinic for Pediatric Surgery, Belgrade, Serbia, ^2^ Medical Faculty University of Belgrade, Belgrade, Serbia

Poster presentations 3, 4 September 2025, 15:15–15:45

Aim:

The evaluation of surgical management of pediatric electrical burns in our cohort during the last ten years.

Methods:

The retrospective study included children with electrical burn injuries treated, by different surgical approaches under analgesia/general anesthesia. Patients were reviewed for age, sex, current strength, total body surface area (TBSA) of burn, degree of burn, type of surgical approach and length of healing.

Results:

The study included 52 children (39 boys, 13 girls), treated between 2014–2024. The mean age was 6.9 years (range 1 month-17 year). Most of them were injured by low voltage current (82.69%), and 9 patients (17.31%) suffered high voltage injury. The mean TBSA of burn was 6,5% (range 1% to 60%). All patients had deep dermal or full-thickness burns. The surgical approach was: operative in 18 patients (34.62%) and nonoperative in 34 children (65.38%). The performed surgical procedures were: necrectomy and skin grafting in 16 children, amputation of the digit or a hand in five patients and fasciotomy in two cases. The average time of the burn wound healing was 13.62 (range 6–130) days.

Conclusions:

Pediatric electrical injuries represent a life-threatening condition. Surgical management consists of nonoperative and operative treatment. Acute surgical procedures were reserved mainly for high-voltage injuries including amputations in the most severe patients. Early necrectomy and skin grafting are the gold standard for most children. The specific surgical approach will depend on the extent and severity of the burn.

P233

The game-changing use of NovoSorb Biodegradable Temporizing Matrix (BTM) in a 3 year old with a 75% TBSA burn.

**Lindford, A.** ^1^, Vänskä, J. ^1^, Gästgifvars, J. ^1^, Valtonen, J. ^1^, Vuola, J. ^1^, Schepel, V. ^1^

^1^ Helsinki University Hospital, Helsinki, Finland

Poster presentations 3, 4 September 2025, 15:15–15:45

Aim:

We present a case of the use of BTM in a major paediatric burn that had a dramatic impact on the course and outcome of treatment.

Methods:

A 3 year old boy sustained a 75%TBSA flame burn from cooking marshmallows on a bio ethanol fire and was admitted to the New Children’s Hospital in Helsinki. During the first week all burns were tangentially excised and covered with either autografts, allografts or BTM. On day 7 the patient had become septic with widespread growth of pseudomonas. This resulted in partial loss of the autografts, infected BTM covering the anterior trunk and deepening of the majority of the burn. Radical debridement and coverage with allograft stabilised the situation and on day 11 the majority of the excised burn was covered with BTM (around 50% TBSA). Thereafter donor sites were recycled and small meshed skin grafts and Meek grafts were applied for definitive closure. This was supplemented by the use of cultured epithelial autografts and the Helsinki cell suspension therapy.

Results:

The boy was extubated 14 days post injury, length of ICU stay was 34 days, he was spending time at home by day 42 and was discharged home on day 91.

Conclusions:

BTM proved to be a game changer as it enabled: rapid physiological closure of a large burn wound, early rehabilitation of the child, the use of small mesh skin grafts and obviated the need for repeated allograft skin use.

P234

Open abdomen as a unique late complication 40 years following a 80% TBSA flame burn in a 4 year old.

**Gästgifvars, J.** ^1^, Schepel, V. ^1^, Lindford, A. ^1^

^1^ Helsinki University Hospital, Helsinki, Finland

Poster presentations 3, 4 September 2025, 15:15–15:45

Aim:

We present a cautionary tale of a late life threatening truncal burn scar sequela that to our knowledge has not been previously described.

Methods:

A 42 year old male had suffered a 80% TBSA flame burn as a 4 year old. Nearly 40 years later he underwent elective surgery for hiatus hernia correction. Initially, laparoscopic access was aborted due to failure of carbon dioxide insufflation and converted to a laparotomy. Following hernia repair the abdomen was closed with difficulty due to the tight burn scar. Abdominal compartment syndrome and renal failure developed over the next few days. The abdomen was opened and negative pressure wound therapy (NPWT) commenced. He required widespread truncal ‘burn scar escharotomies’ to further improve ventilation and these subsequent escharotomy wounds were closed with BTM, cell suspension therapy and Meek skin grafts. Abdominal wall closure was assisted with a combination of Botolinum toxin A (BTA) mediated chemical muscle relaxation and mesh-mediated fascial traction (MMFT) and NPWT. Ultimately, the abdominal defect was closed with skin autografts harvested from the scalp and allograft sandwich grafts.

Results:

Abdominal wall closure was achieved within 2 months and the patient discharged home at 3 months. The closure has remained stable at 1.5 years follow up.

Conclusions:

This case highlights the need to consider the risks of abdominal surgery following deep paediatric truncal burns.

P235

Bromelain-based enzymatic burn debridement in the children: a systematic review of clinical studies on patient safety, efficacy and long-term outcomes.

**Marino Garcia, M.** ^1^, Yuste, V., Iglesias, J.

^1^ Hospital Universitario Miguel Servet, Zaragoza, Spain

Poster presentations 3, 4 September 2025, 15:15–15:45

Aim:

Our purpose was to assess the evidence of NexoBrid application in children with intermediate and deep-thickness burns concerning efficacy, safety, logistic and functional and cosmetic outcomes in children.

Methods:

We conducted a systematic review of the literature, including main databases and the references of included articles. The participants were children younger than 18 years old who underwent application of NXB in intermediate and full-thickness burn injuries. The efficacy, safety, logistic and functional and cosmetic outcomes were compared with the SOC (tangential, Versajet or dermabrasion debridement) and/or the application of NXB in adults.

Results:

Despite the scarce literature, promising efficacy and logistics outcomes have been found. Both RCT found significant reduction of time to complete debridement and surgical burden. Korzenowski et al. suggested a reduction of length of stay. We found promising selectivity outcomes, such as the reducing number of patients necessitating autografting after NXB debridement. Long-term outcomes follow the same lines. Claes et al. strongly appeal to limiting the operating room procedures and to developing new pain management protocols, specially in children suffering from burn injuries of one upper limb or one or both lower limbs.

Conclusions:

The management of burn children presents a formidable challenge that NXB may alleviate in several aspects. The clinical benefits of an early eschar removal with reduced need for surgery and its related morbidity while maintaining a favourable long-term final outcome is of particular importance in paediatric patients. Additional studies with NXB in the paediatric population are needed to further strengthen these results.

P236

Management of thermal burns in neonates in a tertiary center—A 5-year retrospective study

**Martinov, M.** ^1^, Argirova, M. ^1^, Pashov, I. ^1^

^1^ Umhatem “N.I. Pirogov”, Sofia, Bulgaria

Poster presentations 3, 4 September 2025, 15:15–15:45

Thermal injuries in neonates are rare, often result of an accident or iatrogenic injury. They have unique physiological and anatomical characteristics which reflect in the overall management of the thermally injured babies. Their management is difficult as the exact protocols are not clearly depicted.

Aim:

The aim of the study is to analyze retrospectively the magnitude of injury, local wound management, role of surgery and outcome in neonatal burns admitted to two Burns Centers.

Methods:

Between 2019 and 2023, nineteen neonates, aged less than 28 days with burns of various areas, depth and localization were managed in two Burn centres in Bulgaria. Demographic and statistical analyses have been carried out. Topical wound care started with SSD and was changed with Acticoat and Aquacel Ag.

Results:

The mean age of the neonates was 17.32 ± 6.7 days. The scalds were the commonest mechanism of the injury. The mean TBSA burnt was 7.16% ± 8.11. The most common areas affected were face, chest and upper limb. Seven sustained superficial partial thickness burns, 10 deep partial thickness and 2 full thickness injuries. The mean hospital stay was 9.31 ± 7.80 days. The mortality was 5.26%. Hypertrophic scars were observed in 9 babies. The burn wounds fully epithelized in 16 neonates. Only 3 babies were operated on in one stage procedure—early tangential excision and split thickness autografts.

Conclusions:

Neonatal burns are extremely challenging to the burn team and require special attention. Adequate resuscitation, close monitoring, topical wound care, skin grafting (when indicated), remain the keynote of treatment.

P237

The role of negative pressure wound therapy (NPWT) in the management of pediatric burns: a five-year experience.

**Nacea, D.** ^1,2^, Enescu, D. ^1,2^, Stoica, C. ^2^, Prisecaru, B. ^2^, Tatar, R. ^1,2^

^1^ ”Carol Davila” University of Medicine and Pharmacy, Bucharest, Romania, Bucharest, Romania, ^2^ ”Grigore Alexandrescu” Clinical Emergency Hospital for Children, Bucharest, Romania, Bucharest, Romania.

Poster presentations 3, 4 September 2025, 15:15–15:45

Aim:

The purpose of our study is to synthetize the experience of our department regarding the role of NPWT in pediatric patient burns, in order to establish its place within burn treatment protocols.

Methods:

We performed a retrospective study from January 2020 till December 2024. The inclusion criteria were the presence of burn injury, in-patient treatment, and the use of the NWPT. From the medical files of the selected cases we collected demographic data regarding the patients, clinical description of the burn injury, surgical approach, NPWT details, complications and outcomes.

Results:

We identified 17 patients, 12 boys and 5 girls, aged between 10 months and 17 years. They were admitted with burned areas ranging from 5% to 80% TBSA. With regard to etiology, there were 6 scalds, 7 electrical burns and 4 flame burns. We could establish two main indications for using NPWT in our department. First, immediate post-burn use for deep-partial burns, showing a rapid decrease of the edema and enhancing further healing by spontaneous re-epithelialization in most of cases. The second indication is for very deep, 4th degree burns with tendons and bones exposed, sometimes associated also with presence of local infections, when the purpose of NPWT was to stimulate granulation tissue formation and to prepare the wound bed for grafting.

Conclusions:

NPWT proved to be a safe and effective tool for the management of pediatric burns in multiple indications, depending on the depth and extension on the wound.

P240

Four in a row—lessons learnt from a very busy year in Cologne’s paediatric burn centre

**Pohle, R.** ^1^, Wendenburg, W. ^1^, Acero Moreno, D. ^1^, Klein, T. ^1^, Dittmann, L. ^1^, Boemers, T. ^1^

^1^ Paediatric Burn Centre, Children’s Hospital of Cologne, Cologne, Germany

Poster presentations 3, 4 September 2025, 15:15–15:45

Aim:

The incidence of very severe burns in children in Germany has decreased within the last decades. With 20 paediatric burn centres in Germany, this decrease of incidence has lead to a decreasing number of patients per burn centre, with some burn centres treating only one severely burnt child every few years. Since the beginning of the war in Ukraine, Europe has suddenly been confronted with a high number of severely burnt children. We would like to share our experience of treating several within a short period of time.

Methods:

Our paediatric burn centre in Cologne treated three severely burnt children from Ukraine and one local child with severe burn injuries within one year, quadrupling our normal incidence of critically burnt children. The treatment modalities, challenges, problems and outcomes are discussed.

Results:

All four patients had severe burn injuries TBSA > 60% and survived with a good overall outcome. The wounds were treated using dermal templates, cultured keratinocytes and non-cultured cell-suspensions in combination with split-thickness skin grafting.

Conclusions:

This very busy year has tought us many lessons, be it surgically, microbiologically, psychologically, logistically, or teamwork-wise. We would like to share our experience with the many other burn centres in Europe that might face similar challenges.

P241

Polylactide copolymer in pediatric facial burns: a useful substitute for facial skin regeneration

**Ribeiro, S.** ^1^, Fernandes, S. ^1^, Barbosa-Sequeira, J. ^1^, Campos, M. ^1^, Garcia, M. ^1^

^1^ Unidade Local De Saúde São João, Porto, Portugal

Poster presentations 3, 4 September 2025, 15:15–15:45

Aim:

To report the successful use of polylactide copolymer in facial burns in children, with favorable short and long-term outcomes.

Methods:

The adequate management of a facial burn is of true importance due to the inherent negative social impact that sequelae scars in this area can create. Suprathel^®^ is a polylactide copolymer membrane classically used as an epidermal substitute in superficial skin loss. We applied Suprathel^®^ in facial superficial partial-thickness skin burns of 4 pediatric patients. Secondary dressing was executed in alternating days without sedation or opioid use. Demographic data and short and long-term outcomes were collected and evaluated.

Results:

Complete re-epithelialization was verified in all cases after 1 week. No pain was reported during this process. Patients were discharged and followed at regular pediatric surgery burn consultation. Median follow-up time was 30 months (min 4:max 59, months). No skin loss, infection, hypertrophy or significant heterochromia were registered during this period.

Conclusions:

Optimizing facial burn treatment is a challenge because of the intrinsic difficulties of dressing application and changes, especially in a child. Polylactide copolymer permits opioid and sedation-free care in these patients. Suprathel^®^ allowed not only an efficient experience, but also an effective treatment with rapid regeneration of facial damage and successful follow-up results. To our knowledge, this is one the first group of cases reporting the use of polylactide copolymer specifically on the face.

P242

Experience after implementing a protocol for enzymatic debridement application in pediatric burn population in a tertiary center.

**Santin Perez, N.** ^1^, Minguez, S. ^1^, Martin, P. ^1^

^1^ Osakidetza, Barakaldo, Spain

Poster presentations 3, 4 September 2025, 15:15–15:45

Aim:

To describe the protocol for the use of enzymatic debridement in pediatric population stablished at Cruces University Hospital, and describe our clinical experience.

Methods:

A systematic review of the available articles was done, and a detailed multidisciplinar protocol was stablished in 2021. This protocol was used in all pediatric burn patients treated with enzymatic debridement, and data were collected regarding sedation, monitoring, outcomes, complications (major and minor), and demographic data.

Results:

Between 2021–2024 the protocol was used in 20 patients with ages between 11 months and 16 years, mean total body surface affected was 15% (1–60%) and total hospital stay mean was 22.9 days (1–69 days). Most frequent etiology was scald (11 cases), followed by flame (7 cases) and electric (2 cases). No major complications were observed (renal failure, sepsis, anemia) and a low rate of minor complications was recorded (8 local infections and 5 corrected hypovolemias). The implementation of the protocol has allowed a better coordination between medical and nursing staff from Emergency Department, Great Burns Unit and Pediatric ICU, reducing complication rates and enabling a more standardised treatment of this patients.

Conclusions:

In our experience the use of a multidisciplinary protocol allows a safer and more efficient management of pediatric population that might benefit from the use of enzymatic debridement. It optimizes the quality of patients care and accelerates the whole process facilitating better outcomes and minimizing complications.

P243

Managing children with Steven-Johnson syndrome/toxic epidermal necrolysis in a tertiary burns center: our experience over a 12-year period.

**Obaid, S.** ^1^, Bunni, L. ^1^, Swist-Szulik, K. ^1^, Shah, M. ^1^

^1^ Manchester Foundation NHS Trust, Manchester, United Kingdom

Poster presentations 3, 4 September 2025, 15:15–15:45

Aim:

To review current management of children with SJS/TENS, identify challenges and lessons learnt.

Methods:

A retrospective cohort study of children presenting with SJS/TEN syndrome, admitted to our burn center over last 12 years was performed. Clinical records were reviewed. Demographics, prodromal symptoms, management during the entire course of illness and outcomes were extracted into an Excel spreadsheet. Numerical data were analysed by simple statistics. Thematic analyses of descriptive data were performed and lessons learnt were identified.

Results:

11 patients (age: median 76 months; range:17–158 months) were admitted to our burn center and all patients survived. The median body surface area involved was 56.25% TBSA (range: 11.25–95%). 7 of the 11 patients required PICU care. Median length of hospital stay was 19.5 days; range:4–53. Pain and nutritional support were proactively managed. Immunoglobulin was given to severely affected patients only and no-one received immunosuppressants.

Facial swelling with oral mucosal bleeding posed challenges to oral hygiene and safe airway management. This was facilitated in 2 patients with tracheostomy. Wound management involved a thorough clean, debridement of loose skin, diagnostic biopsies and appropriate dressings, in theatre. Where the wounds did not appear infected, 50:50 paraffin ointment was applied; where wound swabs demonstrated colonisation/infection, ActicoatTm was used, and all wounds were dressed. No patients required excision or grafting.

One patient has puckered post-tracheostomy scarring requiring revision; 4 patients are having on-going interventions for eyes.

Conclusions:

Early, involvement of multi-disciplinary team and close post-discharge follow-up are key to management of these rare complex patients.

P245

Long-term outcomes of firework-related burns in children

**Van Balen, N.** ^1^, de Haan, J. ^1^, Stoop, M. ^1,2^, Pijpe, A. ^2,3,4^, Meij-de Vries, A. ^2,5,6^

^1^ Burn Center, Red Cross Hospital, Beverwijk, The Netherlands, ^2^ Alliance of Dutch Burn Care (ADBC), Burn Center, Red Cross Hospital, Beverwijk, The Netherlands, ^3^ Amsterdam UMC location Vrije Universiteit Amsterdam, Department of Plastic, Reconstructive and Hand Surgery, Amsterdam, The Netherlands, ^4^ Amsterdam Movement Sciences (AMS), Tissue Function and Regeneration, Amsterdam, The Netherlands, ^5^ Department of Surgery, Red Cross Hospital, Beverwijk, The Netherlands, ^6^ Amsterdam UMC location University of Amsterdam, Pediatric Surgical Center, Emma Children’s Hospital, Amsterdam, The Netherlands.

Poster presentations 3, 4 September 2025, 15:15–15:45

Aim:

To evaluate the long-term impact of firework-related burns in children.

Methods:

A single-center, cross-sectional study was conducted at the burn center of the Red Cross Hospital in Beverwijk, The Netherlands. Children under the age of 18 who sustained burns from fireworks and were treated between 2019 and 2024 were eligible for inclusion. Both children and their parents were invited to participate in either a face-to-face or online questionnaire-based interview, focusing on the long-term physical and psychosocial consequences of the injuries. Additionally, participants were asked about their treatment experience and their current behaviours regarding firework use.

Results:

Of the 108 children treated for firework-related burns, 35 were included. All participants were boys with a mean age of 11 years. Most burns were caused by low-category fireworks, specifically “children’s fireworks”, with improper handling being the primary cause of injury. The majority of burns had a mixed dermal depth, with a median Total Body Surface Area (TBSA) of 0.5%. Long-term physical and psychosocial impacts were minimal. Both children and parents generally rated the provided care positively. The injuries and subsequent scarring had a limited effect on the children’s current firework-related behavior, although most reported taking greater caution when handling fireworks.

Conclusions:

Firework-related burns predominantly affect young boys and are primarily caused by low-category fireworks. Given the continuous use and strong appeal of fireworks, combined with the fact that most injuries result from improper use, prevention campaigns are likely to be most effective when focused on safe handling and (parental) supervision.

P246

Dermal Substitution with Glyaderm in Paediatric Burns

**van de Warenburg, M.** ^1,2^, Hummelink, S. ^2^, Ulrich, D. ^1,2^, Vehmeijer-Heeman, M. ^1,2^

^1^ Amalia Centre of Expertise Pediatric Trauma and Burns, Radboud University Medical Centre, Nijmegen, The Netherlands, Nijmegen, The Netherlands, ^2^ Department of Plastic, Reconstructive, and Hand Surgery, Radboud University Medical Center (Radboudumc), Nijmegen, The Netherlands.

Poster presentations 3, 4 September 2025, 15:15–15:45

The gold standard in accelerated closure of deep dermal to full-thickness burns is the use of split-thickness skin grafts (STSGs). However, this often results in hypertrophic scarring and contractures, presumably due to a lack of dermal components. Therefore, treatment of deeper burns demands the application of both a skin graft and a dermal component. Glyaderm is a dermal substitute derived from human cadaveric donors. Previous studies have shown that using Glyaderm in adult burns leads to better scar quality compared to control wounds treated with STSGs alone.

A prospective case series is being conducted including 20 pediatric patients who have deep burn wounds, aiming to evaluate the effect of Glyaderm on scar maturation and quality. The primary outcomes include assessing scar quality using the Patient and Observer Scar Assessment Scale (POSAS) at 3, 6, and 12 months postoperatively. Secondary objectives include measurements on wound and donor site healing, scar surface area over time, quality of life using the TAPQOL or PedsQL instruments, and the rate of scar hypertrophy and contractures.

Currently, 8 patients have been enrolled, with a mean age of 5 years (range 10 months–12 years). The burn etiologies include hot water (57%), flame (29%), and grease (14%). The mean Total Body Surface Area affected is 9.9% (range 1–20%).

Preliminary results suggest favorable scarring, indicating that Glyaderm may be a promising treatment option for pediatric full-thickness burns.

P247

Would children benefit more than adults from enzymatic debridement? What we learned in the last 5 years.

**Vigato, E.** ^1^, Edoardo, D., Maurizio, G.

^1^ Aoui Verona, Italy, Verona, Italy

Poster presentations 3, 4 September 2025, 15:15–15:45

Aim:

Enzymatic debridement has been approved few years ago also for pediatric patients. Many studies demonstrated the safety and efficacy of Nexobrid in adults, but the experience in pediatric patients has still to be consolidated. The child burn patient could theoretically benefit the most from early treatment: sparing dermis and the reduction of the area to be grafted leads to better functional and aesthetical outcomes.

Methods:

A retrospective study was conducted on pediatric patients admitted to Verona Burn Center from 2020 to 2025. The group of children treated with enzymatic escharolysis was compared with a similar group treated according to the Standard of care (Topical dressings/homografts + eventual surgery) in terms of length of hospitalization, need for surgery, extent of surgery, complications and quality of scars.

Results:

27 of the 84 pediatric patients (mean age 5.3 years, range 0.6–17 years) admitted since 2020 were treated with Nexobrid (75% within the first 24 h, 20% within the first 48 h, 5% on the third day). The mean TBSA treated is 9% (range: 3–65%). Five children had deep burns greater than 50%. Patients treated with Nexobrid underwent tangential escarectomy in 20% of cases, with a smaller area covered with autograft compared to the control group. Mean hospital stay was comparable in the two groups (14.3 days). No adverse events occurred.

Conclusions:

Enzymatic debridment is a safe and effective procedure in pediatric burn patients, with a reduction of the area to be grafted, and better healing results.

P248

Understanding burn-related neuropathy in children: clinical and neurophysiological perspectives

Rubino, A., De Martino Bernardo, M., Bruno Bernardo, G., Esposito, F., **Zamparelli, M.** ^1^

^1^ Santobono Pausilipon National Children’s Hospital, Napoli, Italy

Poster presentations 3, 4 September 2025, 15:15–15:45

Aim:

Peripheral neuropathy is a debilitating consequence of severe burns, under-diagnosed in pediatrics, with prevalence rates ranging from 11 to 52%. Various neuropathy types have been observed: mononeuropathy, multi-mononeuropathy, polyneuropathy. Their occurrence is linked to injury mechanism, total body surface area (TBSA) affected, depth of burns. This study aims to delineate clinical and neurophysiological aspects of burn-related neuropathy in a pediatric case series.

Methods:

Four pediatric patients (aged 5–12) with burns >25% TBSA admitted to our Pediatric Burn Center were studied. Patients underwent neurological and neurophysiological examination. MRI and US of peripheral nerve were performed.

Results:

Decreased of coumpoud motor and sensory action potential with a length-dependent distribution ascribable to axonal polyneuropathy occurred in all patients. Overlap with multiple mononeuropathy was observed in 3 and isolated mononeuropathy in 1. Mononeuropathies were observed in burned (3) as well as in unburned areas (3) with involvement of upper (3) and lower (2) nerve. After 6 months, axonal polineuropathy improved in all patients; partial recovery occurred in mononeuropathy.

Conclusions:

Peripheral neuropathies are complications of severe burn injury, especially in critically ill. In our series, patients displayed clinical and instrumental evidence of complex involvement, probably because of the heterogeneous underlying pathogenic mechanisms. Peripheral neuropathy is well-documented in adult burn populations; systematic studies in pediatrics are lacking. This report represents the first pediatric case series of burn-related neuropathy. Early and accurate neurological assessments coupled with neurophysiological studies and imaging evaluation should be systematically performed to minimize morbidity associated with burn-related peripheral nervous system involvement.

P250

Facilitating wellbeing and recovery for burn patients through outdoor sports: A sports day event

**Armitage, L.** ^1^

^1^ St. Andrews Centre, Chelmsford, Chelmsford, United Kingdom

Poster presentations 3, 4 September 2025, 15:15–15:45

Purpose: This aimed to improve the physical and psychological well-being of burn patients and enhance social interaction by introducing a ‘sports day’ themed afternoon, providing outdoor physical activity opportunities within the hospital setting. The expected outcomes included improved mood, increased self-esteem, and enhanced social interaction.

Methods:

The study used the Plan, Do, Study, Act (PDSA) methodology to introduce the ‘sports afternoon’. Feedback was gathered from burn patients and staff participants through structured surveys to assess the impact of the event on physical activity, mood, and social interaction.

Results/Discussion: Five patients attended the sports day: two paediatrics (mean age 11.5 years, range 10–13 years) with a mean TBSA of 33.5% (range 22–45%) and three adults (mean age 56.3 years, range 18–82 years) with a mean TBSA of 32.23% (range 12–45%). Feedback was provided by 100% of participants and was positive. Patients expressed appreciation for the opportunity to engage in outdoor activities, which allowed them to temporarily escape the hospital environment. Many reported improved mood, increased self-esteem, and enhanced social interaction, underscoring the importance of meaningful engagement during their recovery process.

Conclusions:

Implementing outdoor ‘sports days’ enhances burn patient recovery by combining physical activity with outdoor exposure, leading to improvements in mood, self-esteem, and physical health. The results suggest that such events are a valuable component in promoting holistic recovery for burn patients and should be considered for broader implementation in hospital settings.

P251

BreeZe—a program for self-management support after a burn injury

**Blok, S.** ^1,2,3^, Nieuwenhuis, M. ^1,3,4^, Scholten-Jaegers, S. ^1^, Niemeijer, A. ^1^, Bijker, G. ^1^, Visser, I. ^5^, van der Sluis, C. ^2^, van Uden, D. ^6^, Verwilligen, R. ^7,8,9^, Boekelaar-van den Berge, A. ^7^, Bosma, E. ^1,10^, Geelen, S. ^1,3^

^1^ Alliance of Dutch Burn Care (ADBC), Burn Centre, Martini Hospital, Groningen, The Netherlands, ^2^ University of Groningen, University Medical Center Groningen, Department of Rehabilitation Medicine, Groningen, The Netherlands, ^3^ Research group Healthy Ageing, Allied Health Care and Nursing, Hanze University of Applied Sciences, Groningen, The Netherlands, ^4^ University of Groningen, University Medical Center Groningen, Department for Human Movement Sciences, Groningen, The Netherlands, ^5^ Dutch Association of Burn Survivors, Beverwijk, The Netherlands, ^6^ Alliance of Dutch Burn Care (ADBC), Burn Centre, Maasstad Hospital, Rotterdam, The Netherlands, ^7^ Alliance of Dutch Burn Care (ADBC), Burn Centre, Red Cross Hospital, Beverwijk, The Netherlands, ^8^ Amsterdam UMC location Vrije Universiteit Amsterdam, Department of Plastic, Reconstructive and Hand Surgery, Amsterdam, The Netherlands, ^9^ Amsterdam Movement Sciences (AMS), Tissue Function and Regeneration, Amsterdam, The Netherlands, ^10^ Martini Hospital, Department of Surgery, Groningen, The Netherlands.

Poster presentations 3, 4 September 2025, 15:15–15:45

Aim:

The aim of this study was to develop a self-management support program specifically designed for burn survivors.

Methods:

Between April 2022 and April 2024, the self-management support program BreeZe was developed to improve burn survivors’ self-management skills after discharge from a burn center using a co-creational approach including burn survivors, healthcare professionals, and burn care managers. This approach followed a structured 5-stage process using established frameworks for designing complex interventions.

Results:

BreeZe (‘BRandwonden En ZElfmanagement) is a self-management support program that aims to enhance burn survivors’ intrinsic motivation and self-efficacy regarding self-management post-discharge and includes five key constructs: (1) a holistic approach, (2) shared decision-making, (3) goal setting and action planning, (4) a solution-focused approach, and (5) case-management. BreeZe accomplishes this by providing healthcare professionals education on self-management, training on communication skills (e.g., motivational interviewing), and communication- and decision aid tools. Furthermore, we developed a workbook for burn survivors. In practice, burn survivors will identify and prioritize their problems using a decision aid tool (i.e., the self-management web), set personalized goals together with their healthcare professional, and form an action plan for goal attainment.

Conclusions:

Self-management skills are essential for burn survivors to manage and integrate the consequences of a burn injury into their daily life. Healthcare professionals can play an important role in supporting the self-management of burn survivors. At present, there are no burn-specific self-management support interventions. To address this gap, we developed BreeZe and are currently performing an evaluation study to assess the effectiveness of BreeZe.

P252

Self-reported health evaluation and image satisfaction in severe burn patients undergoing rehabilitation

**Carvalho, E.** ^1^, Mazzoti, B. ^1^, Leger, B. ^2^, Phuoc Duong, H. ^2^, La Carré, J. ^2^, Crittin, V. ^2^, Lecroc, S. ^1,2^, Iakova, M. ^1,2^

^1^ Departement of Musculoskeletal Rehabilitation, Clinique Romande de Réadaptation, Sion, Switzerland, ^2^ Department of Medical Research of the Suva clinics, Clinique Romande de Réadaptation, Sion, Switzerland

Poster presentations 3, 4 September 2025, 15:15–15:45

Aim:

To assess the burn specific aspects of function, satisfaction with appearance and body image in severely burned patients using patient-reported questionnaires.

Methods:

We included patients hospitalized in a tertiary rehabilitation center with ≥20% of Total Body Surface Area (TBSA) between 2012 and 2021. We used the 40-item Burns Specific Health Scale-Brief (BSHS-B) questionnaire; and the 14-item Satisfaction with Appearance Scale (SWAP) at discharge to assess health aspects.

Results:

Forty-eight patients were included. The median age was 37 years (range 16–69 years) and 73% were men. Median TBSA = 45% (range: 20–90%). Twenty-five patients completed BSHS-B. The median scores of the 9 subscales of the BSHS-B were as follow: simple abilities (3.5), hand function (3.6), affect (3.1), interpersonal relationship (4), sexuality (3.7), body image (3), heat sensitivity (1.5), treatment regimen (2.8) and work (2.0). Twenty-one patients completed the SWAP questionnaire. The median score of SWAP was 41 points (range 4–84), and its 4 subscales scores were as follows: facial: 10 (range 0–24), non-facial: 12 (range 0–24), social distress: 3 (range 0–18) and perceived social impact: 8 (range 0–18).

Conclusions:

The burn treatment regimen and heat sensitivity, remain challenging to manage. For the patients, dissatisfaction with appearance was also found in facial and non-facial features and perceived social impact. These elements must be taken into consideration in order to improve our rehabilitation care.

P253

Improving Stump Volume in Limb Amputations with Dermal Matrices

**Cordova-Orrillo, J.** ^1^, Lobaton-Rosas, R., Perez-Sovero, R.

^1^ Auna Hospitals, Lima, Peru

Poster presentations 3, 4 September 2025, 15:15–15:45

Aim:

To demonstrate that using multi-layered dermal matrices in stump reconstruction increases volume, creating a soft and resilient cushion that facilitates prosthetic or orthotic use. This approach helps prevent ulcerations and complications while preserving stump length, which has proven beneficial for functional outcomes in physical medicine and rehabilitation.

Methods:

We present nine cases of traumatic amputations involving upper and lower limbs, including both avulsion and necrosis injuries. The procedures included the application of multi-layered dermal matrices:

- Seven cases with a dermal regeneration template (collagen-glycosaminoglycan matrix).
- Two cases with a collagen and elastin filler to improve stump morphology and prosthetic adaptation.

Negative pressure therapy was used to enhance matrix adherence, and meshed skin grafts were applied using the Meek system. Follow-up included ultrasound evaluation of stump integration and functionality.

Results:

- Patient demographics: Nine cases, age 28–55 (mean 38.7), 66.7% male, 33.3% female.
- Types of amputations:
- Upper limb: 4 cases (2 avulsion, 2 necrosis by attrition).
- Lower limb: 5 cases (4 thigh, 1 bilateral leg).
- Clinical outcomes:
- Integration rate: 97–100%.
- Improved scar quality and pliability, resembling normal skin.
- Increased stump volume with adequate cushioning, preventing ulceration.
- Stump length preserved, optimizing prosthetic adaptation.
- No significant complications or graft rejections.

Conclusions:

The use of multi-layered dermal matrices, including both collagen-glycosaminoglycan and collagen-elastin fillers, effectively improves stump volume and cushioning. This approach enhances prosthetic adaptation, minimizes ulceration risk, and preserves stump length, crucial for functional rehabilitation. Further studies are recommended.

P254

Health recovery in burn patients: comparing Belgian aftercare approaches in the BRANACO study.

**Demarbaix, T.** ^1,2^, Mao, Z. ^3^, Moortgat, P. ^1^, Legrand, M. ^5^, De Cuyper, L. ^1,4^, Beutels, P. ^3^, Maertens, K. ^1,6^

^1^ OSCARE, Organisation for Burns, Scar Aftercare and Research, Merksem, Belgium, ^2^ Department of Rehabilitation Sciences and Physiotherapy REVAKI-MOVANT, Faculty of Medicine and Health Sciences, University of Antwerp, Wilrijk, Belgium, ^3^ Centre for Health Economics Research and Modelling Infectious Diseases (CHERMID), University of Antwerp, Wilrijk, Belgium, ^4^ Burn Centre, ZAS Cadix, Antwerp, Belgium, ^5^ Burns Foundation, Brussels, Belgium, ^6^ Department of Clinical and Lifespan Psychology, Vrije Universiteit Brussel, Brussels, Belgium.

Poster presentations 3, 4 September 2025, 15:15–15:45

Aim:

This study examines how different aftercare approaches in Belgium affect burn patients’ HRQoL, providing insights for healthcare providers and policymakers.

Method:

A prospective observational study comparing aftercare approaches over 12 months, assessing HRQoL using EQ-5D-5L utility scores and VAS.

Results:

Given that the data collection is still ongoing, the current analysis is based on the available sample. This preliminary report analyzes a total of 101 patients: 36 in specialized aftercare, 50 in outpatient burn clinics, and 15 in home care. Severe burns (>20% TBSA) were more frequent in outpatient clinic patients (31%) than in specialized aftercare (8%).

Specialized aftercare patients showed the highest HRQoL, with utility scores increasing from 0.73 to 0.87 and VAS from 68.25 to 81.90. Home care patients had the lowest scores, with utility values rising from 0.25 to 0.42 and VAS from 45.6 to 52.47. Outpatient clinic patients saw moderate improvements, with utility increasing from 0.39 to 0.51 and VAS from 54.74 to 55.31. Linear mixed models were adopted to address the issue of missing data and confirmed that the estimated HRQoL was highest in specialized aftercare and lowest in home care.

Conclusions:

HRQoL outcomes indicate that intensive aftercare may aid recovery, but several influencing factors must be considered, including differences in care structure, accessibility, and provider specialization. Patient characteristics and baseline health may partly explain outcome variations. The ongoing data collection limits current findings, necessitating further statistical modeling to control for confounding variables such as gender, age, TBSA, and other health-related factors.

P256

Prevalence of kinesiophobia among burned population. The implications of fear of movement

**Grossi Garriga, L.** ^1^, Torrent Bertran, M., Montinari, M., López Lebrato, S.

^1^ Hospital Universitari Vall D’hebron, Barcelona, Spain

Poster presentations 3, 4 September 2025, 15:15–15:45

Aim:

The aim of this study is to identify and quantify the presence of kinesiophobia in burned patients, through a specific intervention in subacute outpatients who attend treatment in our therapeutic area.

Methods:

A comprehensive literature review was conducted to examine existing research on kinesiophobia in burned patients; however, no significant results were found addressing this specific population.

Subacute outpatients in our area were screened with a two-day timed intervention using Tampa Scale for Kinesiophobia (TSK) and Generalized Anxiety Disorder Scale (GAD-7) in order to identify symptoms of kinesiophobia.

Results:

Preliminary findings indicated a notable prevalence of kinesiophobia, with many individuals demonstrating a heightened fear of movement or reinjury. Six outpatients aged 26 to 79 responded to the TSK, with scores ranging from 23 to 40, indicating a high presence of kinesiophobia. Regarding the GAD-7 Scale, results varied: 2 patients scored 0–4 (minimal anxiety), 3 patients scored 5–9 (mild anxiety), and 1 patient scored over 15 (severe anxiety), suggesting a strong link between psychological distress and fear of movement.

Conclusions:

The identification of kinesiophobia in burn patients is critical for designing effective rehabilitation programs. Addressing this fear through targeted interventions, including gradual exposure to movement, can enhance functional abilities and improve overall recovery outcomes.

The findings underscore the importance of creating a specialized rehabilitation therapy to optimize treatment strategies and support patients’ long-term functional independence. Therefore, we propose to implement in our Unit a systematic data collection using the above questionnaires to determine the prevalence of kinesiophobia in our area.

P257

Return to customs and fire culture after suffering burns. The experience of correfocs

**Grossi Garriga, L.** ^1^, Torrent Bertran, M. ^1^, Montinari, M. ^1^

^1^ Hospital Universitari Vall D’hebron, Barcelona, Spain

Poster presentations 3, 4 September 2025, 15:15–15:45

As occupational therapists, our aim is enable our patients to return to their daily life activities, not only basic and instrumental tasks, but also those meaningful activities that make up their roles, and those in which they participate within their community or cultural group.

In Catalonia there is a deeply rooted culture and tradition linked to activities with fire, starring groups of devils who meet and perform dances with flares and fire, and often with figures from the Catalan imagination representative of their town and area.

Although it may seem the opposite, in correfocs the participants are protected, and very rarely have we received patients with significant burns during these festive events.

However, when this has happened, and if it is the patients wish, our duty as therapists is to facilitate the return to these activities. Therefore, working on the motor skills needed to participate in a correfoc, from the manipulation of flares to dancing and torches, and also working on possible fears of fire activities, giving guidelines to be able to approach the fire again, the smell of gunpowder, and to return safely to the activity where the burn occurred.

In conclusion, our mission is to enable patients to effectively and safely return to their meaningful activities, including those involving fire.

P258

Impressions of the usability of LPG treatment in burns. A pilot study

**Grossi Garriga, L.** ^1^, Torrent Bertran, M. ^1^, López Lebrato, S. ^1^, Garcia Rodríguez, G., Garcia Gutiérrez, R., Quintana Araya, A.

^1^ Hospital Universitari Vall d’Hebron, Barcelona, Spain

Poster presentations 3, 4 September 2025, 15:15–15:45

A pilot study was made to assess the scar evolution, quality of life and satisfaction survey after using LPG endermologie technique.

It was made with 4 patients using an endodermic mechanotherapy treatment with LPG on their scars, showing an improvement measured with the Vancouver Scale, with an average percentage difference of 50% (25–110%). However, due to the sample shortage, it’s not possible to asseverate if this improvement is given by the treatment or by the normal evolution of the scar.

In order to resolve this question, we design a treatment and assessment protocol for this technique.

A control group study with blindness observer, will compare the scar evolution, quality of life and patient’s satisfaction survey after 2 months of treatment.

Adult outpatients with 2nd and 3rd degree burns in a subacute phase will be recruited during 4 months. All patients will receive the standard rehabilitation treatment, and the study group will also do the treatment with LPG machine twice a week during 2 months.

Beginning and ending evaluations will be made with blind observer, collecting general information of the patients, scar assessment with Vancouver Scale for observer and POSAS Scale for patient, quality of life with Burns Specific Health Scale—Brief (BSHS-B) and a satisfaction survey with Likert Scale.

Our aim is corroborate our clinical impression of the pilot study through a regulated study to see if there are statistically significant differences treating burned scars with LPG to avoid or reduce hypertrophy.

P259

Thematic and Methodological Analysis of Theses in the Field of Burn Rehabilitation in Turkey

**Seyyah, M.** ^1^

^1^ Fenerbahçe University, İstanbul, Turkey

Poster presentations 3, 4 September 2025, 15:15–15:45

Aim:

This study aims to analyze postgraduate theses in the field of burn rehabilitation in Turkey from thematic and methodological perspectives and to evaluate the academic development of the field.

Materials and Methods:

Theses in Physiotherapy and Rehabilitation were scanned via the Turkish Council of Higher Education’s “National Thesis Center.” Eleven theses (8 Master’s, 3 Doctoral), completed between 1984 and 2023 and selected using keywords like “burn,” “rehabilitation,” and “physiotherapy,” were included. A researcher-developed thesis review form was used to evaluate data such as year, university, thesis type, method, advisor’s academic title, sample, assessment tools, and publication status.

Results:

Of the 11 theses, 73% were Master’s and 27% were Doctoral. The oldest was from 1984, and the newest from 2023, with 82% completed in the last decade. Most (82%) were prospective clinical studies; one was an animal study. Advisors were mostly professors (55%). The theses were mainly conducted at Hacettepe University (*n* = 4) and Hasan Kalyoncu University (*n* = 3). Seven theses (64%) were later published in peer-reviewed journals. The most productive period was between 2018 and 2021.

Conclusions:

Burn rehabilitation is an increasingly active research area in Turkey. Despite the growth, improving methodological quality, fostering interdisciplinary collaboration, and increasing publication rates remain key for sustainable academic development.

P262

Nutritional interventions and barriers for patients early after burn injury: a retrospective evaluation of medical records.

**Dimander, J.** ^1^, Huss, F. ^1^, Andersson, A. ^2^, Miclescu, A. ^1^, Lindqvist, C. ^3^

^1^ Uppsala University, Uppsala, Sweden, ^2^ Uppsala University, Uppsala, Sweden, ^3^ Karolinska Institute, Stockholm, Sweden

Poster presentations 3, 4 September 2025, 15:15–15:45

Aim:

To describe nutritional interventions, identify barriers to nutritional intervention, and compare the documentation of nutrition for patients after minor and major burn injuries.

Methods:

A retrospective single-centre medical record review was conducted. Patients aged ≥18, admitted for ≥72 h at our burn centres between 2017 and 2019 were included. Content analysis and descriptive statistics were employed, and differences were explored between patients with minor and major burn injuries.

Results:

A total of 134 patients were included in the study. Nutritional supplement therapy (93%) and nutrition prescription (91%) were the most common interventions. Interventions targeting meals and snacks (43%) and meal support (40%), were documented less frequently. Fasting (93%) and gastrointestinal symptoms (49%) were the most common barriers.

Conclusions:

Although most patients had oral intake, vitamin and mineral supplementation, along with medical nutritional therapy, were more commonly documented than meal/snack interventions and meal support. The lack of dietary interventions, whether unperformed or undocumented, needs further investigation. The frequent documentation of barriers to nutritional interventions indicates that patients recovering from burn injuries may be at risk of insufficient energy and protein intake. Therefore, it is essential to prioritize and document nutritional therapy in post-burn care, regardless of the severity of the injury.

P263

Up to date on metabolic disorders and nutritional requirements in critically injured burn patients.

Moreno, D. ^1^, Izquierdo Granadero, B., Castro Sevilla, A., Armendariz Solera, E., **Sinovas Rodriguez, M.**

^1^ OSAKIDETZA, Bilbao, Spain

Poster presentations 3, 4 September 2025, 15:15–15:45

Aim:

Review the current state of knowledge regarding metabolic alterations and nutritional strategies in critically ill burn patients.

Methods:

Bibliographic research was conducted using PubMed, which includes the MEDLINE database and other sources, using the terms “Metabolism”, “Nutrition”, “Nutritional Therapy”, and “Burn Patient”. Search strategy included Review articles in English and published within the last 10 years. After selection to obtain the most up-to-date and high-quality information, a total of 18 articles were included in this revision.

Results:

Severe burns cause significant oxidative stress, a systemic inflammatory response, and a state of hypermetabolism and hypercatabolism that can persist for months. This process is associated with sarcopenia, organ dysfunction, sepsis, and increased mortality. Energy depletion, negative protein balance, and antioxidant micronutrient deficiency during burn injury are linked to unfavorable clinical outcomes.

Therefore, personalized nutritional therapy is recommended, prioritizing early enteral nutrition from the beginning of the resuscitation phase. An optimized caloric and protein intake is recommended, with a focus on the administration of antioxidant micronutrients and glutamine, which have shown benefits in recovery. Additionally, pharmacological strategies such as propranolol and oxandrolone have been explored, demonstrating positive effects in reducing catabolism and improving anabolism.

Conclusions:

Nutritional management in critically injured burn patients is essential to improve their prognosis. Early enteral nutrition, adequate micronutrient supplementation, and the use of pharmacological strategies are pillars of treatment for these patients, contributing to improved recovery and reducing complications.

P264

The Burn Repair Molecule? Evaluating FGF-21 in Thermal Injury—A Systematic Review

**Niederegger, T.** ^1^, Brandt, J. ^1^, Schaschinger, T. ^1^, Knoedler, L. ^2^, Knoedler, S. ^3^, Palackic, A. ^4^, Panayi, A. ^2,4^, Hundeshagen, G. ^4^

^1^ University of Heidelberg, Medical Faculty, Heidelberg, Germany, ^2^ Charité—Universitätsmedizin Berlin, corporate member of Freie Universität Berlin, Humboldt-Universität zu Berlin, and Berlin Institute of Health, Department of Oral and Maxillofacial Surgery, Berlin, Germany, ^3^ Department of Surgery, Division of Plastic Surgery, Yale School of Medicine, New Haven, CT, USA, ^4^ Department of Hand, Plastic and Reconstructive Surgery, Burn Center, BG Trauma Center Ludwigshafen, University of Heidelberg, Ludwigshafen, Germany.

Poster presentations 3, 4 September 2025, 15:15–15:45

Aim:

This systematic review investigates the role of Fibroblast Growth Factor 21 (FGF21) in burn injury by synthesizing clinical and experimental evidence on its metabolic, inflammatory, and mitochondrial effects, and explores its potential future application as a biomarker and therapeutic target in burn care.

Methods:

A systematic review was conducted following PRISMA 2020 guidelines, with literature searches across PubMed, EMBASE, Web of Science, Cochrane, and Google Scholar through March 2025. Studies investigating FGF21 in the context of burn injury were included, spanning in vivo, in vitro, and clinical research. Risk of bias was assessed using the Newcastle-Ottawa Scale and SYRCLE tool, with evidence graded per Oxford Levels of Evidence.

Results:

Seven studies from 2015 to 2024 were included. FGF21 was consistently elevated post-burn and linked to white adipose tissue browning, hypermetabolism, mitochondrial stress, and systemic inflammation. Nutritional interventions reduced circulating FGF21 and improved wound healing in human trials. Exogenous FGF21 therapy enhanced angiogenesis, epithelial regeneration, and inflammation resolution in diabetic burn models. However, prolonged endogenous FGF21 elevation was associated with adverse metabolic effects, suggesting context-dependent roles.

Conclusions:

FGF21 plays a dual role in burn injury—as a biomarker of systemic stress and a modulator of repair. While preclinical data support its therapeutic potential, clinical translation requires phase-specific, personalized strategies. Future interdisciplinary research should clarify FGF21’s timing, delivery, and long-term impact in burn care.

P300

Clinical Outcomes of Early Burn Wound Cooling in Scald Burns: A Retrospective Analysis

**Aydogan, C.** ^1^, Erkent, M. ^2^, Abalı, A. ^1^, Kuru, E. ^3^, Gojajev, A. ^2^, Avcı, T. ^4^, Aydın, H. ^2^, Haberal, M. ^1^

^1^ Baskent University Burn Center, Ankara, Türkiye, ^2^ Baskent University Department of General Surgery, Ankara, Türkiye, ^3^ Baskent University School of Medicine, Ankara, Türkiye, ^4^ Baskent University Turgut Noyan Trainig and Research Center, General Surgery, Adana, Türkiye.

Poster presentations 4, 5 September 2025, 10:00–10:30

Aim:

This study compared outcomes among scald-burn patients who received proper burn-wound cooling (running tap water and/or Burnshield^®^, Johannesburg, South Africa) within eight hours versus those who received incorrect or no first aid, focusing on healing times, surgical intervention, antibiotic use, and hospitalization.

Methods:

We retrospectively analyzed 2566 scald-burn patients. Proper first aid entailed immediate application of running water and/or Burnshield^®^, while incorrect first aid included methods like ice or toothpaste. Patients who received neither were classified as having no first aid. We assessed time to complete epithelialization, rates of debridement and grafting, antibiotic prescriptions, and hospital admissions.

Results:

The cohort’s mean age was 29.9 years, with 68% female. Of these, 72% received proper first aid, 18% received incorrect aid, and 9% received none. Correctly cooled wounds healed faster (11 days) compared to the other groups (about 18–19 days), with lower surgical intervention rates, fewer antibiotic prescriptions, and decreased hospital admissions. Subgroup analyses (pediatric, adult, geriatric) confirmed the protective effect of early wound cooling.

Conclusions:

Early application of cool running water or Burnshield^®^ significantly improves clinical outcomes in scald burns. Education on timely burn management can reduce morbidity, highlighting the importance of proper first aid for caregivers, patients, and healthcare professionals.

P301

Genital and Perineal Burns Over 10 Years: A Retrospective Multicenter Analysis

**Aydogan, C.** ^1^, Erkent, M. ^2^, Abalı, A. ^1^, Gojajev, A. ^2^, Avcı, T. ^3^, Kuru, E. ^4^, Aydogan, H. ^4^, Haberal, M. ^1^

^1^ Baskent University Burn Center, Ankara, Türkiye, ^2^ Baskent University Department of General Surgery, Ankara, Türkiye, ^3^ Baskent University Turgut Noyan Trainig and Research Center, General Surgery, Adana, Türkiye, ^4^ Baskent University School of Medicine, Ankara, Türkiye.

Poster presentations 4, 5 September 2025, 10:00–10:30

Aim:

To evaluate demographic and clinical outcomes in patients with genital/perineal burns over 10 years at three burn centers, focusing on etiology, total burn surface area (TBSA), length of stay (LOS), catheter use, urinary tract infections (UTIs), and mortality.

Methods:

We examined 7200 burn patients from January 2013 to January 2025 across three Baskent University Burn Centers, of whom 1791 were hospitalized and 441 (6%) had genital/perineal burns. Data included demographics, burn cause, TBSA, burn depth, catheter use, UTI incidence, treatment (dressings or grafting), LOS, and discharge status. Statistical analyses used Fisher’s exact test and the Mann–Whitney U-test. Institutional ethics approval was obtained, and data were de-identified.

Results:

Of 441 patients, 62% were male. Most burns were scald-related (53.9%), followed by flame. Patients with TBSA <5% typically had superficial-to-mid dermal burns and a median LOS under 10 days, whereas those with TBSA >5% had more extensive injuries and a median LOS of 20 days. Catheters were more frequently used when TBSA >5% or LOS exceeded 10 days, with UTIs in 50% of patients hospitalized longer than 10 days. Dressings sufficed for 85.6% of second-degree burns, while grafting was reserved for deeper wounds. Mortality was 24% overall, rising to 47.8% for flame burns.

Conclusions:

Although uncommon, genital/perineal burns pose risks of infection, prolonged hospitalization, and higher mortality, especially in flame-related and extensive injuries. Prompt care, standardized protocols, and prevention strategies are crucial, particularly for pediatric scalds.

P302

Understanding burn severity and its impact on hospitalization in adult patients

**Bartkova, J.** ^1,2^, Petrásšková, L. ^2^, Selecká, D. ^3^, Kotradyová, M. ^2^, Rucki, J. ^2^, Cederna, P. ^4^

^1^ Department of Burns and Plastic Surgery, The University Hospital, Brno, Czech Republic, ^2^ Faculty of Medicine, Masaryk University, Brno, Czech Republic, ^3^ Department of Dermatovenerology, Šumperk Hospital, Šumperk, Šumperk, Czech Republic, ^4^ Department of Surgery, Section of Plastic Surgery, University of Michigan Health System, Ann Arbor, Michigan, USA.

Poster presentations 4, 5 September 2025, 10:00–10:30

This study examines epidemiological trends, treatment approaches, and key predictors of hospitalization outcomes in adult burn patients.

Methods:

A retrospective study was conducted on adult burn patients admitted to a single Burn Unit from 2016 to 2022. Data analyzed included demographics, burn severity, surgical interventions, length of hospitalization, intensive care unit (ICU) admissions, and factors associated with prolonged hospital stay.

Results:

A total of 1378 adults met the study criteria, with 37.3% from rural areas. Among them, 58.2% were transported to the burn center on the day of injury. Self-transport (39.2%) was the most frequent mode of arrival, followed by emergency medical services (31.5%) and inter-hospital transfers (18.9%). Air rescue services were least used (10.3%). Third-degree burns were present in 74.75% of patients. Inhalation injury occurred in 4.28% of cases. The average total body surface area (TBSA) affected was 7.11% ± 8.98% (range: 0–88%). Surgical intervention was required in 62.0% of patients, with 21.59% needing immediate surgery. The average hospital stay was 16.64 ± 14.24 days (IQR 7–22). Factors prolonging hospitalization included age (*p* < 0.001), TBSA (*p* < 0.001), higher Abbreviated Burn Severity Index (ABSI) (*p* < 0.001), and type II diabetes mellitus (*p* = 0.003). ICU admission was required in 19.96% of patients, with a mean stay of 21.01 ± 17.67 days. Wound infections affected 77.79% of patients, underscoring the need for effective infection prevention strategies.

Conclusions:

These findings highlight the importance of early risk stratification, optimized infection control measures, and tailored treatment approaches to improve patient outcomes.

P303

The influence of burns during pregnancy on maternal and fetal outcomes—Assessment with real-world data.

**Kleinhapl, J.** ^1,2^, Dempsey, J. ^1^, Golovko, G. ^3^, Gainski, L. ^1^, Ekale, J. ^1^, McAuley, R. ^1^, Ji, R. ^1^, Branski, L. ^1,4^, Finnerty, C. ^1^, Wolf, S. ^1^

^1^ Department of Surgery, University of Texas Medical Branch, Galveston, United States, ^2^ Division of Plastic, Aesthetic and Reconstructive Surgery, Department of Surgery, Medical University of Graz, Graz, Austria, ^3^ Department of Pharmacology and Toxicology, University of Texas Medical Branch, Galveston, United States, ^4^ Shriners Children’s Texas, Galveston, United States.

Poster presentations 4, 5 September 2025, 10:00–10:30

Aim:

Burns trigger long-term challenges such as hypermetabolism, systemic inflammation and immune dysfunction. While treatment is generally complex, management of burned pregnant women requires special consideration of fetal demands. We aimed to identify the effects of burns on maternal and fetal outcomes during pregnancy.

Methods:

We performed a retrospective study in the real-world database TriNetX and compared pregnant women with burn during pregnancy (ICD-10 codes T31 for burns) to unburned controls. Mortality, clinical outcomes and burn extent were assessed. Statistical analysis and propensity score matching were done with TriNetX, significance was set at *p* < 0.05.

Results:

4614 patients with burn during pregnancy were identified (mean age of 36 ± 9 years). 96.79% (*n* = 4466/4614) of them had <20% TBSA (total body surface area) burned, 2.04% (*n* = 94/4614) 20–39% TBSA, and 1.17% (54/4614) >39% TBSA. Burned pregnant women had a significantly higher risk for pregnancy loss (*p* = 0.0047), pneumonia (*p* = 0.0001), thrombosis (*p* = 0.0043) and gestational diabetes (*p* = 0.0441). Furthermore, psychological outcomes such as PTSD, anxiety and postpartum depression were significantly higher (*p* < 0.0001) in burned vs unburned pregnancies.

Conclusions:

Our results indicate that pregnant women who suffer a burn are at higher risk for fetal mortality. Furthermore, pregnancy in combination with burns seems to be associated with a higher risk for both maternal and fetal complications. The increased risk for pneumonia and thrombosis can be an indicator for treatment challenges. These results highlight the need for focussed research on this highly specialised field of burn care to reduce fetal mortality and optimise clinical outcomes.

P304

A 30-year experience treating electrical burns in the helsinki burn centrepo

Korkiamäki, A. ^1^, Kinnunen, E. ^1^, **Lindford, A.** ^1^, Vuola, J. ^1^

^1^ Helsinki University Hospital and University of Helsinki, Helsinki, Finland

Poster presentations 4, 5 September 2025, 10:00–10:30

Aim:

To review the epidemiology and surgical outcomes of electrical burns treated in the National Burn Centre during a 30-year period.

Methods:

This retrospective study analyzed patients treated for electrical burns between 1993 and 2022. Epidemiological and surgical outcome data were collected. In addition, patients who died at the scene were retrieved from the Finnish Safety and Chemicals Agency.

Results:

The study included 133 patients with an average of 4.4 electrical injuries per year (range: 1–10). 84 individuals died from electrical injuries at the scene in Finland during the same period. The median age of the study patients was 28 years (range: 1–68) and most were male (*n* = 120, 90.2%). Occupational injuries accounted for the majority (*n* = 74, 55.6%). 60.9% (*n* = 81) of the injuries were high-voltage injuries. The incidence of low-voltage injuries decreased during the study period. The mean burn size was 12.3% of the total body surface area (range: 0–93). 63 patients (47.4%) required surgical intervention, with skin grafting being the most common procedure (*n* = 57, 42.9%). Major amputation was required for 6 (4.5%) patients. Three patients (2.3%) died in hospital. The median length of stay in the Burn Centre was 7 days (range: 1–141).

Conclusions:

Electrical burns predominantly affect young men of working age. The severity of these injuries ranged from minor burns to severe, life-threatening burns requiring extensive surgical intervention. These injuries can have a significant impact on the individual as well as on the years of working life lost.

P305

The Impact of Burn Injuries on Subsequent Cardiovascular Morbidity and Mortality

**Lipovy, B.** ^1^, Krbcova Moudra, V. ^2^, Trinh, M. ^2^, Mensikova, K. ^3^, Jarkovsky, J. ^3,4^, Tresnerova, I. ^5^, Stonova, C. ^1^, Tousek, P. ^2^, Zajicek, R. ^1^

^1^ Department of Burns Medicine, Third Faculty of Medicine, Charles University and University Hospital Kralovske Vinohrady, Prague, Czech Republic, ^2^ Department of Cardiology, Third Faculty of Medicine, Charles University and University Hospital Kralovske Vinohrady, Prague, Czech Republic, ^3^ Institute of Health Information and Statistics of the Czech Republic, Brno, Czech Republic, ^4^ Institute of Biostatistics and Analyses of the Faculty of Medicine of the Masaryk Universit, Brno, Czech Republic, ^5^ Department of Burns and Plastic Surgery, Institution Shared with University Hospital Brno, Faculty of Medicine, Masaryk University, Brno, Czech Republic.

Poster presentations 4, 5 September 2025, 10:00–10:30

Aim:

The objective of this study was to investigate the association between burn trauma and long-term adverse cardiovascular (CV) outcomes, including mortality and cause-specific rehospitalization.

Methods:

This study evaluated a Czech Republic Burn Center cohort (2000–2015) using national health data. Patients were stratified by age (<35, 35–50, ≥50 years) and burn severity (TBSA: <10%, 10–19%, 20–39%, ≥40%). Outcomes included all-cause and CV mortality (Kaplan-Meier analysis) and CV-related hospitalizations, expressed in person-years.

Results:

This cohort included 7048 patients with a mean follow-up of 13.2 years. Overall, 1582 (22.4%) died, with circulatory diseases being the leading cause (*n* = 580, 36.7%). The predominant CV deaths were chronic ischemic heart disease (*n* = 204, 35.2%). CV mortality was similar across burn size groups (<10% *n* = 361, 41.5%; 10–20% *n* = 146, 36.0%; 20–40% *n* = 64, 35.2%), but lowest in severe burns (≥40% TBSA, *n* = 9, 7.1%). Mortality increased with age, peaking in patients >50 years (*n* = 497, 85.7%).

We recorded 3006 CV-related hospitalizations. Hospitalization risk increased with age (35–50y: 3.5x higher; >50y: 14x higher vs. <35y, both *p* < 0.001) but was unaffected by burn size (<40% TBSA). Notably, severe burns (≥40% TBSA) showed significantly lower CV rehospitalization rates (RR 0.60, 95% CI 0.48–0.75, *p* < 0.001) compared to those with <10% TBSA burns.

Conclusions:

CV diseases were the leading age-dependent cause of death post-burn. Neither CV mortality nor rehospitalization correlated with burn size, likely due to competing risks from trauma-related deaths. Burn injury itself did not appear to increase CV rehospitalization risk.

P306

Severe Burn Injury and Its Impact on Long-Term Cardiovascular Health

**Bakalar, B.** ^1,2^, Krbcova Moudra, V. ^3^, Trinh, M. ^3^, Malikova, H. ^4^, Weichet, J. ^4^, Stonova, C. ^2^, Zajicek, R. ^2^, Tousek, P. ^3^

^1^ Department of Anesthesia and Intensive Care Medicine, Third Medical Faculty, Charles University and FN Kralovske Vinohrady University Hospital in Prague, Prague, Czech Republic, ^2^ Department of Burns Medicine, Third Faculty of Medicine, Charles University and University Hospital Kralovske Vinohrady, Prague, Czech Republic, ^3^ Department of Cardiology, Third Faculty of Medicine, Charles University and University Hospital Kralovske Vinohrady, Prague, Czech Republic, ^4^ Department of Radiology and Nuclear Medicine, Third Faculty of Medicine, Charles University and University Hospital Kralovske Vinohrady, Prague, Czech Republic.

Poster presentations 4, 5 September 2025, 10:00–10:30

Aim:

This study aimed to evaluate cardiovascular outcomes in patients with severe burn injuries two years after hospital discharge.

Methods:

This prospective cohort study included adult patients with burns >30% of total body surface area (TBSA) and no history of cardiovascular disease hospitalized during the period from 12/2017 to 02/2021. The patients were followed at one year and two years after discharge. Follow-ups included laboratory tests and cardiac magnetic resonance imaging (MRI). In addition, a cardiac MRI was performed on the control group (healthy volunteers).

Results:

This study included 16 patients (1 female, 15 males) with a mean burn area of 47 ± 14.7% TBSA, age 44.2 ± 12.7 years, and hospitalization lasting 100 days. Common cardiovascular events during acute care were heart failure and hypertension (37.5% each), followed by arrhythmia (18.8%).

High-sensitive troponin levels remained stable post-discharge (discharge: 10.8 ± 13.2 ng/L; follow-ups: ~4.7 ng/L). NT-proBNP decreased significantly by the second follow-up (82.6 ± 61.4 vs. 60.1 ± 94.7 ng/L, *p* = 0.033). Cardiac magnetic resonance imaging revealed no ejection fraction changes (follow-ups: ~57%; vs. controls: ~60.5%, *p* = NS). Myocardial fibrosis (Late gadolinium enhancement) was present in 25% of patients.

Conclusions:

Data indicate severe burn patients exhibit elevated myocardial stress (high NT-proBNP at discharge) that significantly decreases over two years. While ejection fraction remained normal (vs controls and over time), the presence of myocardial fibrosis (25% of cases) suggests burns may cause reduced ventricular compliance, potentially leading to heart failure with preserved ejection fraction.

P307

Ambulance calls for burns in Poland—6-year retrospective analysis of 37,596 interventions

**Marzec, L.** ^1^

^1^ Kikgel, Ujazd, Poland, ^2^ Polish Association for the Study of Pain, Krakow, Poland

Poster presentations 4, 5 September 2025, 10:00–10:30

Aim:

The aim of this study was to analyze the epidemiological analysis of ambulance interventions for burns in Poland from 2018 to 2023. Methods:

Data from ambulance medical records obtained from the National Emergency Coordination Center on burn calls from 2018–2023 were analyzed retrospectively. The calls were analyzed by month of the year, time of day, province, and age and gender of the victims.

Results:

There were 37,596 ambulance calls for burns in study period, an average of 6266 per year (Me = 6146). The highest number of calls was shown in 2018 (18.83%), in January (9.19%), on Saturdays (16.36%), at 7 p.m. (7.22%), at home (77.69%), and in the Mazowieckie province (14.73%). Among the victims whose age was recorded (97.87%), adults (63.65%) and men (56.12%) predominated, as well as those with thermal burns. Conclusions: Burns continue to be a challenge for public health and emergency services. Among those with burns, there is a preponderance of adults who most often suffer thermal burns at home on days off and in the province with the highest population density.

P310

Management of Full Thickness Burn Wounds in a Canine Model

Utilizing Acellular Fish Dermis and Autologous Sprayed Skin Cells

**Cordova, A.** ^1^, Trethan, N. ^2^, Shihadeh, S. ^1^, Young, V. ^1^

^1^ Sarasota Memorial Hospital, Sarasota, United States, ^2^ Oakdale Veterinary Group, Oakdale, United States.

Poster presentations 4, 5 September 2025, 10:00–10:30

Aim:

Full thickness skin burn wounds may be challenging to treat, particularly on animals. Dermal matrices may enhance the development of an optimal vascularized wound bed for grafting and provide temporary wound coverage. Decellularized and lyophilized north Atlantic cod fish dermis have putative wound healing properties. Subsequent resurfacing with autologous split-thickness skin graft (STSG) and autologous sprayed skin cell transplantation (ASSCT) may lead to faster and complete healing of the skin grafts.

Methods:

A 6-year old neutered male dog, boxer, experienced a full-thickness skin burn while undergoing mass removal and dental procedure. Iatrogenic injuries to bilateral lower extremities affecting 10%TBSA were sustained from warm air device while undergoing general anesthesia. Once demarcated, all the burn wounds were tangentially excised. Subsequently, the wounds were resurfaced with fish dermis and negative pressure wound therapy was applied. 14-days later, when the wound beds appeared optimal for grafting, autologous STSG was performed, using punch biopsies, in addition to ASSCT.

Results:

Xenograft integration and a vascularized wound bed was evidenced in >95% surface area within 14-days post-grafts application. This was considered optimal for resurfacing. Skin coverage with a STSG and ASSCT revealed nearly 100% skin graft take and epithelization within 14 days.

Conclusions:

Decellularized and lyophilized fish dermis provide excellent wound coverage and enhances the formation of an optimal bed for grafting on a canine burn patient. Subsequent, ASSCT reduces time of healing. Further animal studies may be performed to reproduce these results to further validate in human beings for the treatment of full-thickness burns.

P311

Lyophilized and Acellular Fish Dermis for the Management of Partial and Full Thickness Friction Skin Burns.

**Cordova, A.** ^1^, Shihadeh, S. ^1^, Young, V. ^1^, Selembo, T. ^1^

^1^ Sarasota Memorial Hospital, Sarasota, United States

Poster presentations 4, 5 September 2025, 10:00–10:30

Aim:

Friction skin burns occur as a result of thermal injuries when skin rubs against abrasive surfaces scraping/wearing it away. This commonly occur on surfaces such as carpets and asphalt/concrete. In motor vehicle accidents, most frequently occurs to motorcyclist. Skin substitutes may act as a biological dressing and reduce pain in the setting of a partial thickness injury. While in full thickness injuries, it may provide temporary wound coverage and enhance a vascularized wound bed for grafting.

Methods:

Two-patients sustained partial and full thickness friction skin burns and underwent debridement and management with decellularized fish skin. A 40-year-old female, cyclist struck by a car and dragged under the vehicle. She sustained multiple orthopedic injuries and extensive friction burns. Second patient, a 24-year-old male motorcyclist involved in a crash. Both underwent tangential excision, Xenografted application, and subsequently autologous STSG and sprayed suspended-cell transplantation.

Results:

In partial thickness skin injuries, complete Xenograft incorporation and 100% epithelization and wound healing was noted within 2-weeks. In full thickness, integration and optimal granulation tissue evidenced in >95% of surface area as early as 5-days after application. This was considered ideal for resurfacing. Skin coverage with meshed STSG revealed nearly 100% skin graft-take and epithelization within 2 weeks.

Conclusions:

Decellularized fish dermis provide excellent wound coverage of full thickness friction burn wounds and enhances the formation of the optimal wound bed for skin grafting. For partial thickness wounds, it also provided significant pain control and complete wound healing and re-epithelialization. Follow-up may determine the long-term outcome.

P312

Combined Use of Polylactic Acid-Based Membranes and Matrices for the Management of Mixed-Depth Burn Injuries: A Case Seriespo.

**Cordova-Orrillo, J.** ^1^, Lobaton -Rosas, R. ^1^, Lobaton-Rosas, L. ^1^, Perez-Sovero, R. ^1^, Angulo-Bazan, R. ^1^

^1^ Auna Hospitals, Lima, Peru

Poster presentations 4, 5 September 2025, 10:00–10:30

Aim:

This case series describes the outcomes of a depth-stratified treatment approach using two polylactic acid (PLA)-based biomaterials—a membrane for partial-thickness burns and a matrix for deep partial- to full-thickness burns—in patients with mixed-depth thermal injuries.

Methods:

Three adult patients, aged 30 to 40 years, sustained flash burns involving 16% to 63% of total body surface area, including both second- and third-degree injuries. Following early hydrodebridement, partial-thickness burns were managed with PLA membranes, while full-thickness injuries were covered using PLA matrices. Treated areas were dressed with paraffin gauze and left undisturbed for two to three weeks to preserve the bioactivity of the materials. Clinical endpoints included epithelialization, granulation tissue development, graft uptake, and time to wound closure.

Results:

In all cases, partial-thickness burns treated with the PLA membrane achieved complete epithelialization without needing autografts. Full-thickness areas covered with the PLA matrices developed neodermis that supported subsequent graft integration. Wound inspection at 10 to 28 days revealed stable dressing adherence, progressive healing, and absence of infection or dressing-related complications. Notably, facial and upper extremity burns healed with minimal contracture or scarring.

Conclusions:

The combined use of PLA membranes and matrices, selected according to burn depth, provides a biologically rational and effective strategy for managing complex burns. This dual approach supports spontaneous healing in partial-thickness burns and prepares full-thickness wounds for grafting. The shared lactate-releasing profile modulates inflammation, pH, and cellular recruitment, which together foster favorable clinical outcomes. Prospective studies are warranted to validate this depth-adapted approach in broader patient populations.

P313

Results of Modified Meek Micrografting Combined with Dermal Matrices for Extensive Burns

**Cordova-Orrillo, J.** ^1^

^1^ Auna Hospitals, Lima, Peru

Poster presentations 4, 5 September 2025, 10:00–10:30

Aim:

To evaluate the clinical outcomes of modified Meek micrografting combined with dermal matrices for extensive burns.

Method:

A retrospective analysis was conducted on 10 adult patients (age range 18–65) with full-thickness burns treated between 2021–2023. Selection criteria included: full-thickness burns with clean wounds, free of devascularized tissue.

Of the 10 patients, 4 received a dermal matrix combining glycosaminoglycan, collagen, and elastin, while the remaining 6 received a dermal matrix using glycosaminoglycan and collagen alone, both with the modified Meek micrografting technique. Primary outcomes assessed included healing time, number of interventions, and graft integration rates.

Results/Discussion:

Among the 10 patients, 60% were males, and 40% had more than 60% TBSA. Thermal burns accounted for 70% of cases, followed by chemical (20%) and electrical injuries (10%).

The combined matrices approach required four interventions over 28 days, while the collagen/elastin matrix alone (6 patients) required single-stage autografting. Graft integration rates averaged 95%, with one outlier achieving 30% integration, requiring successful regrafting. Remaining open areas needed only conservative wound care. The technique was valuable in cases with limited donor sites, maximizing skin expansion while maintaining graft quality.

Conclusions:

The integration of modified Meek micrografting with dermal matrices represents an effective approach for extensive burn management, achieving high graft integration rates. This technique should be considered for large TBSA burns with limited donor availability.

Key words: meek micrograft, burns, dermal matrices

P314

P315

Extensive Enzymatic Debridement with Nexobrid^®^ in a Critically Burned Patient: A Case Report

**Costa, A.** ^1^

^1^ Azienda Ospedale Università Di Padova, Padova, Italy

Poster presentations 4, 5 September 2025, 10:00–10:30

Aim:

To describe early extensive enzymatic debridement with Nexobrid^®^ in a patient with major burns and critical illness.

Methods:

A 28-year-old obese male (110 kg, 185 cm) sustained 70% TBSA burns from a gasoline container explosion, with associated inhalation injury. Intubated on scene, he received Nexobrid^®^ 4 h post-trauma (25% TBSA, circumferential areas). He was admitted to ICU for ventilatory support. A second enzymatic debridement (25% TBSA) was completed at 28 h. Forty-eight hours later, 10,000 cm^2^ of cryopreserved allograft was applied. Wounds were managed with hydrogel and non-adherent gauze.

Results:

Debridement was completed by day 2, exceeding EMA’s 15% TBSA/session guideline. No surgical escharotomy was required. Close ICU supervision enabled safe and extended enzymatic treatment in the presence of inhalation injury. Although initial caution in ventilated patients has been suggested, recent evidence supports safety of Nexobrid^®^ even in extensive burns with airway involvement. Hemodynamics remained stable without vasopressors after initial resuscitation. Coagulation was preserved (normal INR, improving antithrombin III), and hemoglobin remained ≥9.9 g/dL in the first week without transfusions. A tracheostomy was performed on day 8. The patient was extubated on day 21 after progressive weaning.

Conclusions:

This case suggests that early extensive enzymatic debridement can replace surgical escharotomy and support circulatory and coagulative stability in high-TBSA burns with inhalation injury. ICU-level monitoring was essential to safely exceed standard use thresholds of Nexobrid^®^.

P316

Functional Outcomes in Adult Patients with Hand Burns: Predictive Factors and the Role of Amniotic Membrane Grafting.

**Costa, A.** ^1^

^1^ Azienda Ospedale Università Di Padova, Padova, Italy

Poster presentations 4, 5 September 2025, 10:00–10:30

Aim:

This study aimed to assess long-term functional outcomes in adult patients with hand burns, focusing on predictive factors of impairment and the potential benefits of amniotic membrane grafting.

Methods:

We conducted a retrospective cohort study on 88 adult patients admitted to a tertiary burn center with unilateral or bilateral hand involvement. Demographic, burn-related, and clinical data were collected. Functional outcomes were evaluated at one year post-injury using the DASH (Disabilities of the Arm, Shoulder, and Hand) score. Non-parametric tests were used for subgroup comparisons, and a multivariate regression model was employed to identify predictors of severe functional impairment.

Results:

Patients with higher TBSA had longer hospital stays; however, neither burn size nor depth independently predicted hand function loss. Instead, anatomical site, particularly digital and bilateral involvement, was strongly associated with higher DASH scores. Older age emerged as an independent predictor of poor functional outcomes. Surgical strategies varied, but the use of amniotic membrane grafts was associated with lower DASH scores, suggesting better functional recovery.

Conclusions:

Anatomical location, especially finger and bilateral hand burns, is a key predictor of long-term functional impairment, outweighing burn depth or TBSA. Age is another significant prognostic factor. Amniotic membrane grafting appears to promote improved tissue healing and functional recovery, supporting its use in tailored early interventions. Multicentric studies and integration of patient-reported outcomes are warranted to validate these findings and guide future rehabilitation protocols.

P317

Cross-Finger and Quaba Flaps: A Case of Finger Circumferential Burn Reconstruction

**Cuadros Ramirez, M.** ^1^, Serracanta i Domenech, J. ^1^, Rivas Nicolls, D. ^1^, Bulla, A. ^1^, Lopez Martinez, J. ^1^, Orois Gomez, S. ^1^, Iglesias Alvarez, A. ^1^, Gonzales Peña, A. ^1^, Barret Nerin, J. ^1^

^1^ Vall D’Hebron University Hospital, Barcelona, Spain

Poster presentations 4, 5 September 2025, 10:00–10:30

Aim:

To describe the surgical management of a circumferential finger burn.

Methods:

A 56-year-old male patient, with no relevant medical history, was admitted to the Vall d’Hebron Burn Unit after sustaining a full-thickness circumferential burn at the proximal phalanx of the ring finger. The burn resulted from excessive heating of the ring while attempting to cut it with an electrical saw.

Following initial assessment, burn debridement was performed, and the defect was covered with a full-thickness skin graft harvested from the groin. However, during follow-up, graft failure was observed, necessitating further debridement and an alternative coverage approach. A Cross-Finger flap was designed from the third finger to cover the volar surface of the ring finger, while a Quaba flap was used for dorsal coverage. The donor site was covered with an inguinal full-thickness skin graft. Two weeks later, the second stage of the Cross-Finger flap was performed. During follow-up, adequate defect coverage was achieved, allowing for early physiotherapy and rehabilitation, although neuropathic pain persisted.

Results:

The combination of the Cross-Finger flap and the Quaba flap, based on the perforator of the dorsal metacarpophalangeal artery, appears to be a valuable reconstructive option for circumferential burns affecting the proximal phalanx.

Conclusions:

Circumferential third-degree burns of the fingers present a significant reconstructive challenge, emphasizing the need for a broad spectrum of surgical techniques to ensure both optimal coverage and functional recovery of the affected extremity.

P318

Tissue engineering as a tool in a novel approach to the comprehensive treatment and management of a deeply and extensively burned patient—Case Report.

**Czerny, K.** ^1^, Łabuś, W. ^1^, Słaboń, A. ^1^, Ziółkowska, K. ^1^

^1^ Stanislaw Sakiel Burn Treatment Center In Siemianowice Slaskie, Siemianowice Slaskie, Poland

Poster presentations 4, 5 September 2025, 10:00–10:30

Aim:

Deep and extensive thermal burns with respiratory burns can be associated with a high mortality rate in elderly patients. To obtain a good quality scar, the use of allogeneic acellular dermal matrices can be proposed as a co-graft for the autologous skin graft. The application of in vitro cultured, autologous keratinocytes and fibroblasts can accelerate the healing process of a burn wound. In addition, allogeneic amnion transplantation can be performed to accelerate healing of donor sites.

Methods:

This is a case report of a 65-year-old patient with thermal burns—thorax, abdomen, back, right shoulder area, including the elbow area, right thigh—third-degree burns 26% TBSA and airway. The patient underwent multistage surgical treatment. After the excision of necrotic tissues a graft of acellular dermal matrix (ADM) and free split-thickness skin graft (STSG), in vitro cultured skin cells and local negative pressure wound therapy (NPWT) were used. An allogeneic amnion graft was used for the donor sites. The donor sites were used several times after healing. Healing progress was monitored using laser speckle contrast analysis. The scar viscoelasticity, transepidermal water loss, scar melanin content, epidermal thickness, temperature and high-frequency ultrasound were examined after healing. Selected skin parameters were also examined using.

Results:

The patient was discharged at day 77 (41 days in the surgical ward and 36 days in the rehabilitation ward) after admission with healed wounds.

Conclusions:

Tissue engineering may be useful tool in elderly patients with deep and extensive thermal burns.

P319

Novel Treatment Strategy for Toxic Epidermal Necrolysis (TEN): Combining Serial Debridement, Polylactic Membrane Dressings, and Water-Filtered Infrared-A Radiation (wIRA).

**Dastagir, N.** ^1^, Obed, D. ^1^, Kijas, D. ^1^, Tamulevicius, M. ^1^, Vogt, P. ^1^, Dastagir, K. ^1^

^1^ Hannover Medical School, Hannover, Germany

Poster presentations 4, 5 September 2025, 10:00–10:30

Aim:

Toxic epidermal necrolysis (TEN) is a life-threatening condition with a mortality rate of approximately 25% to 30%. Early and adequate wound coverage is essential due to extensive skin defects. Previous reports on wound care in TEN patients have described radical debridement of the entire affected body surface prior to the application of modern wound dressings. However, heavy wound secretion in TEN may lead to complications such as hypothermia, hypovolemic shock, wound infection, and subsequent sepsis.

Methods:

This study presents a novel strategy involving serial hydrotherapeutic wound debridement and stepwise application of polylactic membrane (PLM) to affected areas. Water-filtered infrared A light (wIRA) was utilized to maintain dryness over PLM-covered areas. Retrospective data from patients treated with polyhexanide gel (control group 1) and those treated exclusively with PLM (control group 2) were collected for matched-pair analysis. The length of stay in the intensive care unit (ICU) and the need for catecholamines were compared among the three groups.

Results:

By employing serial debridement combined with wIRA treatment and PLM dressings, we significantly reduced catecholamine requirements, minimized risks of hypothermia and infection, and shortened ICU stays compared to the control groups.

Conclusions:

This methodology demonstrates improved outcomes in promoting wound closure and healing for TEN patients and should be considered for integration into the standard of care.

P320

Fish Skin Grafts with Omega-3 in pediatric wound management.

**De Buys Roessingh, A.** ^1^, Poget, M. ^1^

^1^ CHUVaudois, Lausanne, Switzerland

Poster presentations 4, 5 September 2025, 10:00–10:30

Aim:

Wound healing in the paediatric population is known be unique and poorly studied. Split thickness skin grafts and flaps overlap their applications with the growing field of cellular and tissue-based therapies. The aim of the study is to show the utility of Kerecis^®^ Omega3 wound patch to accelerate the granulation process and acts as a scaffold for ingrowth and adhesion of dermal cells and capillaries.

Methods:

We described the application of Kerecis^®^ (Ísafjörður, Iceland) in five patients who were treated in the region of the scalp, the axillary region (2) and the lower extremity (2). Among them, Kerecis^®^ was used as dermal and/or epidermal substitute to cover either a large incised area of a Verneuil disease or a very severe wound injury of a foot.

Results:

The mean time required to achieve complete epithelialization was 28 days after two to three applications of the fish substitute and multi-weekly dressings. The definitive outcome was excellent in all wounds, meaning acceptable retraction, no hypertrophic scar and complete wound coverage.

Conclusions:

Kerecis^®^ is a therapeutic dermal substitute in the treatment of loss of substance in children.

Keywords: wound—dermal substitute-replacement

P322

Single layer dermal regenerative template (Integra): a versatile option for full thickness burns to the hand

**Dorai, A.** ^1^

^1^ Dr. Ananda Dorai, Petaling Jaya, Malaysia

Poster presentations 4, 5 September 2025, 10:00–10:30

Aim:

To monitor the postoperative outcome using the single layer dermal regenerative template (Integra) on full thickness burns to the hand.

Methods:

A retrospective study was conducted on all cases which used the single layer dermal regenerative template (Integra) on full thickness burns to the hand.

Results:

Two patients were identified using the single layer dermal regenerative template (Integra). The first patient was 42 years old with 20% chemical burns. He had the dermal template applied on day 48 of injury to dorsum of both hands. The second patient was 39 years old with 46% flame burns and had the dermal template applied on day 14 of injury to the right dorsum hand. Both patients had the dermal template with split skin grafting done simultaneously and the skin graft was meshed 1:1 using the Humeca skin mesher. Both patients had a good graft take and were monitored for the any contractures. Both patients did not have any revisional surgeries and had good range of movements after 5 years.

Conclusions:

The single layer dermal regenerative template (Integra) is a versatile option for full thickness burns to the hand with good functional outcome.

P324

Skin graft take rate among minor burns—A cohort study to investigate the effect of factors such as burn depth, burn size, and timing of the operation.

**Elmasry, M.** ^1^

^1^ Linköping University Hospital, Linkoping, Sweden

Poster presentations 4, 5 September 2025, 10:00–10:30

Aim:

Early surgical excision and skin grafting has become the most important part of successful healing in burn management, especially in major burns. However, it is not entirely clear whether early excision and skin transplantation has the same advantages in smaller burns and there is no consensus on the timing of skin grafting in this group. The aim was to investigate the effect of timing and other factors for skin graft take rate among minor burns.

Methods:

This retrospective study included patients with thermal injury, a burn size smaller than 11% total body surface area (TBSA), and who were treated with a skin graft operation. Take rate at the second dressing change after operation was used as main outcome, a cut-off of 95% take rate was for the multivariable logistic regression.

Results:

A total of 195 patients were included, median (IQR) age was 42 (9–68) years, 65% were male, and median (IQR) area of deep burns was 2 (1–4)% of the body surface area (BSA). Multivariable regression showed that smaller area of deep burns and scalds (compared with flame and contact burns) were associated with a take rate of ≥95%. Age, timing of the skin graft transplantation, and plasma.

Conclusions:

Our results suggest that the extent and depth of the burn are the most important factors for skin graft take rate among minor burns, while timing of the transplantation is not associated with take rate for the skin graft.

P325

Enzyme Alginogel Adjunctive Treatment for Partial Thickness Burn

**Erdogan, A.** ^1^, Bekki, Y. ^1^, Uçar, A. ^1^, Yıldırım, M. ^1^

^1^ University of Health Science Izmir City Hospital, izmir, Turkey

Poster presentations 4, 5 September 2025, 10:00–10:30

Aim:

Effect of enzyme alginogel adjunctive therapy on the treatment of partial thickness burns

Introduction:

Achieving functional and aesthetically good wound healing in second-degree burns is one of the main goals of burn treatment. Although the existence of numerous medical product alternatives suitable for the characteristics of the patient and the burn injury can be confusing, enzyme alginogel (Flaminal, Flen Pharma, Kontich, Belgium) stands out with its composition that combines different effect properties.

Methodology:

We presented four patients with partial thickness burns. Burn areas were treated by using enzyme alginogel, which has debriding, antimicrobial and non-toxic properties.

Results:

Burn areas left to secondary healing with enzyme alginogel, much better results were obtained in wound healing, functionally and aesthetically, with less pain, burning, stinging and itching sensations than expected.

Conclusions:

With the use of the right medical product in the appropriate patient, good results can be obtained in partial thickness burn areas. Enzyme alginogel, which is a product that the patient can use in outpatient treatment at every stage of wound healing with its debriding effect, anti-microbial and non-toxic effect, is a good medical product alternative.

Applicability to Clinical Practice: Enzyme alginogel, which patients can use on an outpatient basis at any phase of wound healing, is a good treatment alternative that reduces sensations such as pain and itching and provides optimal functional and aesthetic results during the secondary healing process.

P326

Hypernatremia in Severe Burn Patients: Risk Factors and Prognosis

**Amarelle, L.** ^1,2,3^, Acevedo, S. ^3^, Duarte, M. ^3^, Mollo, E. ^3^, Moreira, V. ^3^, Nuñez, M. ^3^, Reinoso, L. ^3^, Aramendi, I. ^1,2,3^

^1^ Centro Nacional de Quemados, Montevideo, Uruguay, ^2^ Centro de Tratamiento Intensivo, Hospital de Clínicas, Montevideo, Uruguay, ^3^ Facultad de Medicina, Universidad de la Republica, Montevideo, Uruguay.

Poster presentations 4, 5 September 2025, 10:00–10:30

Aim:

To evaluate the incidence of hypernatremia, its risk factors, impact, and relationship with clinical severity in severe burn patients.

Methods:

A retrospective, observational, unicenter study was conducted, including 141 severe burn patients with a total body surface area (TBSA) burned greater than 20%, admitted between January 2019 and December 2023.

Results:

The incidence of hypernatremia was 57%, with 37% of cases classified as severe. The average onset occurred at 5.9 ± 11.6 days, with an average duration of 9.3 ± 8 days. Patients with hypernatremia had a higher TBSA than those without (40 ± 14.7% vs. 29 ± 9%), with significantly higher sodium levels on days 8, 9, and 10 in patients with more than 40% TBSA. Additionally, hypernatremia patients showed greater clinical severity, as assessed by the BAUX, BOBI, and ABSI scores. Independent risk factors for hypernatremia included age, TBSA, renal injury, and mechanical ventilation. The crude mortality rate was higher in hypernatremia patients (53.1% vs. 13.3%), but in multivariate analysis, hypernatremia was not independently associated with mortality. Factors independently associated with mortality included TBSA, acute renal injury, need for hemodialysis, and fluid gain >10 L.

Conclusions:

Hypernatremia is common in severe burn patients, particularly in those with higher TBSA, renal failure, and mechanical ventilation. While not independently associated with mortality, it correlates with greater clinical severity. Optimizing electrolyte management and monitoring sodium levels is recommended.

P327

Managing Cardiovascular Instability Consequent to Auto-PEEP in a Patient with Inhalation Injury.

**Arbour, R.** ^1^

^1^ Temple University Health System/Temple University Hospital, Philadelphia, Pennsylvania, United States

Poster presentations 4, 5 September 2025, 10:00–10:30

Aim:

The aim of this case report is to illustrate cardiovascular instability following inhalation injury, patient-ventilator dys-synchrony, auto-PEEP and alveolar hyperinflation affecting right and left-heart preload linking with clinical findings and management.

Methods:

A 46-year-old male experienced superficial burn (less than 6% TBSA) in an enclosed space. Grade-3 inhalation injury was identified, and he was intubated for controlled ventilation. Arterial catheterization and intra-arterial monitoring were initiated with real-time derivation of hemodynamic indices. Crystalloid was administered for hypotension consequent to insensible losses and relative hypovolemia/vasodilitation with pro-inflammatory state. Hypotension and decreased cardiac indices were pronounced in peak inspiration. Evaluating patient-ventilator interface and ventilator graphics identified auto-PEEP (7–10 cm H_2_O). Titration of midazolam/fentanyl, cis-atracurium infusions and adjusting ventilator flow rates resolved ventilator dys-synchrony.

Results:

Resolving patient-ventilator dys-synchrony and auto-PEEP improved hemodynamic instability. Ventilation and mechanism-based care for inhalation injury were down-titrated toward ventilator liberation. This case illustrates fragile cardiovascular state affected by dynamic thoracic pressures in phase with controlled ventilation resulting in alveolar hyperinflation, increased RV afterload and left ventricular diastolic dysfunction. Addressing patient-ventilator dys-synchrony as causation of cardiovascular instability was decisive in management.

Conclusions:

Inhalation injury and pro-inflammatory state/vasodilitation risks patient-ventilator dys-synchrony, auto-PEEP and hypotension. Early recognition of patient-ventilator dys-synchrony and auto-PEEP with detailed evaluation of ventilator graphics identifies causes of cardiovascular instability and treatment opportunities. When patients are encountered as described, evaluating ventilator graphics and hemodynamic indices facilitate rapid identification and management of unstable cardiac state from auto-PEEP causing recovery of supra-normal cardiac indices in inhalation injury.

P328

Managing Profound Respiratory Failure and Concurrent ECMO Candidacy Post Burn Trauma

**Arbour, R.** ^1^

^1^ Temple University Health System/Temple University Hospital, Philadelphia, Pennsylvania, United States

Poster presentations 4, 5 September 2025, 10:00–10:30

Aim:

The aim of this case report is to illustrate respiratory failure following burn trauma, early identification of progressive disease and application of clinical triggers escalating ventilator support, prone positioning and evaluation for extracorporeal support.

Methods:

A 51-year-old male experienced 54% TBSA flame surface burn. He was intubated for airway protection. Burn shock resuscitation was based on TBSA/body weight and titrated for cardiovascular response and urine output. Circumferential edema and compartment syndromes necessitated escharotomies. Anemia/coagulopathy and hypotension necessitated aggressive blood product administration, intravascular volume titration and vasopressor support. Lung compliance and wound care tolerance decreased with ARDS evolution. Sedation/analgesia and neuromuscular blockade facilitated comfort, ventilator synchrony and oxygenation. Before ECMO initiation, prone positioning therapy was initiated.

Results:

During 8 days of prone positioning therapy, clinical/radiographic recovery was evident. Ventilator support was titrated downward with liberation from ventilator/vasopressor support. He was ultimately discharged home. This case illustrates evidence-based interdisciplinary care including non-physiologic ventilation modes and prone positioning for severe respiratory failure.

Conclusions:

Care trajectory following burn trauma varies due to evolving edema, compartment syndrome, therapy complications and proinflammatory state/sepsis causing respiratory failure. Early recognition of evolving respiratory failure can trigger advanced ventilation, prone positioning and ECMO preparation, eliminating delay in extracorporeal support. Interdisciplinary care in these settings can be ultimately successful as a bridge to recovery, facilitating rehab and home discharge. When patients are encountered as described, with either ARDS or severe inhalation injury, non-physiologic ventilation, supportive care and prone positioning therapy facilitate recovery with backup ECMO support.

P329

Identifying Probable Intracranial Compartment Syndrome Following Significant Cutaneous Burn Injury.

**Arbour, R.** ^1^

^1^ Temple University Health System/Temple University Hospital, Philadelphia, Pennsylvania, United States

Poster presentations 4, 5 September 2025, 10:00–10:30

Aim:

The aim of this case report is to illustrate relationships among cardiovascular instability, vasopressor requirements, loss of brainstem reflexes and fixed, dilated pupils with significant TBSA burn and large volume resuscitation indicating probable catastrophic intracranial compartment syndrome.

Methods:

A 35-year-old male experienced widespread mixed-thickness burns (greater than 85% TBSA). He was intubated for controlled ventilation and intra-arterial monitoring guided resuscitation. Large-volume crystalloid was administered for resuscitation, hypotension from insensible losses, relative hypovolemia/vasodilitation and vascular endotheliopathy. Escharotomies facilitated ventilation and tissue perfusion. On ICU day-3, a flaccid areflexic neurological exam with pupil fixed, dilated and midpoint with marked vasopressor-dependent cardiovascular instability was evident. Drug therapies were insufficient to mimic loss of brainstem function. Evaluating loss of brainstem function with cardiovascular instability can potentially identify intracranial compartment syndrome and brain herniation.

Results:

Blood pressure was supported with escalating vasopressors dosing and volume management. Appropriate communication was maintained with family regarding goals of care and anticipated mortality/prognosis. On ICU day-4 family elected to transition to comfort-directed care and natural death.

Conclusions:

Significant (greater than 85% TBSA) flame burn causes vasodilatation, inflammation, vascular endotheliopathy and widespread edema even in non-burned body areas. Central and peripheral compartment syndromes are identified by ventilator graphics, abdominal pressure monitoring and physical assessment. Intracranial compartment syndromes causing lethal intracranial pressure elevations are evident upon loss of brain/brainstem function and cardiovascular instability. When patients are encountered as described, neurological, cardiovascular evaluation with escalating vasopressor needs may indicate terminal intracranial compartment syndrome, informing care and family communication around end-of-life decisions.

P332

Tracheoesophageal fistula in a burn patient on mechanical ventilation

**Banožić, I.** ^1^, Škunca, A. ^1^, Muratović, A. ^1^, Banić, M. ^1^

^1^ University Hospital Center, Zagreb, Croatia, Vinogradska cesta 29, Zagreb, Croatia

Poster presentations 4, 5 September 2025, 10:00–10:30

Aim:

Tracheoesophageal fistula (TEF) is a life-threatening complication of prolonged mechanical ventilation, occurring in 0.3–3% of patients undergoing prolonged mechanical ventilation. The aim of this study is to present, through a specific case, how TEF appeared and manifested, and how it was medically treated.

Method:

A 39-year-old female, without comorbidities, was admitted to the intensive care unit due to second and third-degree burns on the head, neck, torso, and all extremities (TBSA 70%) which occurred while igniting a gas stove. Upon admission, she was intubated and mechanically ventilated. She underwent a tracheotomy on the 13th day after intubation. She was on mechanical ventilation for a total of 45 days with an inflated cuff. Two months after the injury, the patient was weaned off mechanical ventilation, and a TEF was observed because the patient developed a cough during feeding. As there were no signs of spontaneous healing, surgical intervention was indicated, resulting in successful surgical treatment.

Results:

Tracheoesophageal fistula is a result of prolonged mechanical ventilation and hyper-inflated endotracheal tube cuff. Though less common than other mechanical ventilation complications, its high morbidity and mortality make it a significant clinical concern. The incidence decreased after the use of low-pressure and high-volume endotracheal cuffs.

Conclusions:

Understanding TEF pathophysiology—primarily pressure-induced ischemic damage to the tracheal mucosa—is essential for implementing effective prevention strategies. Prevention is key, emphasizing correct cuff pressure, regular monitoring, and new technologies to minimize mucosal damage. As ventilation technology improves, protecting the tracheal lining can help prevent this complication.

P333

Evaluation of coagulopathy in burn patients using the QUANTRA^®^ viscoelastic system: TROMBURNT study.

**Bayo Montoliu, M.** ^1^, García García, M. ^1^, Pérez del Caz, M. ^1^, Guijarro Pérez, C. ^1^, Ferre Colomer, M. ^2^, Vidal Peraire, A. ^2^

^1^ Plastic and Reconstructive Surgery Department, Hospital Universitari I Politècnic La Fe, Valencia, Spain, ^2^ Anesthesiology and Resuscitation Department, Hospital Universitari I Politècnic La Fe, Valencia, Spain.

Poster presentations 4, 5 September 2025, 10:00–10:30

Aim:

To analyze whether the QUANTRA^®^ viscoelastic system (HemoSonics, Durham, NC, USA) (a novel technique using ultrasound technology to measure viscoelasticity) provides better assessment of coagulopathy in burn patients, which would allow for optimization in its management.

Methods:

A prospective, observational, single-center study was designed in adult patients admitted to our Burn Unit with TBSA equal to or greater than 15%, susceptible to surgical and/or enzymatic debridement.

Demographic and follow-up data (analytical and QUANTRA) were collected at admission, at 48–72 h (after debridement) and at one week. As outcome variables, transfusion needs, fluid therapy and the presence of complications were recorded.

Statistical analysis of the results at 18 months of study was performed.

Results:

Data were collected from 19 patients admitted between June 2023 and November 2024, with a mean age of 48 years, an average TBSA of 37%, and a mean ABSI score of 8.5.

Clot time (CT), clot stiffness (CS), platelet and fibrinogen contribution to clot stiffness (PCS and FCS) and clot stability to lysis (CSL) were tested.

The data shows a statistically significant tendency for CT to lengthen, as well as CS, PCS and FCS to increase during the hospital stay. CSL remained stable.

Conclusions:

Burn-induced coagulopathy is a poorly understood disorder that appears early in the burn patient’s physiopathology. Quantra^®^ analysis suggests that the initial coagulopathy is less severe and subsequently evolves to a prothrombotic state. This analysis does not correlate with traditional coagulation tests and can therefore be used in guiding blood transfusion needs in burn patients.

P334

Mortality Analysis of ICU Burn Patients in Indonesia’s National Referral Hospital: A 2-Year Retrospective Study.

Wardhana, A. ^1^, **Cristabella, R.** ^1^, Farhana, N. ^1^, Leksono, T. ^1^

^1^ Division of Plastic Reconstructive and Aesthetic Surgery, Dr. Cipto Mangunkusumo Hospital, Jakarta, Indonesia

Poster presentations 4, 5 September 2025, 10:00–10:30

Aim:

To determine whether burn deaths are caused by sepsis, acute respiratory distress, renal failure, sepsis or gastrointestinal bleeding among burn patients admitted to the ICU at Cipto Mangunkusumo Hospital, Jakarta, Indonesia.

Methods:

We retrospectively reviewed 65 patients admitted to the Burn ICU of a tertiary referral hospital in Indonesia between January 2023 and December 2024. Data collected included age, gender, total body surface area (TBSA) burned, burn depth, etiology, timing and type of surgical intervention, length of stay (LOS), complications, and mortality. The Abbreviated Burn Severity.

Index (ABSI) score was calculated for all patients.

Results:

The cohort had a mean age of 40.15 ± 18.24 years and a male-to-female ratio of 2.6:1. Burns exceeding 40% TBSA were predominant (75.4%). Most burns were superficial to full thickness (93.8%), and flame burns accounted for 86.2% of cases. Surgical treatment was performed in 69.2% of patients, with early tangential excision in 55.4%. Nineteen out of 20 non-surgically treated patients died. The mean LOS was 14.34 ± 13.91 days. Complications included sepsis (83.1%), ARDS (40%), kidney failure (15.4%), inhalation injury (9.2%), and GI bleeding (1.5%).

The overall mortality rate was 69.2%.

The mean ABSI score was 9.58 ± 2.60.

Conclusions:

Burn injuries requiring ICU care in our center are predominantly extensive and flame-related, with high mortality and sepsis rates. Sepsis is still the leading cause of death. Whether tangential excision reduces mortality in the ICU burn population has not been studied. Future efforts should focus on early excision, infection control, and intensive critical care protocols.

P335

Management of Patients with Electrical Burns in the Major Burn Unit of Cruces University Hospital: Our Experience over the Last 25 Years.

**Estradera Lainez, J.** ^1^, Martín-Playá, P. ^1^, González-Alaña, I. ^1^, Caramés-Estefanía, J. ^1^

^1^ Cruces University Hospital, Bilbao, Spain

Poster presentations 4, 5 September 2025, 10:00–10:30

Aim:

To analyze the experience and clinical outcomes of patients with electrical burns at Cruces University Hospital.

Methods:

A retrospective, descriptive study including patients admitted to our Burns Unit due to electrical burns between 2000 and 2024 (*n* = 115).

Epidemiological data, burn characteristics and circumstances, management and complications during hospitalization were recorded.

Results:

Electrical burns accounted for 6% of all burns. A decrease in incidence was observed over the last decade. The most frequent injury mechanisms were electrical current passage (40.9%) and electrical flash burns (38.3%), while fulguration was the least common (6.1%). Multiple mechanisms were involved in 14.8% of cases. 23.5% associated secondary trauma.

91.3% of patients were men, with a mean age of 39 years (13–81). Work-related accidents accounted for 61.7% of cases, with high-voltage involvement in 63.5%, affecting an average of 13.7% of the body surface area (1–90%) and mostly deep burns. Among all patients, 27.8% required fasciotomies due to compartment syndrome and 15.7% underwent amputations. The average number of surgeries per patient was 2.

The most common complications were ocular (bilateral keratitis or corneal ulcers), musculoskeletal (regarding bone fractures or muscle necrosis), infectious (burn wound or respiratory infection) and neurological involvement (episode-related amnesia or agitation).

Conclusions:

Electrical burns are uncommon but more severe than other mechanisms, as they tend to cause deeper burns with a high morbidity rate, partly due to the high incidence of amputations and secondary trauma.

A multidisciplinary approach is key in order to reduce severe sequelae associated to electrical burns.

P337

Delayed Spinal Cord Injury Following High-Voltage Electrical Burn: A Case Report

**Iglesias Huerga, J.** ^1^, Yuste Benavente, V., Marino Garcia, M., Páez Ordoño, C.

^1^ Hospital Universitario Miguel Servet, Zaragoza, Spain

Poster presentations 4, 5 September 2025, 10:00–10:30

Aim:

To describe a rare case of delayed spinal cord injury following a high-voltage electrical burn, emphasizing clinical evolution, diagnostic challenges, and management strategies.

Method:

We present the case of a 40-year-old male who sustained deep second- and third-degree burns (13% TBSA) after colliding with a high-voltage power line while paragliding. Initially, he had no neurological deficits, but on day six post-injury, he developed progressive lower limb weakness. Imaging, including MRI, ruled out traumatic spinal cord compression. Neurological and electrophysiological evaluations confirmed a delayed spinal cord injury with predominant motor impairment.

Results:

Despite initial motor function preservation, the patient developed progressive paraparesis, classified as ASIA B. Sensory function remained intact below the lesion level, suggesting primary involvement of the descending motor tracts. Supportive treatment included neuroprotective strategies and early rehabilitation. The patient’s recovery was limited, with persistent motor deficits despite intensive rehabilitation.

Conclusions:

Delayed spinal cord injury following electrical burns is a rare but severe complication. Early recognition, close neurological monitoring, and a multidisciplinary approach are crucial for optimizing functional outcomes. Advanced neuroimaging and experimental therapies may improve future prognoses.

P338

Is hypoalbuminemia an independant risk factor for mortality in severly burned patients (TBSA > 20%)

**Ils, C.** ^1^, Pantet, O. ^2^

^1^ christoph.ils@chuv.ch, Lausanne University Hospital and University of Lausanne, Lausanne, Switzerland, ^2^ olivier.pantet@chuv.ch, Lausanne University Hospital and University of Lausanne, Lausanne, Switzerland

Poster presentations 4, 5 September 2025, 10:00–10:30

Aim:

Determine if hypoalbuminemia is an independent risk factor for mortality and a cut-off value that best predicts mortality in severely burned patient (TBSA > 20%).

Methods:

Retrospective, single center, cohort study of an intensive care burn unit in Switzerland. Patients admitted between January 2006 and December 2023 (648 total admissions) had their files screened for eligibility. Inclusion criteria were patients ≥14 years old and TBSA ≥ 20%. Our exclusion criteria were admission >8 h following accident and transfer to another burn unit or hospital during week 1.

Results:

161 patients eligible. Univariate analysis shows correlation between lower albumin levels and increased risk of mortality (OR 0.91, 95% C.I. 0.86–0.96, *p*-value = 0.001). In multivariate analysis, correcting for absi, there is poor correlation between albumin levels and mortality and results are not statistically significant. (OR 0.99, 95% C.I. 0.93–1.06, *p*-value = 0.87). Using a Youden-Index, the cut-off value for serum albumin that best predicted mortality in our study was 24 g/L (Sensitivity = 80.95%, Specificity = 49.52%). Albumin levels under 24 g/L showed increased mortality (Hazard Ratio 1.72, 95% C.I 0.79–3.73, *p*-value =0.169) using cox regression. The results are not statistically significant (Log rank test: *p*-value = 0.138).

Conclusions:

We could not prove that hypoalbuminemia was an independent risk factor for mortality. Serum albumin level lower than 24 g/L was determined to increase mortality, but the result was not statistically significant. Multicentric studies with more patients needed and reflexion regarding benefit of substituting hypoalbuminemia in burned patients.

P342

Factors associated with fluid overload in the treatment of burn shock.

**Kohn, S.** ^1^, Brun, M. ^2^, Decurnex, M. ^2^, Furest, I. ^2^, Heguy, M. ^2^, Reyes, D. ^2^, Aramendi, I. ^1^

^1^ Physician, Centro Nacional de Quemados, Montevideo, Uruguay, ^2^ Medical Student, Clinical Hospital, University of the Republic, Montevideo, Uruguay.

Poster presentations 4, 5 September 2025, 10:00–10:30

Aim:

We sought to evaluate clinical factors associated with fluid overload and its impact on morbidity and mortality in major burn patients.

Methods:

Retrospective observational study, including all major burn patients admitted to Centro Nacional de Quemados (CENAQUE) between January 2019 and May 2024. Injury, clinical, and outcome characteristics were compared between patients with significant cumulative fluid balance (SCFB) and those with non-significant cumulative fluid balance (NSCFB), both assessed at 48 h from admission. The SCFB cut-off point was set at 17 litres using binary logistic regression for mortality, adjusted for total body surface area burn (TBSA) and weight at admission. Multivariate analysis was performed to identify factors associated with SCFB.

Results:

125 patients were included. The SCFB group (55 patients) had higher %TBSA (*p* < 0.001), ABSI score (*p* = 0.001), and rBAUX score (*p* < 0.001). They were more frequently ventilated at admission (*p* = 0.015) and 48 h after admission (*p* < 0.001). The cumulative doses of morphine (*p* = 0.003) and midazolam (*p* = 0.001) by day 2 were higher, and they required more days on mechanical ventilation (*p* = 0.002). Multivariate analysis revealed an association between SCFB and both higher TBSA and the presence of mechanical ventilation on day 2. The SCFB group experienced a higher frequency of complications, including respiratory failure, acute kidney injury (AKI), need for renal replacement therapy (RRT), and extremity compartment syndrome.

Conclusions:

TBSA and mechanical ventilation were associated with SCFB. Patients with SCFB experienced a higher frequency of complications.

P344

Time of burn surgery and critical care management, a retrospective cohort study of 197 large burned patients.

**Laurent, M.** ^1^, Baudic, T. ^1^, Bouvet, G. ^1^, Rennuit, I. ^1^, Renner, J. ^1^, Hoffmann, C. ^1^, Schaal, J. ^1^, Donat, N. ^1,2^, Leclerc, T. ^1,2^

^1^ Burn Center Unit, Percy Teaching Military Hospital, Clamart, France, ^2^ French Military Health Service, French Military Health Service Academy, Paris, France.

Poster presentations 4, 5 September 2025, 10:00–10:30

Aim:

Large-burned patient care relies on both intensive care medicine and surgery, in which major steps are resection surgery and burn coverage.

We investigated surgical temporality and schedule along with clinical repercussion: is delayed surgery associated with increased length of stay (LOS) and morbidity?

Methods:

We included all patients with 20% of Total Body Surface Area (TBSA). Collected data included: demographics, medical records, burn characteristics, surgical timing (especially surgery delay, end-resection delay and first-coverage delay).

Hospital care efficiency was assessed using ratio LOS/TBSA. Secondary outcome included: in-hospital mortality, organs failure (SOFA), hemodynamic, respiratory or septic complications. TBSA-related subgroups were made: minor (20–40%), moderate (40–60%), large (>60%); and a linear multivariate model was used to assess relation between surgery times and LOS. Statistical analysis was conducted on R.

Results:

From January 2017 to December 2024, 197 large-burned patients were included. Mean age was 44 years (18), median TBSA was 31% [23–44] and in-hospital mortality was 12% (*n* = 23). Median surgery delay and coverage delay were respectively 9 [5–12] and 10 [7–14] days. LOS/TBSA median ratio was 1,31 [1.00–1.79] day per%.

Large surface patients had longer ventilation, replacement therapy and greater SOFA score. In a linear multivariate model, surgical delay, coverage delay and TBSA were all independently associated with LOS.

Conclusions:

In 197 patients with TBSA > 20%, surgical delay was greater than a week. An increase in surgical delay and coverage delay may be associated with longer LOS, independently of TBSA, which may be a concern for cost-efficiency.

P345

Hemodynamic kinetic in severe burn in the acute phase

**Laurent, M.** ^1^, Baudic, T. ^1^, Bouvet, G. ^1^, Rennuit, I. ^1^, Renner, J. ^1^, Hoffmann, C. ^1^, Schaal, J. ^1^, Donat, N. ^1,2^, Leclerc, T. ^1,2^

^1^ Burn Center Unit, Percy Teaching Military Hospital, Clamart, France, ^2^ French Military Health Service, French Military Health Service Academy, Paris, France.

Poster presentations 4, 5 September 2025, 10:00–10:30

Aim:

Our objective was to describe the hemodynamic kinetic changes immediately after severe burn injuries, which are known to be characterized by complex cardiovascular disorders.

Methods:

We retrospectively collected data from severely burned patients admitted within 24 h of burn with early initiation of PiCCO monitoring. Age, mortality, total burns surface area (TBSA), third degree burn area and smoke inhalation injury were assessed. Using transpulmonary thermodilution and arterial pulse wave contour analysis, hemodynamic features were recorded hourly during the first-96 h care: cardiac index (CI), systemic Vascular Resistance Index (SVRI), Heart Rate and arterial mean pressure.

Fluid resuscitation followed a standardized local protocol.

Hemodynamic parameter curves were plotted for each patient, then analyzed in population, each variable curve was smoothed by a generalized additive model. Hemodynamic patterns were identified through hierarchical clustering. Data analysis was performed with R.

Results:

102 patients were included between January 2017 and January 2025, median age was 52 years [26–78] and median TBSA was 40% [15–65]. In-hospital mortality rate was 21% (*n* = 21). Average CI significantly increased, while average RVSI significantly decreased over the first 96 h.

Three hemodynamic profiles were highlighted, regarding CI evolution: continuously increasing CI, stabilization or decline after initial rise. SVRI decreased in all clusters. Mortality was significantly different between clusters: it was lower in the first one (increasing CI), in this cluster patients were significantly younger.

Conclusions:

These results confirm a two-phases evolution after severe burn injury: first a hypokinetic—vasoconstriction state then a hyperdynamic circulation with vasoplegia.

P346

Fluid resuscitation in patients with major burn injuries using the Acumen advanced monitoring system.

Vasileiadou, G. ^1^, Giannaki, C. ^1^, Hadzigeorgiou, A. ^1^, Mouskeftara, I. ^1^, Tzimou, M. ^1^, Karachristos, C. ^1^, Papaioannou ^1^, Kaimakamis, E. ^1^, **Lavrentieva, A.** ^1^

^1^ Papanikolaou Hospital, Thessaloniki, Greece

Poster presentations 4, 5 September 2025, 10:00–10:30

Objective:

To evaluate fluid resuscitation in patients with major burn injuries and to determine whether the use of advanced hemodynamic monitoring is associated with resuscitation fluid volumes.

Methods:

Data from 12 patients admitted to a burn ICU were analyzed. Advanced hemodynamic monitoring was implemented in all patients using the Acumen system (Acumen IQ sensor and HemoSphere Advanced Monitor Platform [HS-IQ]). The volumes of resuscitation fluids administered were documented and analyzed in relation to the calculated fluid volumes during the initial 24 h post-burn.

Results:

A total of 12 burn patients who underwent advanced hemodynamic monitoring were included in the statistical analysis. The baseline characteristics of the study population were as follows: the mean age was 42.4 years (standard deviation [SD] 19), with a mean APACHE II score of 13.8 (SD 6) and a mean SOFA score of 5.1 (SD 1.8). Most patients (75%) were male. Inhalation injury was present in approximately two-thirds of the cohort (8 patients, 66%). Patients received significantly lower volumes of resuscitation fluids than those calculated using the Parkland formula (8513 ±5837 mL [2.9 mL/kg/TBSA] vs. 12,408 ± 8190 mL [4 mL/kg/TBSA]). The mean fluid volumes administered during the second and third post-burn days remained elevated (7945 ± 2567 mL and 5725 ± 1234 mL, respectively).

Conclusions:

The use of advanced hemodynamic monitoring resulted in lower fluid resuscitation volumes and may potentially enhance patient outcomes. Relatively high fluid volumes were also administered during the later phases of burn injury, warranting further investigation into optimal resuscitation strategies.

P347

The time—course of non-conventional prognostic markers after major burn injury

Körmendi, L. ^1^, **Loibl, C.** ^1^, Prof. Dr. Csontos, C. ^1^, Dr. Keresztes, D. ^1^, Dr. Kiss, T. ^1^, Dr. Rendeki, S. ^1^, Dr. Vértes, J. ^1^, Dr. Rozanovic, M. ^1^

^1^ University Of Pécs, Medical School—Department of Anaesthesiology and Intensive Therapy, Pécs, Hungary

Poster presentations 4, 5 September 2025, 10:00–10:30

Aim:

The goal of this study was to investigate the time course of NLR (neutrophile lymphocyte ratio), PLR (platelet lymphocyte ratio), and PAR (platelet antisedimentation rate) after major burn injury.

Methods:

This prospective, observational study comprised 23 patients with 20% or more TBSA after admission to intensive care unit. NLR, PLR and PAR were followed for a five days observational period. Blood samples for qualitative white blood cells and thrombocytes were collected daily, ratios like NLR and PLR were calculated consequently. PAR was measured by one-hour gravity sedimentation. It detects the percentage of total platelet number crossed the half line of blood sample column, therefore, they can be regarded as cells of decreased specific gravity.

Results:

In the observational period NLR and PLR levels showed a decreasing tendency, on the other hand PAR values showed an increasing tendency, respectively. Comparing the kinetics to the previous days, PAR values were statistically significant (*p* < 0.01) from day 3. Statistically difference were not found in NLR and PLR levels between the uncomplicated and the complicated groups. Comparing to the members of the complicated group, PAR values were significantly higher on day 2 (*p* < 0.05) and from day 3 (*p* < 0.001) in the uncomplicated group.

Conclusions:

These non-conventional markers may have a role in the earlier detection of unfavorable outcomes (e.g., onset of sepsis) after major burn injury. Higher sample size are necessary to declare more reliable correlations and to clarify its application in the everyday practice.

P349

Surgical management of tracheostomy in patients with severe burns and cervical involvement

Thielmann, J. ^1^, Heitzmann, W. ^1^, Ried, M. ^2^, von Kohout, M. ^1^, Fuchs, P. ^1^, Schiefer, J. ^1^, **Markowiak, T.** ^1,3^

^1^ Clinic of Plastic, Reconstructive, Hand and Burn Surgery, Hospital Cologne Merheim, Cologne, Germany, ^2^ Department of Thoracic Surgery, University Medical Center Regensburg, Regensburg, Germany, ^3^ Correspondence: MarkowiakT@kliniken-koeln.de

Poster presentations 4, 5 September 2025, 10:00–10:30

Aim:

This study investigates how the timing, surgical burn treatment and the method of tracheostoma closure influence the course of treatment in patients with severe burns and involvement of the neck.

Methods:

In a retrospective analysis, 48 patients with severe burn trauma and neck involvement between 2015 and 2024 were analyzed. The correlation between tracheotomy and burn defect coverage over time, a comparison of different coverage and tracheostoma closure methods in relation to wound healing disorders and the number of plastic coverings were examined.

Results:

The mean burned body surface area was 28% (±16.9) and the mean age was 48.1 (±17.7) years. The median ABSI score was 8 (IQR 5). Tracheostoma closure was performed surgically in 37.8% of patients and conservatively in 62.2%. The rate of wound healing disorders was 35.1% (*n* = 13). There were no significant differences with regard to the timing (tracheotomy after split-thickness skin grafting (*n* = 16) versus split-thickness skin grafting before or at the same time as tracheotomy (*n* = 8); *p* = 0.56), the method of split-thickness skin grafting (Meek (*n* = 8) versus MESH (*n* = 16); *p* = 0.77) and the type of tracheostoma closure (surgical (*n* = 14) versus conservative (*n* = 23); *p* = 0.63) in relation to the occurrence of postoperative wound healing disorders.

Conclusions:

The rate of wound healing disorders in these patients is high. There is no clear trend regarding the optimal timing and surgical procedure to minimize the occurrence of this complication, so the surgical procedure should be determined individually based on clinical circumstances.

P350

Robot Portrait of tunisian burn patient affected by chronic critical illness

Fredj, H. ^1^, Cheikhrouhou, M. ^1^, Aloui, A. ^1^, Alouini, A. ^1^, Jemi, I. ^1^, Gasri, B. ^1^, Messadi, A. ^1^, **Mokline, A.** ^1^

^1^ Trauma and Burn Center, Tunis, Tunisia

Poster presentations 4, 5 September 2025, 10:00–10:30

Aim of our study was to determine the profile of a Tunisian burn patient affected by Chronic Critical Illness (CCI). 

 Methods:

Retrospective study was conducted over 1 year (2021) in the Burn Care Unit in Tunisia. Adult patients with a hospital stay ≥ 8 days were included. CCI was defined according to criteria established by the Research Triangle Institute: ICU stay ≥ 8 days and at least one of the following 5 conditions:mechanical ventilation (MV) ≥ 96 h, use of tracheotomy, occurrence of infection, stroke or head trauma, and presence of severe wounds.

Results:

During the study period, 463 patients were admitted, among them 85 were included (CCI incidence of 31%). Most patients had at least 2 eligible CCI criteria (*n* = 59; 69.4%). Sepsis was the most frequent criterion (*n* = 36; 42.4%), followed by MV (*n* = 33; 38.8%). Age of patients was 41.5 ± 17 years, with a sex ratio of 1.65. TBSA was 29 ± 16%. Thirty-eight patients (44.7%) were ventilated upon admission, and 17 patients (20%) were in shock. At least one septic episode occurred in 97% of cases patients. Twenty-five patients (29.4%) developed ARDS. Biological complications were: anemia (*n* = 81), thrombocytopenia (*n* = 57), acute renal failure (*n* = 56), and hypophosphatemia (*n* = 63). Length of MV was 8 days and length of ICU stay was 13 days. Mortality was 55.3%.

Conclusions:

The Tunisian burned patient affected by CCI is typically a 41 year-old male, without significant medical history, suffering from extensive thermal burns, had sepsis in all cases and ICU stay of 13 days.

P351

An unusual case of life-threatening idiosyncratic linezolid-induced type B lactic acidosis & serotonin syndrome occurring simultaneously in a major burn patient.

Myers, S. ^1^, **Safa, R.** ^1^

^1^ Sheikh Shakhbout Medical City, Abu Dhabl, United Arab Emirates

Poster presentations 4, 5 September 2025, 10:00–10:30

Aim:

To present two rare but life-threatening reactions to Linezolid, occurring in the same burn patient survivor.

Methods:

Review of the patient records and existing literature.

Results:

We present a case of marked hyperthermia and lactic acidosis in a patient occurring 15 days following a massive burn injury. The hyperthermia occurred acutely and progressed rapidly, necessitating aggressive cooling measures. Based on the acute onset and rapid progression of hyperthermia and acidosis, a drug reaction was suspected. Linezolid is known to be associated with both lactic acidosis and serotonin syndrome, particularly when given in combination with other medications. Linezolid, a member of the oxazolidinone class, is a synthetic antibiotic used not infrequently in burn practice, that inhibits protein synthesis by binding to bacterial ribosomal RNA (rRNA), and also exerts a weak inhibitory effect on monoamine oxidase. The former effect is thought to mediate Linezolid induced lactic acidosis via cross-inhibition of eukaryotic mitochondrial rRNA. The latter effect mediates Linezolid-induced serotonin syndrome. 

Conclusions:

The clinical features and diagnosis of both reactions are considered in relation to the case. Although both diagnoses are by exclusion, the case appeared to exhibit key features of both. The management and outcome are discussed.

P352

Hyperbaric Oxygen Therapy in Carbon Monoxide Poisoning: A Case Series

**Nai, K.** ^1^, Li, Z. ^1^, Tay. R. ^1^, Chua, W. ^2^, Kim, S. ^3^, Tan, M. ^3^, Foo, C. ^1^, Tan, K. ^1^, Tan, B. ^1^, Chong, S. ^1^

^1^ Singapore General Hospital, Department of Plastic, Reconstructive & Aesthetic Surgery, Singapore, Singapore, ^2^ Yong Loo Lin School of Medicine, National University of Singapore, Singapore, Singapore, ^3^ Singapore General Hospital, Hyperbaric and Diving Medicine Centre, Singapore, Singapore.

Poster presentations 4, 5 September 2025, 10:00–10:30

Aim:

Acute carbon monoxide (CO) poisoning is commonly associated with burn incidents. Hyperbaric oxygen therapy (HBOT) has been found to be an effective treatment for preventing the undesired neurological sequelae of CO poisoning. The aim of this study was to review patients who underwent HBOT for acute CO poisoning at our institution, a regional burns centre in Southeast Asia.

Methods:

This case series is a retrospective review of four cases from 2022–2024 that were treated at the Singapore General Hospital with HBOT for acute CO poisoning.

Results:

Three out of the four cases discussed recovered rapidly following HBOT, regaining full consciousness with normalization of their carboxyhaemoglobin (COHb) levels.

Conclusions:

Whilst more research needs to be carried out to better standardize HBOT patient selection, timing of initiation, and cycles of treatment for patients with CO poisoning, this case series demonstrates that HBOT has been able to deliver good neurological results in some patients. It also demonstrates our institution’s experience and capability to safely administer multiple cycles of HBOT to CO-poisoned patients in a timely manner in keeping with global standards.

P353

A Meta-Analytic Perspective on Long-Term Cardiac Dysfunction After Burns, Sepsis, and Trauma

Niederegger, T. ^1^, **Schaschinger, T.** ^1^, Knoedler, L. ^3^, Brandt, J. ^1^, Kauf, C. ^1^, Maheta, B. ^4^, Ramsamooj, A. ^4^, Most, P. ^1^, Ritterhoff, J. ^1^, Panayi, A. ^2^, Hundeshagen, G. ^2^

^1^ University of Heidelberg, Medical Faculty, Heidelberg, Germany, ^2^ Department of Hand, Plastic and Reconstructive Surgery, Burn Center, BG Trauma Center Ludwigshafen, University of Heidelberg, Ludwigshafen, Germany, ^3^ Charité—Universitätsmedizin Berlin, corporate member of Freie Universität Berlin, Humboldt-Universität zu Berlin, and Berlin Institute of Health, Department of Oral and Maxillofacial Surgery, Berlin, Germany, ^4^ California Northstate University College of Medicine, Elk Grove, USA.

Poster presentations 4, 5 September 2025, 10:00–10:30

Aim:

This meta-analysis aims to synthesize four decades of data to assess long-term cardiac dysfunction after burns, sepsis, and trauma, conditions whose lasting myocardial effects remain underexplored despite emerging evidence beyond the well-documented acute changes.

Methods:

PubMed, EMBASE, Cochrane, and Web of Science were searched through 1 March 2025, for studies reporting long-term echocardiographic outcomes. Study quality was evaluated, and statistical analysis was conducted on key echocardiographic parameters, stratified by time post-injury.

Results:

Overall, 46 studies were included, encompassing *n* = 3109 patients with burns (*n* = 1876), sepsis (*n* = 901), and trauma (*n* = 332). Burn studies had longer follow-up (mean ± SD 2.2 ± 3.3 years) than sepsis (mean ± SD 0.8 ± 2.3 years) and trauma (mean ± SD 0.8 ± 2.8 years). Diastolic dysfunction was most evident in sepsis: E/A ratios (normal >0.8) averaged 0.74 and declined to 0.57 beyond 2 weeks. E/e ratios (normal ≤ 8) were elevated in sepsis (16.2) vs. burns (7.6). Heart rates remained tachycardic (>100 BPM) in burns (125.3) and trauma (136.0) beyond one year. Burn patients showed reduced systolic function, with ejection fraction (normal ≥ 60%) averaging 39.8% for up to one year.

Conclusions:

This meta-analysis suggests potential long-term cardiac impairment following burns, sepsis, and trauma, the most pronounced in burn survivors with possible diastolic decline in sepsis patients. While the data were limited and heterogeneous, emerging trends indicate that myocardial changes may persist beyond clinical recovery. These findings underscore the need for further high-quality, longitudinal research and support consideration of extended cardiac monitoring in critical illness survivors.

P354

Application of Cytosorb therapy in patients with severe electrical injuries

**Nietzschmann, I.** ^1^

^1^ BG-Klinik, Halle, Germany

Poster presentations 4, 5 September 2025, 10:00–10:30

Aim:

The Cytosorb adsorber adsorbs hydrophobic low- and medium-molecular substances, including myoglobin, in a concentration-dependent manner. At high concentrations, large quantities can be removed very quickly. As the concentration decreases, the removal rate decreases.

Methods:

We would like to present the case of a severely current-injured patient who was treated with Cytosorb therapy.

Results:

We took the patient with a high current injury directly from the accident site and treated him with a Cytosorb filter within the first 24 h. As a result, acute renal failure (crus kidney) was prevented.

Conclusions:

Patients appear to benefit from the use of a Cytosorb filter in the acute phase.

P356

Long-term effects of severe burns on cardiac function in adult burn survivors: an observational study.

**Palackic, A.** ^1^, Brandt, J. ^1^, Stolle, A. ^1^, Schaschinger, T. ^1^, Niederegger, T. ^1^, Kühn, S. ^1^, Pennekamp, A. ^1^, Kneser, U. ^1^, Hundeshagen, G. ^1^

^1^ Department of Hand, Plastic and Reconstructive Surgery, Burn Center, BG Trauma Center Ludwigshafen, University of Heidelberg, Ludwigshafen, Germany.

Poster presentations 4, 5 September 2025, 10:00–10:30

Aim:

To determine cardiac function and myocardial morphology qualitatively and quantitatively using echocardiography. Inflammatory mediators are correlated with cardiac function and morphology.

Methods:

As part of this prospective, observational clinical study, severely burned patients undergo echocardiography in the acute phase of their injury (up to 3 months) and in the long-term course of outpatient follow-up care of their injury (up to 10 years). Blood samples are taken at any timepoint for molecular biological analysis and inflammatory mediators are determined. A group of healthy, unburned subjects represents the control group. The clinical data on the severity of the initial injury are collected and analyzed for their predictive value with regard to the development of long-term burn-associated cardiac dysfunction.

Results:

Intra-individual controls (the same patient over several time points) and inter-individual comparisons (study participants with different clinical and/or demographic characteristics at the same time points) will characterize and quantify the post-traumatic course. Data obtained on the systemic factors of the post-traumatic long-term reaction will be correlated with clinical data on cardiac function in order to identify pathophysiological correlations, risk factors for cardiovascular dysfunction and starting points for possible interventions. Echocardiography will be used to obtain a functional representation of the decisive parameters in order to visualize burn-associated cardiac dysfunction.

Conclusions:

Severe burns have not yet been identified as a special risk group for early heart failure and may need to be given separate and specific follow-up care and therapy in order to avoid long-term survival and morbidity disadvantages compared to healthy individuals.

P357

Case report: a rare case of extensive burn by phenol acid in a young man at burn centre of Turin

**Pensa, A.** ^1^, Sciarrillo, A. ^1^, Ciocan, C. ^2^, Di Giovanni, F. ^3^, Risso, D. ^1^, Navissano, M. ^1^

^1^ University Of Turin, Torino, Italy, ^2^ Department of Occupational Medicine, Azienda Ospedaliera Città della Salute e della Scienza, Turin, Italy, ^3^ Department of Pathology, Azienda Ospedaliera Città della Salute e della Scienza, Turin, Italy.

Poster presentations 4, 5 September 2025, 10:00–10:30

Aim:

Describe the complications and management of a rare case of burn injury by phenol acid.

Methods:

A 45 year old truck man had an accidental injury by phenol acid occuring a 35% of burn extension. After first assistance and decontamination by polyethylene glycol solution he arrived ventilated and with amine support but he had no other traumatic injury. The local assessment was: TBSA 35% of partial and full thickness burn on perineal region, legs, right arm and a small abdominal region, Revised Baux score 97. During the recovery before he had hepatic insufficiency than a renal failure which was treated by depurative dialysis. After a few days a total scan tomography revealed multiple ischemic cerebral areas. During the recovery a skin biopsy and toxicology analysis about the level of phenol were performed.

Results:

After 12 days from admission and episodes of bronchial bleeding and the patient died due a multiorgan failure.

Conclusions:

This case report reveal the chemical burn of phenol was fatal and the importance of a multidisciplinary management to treat the multi organ complications.

P359

Expanding the Role of Regional Anesthesia in Burn Surgery: A Review of Three Cases

**Skunca, A.** ^1^, Lokosek, V. ^1^, Dobric, M. ^1^, Zagorac, D. ^1^, Pedic, L. ^2^

^1^ Sestre Milosrdnice University Hospital Center, Zagreb, Croatia, ^2^ Zadar General Hospital, Zadar, Croatia

Poster presentations 4, 5 September 2025, 10:00–10:30

Aim:

Pain is the most common complaint among burn-injury patients, ranging from mild to severe. While opioids remain a mainstay of burn pain management, regional anesthesia is increasingly recognized as a safe and effective addition. In addition to reducing opioid consumption, regional anesthesia techniques have been shown to decrease hyperalgesia, vasospasm, and local thrombosis during skin grafting—factors that can compromise graft function. Here we present three cases demonstrating the diverse applications of regional anesthesia in burn care.

Method:

The first case involves a 29-year-old pregnant female (first trimester of pregnancy) that required skin grafting of the forearm and hand, using the axillary nerve block. The second case features a 76-year-old male and application of a popliteal and saphenous nerve block for the grafting of skin on the foot. The third case describes a 38-year-old female scheduled for skin grafting of the right neck and chest region, performed under paravertebral, superficial cervical, and spinal block, with additional propofol sedation.

Results:

All three patients received a combination of regional anesthesia blocks to cover both the recipient and donor sites, experienced no pain for 8–12 h postoperatively and remained comfortable overnight with opioid-free analgesia.

Conclusions:

These cases highlight the adaptability of regional anesthesia in burn patients, especially in vulnerable populations such as pregnant or elderly patients with comorbidities. Beyond pain control, regional anesthesia techniques enhance perioperative safety, facilitate early mobilization, and may improve graft viability. We advocate for its broader adoption in burn patient care—are we ready to embrace its full potential?

P360

The Meek Technique vs partial-thickness skin graft in Major Burns patients: Experience in the National Center of Research and Care of Burns patients in Mexico City

**Lopez, A.** ^1^, Mendieta, A. ^1^, Velez, M. ^1^, Estrada, J. ^1^

^1^ Instituto Nacional de Rehabilitación, Ciudad de Mexico, México

Poster presentations 4, 5 September 2025, 10:00–10:30

Aim:

To understand the behavior of patients treated with the Meek technique compared to split-thickness grafting.

Methods:

A clinical, observational, cross-sectional study conducted from March 2022 to February 2024, comparing hospital length of stay (LOS), intensive care unit LOS, and outcomes in patients with major burn injuries. We compared these data between the Meek technique (*n* = 11) and split-thickness skin grafting (STSG) (*n* = 27).

Results:

Among the patients, in the Meek group (*n* = 11), 6 were male, while in the STSG group (*n* = 27), 18 were male. The mean total body surface area (TBSA) for the Meek group was 63.9 ± 17.9%, and for the STSG group, it was 63.6 ± 19% (*p* = 0.97). The mean LOS at the hospital for the Meek group was 41.1 ± 18 days, while for the STSG group, it was 27.2 ± 22 days (*p* = 0.10). The LOS in the intensive care unit was 32.1 ± 13.1 days for the Meek group and 19.4 ± 11.2 days for the STSG group (*p* = 0.04). A higher frequency of deaths was observed with the STSG technique compared to the Meek technique (59.3% vs 45.5%; *p* = 0.03).

Conclusions:

Preliminary results with the Meek technique are encouraging, showing a lower death rate in patients with severe burns. However, the prolonged LOS is a risk factor to consider in this group of patients.

P361

Monitoring protocol with Masimo technology for optimized care of critical burn patients in deep sedation for enzymatic desbridament (Nexobrid).

Vidal, A. ^1^, **Bayo, M.** ^2^, Roig, G. ^3^, Garcia, A. ^3^, Garcia, M. ^2^, Ferre, M. ^1^, Pérez del Caz, M. ^2^

^1^ Anaesthesia and Critical Care Departament, Hospital Universitari I Politècnic La Fe, Valencia, Spain, ^2^ Plastic and Reconstructive Surgery Departament, Hospital Universitari i Politècnic La Fe, Valencia, Spain, ^3^ Critical Burn Nursing, Hospital Universitari i Politècnic La Fe, Valencia, Spain.

Poster presentations 4, 5 September 2025, 10:00–10:30

Aim:

This study aims to evaluate the benefits of monitoring the Oxygen Reserve Index (ORI) during deep sedation in burn patients undergoing enzymatic debridement with the use of a high-flow nasal cannula (HFNC). ORI provides a non-invasive, continuous assessment of oxygenation status, offering an early warning of desaturation before changes in SpO_2_ occur. The combination of ORI and HFNC may enhance patient safety by maintaining adequate oxygenation throughout the procedure.

Methods:

A review of existing literature on ORI and HFNC was conducted to assess their clinical benefits in high-risk patients. ORI’s ability to provide early warnings of desaturation was analyzed, including its median added warning time of 48.4 s before SpO_2_ dropped to 94% in high-risk surgical patients. The physiological benefits of HFNC, such as improved oxygen delivery and positive airway pressure, were also examined.

Results:

Findings suggest that ORI enables early detection of oxygenation issues, allowing clinicians to intervene before critical desaturation occurs. HFNC further improves oxygenation in burn patients by ensuring high oxygen delivery and respiratory support. The combined use of ORI and HFNC enhances patient safety during enzymatic debridement under deep sedation.

Conclusions:

Implementing ORI monitoring alongside HFNC in burn patients undergoing enzymatic debridement offers significant clinical advantages. This approach provides an additional safety margin, reducing the risk of hypoxia and improving patient outcomes in all clinical settings. Future studies should further explore its impact on morbidity and mortality in this population.

P362

Masimo oximetry monitoring: protocol update in our critical brun unit for improve patient care

Vidal, A. ^1^, Ivañez, B. ^3^, **Bayo, M.** ^2^, Roig, G. ^3^, Garcia, A. ^3^, Ferre, M. ^1^, Garcia, M. ^2^, Pérez del Caz, M. ^2^

^1^ Anaesthesia and Critical Care Departament, Hospital Universitari I Politècnic La Fe, Valencia, Spain, ^2^ Plastic and Reconstructive Surgery Departament, Hospital Universitari i Politècnic La Fe, Valencia, Spain, ^3^ Critical Burn Nursing, Hospital Universitari i Politècnic La Fe, Valencia, Spain.

Poster presentations 4, 5 September 2025, 10:00–10:30

Aim:

To establish a standardized monitoring protocol using Masimo RD Rainbow sensors (Irvine, CA, USA) in patients admitted to the Critical Burn Unit, those undergoing surgery, and those requiring continuous monitoring during scheduled treatments.

Methods:

Patients meeting admission criteria for the Critical Burn Unit are monitored using the RD Rainbow Set-2 sensor (Oxigen Reserve Index—ORi-, Hemoglobin -Hb-, oxygen saturation -SpO2-, Pulse Variability Index -PVi-). If inhalation syndrome is suspected, carboxyhemoglobin (COHb) levels are monitored with the RD Rainbow Neo 8λ SpCO sensor. Monitoring is continued in critical care. In scheduled surgeries, the RD Rainbow Lite Set-2 sensor is used for patients undergoing debridement and grafting procedures with high bleeding risk, as well as for microvascularized flap techniques. Patients over 70 years with comorbidities or respiratory diseases are monitored with the RD Rainbow Lite Set-1 sensor for ORi and SpO2. In the scheduled cure room, patients already monitored in the operating room or critical unit continue using the same sensor, while others receive the RD Rainbow Lite Set-1 sensor for ORi and SpO2 monitoring during sedation.

Results:

The implementation of this protocol ensures continuous, tailored monitoring of burn patients at different stages of care. It optimizes patient safety by enabling early detection of hypoxemia, bleeding, and respiratory complications, particularly in high-risk patients.

Conclusions:

A structured approach to sensor-based monitoring in burn patients enhances clinical decision-making and improves patient outcomes. Standardizing sensor use across critical, surgical, and recovery settings ensures continuous and appropriate physiological assessment.

P364

VR projects at the Prague Burn Centre 2025

**Zajicek, R.** ^1,2^, Bakalar, B. ^2,3^, Simicekova, A. ^1^, Diakonu, A. ^2^, Stonova, C. ^1,2^, Hocko Fajnerová, I. ^2^, Zielina, M. ^4^

^1^ Prague Burn Centre, Faculty Hospital Kralovske Vinohrady, Prague, Czech Republic, ^2^ Charles University, Third Medical Faculty, Prague, Czech Republic, ^3^ Department of Anaesthesia and Intensive Care Medicine, Faculty Hospital Kralovske Vinohrady, Prague, Czech Republic, ^4^ Charles University, Second Medical Faculty, Prague, Czech Republic.

Poster presentations 4, 5 September 2025, 10:00–10:30

Objective:

Virtual reality (VR) is rapidly becoming an accessible and transformative technology in a wide range of medical applications. In burn medicine, the impact of VR is becoming evident in a number of applications.

Methods and results:

At the Prague Burn Centre (PBC), the use of VR is the subject of intensive research. It has also become an integral component of the treatment of burn patients. PBC is currently engaged in five modules of VR applications and research.

1. The AVATAR project is based on a unique combination of VR and illusory movements for the prevention and treatment of polyneuromyopathy.
2. The BURN-VRelax project employs VR relaxation techniques to mitigate anxiety and stress in healthcare professionals.
3. The Cold River project is focused on the utilisation of immersive VR to mitigate procedural pain in burn patients.
4. At PBC, the utilisation of VR in clinical practice for post-burn scar rehabilitation is a customary practice.
5. VR-BURNESTIMATION project serves for the education in the assessment of burn trauma severity factors.

Conclusions:

VR is becoming an integral part of burn management and education at PBC.

P400

Intact fish skin graft and traumatic wound reconstruction: a case series

**Lopez-Quinones, H.** ^1^

^1^ Jacobi Medical Center, Bronx, New York, United States

Poster presentations 5, 5 September 2025, 16:15–16:45

Aim:

To share our experience with application of intact fish skin graft (IFSG) for the reconstruction of complex soft tissue injuries.

Methods:

Burn surgeons frequently encounter patients with non-thermal wounds and can offer a distinct perspective by employing reconstructive strategies proven successful in the field of burn. In this work we describe the use of IFSG as part of the reconstructive ladder for complex traumatic injuries. A retrospective case series was conducted examining patients with non-thermal injuries referred to our Burn Center for wound closure from January to June 2023. Electronic medical records were reviewed to obtain clinical photographs, demographics and surgical outcomes. Patients had planned follow-up of 6-months minimum. Descriptive statistics were used to summarize findings.

Results:

Four patients with traumatic wounds of varying etiology had reconstruction with IFSG. They underwent a series of debridements using a combination of surgical techniques. IFSG was applied to all wounds to rebuild dermal bulk and improve contour. Minimum IFSG applications was two. Once healthy granulation was present wounds underwent definitive closure with meshed skin grafts and autologous skin cell suspension. Time to closure varied from 4–8 weeks. Typically, ≥85% re-epithelialization was present at 1-week and ≥99% by month 2. All wounds showed good evidence of repigmentation.

Conclusions:

Intact fish skin graft is a valuable tool for reconstruction of complex traumatic injuries allowing for adequate wound bed preparation prior to definitive closure and potentially decreasing the need for more complex surgeries.

P401

Do silver dressing promote healing of skin lesions in Lyell’s syndrome ?

Fredj, H. ^1^, Aloui, A. ^1^, Jami, I. ^1^, Gasri, B. ^1^, Messadi, A. ^1^, **Mokline, A.** ^1^

^1^ Burn Intensive Care Unit, Traumatology and Burn Center, Ben Arous, Tunisia

Poster presentations 5, 5 September 2025, 16:15–16:45

Introduction: Silver-based dressings possess antimicrobial and anti-inflammatory properties.

The aim of this study was to evaluate the benefits of using silver dressings in healing skin lesions in patients with Lyell’s syndrome.

Methods:

A retrospective and descriptive study was conducted in the burns intensive care unit of Tunis over a 15-year period (January 2009–December 2024), including patients admitted for the management of Lyell’s syndrome. To assess the impact of silver dressings on wound healing, we compared patients who received silver dressings (G1: Silver+) with those who did not (G2: Silver−).

Results:

During the study period, 84 patients were admitted for Lyell’s syndrome. Among them, 81 were included. The average age was 41 ± 15 years. The sexe-ratio was 0.68. The most frequently implicated drugs were anticonvulsants (37%), antibiotics (21%), and hypouricemic agents (18.5%). The median detached skin surface (DSS) was 40% [30–50%]. Sixty-three patients (78%) received silver dressings (G1: Silver+). These patients were compared to those who did not receive silver dressings (G2: Silver−). The two groups were comparable in terms of age (G1: 32 ± 15.6 years vs. G2: 30.5 ± 7.8 years; *p* = 0.52) and detached skin surface (G1: 35 ± 8% vs. G2: 37 ± 9%; *p* = 0.13). Silver dressings significantly reduced the infection rate (G1: 36% vs. G2: 67%; *p* = 0.03) and shortened the healing time (G1: 15.2 days vs. G2: 21.5 days; *p* = 0.01).

Conclusions:

In patients with Lyell’s syndrome, silver dressings reduced the infection rate of lesions and significantly shortened the healing time.

P403

An expanded access program with NexoBrid for treatment of deep partial and full thickness burn injuries in adult and pediatric patients.

**Martínez Méndez, J.** ^1^

^1^ La Paz University Hospital, Madrid, Spain

Poster presentations 5, 5 September 2025, 16:15–16:45

Aim:

An Expanded Access Program (EAP) in the U.S. allowed centers to gain more experience treating adult and pediatric burn patients with NexoBrid after completing enrollment in global Phase III studies and to maintain burn care preparedness for mass casualty incidents.

Methods:

A single-arm EAP was conducted at 23 U.S. burn centers. Eligible patients included children (<18) and adults (≥18) with deep thermal burns covering up to 30% TBSA. NexoBrid was applied, and treatment followed standard care. Patients were monitored weekly until wound closure and at 3 and 12 months. Outcomes included incidence and time to eschar removal, need for surgical excision or escharotomy, length of hospital stay (LOS), wound closure, and Modified Vancouver Scar Scale (MVSS).

Results:

239 patients (215 adults, 24 pediatric) received NexoBrid (2019–2024), with 142 (131 adults, 11 pediatric) completing the 12-month follow-up. Mean age was 41 and 11 years. The target wound area was around 6% TBSA, with 38 circumferential burns. Eschar removal was achieved in 95% of adults and 100% of pediatric patients in 4 h. Surgical excision was required in 4% of adults, none in pediatric cases. No escharotomies were needed. LOS was 10 days. Wound closure occurred at 22 days (adults) and 28 days (pediatric). High autograft use in deep partial-thickness wounds (49%) resulted in higher MVSS than previous studies. Safety was consistent.

Conclusions:

NexoBrid demonstrated comparable or improved outcomes versus previous Phase III studies and potential efficacy in preventing burn-induced compartment syndrome.

P404

The use of polylactide-based matrix in refractory chronic wounds

**Hecker, A.** ^1,2,3^, Moshammer, M. ^1,2^, Watzinger, N. ^1,2^, Pignet, A. ^1,2^, Kotzbeck, P. ^1,2^, Kamolz, L. ^1,2^

^1^ Medical University of Graz, Deparment of Surgery, Division of Plastic, Aesthetic and Reconstructive Surgery, Graz, Austria, ^2^ Joanneum Research Forschungsgesellschaft mbH, COREMED—Centre for Regenerative Medicine and Precision Medicine, Graz, Austria, ^3^ Hospital Ernst von Bergmann, Department of Plastic, Aesthetic and Reconstructive Microsurgery/Hand Surgery, Potsdam, Germany.

Poster presentations 5, 5 September 2025, 16:15–16:45

Aim:

SupraSDRM^®^ (PMI, Kirchheim unter Teck, Germany) is a novel alloplastic dermal substitute composed of a fully synthetic, polylactide-based, hydrolytically resorbable matrix. This prospective clinical study aims to evaluate the use of SupraSDRM^®^ in patients with therapy-refractory chronic wounds, assessing wound healing and selected wound-related parameters.

Methods:

This monocentric, prospective study included patients with therapy-refractory chronic wounds. Wounds were classified as therapy-refractory if they showed less than 15% reduction in size after two weeks of standard-of-care treatment. Following appropriate debridement, SupraSDRM^®^ was applied weekly for three months. Wound size and volume (3D camera), wound pH (pH meter), and wound quality (Hollander Wound Evaluation Scale) were recorded.

Results:

A total of nine patients (eight men, one woman) with a mean age of 66 years (±7.7) were included. Eight cases of leg ulcers and one case of a diabetic foot ulcer, with an average wound area of 15.7 cm^2^ (±5.8) and an average wound volume of 4.0 cm^3^ (±3.1), were treated with SupraSDRM^®^. After three months, the average wound area had decreased to 12.6 cm^2^ (±11.9), and wound volume was reduced to 2.4 cm^3^ (±2.8). Wound quality improved from 5.9 (±1.3) at baseline to 3.6 (±2.6) after three months. The pH value within the chronic wound decreased from 7.6 (±0.6) at baseline to 7.1 (±0.4) after three months.

Conclusions:

The use of the novel alloplastic dermal substitute SupraSDRM^®^ in therapy-refractory chronic wounds has shown promising results across various wound healing-related parameters, potentially leading to improved healing outcomes in these patients.

P405

PRF-enriched nanofat membrane for the treatment of facial burns after enzimatic debridement

**Iglesias Álvarez, A.**, Serracanta i Domenech, J., Rivas Nicolls, D., Bulla, A., López Martínez, J., Orois Gómez, S., González Peña, F., Cuadros Ramírez, M., Barret Nerín, J.

^1^ Hospital Universitari Vall D’hebron, Barcelona, Spain.

Poster presentations 5, 5 September 2025, 16:15–16:45

Aim:

This report presents our experience with one clinical case in which autologous nanofat membrane was used for the treatment of severe facial burns.

Methods:

We collected one patient with severe facial burns. Initially, enzymatic debridement (ED) with Nexobrid^®^ was applied. Post-ED evaluation showed deep dermal burns and smalls areas of third degree burn. A fatty membrane was manufactured as described by Fakih–Manay (1). Fat was harvested from abdominal area and the inner thighs by syringe-assisted liposuction. The collected fat was then washed out with Puregraft^®^, emulsified to produce nanofat, and then combined with Platelet-Rich Fibrin (PRF) on a non-adherent gauze to create a Nanofat-PRF membrane. The membrane was placed on the affected facial areas and covered with Acticoat^®^. Seven days later it was removed and conventional burn care was provided. Later, a skin graft was performed on a residual area of full-thickness burn on the forehead.

Results:

The patient showed a faster healing time than standard, with reduced scarring and good skin texture. No major complications occured.

Conclusions:

Facial burns present significant challenges due to the need to restore both function and aesthetics. The use of autologous fat membranes could enhance healing due to its theoretical potential to preserve and boost cells with epidermal restorative capabilities. The result achieved in this case suggest that fat membrane may be a viable and a helpful option for burn care in demanding areas of the body, although further studies are needed to validate these findings and establish standardized applications protocols.

P406

Fish Skin as a Novel Biological Dressing in Burn Treatment: A Case Series of First Patients Treated

**Imsirovic, A.** ^1^, Casari, M. ^1^, Tasch, C. ^1^

^1^ Medical University of Innsbruck, Innsbruck, Austria

Poster presentations 5, 5 September 2025, 16:15–16:45

Aim:

The use of fish skin as a biologic wound dressing shows promising results in the treatment of burns and chronic, contaminated wounds.

Methods:

Fish skin was used as a bioactive dressing in five patients. On average, burns were between 3–8% of the total body surface area, and the average healing time was reduced to an average of two weeks. Patients were discharged from hospital after an average of 1–2 weeks.

Results:

Our first patient suffered a third-degree frying oil burn on the forearm. After a two-day delay, debridement and fish skin application conditioned the wound, allowing for a split-thickness skin graft a week later. It healed completely within two weeks.

The second patient had a week-old third degree burn on her abdomen. Complete healing was achieved by fish skin application alone.

The third patient, with alcohol syndrome, sustained third-degree burns on the buttocks and perineum. After a seven-day delay, the wound was cleaned and treated with fish skin. He was discharged with a healed wound after two weeks.

The fourth patient, who had diabetic neuropathy, sustained a forefoot burn that had been treated conservatively for a month. After debridement and fish skin application, the wound healed in 21 days.

The final patient presented with a chronic ulcer and burn-related trauma. Following debridement and fish skin pre-conditioning, a skin graft was applied, leading to full recovery.

Conclusions:

Our findings demonstrate the potential of fish skin as an effective treatment for severe burns, potentially reducing the need for skin grafting.

P407

The Influence of Atopy on Skin Graft Outcomes in Burn Patients: A Case Report

Iturralde, A. ^1,2^, Larre Borges, A. ^1^, **Amarelle, L.** ^1,2^, Silva, J. ^1,3^, Aramendi, I. ^1,2^

^1^ Centro Nacional De Quemados, Montevideo, Uruguay, ^2^ Unidad Académica Medicina Intensiva, Facultad de Medicina, UdelaR, Montevideo, Uruguay, ^3^ Unidad Académica de Cirugía Plástica, Facultad de Medicina, UdelaR, Montevideo, Uruguay

Poster presentations 5, 5 September 2025, 16:15–16:45

Aim:

Atopy is a genetic predisposition to exaggerated immune reactions. While its impact on graft outcomes in burn patients is unclear, it may influence immune responses and increase rejection risk.

Methods:

A 17-year-old girl with a medical history of atopy was admitted to our burn center with burns over 57% of her total body surface area. During the first two months of her stay, she underwent escharectomy and staged autografts, which showed partial adherence, with delayed epithelialization of the autograft donor areas. Over the following 9 months, repeated Meek micrografts had similar outcomes. Multiple types of dressings were tested to improve the wound bed, which remained bloody and caused intense pain.

Despite optimized nutrition, anabolic steroids, and complete treatment of bacterial and fungal infections, epidermal loss persisted. Skin biopsies showed epidermal necrosis with lymphoplasmacytic and eosinophilic infiltrates and dermal fibrosis. Immunoglobulin E levels were elevated. Suspecting an immune-mediated reaction and pathergy linked to dysbiosis, balneotherapies were spaced, topical corticosteroids were initiated, and the monoclonal antibody dupilumab was administered.

Results:

Following this intervention, healing improved, and pain resolved. No further grafts were needed.

Conclusions:

Atopy-related immune alterations may affect skin graft outcomes. Topical therapies, interventions to improve the skin microbiome, and systemic immunosuppressants could enhance graft success in atopic patients.

P408

The utility of Fish skin in treating war wounds.

**Jeffery, S.** ^1^

^1^ Pioneer Wound Clinics, Bentley Pauncefoot, Nr Bromsgrove, United Kingdom, ^2^ British Red Cross, Kyiv, Ukraine

Poster presentations 5, 5 September 2025, 16:15–16:45

Aim:

To describe the utility of using fish skin in treating war injuries

Methods:

The author has extensive experience of treating combat injuries. He has used fish skin during the Armenia-Azerbaijan war, and in 2024 spent 6 weeks with the British Red Cross working in the National Burn Centre in Kyiv. There he introduced the local surgeons to using fish skin.

Results:

Kerecis Omega3 fish skin graft has shown great portability, with easy transfer of knowledge. Kerecis Omega3 fish skin graft has shown faster granulation rates in burn and blast wounds for skin grafting, resulting in improved patient outcomes with no documented infections.

Conclusions:

Kerecis Omega3 fish skin graft is useful in treating burns and blast wounds in time of war.

P409

Coverage of the hand and forearm in electrical burn

Jung, S. ^1^

^1^ Hanil General Hospital, Seoul, Republic of Korea

Poster presentations 5, 5 September 2025, 16:15–16:45

The hand and forearm are the common points of contact and entry for electrical burns. Wounds from electrical burns can range from small to large and superficial to deep. High-voltage electrical burns result in skin and/or soft-tissue defects owing to progressive necrosis caused by intravascular thrombosis, causing complications in the coverage of electrical burns. Various coverage methods have been used for electrical burns on the hands and forearms. Between 2017 and 2022, 61 patients were admitted for electrical burn treatment on the forearm and hand in our department; and 41 underwent coverage surgery. The range of motion was evaluated when feasible pre-and post-operatively. Among the 41 patients who underwent coverage surgery, 16 and 25 suffered deep second-and third-degree burns, respectively. Wounds of the remaining 20 healed with wound dressings as they were superficial or small.

Patients with deep second-degree burns required various procedures, including full-thickness skin graft (FTSG), advanced local flap, split-thickness skin graft (STSG), and STSG with acellular dermal matrix (ADM). Procedures for patients with third-degree burns include an advanced local flap, STSG with ADM, FTSG, and other flap surgeries. All the wounds healed without major complications. We successfully treated 41 burn wounds using different wound coverage methods, such as STSG or STSG with ADM for deep second-degree burns and flap surgery for third-degree burns. Moreover, in third-degree burns, STSG with ADM could substitute for flap surgery if flap surgery is not performed.

P412

The potential of ischemic modified albumin value in predicting subsequent eschar formation in burn patients.

**Kim, Y.** ^1^, Song, W. ^2^, Park, Y. ^3^, Kwon, M. ^4^

^1^ Han-gang Soo Hospital, Seoul, Republic of Korea, ^2^ Han-gang Soo Hospital, Seoul, Republic of Korea, ^3^ Han-gang Soo Hospital, Seoul, Republic of Korea, ^4^ Han-gang Soo Hospital, Seoul, Republic of Korea.

Poster presentations 5, 5 September 2025, 16:15–16:45

Aim:

Eschar formation in burn patients significantly impacts treatment strategies, duration, and prognosis. Eschar formation is not solely determined by Total Body Surface Area (TBSA) but also by depth, making early prediction challenging. Therefore, we aimed to explore if objective blood test values could serve as predictors for eschar formation.

Methods:

We selected 22 hospitalized burn patients (mean age; 46.9 years, M:F = 12:10, mean TBSA; 6.18) who underwent inflammation-related blood tests from March to September 2023 under the care of one burn surgeon. Inflammatory markers included WBC (White blood cells), lymphocyte, neutrophil, CRP (C-reactive protein), ESR (erythrocyte sedimentation rate), SAA (Serum amyloid A), IMA (Ischemic modified albumin), and IMA/albumin ratio.

Results:

Among the 22 patients, factors influencing eschar formation identified through *t*-tests included WBC, lymphocyte, neutrophil, IMA, and IMA/albumin ratio. Specifically, statistically significant differences were observed for IMA (*p*-value = 0.012) and IMA/albumin (*p*-value = 0.003) when assuming unequal variances.

Conclusions:

Our study highlights the potential significance of IMA and IMA/albumin ratios in predicting eschar formation in burn patients. The statistically significant results of IMA and IMA/albumin ratios suggest their utility as objective markers for identifying patients at higher risk of eschar formation. This emphasizes the importance of incorporating these biomarkers into clinical assessments for burn patients to improve prognostic accuracy and guide treatment decisions.

P413

Beyond tradition: The ethical obligation to choose midline forehead flaps over paramedian forehead flaps.

**Kleintjes, W.** ^1,2^, Prinsloo, T. ^3,4^

^1^ Department of Surgical Sciences, Stellenbosch University, Cape Town, South Africa, ^2^ Western Cape Provincial Adult Tertiary Burns Centre, Tygerberg Hospital, Cape Town, South Africa, ^3^ Department of Biomedical Sciences, Cape Peninsula University of Technology, Cape Town, South Africa, ^4^ Department of Emergency Medical Sciences, Cape Peninsula University of Technology, Cape Town, South Africa.

Poster presentations 5, 5 September 2025, 16:15–16:45

Aim:

Despite positive outcomes with one-stage midline forehead flaps (MFF), the paramedian forehead flap (PFF) remains the gold standard in Western practice. This raises ethical concerns about performing PFF in the interest of the patient, when MFF offers superior results. This study aimed to objectively compare MFF and PFF in order to potentially establish an ethical framework for decision-making.

Methods:

Standard MFF and PFF procedures performed on two patients were visually compared in terms of healing assessed immediately post-operation, at 1–2 weeks, and at 1 month. Objective comparisons included artery number, venous and pedicle landmarks, pedicle length, raw wound extent, skin graft necessity, number of operations, sensory maintenance, patient discomfort, visual obstruction, and cost efficacy.

Results:

Early-stage healing and aesthetics were superior with MFF. Moreover, MFF exhibited greater vascularity, consistent central venous lines, and minimal arterial variation. Pedicle length was longer requiring less expansion, with minimal raw wounds and preserved sensation in the flap. MFF consistently allowed a one-stage procedure, seldomly required a second stage (usually minor), and never required skin grafting, which also demonstrated its cost efficacy. Additionally, patient discomfort was minimized with no visual obstruction and early acceptable outcomes that also allowed spectacle usage shortly after.

Conclusions:

Objective and visual comparisons confirmed MFF as the superior technique, aligning with patient best interest. Therefore, performing PFF when MFF is available is ethically questionable. A paradigm shift in nasal reconstruction is necessary to prioritize patient-centered, evidence-based approaches that enhance both physiological and psychological recovery.

P414

3D Scanning in Evaluating Burn Wound

**Lengyel, P.** ^1^, Eliáš, E. ^1^, Demčák, T. ^1^, Bednarčíková, L. ^2^, Michalíková, M. ^2^

^1^ Hospital Agel Košice Šaca, Košice, Slovakia, ^2^ Faculty of Mechanical Engineering, Technical University of Košice, Košice, Slovakia

Poster presentations 5, 5 September 2025, 16:15–16:45

Aim:

Effective treatment of burn wounds and scars requires accurate evaluation of burn severity and progression, and 3D scanning technologies may provide a valuable alternative to traditional clinical assessments

Methods:

To obtain patient data, ethical approval was first secured, and informed consent was obtained from each patient. The study was approved by the Ethics Committee of AGEL Hospital Košice-Šaca (Slovakia) under the number 17-2023. In addition, a set of questionnaires was developed for doctors and nurses, which covered a wide range of information. For each patient, detailed photographic documentation of the affected area was taken to capture the entire burned tissue area and approximately 2 cm of healthy tissue around the edge of the burn.

Results:

A total of 20 patients with burns of various etiologies, stages, and extents have been monitored, 16 of them were men and 4 women. The main etiology of them—9 patient were scald burns, 7 patient had flame burns, 4 patient.

Suffered burns from explosion. 5 patients needed surgical treatment—necrectomy and skin grafting, the others—9 patient healed without surgery. The mean extent of burn wounds was 13% of TBSA.

Conclusions:

3D scanning allows for the creation of 3D models of burned areas and enables the tracking of their progression over time, providing critical data for evaluating the effectiveness of various treatment methods.

Keywords:3D scanning, burn wound, burn analysis.

Acknowledgement:

This work was supported by the Slovak Research and Development Agency under the contract No. APVV-22-0340.

P416

Polylactic macro-micropore acid dressing in combination with negative pressure therapy in a high voltage electrical burn with bone loss and tendon exposure. Case report and follow-up after a year.

**Lopez, A.** ^1^, Vélez, M. ^1^, Estrada, J. ^1^, Chávez, O. ^1^

^1^ Ciudad de Mexico, México

Poster presentations 5, 5 September 2025, 16:15–16:45

Aim:

Electrical injuries cause deep tissue damage greater than visible skin injury, being more likely to suffer compartment syndromes as well as fractures and amputations during acute admission. Cutaneous covering is always a challenge. Lactic acid may stimulate the healing process by supporting angiogenesis, re-building the dermis, and lowering the pH.

Methods:

A 37-year-old male 4th grade electrical burn with skin loss of the lateral malleolus and burned astragalus that was resected by orthopedic surgeons, leaving a skin and tissue defect. Polylactic acid macro-micropore dressing was applied in addition of negative pressure system therapy repeated twice (14 days) and posterior grafting. A subsequent follow-up at 1 year has been carried out.

Results:

The patient showed radical improvement of granulation tissue and partial epithelialization of skin after 14 days of treatment, including granulation tissue over tendons and bone, with complete closure of the astragalus defect. Leading a viable bed that allowed us to use a splitthickness graft with adequate integration and discharge from hospital 7 days later. After a year he is able to walk independently with the use of an ankle and foot immobilizer and acceptable scar quality and life quality.

Conclusions:

The use of polylactic macro-micropore acid dressing improved the process of granulation of the 4th degree electrical injury and created granulation tissue that allowed the coverage of tendons and bones, decreasing necrotic tissue and local infection. and an acceptable functional and aesthetic result during the one-year follow-up.

Keywords: electrical burns, polylactic acid, macro-micropore dressing.

P420

Ocular chemical burns causing elevated intra-ocular pressure

**Zeng, Y.** ^1^, Tey, K. ^1^, Ong, H. ^2^, Foo, C. ^2^, Tan, K. ^2^, Tan, B. ^2^, Chong, S. ^2^

^1^ MOH Holdings Singapore, Singapore, Singapore, ^2^ Singapore General Hospital, Singapore, Singapore

Poster presentations 5, 5 September 2025, 16:15–16:45

Aim:

Ocular chemical burns represent a severe ophthalmic emergency due to their potential for extensive damage. Alkali agents, such as sodium hydroxide, cause liquefactive necrosis resulting in deeper burns due to the rapid and continued penetration of tissues.

Methods:

A 46-year-old male patient sustained severe ocular burns following caustic soda splash in a workplace accident. Initial assessment revealed pH 8 in both eyes, diffuse corneal oedema, subtotal limbal ischemia, and markedly elevated intraocular pressures (IOP)—Right eye: 74 mmHg, Left eye: 86 mmHg. Immediate saline irrigation was initiated on arrival to achieve a final pH of 7. Treatment included intravenous Acetazolamide, mannitol, and an intensive regimen of topical eyedrops to normalize IOP.

Due to extensive limbal ischemia and persistent corneal epithelial defect (ED), he underwent bilateral amniotic membrane transplantation (AMT) with allogenic simple limbal epithelial transplantation (SLET). Persistent corneal ED necessitated repeat AMT, allogenic-SLET, tenoplasty, tarsorrhaphy and botulinum injection over the subsequent 3 months. The IOP remained stable, allowing for eventual withdrawal of glaucoma drops.

Results:

Commonly reported sequelae of chemical burns typically focus on ocular surface complications such as corneal opacification, scarring, and limbal stem cell deficiency (1). However, early recognition of glaucoma as a complication of severe chemical burns is essential to prevent irreversible optic nerve damage and permanent vision loss (2). This has not been described in any burns literature although it has been reported in ophthalmology journals (3,4).

Conclusions:

Regular IOP monitoring in chemical ocular burns is essential for timely intervention and management of this serious complication.

P423

Histologic changes observed in wound bed after fish graft application

**Aballay, A.** ^1^, Bregg, J. ^1^, Sturges, M. ^1^, Zupsic, L. ^1^, Tolchin, E. ^1^

^1^ Allegheny Health Network, Pittsburgh, United States

Poster presentations 5, 5 September 2025, 16:15–16:45

Aim:

Analyze histologic changes during healing and cosmetic outcome of wound.

Methods:

A 35 year-old male patient experienced a high voltage electrical injury (7200 V) while at work. He presented with a 15% TBSA burns, which were full thickness. Debridement of the left shoulder led to severe loss of tissue. NWPT and fish graft was used over the area to fill in the cavity. The wound bed was biopsied on day 5 and 7.

Results:

Fish graft can help to cover contour defects and fill deep cavities with or without NPWT. The observed changes documented by biopsy indicate that the structure formed within the fish graft is indistinguishable from naturally occurring granulation tissue and it seems to progress at least at the same speed. The wound was followed for over a year and remained closed without requiring additional surgery.

Conclusions:

Fish graft can help fill deep cavities and deficits by facilitating the rapid formation of granulation tissue which help fill in the cavity and improves the long-term appearance. The histology shows a cohesive structure in formation during early healing, and in this patient, areas where fish graft was used have acceptable cosmetic results and good functional outcomes in the long term. Further studies are needed to further characterize this healing

P424

Comparison of Allograft and Fish graft in the management of Deep partial thickness burns

Carver, S. ^1^, **Aballay, A.** ^1^, Niedzielski, N. ^1^, Sommers, S. ^1^

^1^ Allegheny Health Network, Allison Park, United States

Poster presentations 5, 5 September 2025, 16:15–16:45

Aim:

Comparison of Allograft and Fish graft in the management of Deep partial thickness burns in a patient.

Methods:

56 year-old male with 48% TBSA flame burns from a gasoline fire. Bilateral upper and lower extremities were burned circumferentially. Prophylactic escharotomies performed. The wounds were determined to be deep partial thickness bilaterally. Debridement was performed in all the wounds with a dermatome set 10/1000. A side-to-side comparison of allograft (right side) and xenograft (left side) was performed.

Results:

The allograft side developed thicker hypertrophic, hyperpigmented scarring, and increased itching, discomfort and skin markings compared to the fish graft side. In addition, the allograft took overall longer time to heal. Wounds located on the lower right leg remained open for several months requiring additional surgery.

Conclusions:

In this case, the use of fish graft provided at least similar and potentially better results than allograft. Unfortunately, a study designed to compare allografts and xenografts results on the same patients will be challenging.

P425

Advancing burn treatment with epicite hydro^®^: clinical evaluation of healing outcomes in second-degree burns of the face and mobility zones

**Alexandru, A.** ^1^, Mihaela, P. ^1,2^, Marian, B. ^1^, Dan-Cristian, M. ^1,2^, Bianca, A. ^1^

^1^ Department of Plastic Surgery and Burns, County Emergency Hospital “St. Spiridon”, Iasi, Romania, ^2^ University of Medicine “Grigore T. Popa” Iasi, Iasi, Romania

Poster presentations 5, 5 September 2025, 16:15–16:45

Aim:

To evaluate the effectiveness of Epicite Hydro^®^ in promoting healing, reducing infection rates, and improving functional and aesthetic outcomes in second-degree burns of the face and flexion-extension areas, compared to conventional treatments.

Methods:

A retrospective cohort study analyzed 156 patients with second-degree burns. Burn depth was assessed using Laser Doppler imaging. The intervention group was treated with Epicite Hydro^®^, a biosynthetic nanocellulose dressing, while the control group received conventional dressings. Dressings were changed every three days. Primary outcomes included healing time, infection rates, pain and satisfaction levels (measured by standardized scales), hypertrophic scar formation, functional recovery, aesthetic outcomes, and the need for surgical intervention.

Results:

Epicite Hydro^®^ significantly accelerated epithelialization, with a median healing time of approximately 10 days. Patients reported reduced pain during dressing changes. The Epicite Hydro^®^ group demonstrated lower infection rates, likely due to its moisture-retentive properties that maintain an optimal healing environment. Functional outcomes were significantly improved, particularly in flexion-extension areas, with reduced contracture formation and enhanced range of motion. Aesthetic outcomes were superior, with minimal hypertrophic scarring and improved skin texture.

Conclusions:

Epicite Hydro^®^ is an effective treatment for second-degree burns, promoting faster healing, reducing complications, and improving both functional and aesthetic outcomes. Integrating Epicite Hydro^®^ into burn management protocols could decrease the need for surgical interventions and improve patient quality of life.

Keywords: epicite^®^, scar prevention, burn care

P426

Comparison of post operative complications between use of soft base tissue expanders to rigid base tissue expanders in plastic surgery reconstructions—A retrospective study.

Alkalay, I. ^1,2^, Cohen, A. ^1,2^, Shoham, Y. ^1,2^, Silberstein, E. ^1,2^

^1^ Faculty of Health Sciences, Joyce & Irving Goldman Medical School at Ben Gurion University of the Negev, Beer-Sheva, Israel, ^2^ Department of Plastic Surgery, Soroka University Medical Center, Ben-Gurion University of the Negev, Beer-Sheva, Israel.

Poster presentations 5, 5 September 2025, 16:15–16:45

Aim:

Using tissue expanders (TEs) is well-established in reconstructive plastic surgery. While prior studies have investigated diverse aspects of expanders’ complications, there is a paucity of published research regarding specific expander bases. This study aims to investigate the association between the expander base and complications.

Methods:

We conducted a retrospective analysis of non-breast-related tissue expansion procedures in patients aged 6 months to 76 years at Soroka University Medical Center (SUMC) from 2000 to 2021. We compared soft-base and rigid-base TEs, analyzing demographic factors, anatomical placement, shape, and indications using mixed models and multivariate Poisson regression to estimate the risk ratio (RR) and adjusted for confounders.

Results:

86 patients underwent non-breast-related TE procedures, utilizing 186 TE devices, with 130 being rigid-base and 56 soft-base. Of these, 54% of patients with rigid-base TEs and 53% of those with soft-base TEs had post-burn reconstructions.

The groups did not differ in demographic characteristics. Univariate analysis revealed complication rates to be 18% for the soft-base and 50% for the rigid-base group (*p* < 0.001). Another significant difference was found specifically in major complications (16% vs. 45%, *p* < 0.001), including infection and exposure (*p* = 0.045 and *p* = 0.013, respectively).

Conclusions:

An association was found between TE base type and outcomes in reconstructive surgeries, particularly in major complications, demonstrating a significant advantage for using soft-based expanders in non-breast tissue expansion reconstructions.

P427

Use of locoregional block in enzymatic debridement (Nexobrid^®^) of partial-thickness burns on the hands.

**Álvarez Hernández, P.** ^1^, Cerón Molina, F. ^1^, Anaya Pérez, P. ^1^, Martinez Méndez, J. ^1^

^1^ University Hospital La Paz, Madrid, Spain

Poster presentations 5, 5 September 2025, 16:15–16:45

Objective:

To describe the results obtained in the Burn Unit of La Paz University Hospital with the use of locoregional blocks during enzymatic debridement with Nexobrid^®^ in the hands.

Methodology:

A retrospective observational study. Patients with hand burns were selected, and an ultrasound-guided locoregional block was applied to perform enzymatic debridement.

Results:

The use of locoregional blocks reduced the length of hospital stay in these patients. No elevated pain was reported after pain assessment using the VAS scale.

Conclusions:

The use of locoregional blocks for the application of enzymatic debridements such as Nexobrid^®^ has provided good results in terms of pain perception. Local-regional anesthesia allows the same surgeon who applies the product to perform the nerve block, which reduces the time it takes to begin treatment.

P428

Treatment of partial-thickness burns with enzymatic alginogel: experience and protocol in a burn unit.

Álvarez Hernández, P. ^1^, **Cerón Molina, F.** ^1^, Martinez Méndez, J. ^1^

^1^ University Hospital La Paz, Madrid, Spain

Poster presentations 5, 5 September 2025, 16:15–16:45

Objective:

To describe the indications for the use of enzymatic alginogel (Flaminal^®^) for partial-thickness burns in the burn unit of La Paz University Hospital and propose a protocol for its use.

Methods:

A retrospective observational study was conducted in patients with second-degree burns at La Paz University Hospital treated with enzymatic alginogel (Flaminal^®^) between 2021 and 2024.

Results:

Optimal wound epithelialization time was observed.

Conclusions:

The use of Flaminal has reported good results in terms of healing time, infection rates, and sequelae.

P429

Optimizing burn mass casualty response in resource-limited settings: lessons from the colectiv fire and the potential of enzymatic debridement with NexoBrid^®^.

**Oproiu, A.** ^1^

^1^ Bucharest University Emergency Hospital, Bucharest, Romania

Poster presentations 5, 5 September 2025, 16:15–16:45

Burn Mass Casualty Incidents place overwhelming demands on healthcare systems, particularly in countries with limited burn care infrastructure. The 2015 Colectiv nightclub fire in Romania tragically revealed critical gaps in national and regional preparedness, with over 150 burn victims surpassing the country’s specialized care capacity.

Objective:

This article analyzes the management challenges during the Colectiv disaster and explores the role of NexoBrid^®^ as a strategic tool for improving burn care in low-resource, high-demand scenarios.

Methods:

We conducted a retrospective assessment of the Colectiv disaster response in our institution and compared it to later BMCI responses in Europe, such as the Centelles incident in Spain, where NexoBrid was used effectively. Data were contextualized within EU disaster preparedness recommendations and the broader framework of burn care inequities across Europe.

Results:

The Romanian response was hindered by a shortage of burn beds, surgical teams, and intensive care infrastructure. At the time, the EU framework for burn mass casualty response had not yet been established. Subsequent events demonstrated that early use of NexoBrid can reduce the surgical dependency, and operating room demand—key advantages for countries with constrained resources. NexoBrid enables timely eschar removal at bedside, bypassing the initial surgical bottlenecks and expanding surge capacity.

Conclusions:

Integrating enzymatic debridement into national burn disaster treatment protocols offers a scalable solution for countries with limited surgical capabilities. The Colectiv fire underscores the urgent need for improved preparedness in the EU framework, staff training, and access to modern medical countermeasures like NexoBrid to optimize outcomes in future BMCIs.

P430

Literature review of clinical effectiveness, cost-effectiveness and quality of life: enzymatic alginogel versus silver sulfadiazine

Castro Sevilla, A. ^1^, Izquierdo Granadero, B. ^1^, Armendariz Solera, E. ^1^, Moreno Franco, D. ^1^, **Sinovas Rodríguez, M.** ^1^

^1^ Nurse at the Major Burns Unit of the Cruces University Hospital of the Basque Health Service (Osakidetza), Bilbao, Spain

Poster presentations 5, 5 September 2025, 16:15–16:45

Aim:

To describe the scientific evidence of burn treatment comparing enzymatic alginogel versus silver sulfadiazine.

Methods:

A literature review was carried out in which 4 articles were selected from the Pubmed, Cochrane Library and Virtual Health Library databases. They contrast the frequency of treatments, the healing process, the quality of life and the cost-effectiveness ratio. The selection includes two multicenter randomized controlled studies, a retrospective cohort study and a study protocol.

Results:

Studies show that silver sulfadiazine can be toxic to epithelial cells, hindering healing. While enzymatic alginogel does not present toxicity (1, 2). Enzymatic alginogel presents greater rate of colonization without detecting signs of infection or influencing antibiotherapy (2, 3, 4). No significant changes are observed in the perception of pain, restlessness and anxiety. Enzymatic alginogel requires less frequent dressing changes (2, 3). No significant differences were observed in the need for surgery, length of hospital stay, quality of life and cost-effectiveness.

Conclusions:

Studies reviewed show better results with enzymatic alginogel regarding quality of healing and frequency of dressings, reducing exposure to situations of pain, anxiety and discomfort. No differences related to hospital stay time, quality of life and cost-effectiveness. Greater colonization of the burn bed was observed with the use of enzymatic alginogel without affecting the incidence of infection or the use of antibiotics. In our experience, reducing the frequency of treatment sessions is beneficial both for the structuring of the unit’s work and for patient well-being.

P432

Tattoos: an unexpected ally in enzymatic debridement with Nexobrid^®^

**Bayo Montoliu, M.** ^1^, García García, M. ^1^, Guijarro Pérez, C. ^1^, Pérez Plaza, A. ^1^, González Alonso, V. ^1^, Pérez del Caz, M. ^1^

^1^ Plastic and Reconstructive Surgery Department, Hospital Universitari I Politècnic La Fe, Valencia, Spain

Poster presentations 5, 5 September 2025, 16:15–16:45

Aim:

To present a clinical case that illustrates the diagnostic and therapeutic importance of enzymatic debridement with Nexobrid^®^, using the patient’s own tattoos (whose ink pigment lies in the deeper portion of the dermis) to guide therapeutic decisions.

Methods:

We present the case of a 36-year-old male with second-degree burns of 32% TBSA after a work-related accident, including both upper limbs, with numerous tattoos in the burned areas. After initial evaluation, early enzymatic debridement of both upper limbs with Nexobrid^®^ was performed.

Results:

After the application of Nexobrid^®^ for 4 h and its subsequent removal, its selective action on the devitalized tissue was observed, having almost completely erased the tattoos in the areas with deeper burns. However, in the more superficial areas, the tattoos were preserved.

This information allowed us to decide on conservative management of the more superficial areas with wound dressings until epithelization was obtained. For the areas with deeper burns, surgical debridement and autografting was applied. Excellent results were obtained in both areas.

Conclusions:

Nexobrid^®^ enzymatic debridement is a vital therapeutic tool in the early management of the burn patient. Its application and especially the exposure of the viable wound bed when the product is removed provides accurate diagnostic information about burn depth. This, in turn, can be a helpful tool to aid making therapeutic decisions when planning wound closure strategies.

P433

Early experiences of the Spectral MD Deep View wound imaging technology in a UK regional Burns Centre

**Beese, M.** ^1^, Norton, J. ^1^, Bache, S. ^1,2^

^1^ Burns Centre, Queen Elizabeth Hospital, Birmingham, United Kingdom, ^2^ Paediatric Burns Centre, Birmingham Children’s Hospital, Birmingham, United Kingdom.

Poster presentations 5, 5 September 2025, 16:15–16:45

Aim:

To report feedback surveys and product evaluation during the first experience of Deep View wound imaging technology in a Burns Centre.

Methods:

The Deep View combines multispectral imaging with an Artificial Intelligence (AI) model, to predict whether a burn will heal within 21 days. It was tested in the real-world setting of the assessment room in a regional Burns Centre. Clinical assessment and management continued as usual. Blue areas on the Deep View images indicated Non-Healing Areas (NHA).

User feedback was collected. A retrospective product evaluation was performed. Predictions by the technology were compared with clinical outcomes (healing within 21 days or requirement for excision and grafting).

Results:

User questionnaires were completed by 21 respondents (9 doctors, 12 nurses). These demonstrated excellent ease of use with 81% reporting the image was quick and easily understood (19% were neutral). All respondents agreed (52%) or strongly agreed (48%) the software was easy to navigate.

13 burn wounds had no NHA and all healed without surgery. NHA were indicated on 17 burn wounds. Of these, 11 clinical outcomes matched the AI prediction, but 6 healed within 21 days. Overall, sensitivity was 100% and specificity was 68%.

Conclusions:

Our early experience suggests that the Deep View is easy to use with minimal training by all team members. The high sensitivity supports the manufacturer’s findings, suggesting that in our practice it is a useful safety-net triage tool for less experienced practitioners to confirm superficial burns that may be managed in a nurse-led clinic.

P434

Novel 3D Skin Autograft Approaches for Enhanced Burn Wound Healing

**Benshoshan, M.** ^1^, Di Segni, A. ^1^, Cohen Gerassi, D. ^2^, Liaani, A. ^1^, Dothan, D. ^1^, Harats, M. ^1^, Adler-Abramovich, L. ^2^, Haik, J. ^1^

^1^ National Burn Center, Sheba Medical Center, Ramat-Gan, Israel, ^2^ Department of Oral Biology, The Goldschleger School of Dental Medicine, Faculty of Medical and Health Sciences, Tel Aviv University, Tel-Aviv, Israel.

Poster presentations 5, 5 September 2025, 16:15–16:45

Aim:

The objective is to develop an advanced smart skin dressing with a biodegradable scaffold that supports skin cell growth, creating a multilayered, multicellular construct mimicking native skin.

Methods:

Ethical Approval and Patient Consent: All procedures adhered to Israeli law, with informed consent from legal guardians.

Primary Cell Culture: Keratinocytes and fibroblasts were isolated from a patient’s biopsy and cultured for CEA sheet production or skin equivalent formation in vitro.

Grafting: CEA sheets (~30 cm^2^) were grafted onto wound areas, 7 days post-surgery. Keratinocytes in suspension were sprayed with EPNM over deep dermal wounds.

Quality Control: Viability, identity, purity, and potency were assessed using flow cytometry and colony-forming efficiency assays.

In-Vitro Experiments: Biocompatibility of engineered skin equivalents (PCL, PCL-2.5% Fmoc-FRGD, PCL-20% Fmoc-FRGD) was tested using the XTT assay.

In-Vivo Experiments: Mice were treated with various groups: control, PCL + skin cells, PCL-2.5% Fmoc-FRGD + skin cells, and CEA sheet. Wound healing was assessed by visual inspection and histological analysis.

Results:

We developed a personalized treatment for hard-to-heal wounds, using suspended autologous keratinocytes integrated with EPNM, allowing for larger wound coverage compared to traditional CEA methods. Additionally, a novel 3D skin autograft combining patient-specific keratinocytes and fibroblasts in a biodegradable scaffold was developed.

Conclusions:

Our bioengineered skin grafts, integrating electrospun nanofibers and autologous skin cells, support cell growth and tissue regeneration. The spray-on cell therapy offers rapid, effective coverage, overcoming traditional CEA limitations.

P435

Suprathel as a treatment strategy for Toxic Epidermal Necrolysis (TEN)

Braña Campos, S., **Gonzalez Estrella, S.**

^1^ Jose Ramón Martines Méndez, Madrid, Spain, ^2^ Abraham Martel Diaz, Madrid, Spain

Poster presentations 5, 5 September 2025, 16:15–16:45

Toxic epidermal necrolysis (TEN) is an infrequent drug-induced skin disorder, with systemic involvement and characterized by extensive mucocutaneous detachment. In the case presented, the probable pharmacological cause was paracetamol vs. codeine in a patient recently diagnosed with Hodgkin’s Lymphoma. The patient was admitted in intensive care unit with a morbilliform rash affecting over 25% of the total body surface area. On the 17th day of admission, mucocutaneous detachment was 100%.

Currently, there are no guidelines or standard of care specifying the most appropriate material for wound coverage. Toxic epidermal necrolysis is a life-threatening condition requiring early and adequate wound coverage due to the extensive skin defects.

Suprathel is a modern wound dressing that shows promising results when treating superficial wounds such as scalds, burns and abrasions. It is easy to apply, requires minimum dressing changes, which improves patient comfort, less painful, lower risk of hypothermia and infection and promotes rapid healing, achieving satisfactory cosmetic and functional outcomes, and shorten ICU stay.

It would be of great interest to incorporate this methodology into standard treatment to promote wound closure and healing in the treatment of patients with NET.

P436

Beta-sitosterol ointment: a viable non-surgical alternative for burn wound management

**Bukid Abella, M.** ^1^

^1^ Our Lady of Lourdes Hospital, Manila, Philippines, ^2^ University of Santo Tomas, Manila, Philippines, ^3^ Jose R. Reyes Memorial Medical Center, Manila, Philippines

Poster presentations 5, 5 September 2025, 16:15–16:45

Aim:

This study presents Beta-sitosterol as the key component of a burn wound ointment that liquefies necrotic tissue and promotes epithelial regeneration, potentially eliminating the need for early excision and skin grafting in critically ill burn patients.

Methods:

Two patients with high-voltage electrical burns, both prophylactically intubated at the emergency room, were admitted to the ICU of Our Lady of Lourdes Hospital for intensive monitoring.

- Patient 1: A 39-year-old male with 68% TBSA burns underwent bilateral lower extremity escharotomy due to compartment syndrome. Bathing and non-surgical wound debridement under sedation were done twice in the operating room.
- Patient 2: A 43-year-old male with 28% TBSA burns had multiple traumatic injuries, including pubic rami fractures, blunt abdominal trauma, and L5 compression fracture, making surgical debridement unfeasible. Bedside wound debridement and change of dressing were done in the ICU.

Both patients received Beta-Sitosterol ointment applied on all burn wounds about half a centimeter thick every 4 h, after necrotic tissue removal using a wooden tongue depressor and sterile gauze. Epithelialization emerged within 3 to 5 days, and notable tissue regeneration transpired within two weeks.

Results & Conclusions:

Beta-sitosterol in Moist Exposed Burn Ointment (MEBO) supports moisture retention, necrotic tissue liquefaction, and epithelial regeneration, offering a viable alternative to surgical debridement in critically ill patients. Its ability to enhance angiogenesis and growth factor interaction may reduce reliance on skin grafting when systemic conditions prohibit surgery.

Keywords: Beta-sitosterol, regeneration, early excision and skin grafting

P438

Quantifying postburn immune responses: using a computational model to predict scars early

**Bumbuc, R.** ^1,2,3^, Sheraton, V. ^3^, van Zuijlen, P. ^1,4^, Korkmaz, I. ^1,2,4^

^1^ Department of Plastic, Reconstructive and Hand Surgery, Amsterdam Movement Sciences (AMS) Institute, Amsterdam UMC, Location VUmc, Amsterdam, Netherlands, ^2^ Department of Molecular Cell Biology and Immunology, Amsterdam Infection and Immunity (AII) Institute, Amsterdam UMC, Location VUmc, Amsterdam, Netherlands, ^3^ Computational Science Lab, Informatics Institute, University of Amsterdam, UvA—LAB42, Amsterdam, Netherlands, ^4^ Burn Center and Department of Plastic and Reconstructive Surgery, Red Cross Hospital, Beverwijk, Netherlands.

Poster presentations 5, 5 September 2025, 16:15–16:45

Aim:

In this study we develop and validate a computational model to simulate patient-specific inflammatory dynamics and consequently predict their impact on tissue regeneration and scar outcomes in burn wounds.

Methods:

A computational model integrating fibroblasts, myofibroblasts (1), neutrophils (2) collagen subtypes (3), and inflammatory signaling proteins such as IL-6, IL-8, IL-1β, CRP (4) and anti-inflammatory signaling such as TGF-β1, IL-10, C1-inh (5) was created. By using ordinary differential equations (6) we are able to simulate and predict the dynamics and their consequences towards healing (7). Our model simulates 60 days post-injury using clinical parameters such as burn severity, depth, %TBSA burned and patient characteristics such as age and sex. We developed equations to quantify collagen ratio, wound contraction and immunological profiles (8).

Results:

We tested different scenarios (9,10), the computational model predicts cellular and molecular interactions that intervene in the inflammatory and proliferative phase during wound healing. We identified key factors influencing scar formation, such as fibroblast differentiation proportions, collagen maturation dynamics, and sustained inflammatory signaling collectively determining scarring outcomes. Our quantification analysis differentiates between normotrophic healing, hypertrophic scarring, and keloid formation through thresholds in extracellular matrix wound composition. These measurements enable early identification of high-risk scars or keloids within 60 days post-injury.

Conclusions:

This tool provides clinicians with a method for assessing individual scarring risks during early wound healing after burn injury. By correlating inflammatory profiles to tissue-level outcomes, it enhances better understanding of scar formation and facilitates the testing of potential treatments in an in-silico environment.

P439

Enzymatic debridement protocol using bromelain in facial burns: A three-year experience in a major burn unit.

**Cerón Molina, F.** ^1^, Álvarez Hernández, P. ^1^, Anaya Pérez, P. ^1^, Martínez Méndez, J. ^1^, Leyva Rodríguez, F. ^1^

^1^ Hospital Universitario La Paz, Madrid, Spain

Poster presentations 5, 5 September 2025, 16:15–16:45

Aim:

To present the outcomes of a standardized protocol for enzymatic debridement with bromelain in patients with facial burns over the past three years in our major burn unit.

Methods:

We conducted a retrospective review of patients treated with bromelain-based enzymatic debridement for facial burns from January 2022 to December 2024. Data on patient demographics, burn characteristics, time to application, number of sessions, and clinical outcomes were analyzed.

Results:

A total of 47 patients with deep partial-thickness or full-thickness facial burns were treated. The protocol was applied within 72 h post-injury in 86% of cases. Complicarions such as facial infections or uncontrolled bleeding were recorded. Re-epithelialization was observed within an average of 12.5 days. The need for surgical excision and grafting was reduced.

Conclusions:

The implementation of a structured enzymatic debridement protocol with bromelain proved to be safe and effective in the management of facial burns. It allowed for early wound bed preparation, reduced surgical intervention, and preserved aesthetic and functional outcomes. Our experience supports its use as a first-line debridement option in selected facial burn patients.

Keywords: enzymatic debridement, bromelain, facial burns

P440

Efficacy of modern antiseptics against multidrug-resistant *Pseudomonas aeruginosa* strains isolated from burn wounds.

**Chornopyshchuk, R.** ^1,2^, Nazarchuk, O. ^1,2^, Denysko, T. ^3^, Bebyk, V. ^2^

^1^ Vinnytsya Regional Clinical Hospital, Vinnytsya, Ukraine, ^2^ National Pirogov Memorial Medical University, Vinnytsya, Ukraine, ^3^ Odesa National Medical University, Odesa, Ukraine.

Poster presentations 5, 5 September 2025, 16:15–16:45

Infectious complications caused by non-fermenting gram-negative bacteria, particularly *Pseudomonas aeruginosa*, represent a major challenge in burn wound management.

Aim:

To evaluate the antimicrobial activity of antiseptics against clinical multidrug-resistance P. aeruginosa strains isolated from burn wounds.

Methods:

Antiseptics tested included decamethoxin 0.1% (DCM), chlorhexidine 0.05% (CHG), octenidine 0.1% (OCT), polyhexanide 0.1% (PHMB), benzalkonium chloride 0.1% (BAC). Susceptibility testing was performed on both reference and clinical MDR strains of P. aeruginosa using the standard macrodilution method. Minimum inhibitory concentrations (MICs) and minimum bactericidal concentrations (MBCs) were determined for each agent.

Results:

DCM, CHG, and OCT exhibited the highest anti-Pseudomonas activity, with MIC values of 22.52 ± 2.83 µg/mL, 22.5 ± 2.36 µg/mL, and 16.4 ± 2.43 µg/mL, respectively. Corresponding MBCs ranged from 72.09 ± 2.06 µg/mL (CHG) to 102.5 ± 10.75 µg/mL (OCT). No significant differences were observed in the antimicrobial efficacy of DCM, CHG, and OCT; however, all three demonstrated significantly superior activity compared to PHMB and BAC (*p* ≤ 0.01). The bacteriostatic concentrations of DCM, CHG, OCT were 2.9–4 times lower than those of PHMB and 6.5–8.9 times lower than BAC. Complete bacterial killing was achieved at concentrations of 192.5 ± 27.70 µg/mL for PHMB and 418.75 ± 78.89 µg/mL for BAC, which exceeded those for DCM, CHG, and OCT by 1.9–2.7-fold and 4.1–5.8-fold, respectively (*p* ≤ 0.01).

Conclusions:

Multidrug-resistance P. aeruginosa strains isolated from burn wound infections remain susceptible to quaternary ammonium compound-based antiseptics DCM, CHG, and OCT. These agents demonstrate high antimicrobial activity and represent promising components of local treatment strategies for managing resistant burn wound infections.

P441

Burn Induced Compartmental Syndrome: the Role of Enzymatic Escharotomy

**Citterio, A.** ^1^

^1^ Niguarda Hospital, Milan, Italy

Poster presentations 5, 5 September 2025, 16:15–16:45

The enzymatic Escharolis is a therapeutic choice for burn induced compartmental syndrome. Giudelines for surgical esharotomy include pressure greater than 30 mmHg

Methods:

We conducted a study on all patients with circumferential deep partial or full thickness burns of the upper and lower extremity. We performed compartment pressure measurements pre and post procedure.

Results:

In all patients there was a reduction in compartment pressure and the was no need for surgical escharotomy. 70% of these patiens then underwent skin grafting.

Conclusions:

Burn induced compatment syndrome is an acute and complicated condition in deep circumferential burns of the extremities. Surgical treatment is not free from complications and requires an expert surgeon and, often the escharotomy is delayed.

Keywords: bromeline, escharotomy, burns

P442

Advantages and Limitations of Enzymatic Debridement in Burns

**Ciurea, M.**, Nica, O., Marinescu, A., Georgescu, M.

^1^ University of Medicine and Pharmacy Craiova, Craiova, Romania

Poster presentations 5, 5 September 2025, 16:15–16:45

Aim:

This study evaluates the effectiveness and limitations of enzymatic debridement with NexoBrid in the treatment of deep and partial thickness burns.

Methods:

This retrospective study includes three patients hospitalized at the Clinical County Emergency Hospital Craiova with burns of varying degrees and locations. Enzymatic debridement was applied according to the protocol and patient evolution was monitored over several weeks. The analyzed parameters included epithelialization time, skin graft integration, and functional recovery.

Results:

NexoBrid allowed selective debridement, minimising the loss of viable tissue in all three patients. Healing was accelerated and hospitalisation time was reduced. In two cases, complete epithelialization was achieved without the need for grafting. One patient required skin grafts, with initial partial integration but complete healing over time. No major complications were reported, and both aesthetic and functional outcomes were satisfactory.

Conclusions:

Enzymatic debridement is an effective method for burn treatment, reducing the need for surgical excision and accelerating recovery. However, it has limitations in chemical, electrical, or circumferential burns and requires careful pain management. Further studies are needed to optimize its application in different types of burns.

P443

Laser speckle contrast imaging for burn assessment: a comparative accuracy analysis with laser Doppler imaging

**Claes, K.** ^1^, De Mey, K. ^1^, De Decker, I. ^1^, Gush, R. ^2^, Verbelen, J. ^1^, De Coninck, P. ^1^, Monstrey, S. ^1^

^1^ Ghent University Hospital, Ghent, Belgium, ^2^ Moor Instruments Ltd., Axminster, United Kingdom

Poster presentations 5, 5 September 2025, 16:15–16:45

Aim:

To assess the diagnostic validity of Laser Speckle Contrast Imaging (LSCI) against Laser Doppler Imaging (LDI) for predicting burn wound healing potential (HP).

Methods:

In this prospective single-center study, 20 hospitalized burn patients underwent LDI and LSCI between days 2 and 5 postburn. A total of 50 wounds (112 ROIs) were analyzed from the LDI perspective, and 52 wounds (130 ROIs) from the LSCI perspective. Color-coded regions were matched for comparison, with LDI as the reference.

Results:

Only wounds expected to heal within 14 days (HP < 14) showed strong LSCI predictive value (AUC = 0.93). Diagnostic accuracy for deeper wounds (HP14–21 and HP > 21) was poor (AUC < 0.5), with significant overlap in flux distributions and low correlation coefficients. LSCI systematically underestimated deep dermal perfusion, likely due to its limited penetration depth of ~1 mm compared to LDI’s 2 mm. LSCI misclassified up to 20% of ROIs at low perfusion thresholds, potentially leading to overtreatment.

Conclusions:

Although LSCI offers logistical advantages (faster scan time, portability), it lacks the depth sensitivity required for reliable burn assessment. LDI remains the gold standard for HP evaluation and surgical decision-making. Further refinement of LSCI technology is needed before clinical adoption in burns can be considered.

P444

Case Series: How Psychiatric Conditions alter the Management of Burnt Patients

**Bodog, R.** ^1,2^, Bodog, F. ^1,2^

^1^ Bihor Emergency Clinical County Hospital, Oradea, Romania, ^2^ Faculty of Medicine and Pharmacy, University of Oradea, Oradea, Romania

Poster presentations 5, 5 September 2025, 16:15–16:45

Aim:

The aim of this study is to highlight the need of particular guides for the management of patients that present with burn injuries concomitant with psychiatric comorbidities (autism, schizophrenia, dementia).

Methods:

A case report study was performed including burnt patients admitted on the Plastic Surgery Unit. An extended history of the patients was collected regarding their psychiatric treatment and psychiatric reevaluation was requested periodically. Their behavior during the process of dressing-changing was observed together with how long their new dressing lasted and a general daily assessment of their state and pain.

Results:

We present four cases of concomitant burn and psychiatric comorbidity in association with different complications. They had no (one out of four) or multiple (three out of four) baseline comorbidities and all were admitted in the hospital for further management, one of them dying after the skin grafting process. One wasn’t cooperative during the dressing-changing process and needed tailored approaches in order to achieve an adequate result. After further psychiatric evaluation one of them had treatment modifications.

Conclusions:

Concomitant severe burns and psychiatric conditions can complicate the patient’s management and subsequently their recovery. This combination mandates for the medical staff to adjust their approach accordingly, with very little specialist training regarding psychiatric patients, and can be associated with a higher economic cost. The high association between psychiatric comorbidities and burn injuries show reason for developing guides to help medical staff understand how to approach these cases.

P445

Co-creation of a patient-centered evidence-based website for pathological scarring: enhancing patient and clinician engagement and health experience

**Maertens, K.** ^1,2^, Moortgat, P. ^1^, Van Loey, N. ^1^, Demarbaix, T. ^1,3^, Meirte, J. ^1,3^

^1^ OSCARE, Antwerp, Belgium, ^2^ Vrije Universiteit Brussel (VUB), Brussels, Belgium, ^3^ University of Antwerp (UA), Antwerp, Belgium

Poster presentations 5, 5 September 2025, 16:15–16:45

Aim:

This study aimed to evaluate and optimize an evidence-based website on pathological scarring by redesigning its content and structure through co-creation with patients and clinicians.

Methods:

We conducted three semi-structured focus group sessions. One focus group, consisting of 7 patients with scars and 3 caregivers (1 carer and 2 healthcare clinicians), held two group sessions. A third focus group session involved 7 different patients and was organized to achieve data saturation. Audio-recordings were transcribed, and thematic analysis was performed to identify key-themes from the discussions.

Results:

Thematic analysis revealed six highly valued characteristics: (1) personal choice and preference, (2) clear, realistic, and recognizable information, (3) use of videos and visuals depicting scars and treatments, (4) consideration of psychosocial aspects, and (5) inclusiveness. An additional patient focus group confirmed these themes and added a sixth theme: communication. Participants requested simplified language, enhanced peer support, and instructional videos on self-management. Clinicians emphasized improvements in the website’s visual structure, classifications, and treatment options. All stakeholders recommended developing a separate platform for children and youth. These insights led to the transformation of the initial website (www.howtotreatscars.com) into a new version (www.myscarspecialist.com).

Conclusions:

This study provides valuable insights and highlights the importance of co-creation in improving online health information and patients’ experience. The enhanced website addresses patients’ and clinicians’ needs by improving accessibility, clarity, and usability. Future research should assess the effectiveness of these changes in diverse healthcare settings and explore further refinements based on user engagement data.

P447

Suicidal inpatients aged 18–28 years in the National Burn Centre compared with same-aged general population.

**Palmu, R.** ^1^, Purola, L. ^1^, Vuola, J. ^1^

^1^ Helsinki University Hospital, Helsinki, Finland

Poster presentations 5, 5 September 2025, 16:15–16:45

Aim:

To collect data on self-harm in burn patients treated in a burn center, and to compare the patient characteristics and suicidality with a nationwide same-aged sample of the general population.

Methods:

We investigated the medical records of acute patients admitted to the National Burn Centre (NBC) in Helsinki, Finland, due to burn injuries by self-harm or attempted suicide during the years 1989–1997 and 2011–2020. For further analysis the 18–28-year-old patients (*n* = 13 and *n* = 14, respectively) of both cohorts were compared to a random sample of the 18–28-year-old general population from the nation-wide Health 2011 Survey in Finland. Suicidality and psychiatric background were investigated.

Results:

Psychiatric disorders were recorded in half of the cases in both self-harm and suicidal samples. In the NBC 1989–1997 sample more than two-thirds (76.9%, *n* = 10/13) had pre-burn psychiatric disorder and care. In the NBC 2011–2020 sample 50% (*n* = 7/14) psychiatric disorder and 42.9% (*n* = 6/14) care. In the reference sample (of general population) 16.4% (*n* = 137/834) of the reported having had at least one psychiatric disorder in lifetime, 12.2% (*n* = 14/115) psychiatric care, 17.9% had earlier suicidality, and 6.3% had attempted suicide at least once.

Conclusions:

Psychiatric history is common among self-harm and suicidal young burn patients, but it exists also in the same-aged suicidal general population.

P448

Impact of psychiatric diseases on the management of major burn patients

**Tissieres, I.** ^1^, Léger, B. ^2^, Iakova, M. ^1^, Lecroc, S. ^1,2^

^1^ Clinique Romande de Réadaptation, Sion, Switzerland, ^2^ Service de recherche des cliniques SUVA, Switzerland

Poster presentations 5, 5 September 2025, 16:15–16:45

Aim:

The aim of this study was to evaluate the impact of psychiatric diseases on the management of major burn patients during their rehabilitation.

Methods:

We collected demographic data, ICD-10 psychiatric diagnoses, TBSA, mechanism of injury, total length of hospital stay, pain severity, kinesophobia and levels of engagement in therapies for patients hospitalized between 2013 and 2023.

Results:

41 participants were included and divided in two groups: those with (*n* = 17) and without (*n* = 24) psychiatric disease at the time of injury. In the first group, main diagnoses were mood (9), psychotic (6), borderline (4), addiction (5) disorders, and five of them had multiple diagnoses. For 11 participants, the injury resulted from a suicide-attempt. Age, gender, TBSA, total length of hospital stay, pain severity and kinesiophobia scores were similar between both groups. However, the level of engagement varied between the two groups. In the group without psychiatric disorders it was moderate to low for 21%, mainly due to pain and fatigue. In the other group it was at 35%, primarily due to reasons related to the symptoms of the psychiatric disease.

Conclusions:

Patients referred to burn units often present a variety of psychiatric issues that can even be the cause of the injury. The benefits of rehabilitation may be limited if patients are not fully engaged in the process. To achieve better care, well-resourced psychological teams in burn units are needed and should work collaboratively using an integrative care approach.

P449

Patient-reported outcome measures in Dutch burn patients: response and outcomes from the Burn centres Outcomes Registry The Netherlands (BORN)

**van Dammen, L.** ^1,2,3^, Boekelaar, A. ^2^, van Zuijlen, P. ^2^, Kolstein-Moerland, M. ^1^, van der Vlies, K. ^1^, Bijker, G. ^3^, Scholten-Jaegers, S. ^3^, van Baar, M. ^1^, on behalf of the Burn centres Outcomes Registry The Netherlands (BORN) group

^1^ Maasstad Hospital, Rotterdam, The Netherlands, ^2^ Red Cross Hospital, Beverwijk, The Netherlands, ^3^ Martini Hospital, Groningen, The Netherlands

Poster presentations 5, 5 September 2025, 16:15–16:45

Aim:

To assess the response and outcomes reported by Dutch burn patients who are part of the outcome registry.

Methods:

All adult patients admitted to one of the three Dutch burn centres (Beverwijk, Groningen, Rotterdam) for >24 h and/or had surgery, were eligible to participate in the outcome registry (BORN), which started in 2018. The outcome registry contains different Patient-Reported Outcome Measures (PROM’s) that patients fill out at discharge and at two weeks, three months, and one year after discharge from the burn centre. Reasons for non-participation in the outcome registry were registered. Patient characteristics and clinical variables were extracted from the clinical registry (the Dutch Burn Repository R3). A subset of outcomes was analyzed for this study, including quality of life (QoL), depressive symptoms, and post-traumatic stress disorder (PTSD) symptoms.

Results:

In 2023 a total of *n* = 479 patients were eligible to fill out PROM’s in the three Dutch burn centres. About one-third of this group completed at least a PROM set at one time point (*n* = 149, 31%). Within this group *n* = 76 completed the questionnaires at all time points. Reasons for non-participation included: language limitations, not willing to fill out questionnaires, and psychiatric comorbidities. Reported QoL and PTSD symptoms improved over time, while depressive symptoms did not show a clear pattern over time.

Conclusions:

The response rate to a PROM registry in Dutch burn patients was 31%, with QoL and PTSD symptoms improving over time.

P451

PROSA: An essential guide in the management of medical procedures in children

Stas, H. ^1^, **Hofland, H.** ^1^, van Baar, M. ^3^, Baartmans, M. ^1^, Leroy, P. ^2^

^1^ Burn Centre, Maasstad Hospital, Rotterdam, Netherlands, ^2^ Maastricht University Medical Centre, dep. pediatric intensive care, Maastricht, Netherlands, ^3^ Alliance of Dutch Burn Care (ADBC), Burn Centre, Maasstad Hospital, rotterdam, Nederland.

Poster presentations 5, 5 September 2025, 16:15–16:45

Aim:

What means PROSA: PROcedural Sedation and Analgesia, represents respect, connection, and trust. Avoiding fear and preserving the child’s trust, the approach is multifactorial.

Methods:

Procedural comfort care refers to an integrated care model that aims to ensure short-term comfort and success during a medical encounter while promoting long-term trust in healthcare providers from both the child and parents. Three key factors are essential; pain, anxiety and attentional focus.

Considering these three domains simultaneously help to identify effective interventions for procedural distress. Throughout the entire healthcare process, traumatic experiences are minimized by considering comfort as at least equally important as the actual medical diagnostic or therapeutic procedure. By fully integrating procedural comfort care into medical practice, a new form of comprehensive care emerges—one that continuously combines caring care with healing care.

Seven complementary strategies are used to achieve procedural comfort care: the 7P model.

Evaluating the Procedure: What does this procedure mean for this child?

Prevention: Avoiding unnecessary procedures.

Psychology and Cognition: Strategies that focus on reducing/avoiding fear and building trust.

Pain Management: Timely and effective pain management.

Process and Environment: Navigating a child’s emotions and attention from fear to trust can be disrupted by environmental factors.

Procedural Sedation: Sedative medications may be necessary.

Post-Procedure Care: debriefing, framing, affirming, and constructing a positive memory.

Results/Conclusions:

PROSA gives tools to manage medical procedures in children.

P452

Effects of an Aftercare Programme on Post-Burn Pain and Physical Role Functioning: A Randomised Controlled Trial.

**Bayuo, J.** ^1^, Wong, F.

^1^ The Hong Kong Polytechnic University, Hong Kong, Hong Kong

Poster presentations 5, 5 September 2025, 16:15–16:45

Aim:

To evaluate the effects of an aftercare programme on post-burn pain and physical role functioning.

Methods:

A randomised controlled trial approach was employed. Sixty adult burn survivors were randomly assigned to intervention and control groups. Participants in the intervention group received the aftercare programme which commenced at discharge and included undertaking pain and physical role assessment, formulating a plan of care with each participant, delivering both pharmacological and non-pharmacological interventions, offering ongoing health education, and providing home-based exercise support over an 8-week period. The Brief Pain Inventory (BPI) and the Disability of the Arm, Shoulder and Hand Symptom Scale (DASH) were used to evaluate pain and physical role functioning respectively. Data were collected at three timepoints: baseline (T0), post-intervention (T1), and 4 weeks follow-up (T2). Generalised estimating equation (GEE) was employed to ascertain the group, time, and interaction effects.

Results:

Using Bonferroni corrected *p* value, statistically significant findings were observed regarding pain intensity and physical role functioning at T1 which were sustained at T2. Regarding pain interference, however, we observed non-significant findings at T1 but statistically significant at T2.

Conclusions:

A comprehensive aftercare programme is helpful at improving post-burn pain and physical role functioning.

P453

Investigation of the Nociception Level (NOL™) Index for Pain Assessment in Intensive Care Units for Burn Patients: A Prospective Study

**Boptsi, A.** ^1^, Lopez, J. ^1^, Stang, F. ^1^, Boos, A. ^1^, Schleußer, S. ^1^

^1^ Plastic Surgery Clinic, UKSH Campus Lübeck, Lübeck, Germany

Poster presentations 5, 5 September 2025, 16:15–16:45

Aim:

To evaluate the effectiveness and feasibility of the Nociception Level (NOL) index in assessing pain in burn patients and its correlation with the Numeric Rating Scale (NRS) and Behavioral Pain Scale (BPS).

Methods:

This prospective cohort study was conducted in a burn- ICU at the university clinic in Lübeck, Germany. Burn patients requiring moderate to deep sedation were included. Nociception was assessed using the NOL index alongside standard monitoring during painful interventions (wound dressing changes, dermabrasion, endobronchial aspiration/bronchoscopy, and patient mobilization).

Results:

NOL values were significantly higher during nociceptive procedures compared to baseline and post-procedure measurements, indicating its sensitivity in detecting pain. Furthermore, the NOL index showed a strong correlation with self-reported pain intensity, reinforcing its potential as an objective pain assessment tool in burn patients.

Conclusions:

Pain and sedation assessment in critically ill burn patients remains a significant challenge, as traditional clinical parameters are subjective and prone to variability. Currently, there is no standardized, objective method for accurately assessing pain in this patient population. Our findings suggest that NOL monitoring offers a reliable and objective measure of nociception, complementing existing pain assessment tools. Integrating NOL monitoring into clinical practice could improve analgesic titration, optimize pain management strategies, and ultimately enhance patient outcomes in burn care.

P454

Pain Evaluation with Epicite Hydro Nanocellulose Substitute Versus Conventional Cure in Adult Patients with Deep Second-Degree Burns

**Chau Ramos, E.** ^1^, Wiegering Cecchi, G., Chau Ramos, C., Salcedo Molina, G.

^1^ Clínica Skin Medical, Lima, Peru

Poster presentations 5, 5 September 2025, 16:15–16:45

Objective:

Pain evaluation using nanocellulose substitute (EPICITE HYDRO); versus conventional cure in deep second degree burns in adult patients.

Materials and methods:

The study is an analytical, retrospective intervention type through clinical history, comparative. 142 patients of both sexes participated, aged between 30 and 75 years, treated in an outpatient clinic between January 2022 and July 2024 at the skin medical clinic. All had deep intermediate second-degree burns, affecting less than 20% of the exposed dermis. The VAS scale was used as a pain measurement for both groups and the measurements were taken at 24 h, 72 h, one week, three weeks.

Results:

Indicated in averages with the pain scale and compared between both groups, nanocellulose (Epicite Hydro) versus conventional cure. Reflecting lower pain averages in the nanocellulose group in the follow-up period compared to conventional treatment. Giving special attention to an improvement in pain in the first 24 h and in a sustained manner in the measurements taken after 72 h, one week and three weeks, corroborating with the statistically significant results (*p* 0.05).

Conclusions:

Improvement in pain was evident with the VAS scale, with the use of the nanocellulose substitute. The existence of mild pain stands out for the group that used nanocellulose in contrast to the moderate pain that was characteristic in cases with conventional cure treatment.

P456

Fast & Furious: role of sublingual sufentanil on managing dress change in burn patients

**Lanza, A.** ^1^, Coletta, F. ^1^, Tomasello, A. ^1^, Foreste, G. ^1^, Mataro, I. ^2^, Petroccione, C. ^2^, Rinaldi, P. ^1^, Baldassarre, D. ^1^, Villani, R. ^1^

^1^ Cardarelli Hospital-Burn Intensive Care Unit, Naples, Italy, ^2^ Burn Unit and Plastic Surgery, Naples, Naples

Poster presentations 5, 5 September 2025, 16:15–16:45

Aim Evaluation of safety and evidence of pain control about subilingual sufentanil, a new painkiller, for procedural acute pain in dressing change in severe burn adult patients

Methods preliminary study included nine adult patients (5M, 4 F, mean age 55) with a TBSA > 30%. Before dressing change (10–20 min) pts received 30 mcg of sublingual sufentanil monitoring main parameters (HR, RR, SpO2%) and rescue analgesia if required made by acetaminophene available.

Measure of Pain was made by NRS scale (numeric rating scale) immediately after dressing change (T1) and 3 (T2) and 6 (T3) hours after procedures-We studied pts over 5 dressing change days.

Results Mean NRS score was 2.5 at T1, 3 at T2 and 2 at T3- rescue analgesia required by 2 pts, and safety was obtained in all pts and one adverse effect, nausea, managed with ondansetron. All procedures was obtained before lunch and nobody of them skipped the meal.

Conclusion Pain relief and safety of sublingual sufentanil as well as a fast track use in intensive, subintensive and normal ward or even in emergencies could be the new weapon against procedural pain in burn patients.

P458

Pain management in burned patients by Emergency Medical Teams in Poland—a retrospective analysis of 37,596 cases

**Marzec, L.** ^1^

^1^ Kikgel, Sandomierz, Poland, ^2^ Janusz Piotr Sikora, Lodz, Poland, ^3^ Jakub Karawani, Warsaw, Poland

Poster presentations 5, 5 September 2025, 16:15–16:45

Every burn causes pain that requires relief. Aim: Analyse of the pain management by ambulance medical staff in burned patients.

Methods:

Ambulance interventions due to burns in Poland in the period 1 January 2018–31 December 2023 were analysed. Data were analysed in terms of patient gender, age, and type of burn. Pain treatment was analysed in terms of the use of pain scales and analgesics used.

Results:

There were 37,596 ambulance interventions due to burns. In the study group of 37,596 patients, the age of the patient was indicated in the medical records of 36,794 (97%). There were a predominance of men (21,691, 63%), adults (23,421, 63%,) over children (13,373, 36%) and thermal burn patients (33,884, 90.13%). The pain intensity scale was used for 21,361 (57%) patients: NRS (15,730, 74%), FACE (4900, 23%) and VAS (731, 3%). Pain in the range of 1–10 points was experienced by 19,295 patients (90%). Severe pain (81,576/19,295, 43%) and treatment with one non-opioid analgesic (11,476/22,296, 52%), especially with the use of Acetaminophen (5248, 46%) were predominated.

Conclusions:

1. Burns continue to be a significant public health problem, affecting adults and men more frequently. 2. Ambulance are called more frequently to pateints who have suffered a thermal burn and who are in severe pain. 3.The most common pain management strategy for patients with burns is still the administration of a single analgesic- Acetamonophen.

P459

Modulation of Pain in Burn Injuries Through Lactate-Mediated Mechanisms: A Mechanistic Review of Polylactic Acid Membranes Clinical and Biological Impactpo

**Ramirez-Garcialuna, J.** ^1,2^, Martinez-Jimenez, M. ^2^

^1^ Mcgill University, Montreal, Canada, ^2^ Universidad Autonoma de San Luis Potosi, San Luis Potosi, Mexico

Poster presentations 5, 5 September 2025, 16:15–16:45

Aim:

To explore the clinical and mechanistic evidence supporting pain reduction in burn patients treated with polylactic acid (PLA) membranes, emphasizing lactate-mediated modulation pathways.

Methods:

This mechanistic review integrates data from randomized clinical trials (RCTs), in vivo models, and molecular studies assessing the biological effects of PLA membranes in burn care. Particular focus was placed on its influence on inflammatory modulation, neuroreceptor signalling, and the wound microenvironment.

Results:

In RCTs, PLA membranes have consistently demonstrated rapid and sustained analgesia in partial-thickness burns and donor site wounds, with pain reduction observed as early as the first dressing application. Mechanistically, its degradation product—lactate—exerts modulatory effects through several pathways: (1) acidification of the wound bed, potentially desensitizing nociceptors; (2) modulation of TRPV1 receptor activity, decreasing neurogenic transmission and inflammation; (3) downregulation of pro-inflammatory cytokines, such as IL-1β and TNF-α; and (4) support of cell survival under hypoxia, indirectly stabilizing the healing niche. These combined effects contribute to a less inflamed, less painful wound microenvironment.

Conclusions:

Compelling mechanistic evidence supports the clinical analgesic effects of PLA membranes. Lactate, traditionally seen as a metabolic byproduct, plays a bioactive role in reducing nociception and inflammation. These findings support the routine use of PLA membranes not only as a skin substitute but also as a pain-modulating intervention in burns.

P461

Bibliometric analysis of burn pain assessment tools.

**Erden, S.** ^2^, Tuncbilek, Z. ^1^, Acikgoz, F. ^1^

^1^ Hacettepe University, Ankara, Turkey, ^2^ Cukurova University, Adana, Turkey.

Poster presentations 5, 5 September 2025, 16:15–16:45

Aim:

In this study, we used bibliometrics to explore the literature on burn pain assessment tools, with aim of identifying research progress and predicting future research hot spots.

Methods:

This study was performed as a bibliometric analysis. The Web of Science Core Collection database was retrieved on 30 March 2025. The search strategy was (TS = (“burn pain” OR “pain after burn” OR “burn injury pain” OR “post-burn pain”) And TS = (“pain assessment” OR “pain measurement” OR “pain scale” OR “pain questionnaire” OR “pain intensity scale” OR “pain evaluation”)). This analysis included studies published between 1989 and 2024, written in English. VOSviewer software was used for mapping quantitative data on countries, journals, and keywords.

Results:

A total of 30 articles on burn pain assessment were identified, with 21 incorporating assessment tools. These tools varied across adult and pediatric patients, including VAS, NRS, McGill Pain Questionnaire, Neuropathic Pain Scale, and others. Burns (*n* = 7) and Journal of Burn Care & Research (*n* = 3) were key journals. The USA (*n* = 9) and Australia (*n* = 5) were the most prolific contributors among 11 countries. The University of Washington had the highest citation impact (*n* = 157) with four documents. Patterson, D was the most productive author, while Laterjet, J and Choinere, M (1995) received the highest citations (*n* = 104). Keyword analysis revealed a focus on burns, pain, pain management, and burn pain.

Conclusions:

The results of the study can provide a valuable resource for researchers working in the field of post-burn pain to understand key elements of the topic and related trends.

P462

Post-burn pruritus: prevalence, documentation, and treatment outcomes in inpatients and outpatients.

**Sahib, Y.** ^1^, Fawzi, M. ^1^, Markeson, D. ^1^

^1^ Chelsea & Westminster Hospital, London, United Kingdom.

Poster presentations 5, 5 September 2025, 16:15–16:45

Aim:

To evaluate the prevalence, documentation, and management of post-burn pruritus in inpatients and outpatients and assess the effectiveness of antihistamines and gabapentin in symptom control.

Methods:

A retrospective cohort study was conducted on 100 burn patients (50 inpatients and 50 outpatients) treated between January and February 2024. Medical records were reviewed to determine the frequency of pruritus, treatment interventions, and documentation practices. Antihistamines were prescribed as the first-line therapy, with gabapentin introduced if symptoms persisted. Treatment efficacy and documentation consistency were analysed.

Results:

Of 100 patients, pruritus was documented in 4 out of 18 inpatients assessed and 3 out of 14 outpatients. Antihistamines were the initial treatment, but where ineffective, gabapentin was administered, demonstrating notable symptom relief. However, inconsistent documentation practices were identified, limiting a comprehensive understanding of pruritus prevalence and treatment efficacy. Despite its impact on patient quality of life, pruritus often remained underreported, highlighting a need for standardised assessment and recording.

Conclusions:

Post-burn pruritus is a prevalent but under-recognised complication, often inadequately documented, which may hinder effective management. While antihistamines are the primary treatment, gabapentin serves as an effective second-line therapy. Standardised documentation and further research into alternative treatments and long-term outcomes are crucial for optimising care.

